# Supplementary material for: Synthesis of arylamines and N-heterocycles by direct catalytic nitrogenation using N2
Source: Nat Commun. 2021 Jan 11;12:248. doi: 10.1038/s41467-020-20270-5 (PMC7801372; doi:10.1038/s41467-020-20270-5)
Supplement: Supplementary file 1 — Supplementary Information [file 41467_2020_20270_MOESM1_ESM.pdf]

Synthesis of Arylamines and N-Heterocycles by Direct catalytic Nitrogenation using N_2

Wang, et al.

Supplementary Information

Synthesis of Arylamines and N-Heterocycles by Direct catalytic Nitrogenation using N_2

Kai Wang,[a] Zi-Hao Deng,[a] Si-Jun Xie,[a] Dan-Dan Zhai,[a] Hua-Yi Fang,[a] and Zhang-Jie Shi*[a,b]
Department of Chemistry, Fudan University, Shanghai 200433; State Key Laboratory of Organometallic Chemistry, Chinese Academy of Science, Shanghai 200032, China
E-mail: zjshi@fudan.edu.cn, hfang@fudan.edu.cn

Table of Content

I	General Information	
II	Supplementary Tables	
III	Substrates	
IV	Synthesis and Analytical Data for Products	
V	NMR Spectral Data for Products	
VI	IR Spectral Data for Products	
VII	GPC Spectral Data for Polyaniline Products	
VIII	HRMS Spectral Data for Products	
IX	Proposed Mechanism	
X	Supplementary References	

(I) General Information

Unless noted otherwise, all the reactions were carried out through *General Procedure* under nitrogen atmosphere using standard Schlenk technique and heated in the sand bath. Dioxane was distilled under nitrogen from the sodium ketyl of benzophenone. The catalyst $Pd_2(dba)_3$ and Phosphine ligands, such as 2-dicyclohexylphosphino-2',6'-diisopropoxybiphenyl(Ruphos) and butyldi-1-adamantyl phosphine(Ad_2P^nBu) purchased from Stream were used as received without further purification. ^{1}H NMR (400 MHz)/^{13}C NMR (100 MHz) were registered on Bruker 400 M spectrometers with $CDCl_3$ or DMSO-d_6 as solvent and tetramethylsilane (TMS) as internal standard. Data are presented as follows: chemical shifts were reported in parts per million (ppm, δ), downfield from tetramethylsilane (TMS, δ=0.00 ppm) and were referenced to residual solvent ($CDCl_3$, δ=7.26 ppm (^{1}H) and 77.00 ppm (^{13}C); DMSO-d_6, δ=2.50 ppm (^{1}H) and 39.50 ppm (^{13}C)). All the ^{19}F chemical shifts and ^{15}N chemical shifts were not referenced. Coupling constants were reported in Hertz (Hz). Data for ^{1}H NMR spectra were reported as follows: chemical shift (ppm, referenced to protium, s = singlet, d = doublet, t = triplet, q = quartet, dd = doublet of doublets, td = triplet of doublets, ddd = doublet of doublet of doublets, hept = heptet, m = multiplet, coupling constant (Hz), and integration). Column chromatography was performed on silica gel 200-300 meshes. HRMS were performed by the State-authorized Analytical Center in Shanghai institute of Organic Chemistry, Chinese Academy of Sciences. IR were performed by the State-authorized Analytical Center in Shanghai institute of Organic Chemistry and Research Center for Molecular Recognition and Synthesis Department of Chemistry, Fudan University. GPC were performed by Changchun Institute of Applied Chemistry Chinese Academy of Sciences. Compounds **4b** to **4d**, **8**, **14**, **16** were prepared according to the previously reported literatures. All the other compounds purchased from J&K or Bide Pharmatech Ltd. were used as received without further purification.

(II) Supplementary Tables

Supplementary Table 1. Control experiments

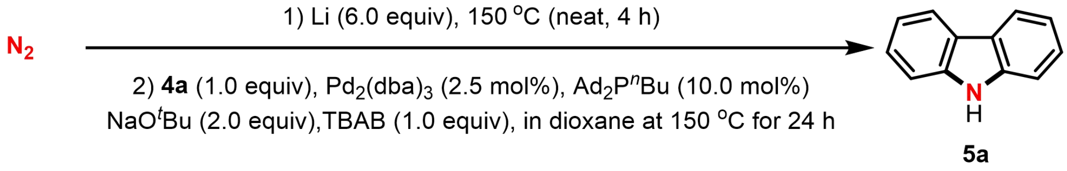

entry	deviation	Yield [%][a]
1	none	84
2	no $Pd_2(dba)_3$	0
3	no Ad_2P^nBu	0
4	no NaO^tBu	12
5	no TBAB	46
6	Ar instead of N_2 in 2)	82
7	Ruphos of Ad_2P^nBu	78
8	PPh_3 instead of Ad_2P^nBu	57
9	toluene instead of dioxane	44
10	no solvent	0
11	25 °C instead of 150 °C	0
12	80 °C instead of 150 °C	14
13	115 °C instead of 150 °C	71

[a]The reaction was run through one-pot/two-step : (i) Li powder (3.0 mmol) under N_2 at 150 °C for 4 h. (ii) **4a** (0.50 mmol), $Pd_2(dba)_3$ (0.0125 mmol), Ad_2P^nBu ligand (0.05 mmol), NaO^tBu (1.0 mmol), TBAB (0.50 mmol) and dioxane (2.0 mL) were added sequentially into the above (i) under N_2 at 150 °C for 24 h. **4a** is 2,2'-dibromobiphenyl. The isolated yields were based on **4a**.

Supplementary Table 2. Mercury (0) poisoning tests

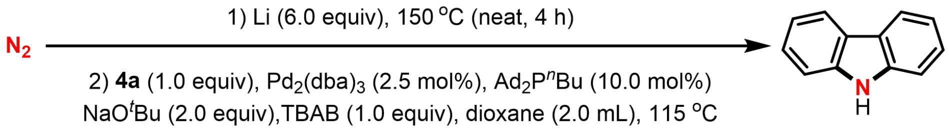

5a

entry	t (h)	Additive	Yield (%)[a,b]
1	0.25 h	/	22
2	4.5 h	/	59
3	4.5 h	Hg[c]	27
4	12 h	/	67
5	12 h	Hg[c]	30

[a]Reactions were carried out through one-pot/two-step : (i) Li powder (6.0 mmol) under N_2 at 150 °C for 4 h. (ii) **4a** (1.0 mmol), $Pd_2(dba)_3$ (0.025 mmol), Ad_2P^nBu ligand (0.10 mmol), NaO^tBu (2.0 mmol), TBAB (1.0 mmol) and dioxane (2.0 mL) were added sequentially into the above (i) under N_2 at 115 °C for t h. **4a** is 2,2'-dibromobiphenyl. [b]Yields was determined by ^{1}H-NMR using 1,3,5-trimethoxylbenzene as the internal standard. [c]Mercury (15 mmol) was added after 15 min of the step (ii) start.

Supplementary Table 3. ^{15}N-labelling experiments

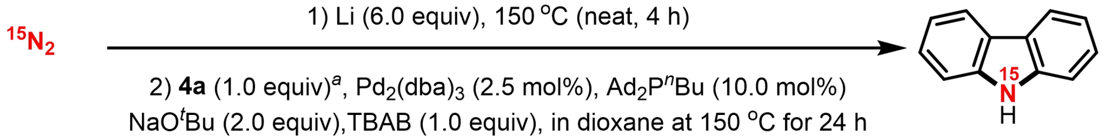

^{15}N-5a

entry	Atmosphere in step 2)	Yield of ^{15}N-5a (%)[a,b]	Yield of 5a (%)[a,b]
1	Ar	76	N.D.[c]
2	N_2	73	N.D.[c]

[a]Reactions were carried out through one-pot/two-step : (i) Li powder (3.0 mmol) under $^{15}N_2$ at 150 °C for 4 h. (ii) **4a** (0.50 mmol), $Pd_2(dba)_3$ (0.0125 mmol), Ad_2P^nBu ligand (0.05 mmol), NaO^tBu (1.0 mmol), TBAB (0.50 mmol) and dioxane (2.0 mL) were added sequentially into the above (i) at 150 °C for 24 h. **4a** is 2,2'-dibromobiphenyl. **5a** is carbazole. [b]Isolated yields. [c]not detected.

(III) Substrates

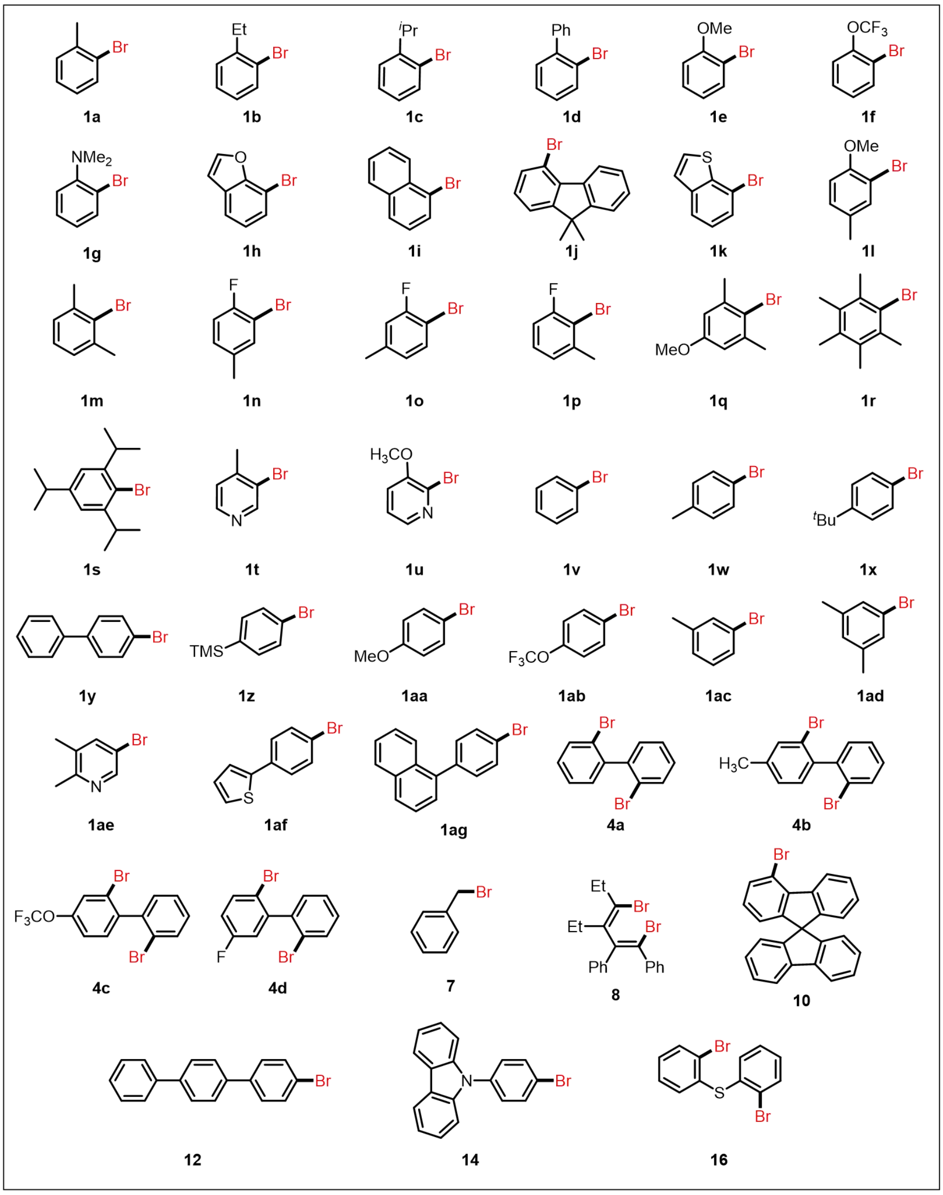

(IV) Synthesis and Analytical Data for Products

General Procedure A: In an argon-filled glove-box, a 25 mL oven-dried seal-tube equipped with a magnetic stir bar was charged with lithium powder (6.0 equiv., 3.0 mmol, 0.0210 g). The tube was removed from the glovebox, degassed and refilled with N_2. The tube was stirred under nitrogen atmosphere at 150 °C for 4 h. Then aryl bromide (1.0 equiv., 0.50 mmol), tris(dibenzylideneacetone)dipalladium (0.025 equiv., 0.0125 mmol, 0.0114 g), 2-dicyclohexylphosphino-2',6'-diisopropoxybiphenyl (0.10 equiv., 0.05 mmol, 0.0233 g), sodium *tert*-butoxide (2.0 equiv., 1.0 mmol, 0.0961 g), tetrabutylammonium bromide (1.0 equiv., 0.50 mmol, 0.1610 g) and dioxane (2.0 mL) were added into the

above tube, and the reaction mixture was stirred under nitrogen atmosphere at 150 °C for 24 h. The reaction mixture was allowed to cool to room temperature and quenched by water. A saturated solution of brine and ethyl acetate were added. The aqueous phase was extracted three times with ethyl acetate. The combined organic layer was washed with brine and filtered. The solvent was removed under reduced pressure. Finally, the residue was purified by flash chromatography on silica gel.

General Procedure B: In an argon-filled glove-box, a 25 mL oven-dried seal-tube equipped with a magnetic stir bar was charged with lithium powder (6.0 equiv., 3.0 mmol, 0.0210 g). The tube was removed from the glovebox, degassed and refilled with N_2. The tube was stirred under $^{15}N_2$ atmosphere at 150 °C for 4 h. Then aryl bromide (1.0 equiv., 0.50 mmol), tris(dibenzylideneacetone)dipalladium (0.025 equiv., 0.0125 mmol, 0.0114 g), 2-dicyclohexylphosphino-2',6'-diisopropoxybiphenyl (0.10 equiv., 0.05 mmol, 0.0233 g), sodium *tert*-butoxide (2.0 equiv., 1.0 mmol, 0.0961 g), tetrabutylammonium bromide (1.0 equiv., 0.50 mmol, 0.1610 g) and dioxane (2.0 mL) were added into the above tube, and the reaction mixture was stirred under argon atmosphere at 150 °C for 24 h. The reaction mixture was allowed to cool to room temperature and quenched by water. A saturated solution of brine and ethyl acetate were added. The aqueous phase was extracted three times with ethyl acetate. The combined organic layer was washed with brine and filtered. The solvent was removed under reduced pressure. Finally, the residue was purified by flash chromatography on silica gel.

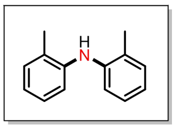

Compound 2a: di-o-tolylamine. **Procedure:** The title compound was synthesized via *General Procedure A* from 1-bromo-2-methylbenzene **1a** (85.0 mg, 0.50 mmol). The product was isolated in 87% yield (42.9 mg) via flash chromatography (petroleum ether). White solid. **Analytical data :** ^{1}H NMR (400 MHz, $CDCl_3$) δ 7.22 (d, *J* = 7.4 Hz, 2H), 7.14 (t, *J* = 7.7 Hz, 2H), 7.01 (d, *J* = 8.0, 2H), 6.93 (t, *J* = 7.4 Hz, 2H), 5.17 (s, 1H), 2.29 (s, 6H); ^{13}C NMR (100 MHz, $CDCl_3$) δ 141.94, 130.79, 127.49, 126.78, 121.33, 118.26, 17.77. IR (neat, cm^{-1}) 589.98, 711.04, 739.40, 850.20, 881.33, 925.90, 986.30, 1038.08, 1110.69, 1153.87, 1256.61, 1287.72, 1298.56, 1378.94, 1423.78, 1439.35, 1466.63, 1490.74, 1578.60, 2913.97, 3011.46, 3431.26. HRMS calcd. for $C_{14}H_{16}N$: 198.1277 [M+H$^+$]; found (ESI+): 198.1274. Mp: 46-48 **°C**. The spectroscopic data match a literature report.[1]

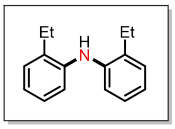

Compound 2b: bis(2-ethylphenyl)amine. **Procedure:** The title compound was synthesized via *General Procedure A* from 1-bromo-2-ethylbenzene **1b** (92.0 mg, 0.50 mmol). The product was isolated in 88% yield (49.5 mg) via flash chromatography (petroleum ether). Colorless oil. **Analytical data :** ^{1}H NMR (400 MHz, $CDCl_3$) δ 7.22 (d, *J* = 7.5 Hz, 2H), 7.16 – 7.07 (m, 2H), 7.01 (d, *J* = 8.1 Hz, 2H), 6.98 – 6.90 (m, 2H), 5.28 (s, 1H), 2.64 (q, *J* = 7.5 Hz, 4H), 1.28 (t, *J* = 7.5 Hz, 6H); ^{13}C NMR (100 MHz, $CDCl_3$) δ 141.62, 133.31, 128.81, 126.69, 121.50, 118.67, 24.33, 13.72. IR (neat, cm^{-1}) 573.03, 723.56, 744.94, 806.05, 927.91, 1236.83, 1291.48, 1324.90, 1450.42, 1492.64, 1503.23, 1585.60, 1599.46, 2851.50, 2922.00, 2958.84, 3415.38. HRMS calcd. for $C_{16}H_{20}N$: 226.1590 [M+H$^+$]; found (ESI+): 226.1585.

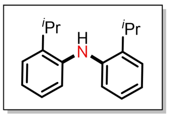

Compound 2c: bis(2-isopropylphenyl)amine. **Procedure:** The title compound was synthesized via *General Procedure A* from 1-bromo-2-isopropylbenzene **1c** (1.0 equiv., 0.50 mmol, 0.0995 g). The product was isolated in 82% yield (51.9 mg) via flash chromatography (petroleum ether). Colorless oil. **Analytical data :** ^{1}H NMR (400 MHz, $CDCl_3$) δ 7.31 (d, *J* = 7.6 Hz, 2H), 7.11 (t, *J* = 7.6, 2H), 6.99 (t, *J* = 8.8 Hz, 4H), 5.35 (s, 1H), 3.13 (hept, *J* = 6.8 Hz, 2H), 1.31 (d, *J* = 6.8 Hz, 12H); ^{13}C NMR (100 MHz, $CDCl_3$) δ 141.34, 138.13, 126.48, 125.75, 121.77, 119.35, 27.76, 22.75. IR (neat, cm^{-1}) 572.45, 644.12, 724.75, 744.77, 806.48, 865.57, 899.78, 928.46, 996.74, 1035.99, 1081.09, 1109.08, 1159.99, 1291.40, 1361.11, 1383.73, 1448.96, 1489.25, 1582.27, 1598.37, 2868.09, 2959.94, 3050.75, 3415.23. HRMS calcd. for $C_{18}H_{24}N$: 254.1903 [M+H$^+$]; found (ESI+): 254.1901. The spectroscopic data match a literature report.[1]

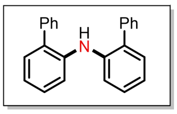

Compound 2d: di([1,1'-biphenyl]-2-yl)amine. **Procedure:** The title compound was synthesized via *General Procedure A* from 2-bromobiphenyl **1d** (1.0 equiv., 0.50 mmol, 0.1166 g). The product was isolated in 89% yield (71.4 mg) via flash chromatography (petroleum ether). Colorless oil. **Analytical data :** ^{1}H NMR (400 MHz, $CDCl_3$) δ 7.43 (d, *J* = 8.2 Hz, 2H), 7.34 – 7.14 (m, 14H), 6.98 (td, *J* = 7.4 Hz, 2H), 5.80 (s, 1H); ^{13}C NMR (100 MHz, $CDCl_3$) δ 140.15, 138.89, 132.05, 130.64, 129.00, 128.65, 128.14, 127.16, 120.78, 117.01. IR (neat, cm^{-1}) 551.64, 576.50, 614.73, 697.71, 721.54, 741.07, 761.89, 833.38, 914.34, 995.41, 1008.19, 1038.85,

1073.72, 1113.27, 1156.51, 1191.07, 1278.26, 1293.63, 1315.58, 1364.46, 1433.40, 1460.37, 1480.47, 1511.90, 1579.40, 1596.76, 2853.04, 2921.28, 3033.47, 3408.68. HRMS calcd. for $C_{24}H_{20}N$: 322.1590 [M+H$^+$]; found (ESI+): 322.1587. The spectroscopic data match a literature report.[1]

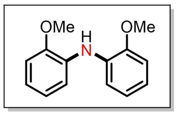

Compound 2e: bis(2-methoxyphenyl)amine. **Procedure:** The title compound was synthesized via *General Procedure A* from 2-bromoanisole **1e** (1.0 equiv., 0.50 mmol, 0.0935 g). The product was isolated in 72% yield (41.3 mg) via flash chromatography (petroleum ether/ethyl acetate = 100 : 1). Colorless oil. **Analytical data :** ^{1}H NMR (400 MHz, $CDCl_3$) δ 7.45 (d, *J* = 6.6 Hz, 2H), 7.03 – 6.86 (m, 6H), 6.57 (s, 1H), 3.94 (s, 6H); ^{13}C NMR (100 MHz, $CDCl_3$) δ 148.90, 132.38, 120.62, 120.04, 115.35, 110.49, 55.50. IR (neat, cm^{-1}) 570.40, 591.16, 630.27, 661.24, 686.18, 735.23, 772.47, 797.82, 821.80, 838.87, 888.02, 907.84, 943.45, 970.23, 1023.72, 1047.70, 1087.75, 1112.66, 1153.62, 1173.98, 1239.55, 1287.94, 1316.87, 1343.00, 1394.61, 1422.24, 1452.58, 1495.80, 1512.47, 1587.71, 1599.92, 2834.23, 2929.92, 3043.08, 3419.19. HRMS calcd. for $C_{14}H_{16}NO_2$: 230.1176 [M+H$^+$]; found (ESI+): 230.1173. The spectroscopic data match a literature report.[1]

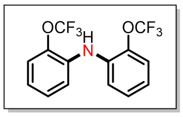

Compound 2f: bis(2-(trifluoromethoxy)phenyl)amine. **Procedure:** The title compound was synthesized via *General Procedure A* from 2-(trifluoromethoxy)bromobenzene **1f** (1.0 equiv., 0.50 mmol, 0.1205 g). The product was isolated in 51% yield (43.0 mg) via flash chromatography (petroleum ether/ethyl acetate = 100 : 1). Colorless oil. **Analytical data :** ^{1}H NMR (400 MHz, $CDCl_3$) δ 7.38 (d, *J* = 8.2 Hz, 2H), 7.30 (d, *J* = 7.5 Hz, 2H), 7.27 – 7.18 (m, 2H), 7.06 – 6.88 (m, 2H), 6.17 (s, 1H); ^{13}C NMR (100 MHz, $CDCl_3$) δ 139.20, 135.06, 127.48, 122.10, 121.69, 120.76 (q, *J* = 255 Hz), 118.34; ^{19}F NMR (377 MHz, $CDCl_3$) δ -58.08. IR (KBr, cm^{-1}) 418.5, 616.8, 720.1, 1108.8, 1378.4, 1464.4, 1633.3, 1659.4, 1738.7, 2850.9, 2921.9, 2956.4, 3191.6, 3360.2. HRMS calcd. for $C_{14}H_{10}F_6NO_2$: 338.0610 [M+H$^+$]; found (ESI+): 338.0606.

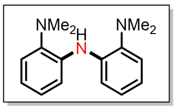

Compound 2g : N^1-(2-(dimethylamino)phenyl)-N^2,N^2-dimethylbenzene-1,2-diamine. **Procedure:** The title compound was synthesized via *General Procedure A* from 2-bromo-N,N-dimethylaniline **1g** (1.0 equiv., 0.50 mmol, 0.0995 g). The product was isolated in 75% yield (47.9 mg) via flash chromatography (petroleum ether/ethyl acetate = 40 : 1). R_f = 0.5. Brown oil. **Analytical data :** ^{1}H NMR (400 MHz, $CDCl_3$) δ 7.40 (d, *J* = 8.0, 2H), 7.16 (s, 1H), 7.11 (d, *J* = 7.8 Hz, 2H), 7.01 (t, *J* = 7.7 Hz, 2H), 6.87 (t, *J* = 7.6 Hz, 2H), 2.70 (s, 12H); ^{13}C NMR (100 MHz, $CDCl_3$) δ 143.30, 137.48, 123.59, 119.91, 119.36, 115.39, 43.90. IR (neat, cm^{-1}) 570.35, 593.71, 630.10, 661.60, 686.00, 709.66, 739.02, 774.23, 791.43, 799.04, 822.41, 840.68, 858.88, 874.87, 888.13, 943.54, 970.35, 1019.52, 1037.67, 1087.79, 1112.12, 1152.79, 1238.52, 1265.14, 1288.61, 1316.35, 1343.40, 1394.25, 1454.35, 1500.39, 1510.59, 1599.71, 1665.82, 2342.03, 2849.85, 2919.59, 3035.60, 3313.55. HRMS calcd. for $C_{16}H_{22}N_3$: 256.1808 [M+H$^+$]; found (ESI+): 256.1809. The spectroscopic data match a literature report.[2]

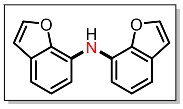

Compound 2h: di(benzofuran-7-yl)amine. **Procedure:** The title compound was synthesized via *General Procedure A* from 7-bromo-1-benzofuran **1h** (1.0 equiv., 0.50 mmol, 0.0985 g). The product was isolated in 31% yield (19.3 mg) via flash chromatography (petroleum ether/ethyl acetate = 100 : 1). Yellow oil. **Analytical data :** ^{1}H NMR (400 MHz, $CDCl_3$) δ 7.65 (t, *J* = 1.8 Hz, 2H), 7.27 (d, *J* = 6.5 Hz, 2H), 7.24 – 7.11 (m, 4H), 6.81 (t, *J* = 1.8 Hz, 2H), 6.51 (s, 1H); ^{13}C NMR (100 MHz, $CDCl_3$) δ 145.68, 144.53, 128.19, 128.06, 123.38, 113.66, 111.31, 107.27. IR (neat, cm^{-1}) 565.53, 606.49, 628.14, 724.78, 745.99, 781.06, 832.05, 844.75, 880.95, 1024.85, 1077.08, 1121.85, 1165.24, 1184.66, 1203.93, 1254.35, 1303.83, 1327.17, 1358.99, 1406.62, 1436.52, 1485.22, 1547.23, 1587.63, 1627.02, 2919.74, 3050.35, 3405.19. HRMS calcd. for $C_{16}H_{12}NO_2$: 250.0863 [M+H$^+$]; found (ESI+): 250.0860.

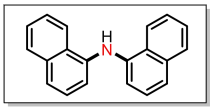

Compound 2i: di(naphthalen-1-yl)amine. **Procedure:** The title compound was synthesized via *General Procedure A* from 1-bromonaphthalene **1i** (1.0 equiv., 0.50 mmol, 0.1035 g). The product was isolated in 77% yield (51.8 mg) via flash chromatography (petroleum ether/ethyl acetate = 100 : 1). Yellow solid. **Analytical data :** ^{1}H NMR (400 MHz, $CDCl_3$) δ 8.20 – 8.02 (m, 2H), 8.00 – 7.81 (m, 2H), 7.67 – 7.43 (m, 6H), 7.35 (t, *J* = 7.8 Hz, 2H), 7.04 (d, *J* = 7.4 Hz, 2H), 6.34 (s, 1H); ^{13}C NMR (100 MHz, $CDCl_3$) δ 140.27, 134.65, 128.61, 126.90, 126.18, 126.10, 125.65, 122.37, 121.76, 115.41. IR (neat, cm^{-1}) 568.35, 591.51, 621.01, 691.78, 746.31, 771.65, 790.55, 839.72, 956.30, 974.35, 1017.62, 1074.36, 1096.06, 1115.95, 1171.12, 1275.14, 1315.82, 1393.24, 1469.38, 1502.44, 1583.54, 3059.68, 3394.17. HRMS calcd. for $C_{20}H_{16}N$: 270.1277 [M+H$^+$]; found (ESI+): 270.1275. Mp: 108-110 **°C**. The spectroscopic data match a literature report.[3]

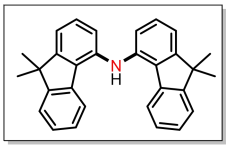

Compound 2j: bis(9,9-dimethyl-9H-fluoren-4-yl)amine. **Procedure:** The title compound was synthesized via *General Procedure A* from 4-bromo-9,9-dimethyl fluorene **1j** (1.0 equiv., 0.50 mmol, 0.1366 g). The product was isolated in 79% yield (79.2 mg) via flash chromatography (petroleum ether/ethyl acetate = 60 : 1). R_f = 0.5. White solid. **Analytical data :** ^{1}H NMR (400 MHz, $CDCl_3$) δ 7.86 (d, *J* = 7.3 Hz, 2H), 7.59 – 7.44 (m, 2H), 7.35 – 7.20 (m, 6H), 7.16 (d, *J* = 7.3 Hz, 2H), 7.03 (d, *J* = 7.6 Hz, 2H), 6.37 (s, 1H), 1.57 (s, 12H); ^{13}C NMR (100 MHz, $CDCl_3$) δ 155.71, 153.67, 139.27, 138.55, 129.15, 128.15, 127.08, 126.46, 122.47, 122.35, 118.20, 116.36, 46.65, 27.47. IR (KBr, cm^{-1}) 418.3, 447.5, 533.9, 567.6, 590.8, 608.5, 649.4, 681.3, 704.2, 730.7, 759.1, 772.4, 797.9, 939.5, 997.9, 1031.4, 1065.5, 1115.7, 1143.9, 1159.4, 1207.4, 1265.0, 1307.2, 1350.1, 1359.5, 1389.2, 1455.5, 1485.6, 1580.9, 1603.0, 1910.4, 2860.7, 2922.1, 2959.9, 3014.7, 3049.2, 3383.3, 3465.2. HRMS calcd. for $C_{30}H_{28}N$: 402.2216 [M+H$^+$]; found (ESI+): 402.2211. Mp: 58-60 **°C**.

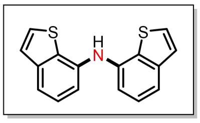

Compound 2k: bis(benzo[b]thiophen-7-yl)amine. **Procedure:** The title compound was synthesized via *General Procedure A* from 7-bromo-1-benzothiophene **1k** (1.0 equiv., 0.50 mmol, 0.1065 g). The product was isolated in 58% yield (40.8 mg) via flash chromatography (petroleum ether/ethyl acetate = 100 : 1). Yellow solid. **Analytical data :** ^{1}H NMR (400 MHz, $CDCl_3$) δ 7.53 (d, *J* = 7.8 Hz, 2H), 7.38 (q, *J* = 5.5 Hz, 4H), 7.29 (t, *J* = 7.8 Hz, 2H), 7.05 (d, *J* = 7.6 Hz, 2H), 5.72 (s, 1H); ^{13}C NMR (100 MHz, $CDCl_3$) δ 141.27, 137.39, 131.41, 125.66, 125.23, 124.81, 117.90, 113.79. IR (neat, cm^{-1}) 593.57, 639.92, 687.82, 696.25, 731.84, 774.70, 783.57, 803.40, 840.14, 892.38, 956.18, 982.05, 1033.19, 1052.32, 1093.97, 1104.81, 1172.20, 1212.33, 1289.61, 1317.44, 1334.97, 1395.87, 1457.46, 1513.67, 1561.46, 1591.37, 2359.56, 3056.40, 3097.19, 3378.66. HRMS calcd. for $C_{16}H_{12}NS_2$: 282.0406 [M+H^{+}]; found (ESI+): 282.0403. Mp: 93-95 °C.

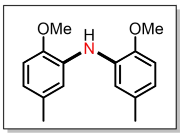

Compound 2l: bis(2-methoxy-5-methylphenyl)amine. **Procedure:** The title compound was synthesized via *General Procedure A* from 3-bromo-4-methoxytoluene **1l** (1.0 equiv., 0.50 mmol, 0.1005 g). The product was isolated in 59% yield (38.0 mg) via flash chromatography (petroleum ether/ethyl acetate = 100 : 1). White solid. **Analytical data :** ^{1}H NMR (400 MHz, $CDCl_3$) δ 7.19 (d, *J* = 2.0 Hz, 2H), 6.79 (d, *J* = 8.1 Hz, 2H), 6.71 – 6.62 (m, 2H), 6.38 (s, 1H), 3.86 (s, 6H), 2.28 (s, 6H); ^{13}C NMR (100 MHz, $CDCl_3$) δ 147.05, 132.28, 130.07, 120.31, 116.48, 110.58, 55.79, 21.00. IR (KBr, cm^{-1}) 418.6, 447.3, 518.8, 585.0, 615.6, 698.9, 725.7, 794.0, 850.4, 1033.3, 1134.0, 1163.0, 1182.3, 1228.2, 1247.6, 1287.7, 1337.2, 1412.0, 1456.3, 1507.5, 1536.9, 1592.0, 2833.6, 2850.4, 2919.9, 2999.1, 3420.0. HRMS calcd. for $C_{16}H_{20}NO_2$: 258.1489 [M+H^{+}]; found (ESI+): 258.1486. Mp: 71-72 °C.

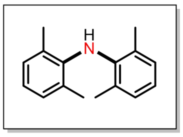

Compound 2m: bis(2,6-dimethylphenyl)amine. **Procedure:** The title compound was synthesized via *General Procedure A* from 2-bromo-m-xylene **1m** (1.0 equiv., 0.50 mmol, 0.0925 g). The product was isolated in 76% yield (42.8 mg) via flash chromatography (petroleum ether). White solid. **Analytical data :** ^{1}H NMR (400 MHz, $CDCl_3$) δ 7.00 (d, *J* = 7.4 Hz, 4H), 6.86 (t, *J* = 7.5 Hz, 2H), 4.82 (s, 1H), 2.04 (s, 12H); ^{13}C NMR (100 MHz, $CDCl_3$) δ 141.73, 129.54, 128.68, 121.69, 19.09. IR (neat, cm^{-1}) 560.58, 577.73, 679.51, 699.67, 726.81, 753.76, 766.41, 876.83, 912.74, 932.30, 950.36, 987.96, 1027.22, 1097.22, 1157.40, 1204.06, 1231.33, 1272.34, 1295.39, 1375.80, 1435.89, 1468.13, 1485.20, 1588.39, 2917.49, 2946.92, 3036.35, 3408.94. HRMS calcd. for $C_{16}H_{20}N$: 226.1590 [M+H^{+}]; found (ESI+): 226.1589. Mp: 95-97 °C. The spectroscopic data match a literature report.[1]

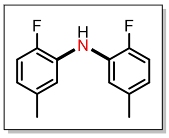

Compound 2n: bis(2-fluoro-5-methylphenyl)amine. **Procedure:** The title compound was synthesized via *General Procedure A* from 3-bromo-4-fluorotoluene **1n** (1.0 equiv., 0.50 mmol, 0.0945 g). The product was isolated in 60% yield (35.0 mg) via flash chromatography (petroleum ether). White solid. **Analytical data :** ^{1}H NMR (400 MHz, $CDCl_3$) δ 7.04 (d, J = 8.2 Hz, 2H), 6.94 (dd, J = 11.0, 8.3 Hz, 2H), 6.67 – 6.64 (m, 2H), 5.70 (s, 1H), 2.25 (s, 6H); ^{13}C NMR (100

MHz, $CDCl_3$) δ 151.88 (d, *J* = 238 Hz), 133.90 (d, *J* = 3.0 Hz), 130.22 (d, *J* = 12 Hz), 121.90 (d, *J* = 6.0 Hz), 118.82, 115.21 (d, *J* = 19 Hz), 21.05; ^{19}F NMR (377 MHz, $CDCl_3$) δ -135.94. IR (KBr, cm^{-1}) 441.7, 462.2, 479.6, 578.8, 620.9, 703.8, 720.2, 738.1, 800.3, 861.3, 924.8, 934.3, 965.2, 1011.2, 1039.6, 1107.6, 1141.1, 1157.4, 1210.3, 1257.0, 1298.5, 1329.6, 1381.6, 1417.2, 1462.3, 1506.1, 1532.9, 1613.3, 1849.3, 2865.8, 2926.2, 3051.2, 3436.7. HRMS calcd. for $C_{14}H_{14}F_2N$: 234.1089 [$M+H^+$]; found (ESI+): 234.1087. Mp: 51-52 **°C**.

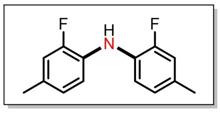

Compound 2o: bis(2-fluoro-4-methylphenyl)amine. **Procedure:** The title compound was synthesized via *General Procedure A* from 1-bromo-2-fluoro-4-methylbenzene **1o** (1.0 equiv., 0.50 mmol, 0.0940 g). The product was isolated in 68% yield (39.6 mg) via flash chromatography (petroleum ether). White solid. **Analytical data :** ^1H NMR (400 MHz, $CDCl_3$) δ 7.11 (t, *J* = 8.5 Hz, 2H), 6.93 (dd, *J* = 11.9, 1.9 Hz, 2H), 6.85 (d, *J* = 8.2 Hz, 2H), 5.63 (s, 1H), 2.31 (s, 6H); ^{13}C NMR (100 MHz, $CDCl_3$) δ 153.49 (d, *J* = 240 Hz), 131.57 (d, *J* = 7.0 Hz), 128.35 (d, *J* = 11 Hz), 124.64 (d, *J* = 4.0 Hz), 118.27, 116.21 (d, *J* = 19 Hz), 20.62; ^{19}F NMR (377 MHz, $CDCl_3$) δ -131.67. IR (KBr, cm^{-1}) 616.8, 890.0, 1108.2, 1352.2, 1383.9, 1462.7, 1667.1, 2871.7, 2933.4, 2957.9, 3423.5. HRMS calcd. for $C_{14}H_{14}F_2N$: 234.1089 [$M+H^+$]; found (ESI+): 234.1085. Mp: 44-45 **°C**.

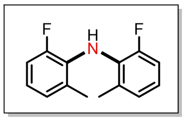

Compound 2p: bis(2-fluoro-6-methylphenyl)amine. **Procedure:** The title compound was synthesized via *General Procedure A* from 2-bromo-3-fluorotoluene **1p** (1.0 equiv., 0.50 mmol, 0.0945 g) (85.0 mg, 0.50 mmol). The product was isolated in 69% yield (40.3 mg) via flash chromatography (petroleum ether). White solid. **Analytical data :** ^1H NMR (400 MHz, $CDCl_3$) δ 6.93 – 6.86 (m, 6H), 4.97 (s, 1H), 2.15 (s, 6H); ^{13}C NMR (100 MHz, $CDCl_3$) δ 155.74 (d, *J* = 241 Hz), 132.01, 125.92 (d, *J* = 3.0 Hz), 122.27 (d, *J* = 8.0 Hz), 113.23 (d, *J* = 20 Hz), 18.13; ^{19}F NMR (377 MHz, $CDCl_3$) δ -127.64. IR (KBr, cm^{-1}) 459.5, 502.7, 544.5, 705.4, 718.1, 769.0, 882.5, 938.8, 1024.7, 1084.7, 1156.8, 1206.1, 1231.5, 1263.8, 1311.5, 1379.4, 1447.7, 1485.5, 1584.8, 1614.9, 2853.8, 2923.2, 3027.6, 3412.5. HRMS calcd. For $C_{14}H_{14}F_2N$: 234.1089 [$M+H^+$]; found (ESI+): 234.1090. Mp: 63-65 **°C**.

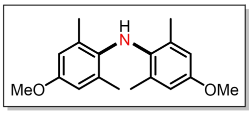

Compound 2q: bis(4-methoxy-2,6-dimethylphenyl)amine. **Procedure:** The title compound was synthesized via *General Procedure A* from 4-bromo-3,5-dimethylanisole **1q** (1.0 equiv., 0.50 mmol, 0.1075 g). The product was isolated in 74% yield (52.8 mg) via flash chromatography (petroleum ether/ethyl acetate = 100 : 1). White solid. **Analytical data :** ^1H NMR (400 MHz, $CDCl_3$) δ 6.55 (s, 4H), 4.43 (s, 1H), 3.75 (s, 6H), 2.00 (s, 12H); ^{13}C NMR (100 MHz, $CDCl_3$) δ 154.11, 135.87, 130.90, 113.89, 55.38, 19.35. IR (KBr, cm^{-1}) 482.4, 571.6, 609.6, 639.8, 717.8, 827.8, 851.9, 871.1, 944.9, 957.1, 994.3, 1064.1, 1154.0, 1193.0, 1212.2, 1243.5, 1282.8, 1324.4, 1374.0, 1437.9, 1463.3, 1483.9, 1601.9, 2731.5, 2832.8, 2911.2, 2957.1, 2994.7, 3400.6. HRMS calcd. for $C_{18}H_{24}NO_2$: 286.1802 [$M+H^+$]; found (ESI+): 286.1800. Mp: 100-102 **°C**.

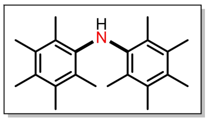

Compound 2r: bis(2,3,4,5,6-pentamethylphenyl)amine. **Procedure:** The title compound was synthesized via *General Procedure A* from bromopentamethylbenzene **1r** (1.0 equiv., 0.50 mmol, 0.1136 g). The product was isolated in 79% yield (61.1 mg) via flash chromatography (petroleum ether). White solid. **Analytical data :** ^{1}H NMR (400 MHz, $CDCl_3$) δ 4.77 (s, 1H), 2.23 (s, 6H), 2.21 (s, 12H), 1.96 (s, 12H); ^{13}C NMR (100 MHz, $CDCl_3$) δ 140.60, 132.69, 128.11, 125.08, 16.91, 16.58, 15.48. IR (KBr, cm^{-1}) 571.6, 617.0, 701.9, 730.4, 776.3, 816.8, 835.7, 1013.2, 1061.9, 1125.0, 1259.5, 1317.0, 1373.0, 1412.2, 1459.3, 1568.5, 2727.6, 2918.5, 3431.4. HRMS calcd. for $C_{22}H_{32}N$: 310.2529 [M+H$^+$]; found (ESI+): 310.2527. Mp: 200-202 °C.

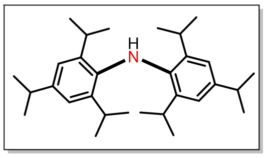

Compound 2s: bis(2,4,6-triisopropylphenyl)amine. **Procedure:** The title compound was synthesized via *General Procedure A* from 1-bromo-2,4,6-triisopropylbenzene **1s** (1.0 equiv., 0.50 mmol, 0.1416 g). The product was isolated in 72% yield (75.8 mg) via flash chromatography (petroleum ether). White solid. **Analytical data :** ^{1}H NMR (400 MHz, $CDCl_3$) δ 6.92 (s, 4H), 4.68 (s, 1H), 3.10 (hept, *J* = 6.9 Hz, 4H), 2.86 (hept, *J* = 6.9 Hz, 2H), 1.25 (d, *J* = 6.9 Hz, 12H), 1.08 (d, *J* = 6.9 Hz, 24H); ^{13}C NMR (100 MHz, $CDCl_3$) δ 142.74, 140.70, 138.59, 121.57, 33.90, 27.77, 24.25, 23.64. IR (KBr, cm^{-1}) 418.6, 644.8, 743.4, 877.2, 940.7, 1054.7, 1070.3, 1120.9, 1167.7, 1182.6, 1264.3, 1319.9, 1341.1, 1362.1, 1381.7, 1468.0 1633.6, 1659.8, 2854.0, 2924.6, 2957.7, 3360.3, 3439.3. HRMS calcd. for $C_{30}H_{48}N$: 422.3781 [M+H$^+$]; found (ESI+): 422.3776. Mp: 94-95 °C.

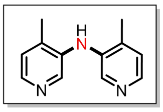

Compound 2t: bis(4-methylpyridin-3-yl)amine. **Procedure:** The title compound was synthesized via *General Procedure A* from 3-bromo-4-methylpyridine **1t** (1.0 equiv., 0.50 mmol, 0.0860 g). The product was isolated in 71% yield (35.3 mg) via flash chromatography (dichloromethane/methanol = 20 : 1). R_f = 0.6. Yellow solid. **Analytical data :** ^{1}H NMR (400 MHz, $CDCl_3$) δ 8.07 (d, *J* = 4.8 Hz, 2H), 7.99 (s, 2H), 7.05 (d, *J* = 4.8 Hz, 2H), 5.68 (s, 1H), 2.18 (s, 6H); ^{13}C NMR (100 MHz, $CDCl_3$) δ 143.57, 140.70, 138.14, 137.16, 125.56, 17.26. IR (KBr, cm^{-1}) 415.3 423.8, 438.3, 458.3 503.9, 549.4, 606.0, 630.5 698.6, 731.5, 770.8, 798.1 829.6, 865.1, 900.9, 924.1, 995.1, 1037.1, 1071.5, 1113.3, 1175.8, 1202.6, 1215.0, 1245.0, 1289.7, 1321.8, 1375.4, 1400.0, 1426.2, 1442.8, 1499.3, 1525.0, 1557.5, 1577.7, 1598.0, 1697.8, 2297.8, 2985.4, 3054.8, 3226.9. HRMS calcd. for $C_{12}H_{14}N_3$: 200.1182 [M+H$^+$]; found (ESI+): 200.1183. Mp: 46-48 °C.

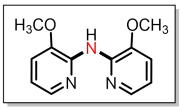

Compound 2u: bis(3-methoxypyridin-2-yl)amine. **Procedure:** The title compound was synthesized via *General Procedure A* from 2-bromo-3-methoxypyridine **1u** (1.0 equiv., 0.50 mmol, 0.0935 g). The product was isolated in 65% yield (37.6 mg) via flash chromatography (dichloromethane/methanol = 20 : 1). R_f = 0.5. Yellow solid. **Analytical data :** ^{1}H NMR (400 MHz, $CDCl_3$) δ 8.03 (d, *J* = 5.1 Hz, 2H), 7.97 (s, 1H), 7.05 (dd, *J* = 7.9, 1.5 Hz, 2H), 6.82

(dd, *J* = 7.9, 5.0 Hz, 2H), 3.92 (s, 6H); ^{13}C NMR (100 MHz, $CDCl_3$) δ 144.55, 143.67, 139.35, 115.81, 115.58, 55.54. IR (neat, cm^{-1}) 568.79, 629.68, 660.77, 686.15, 701.55, 750.53, 775.43, 790.63, 821.29, 839.33, 943.67, 969.88, 1015.56, 1070.88, 1109.89, 1152.95, 1175.83, 1194.54, 1234.95, 1288.11, 1316.20, 1342.98, 1394.44, 1429.76, 1456.05, 1509.12, 1602.83, 1644.97, 2849.82, 2921.23, 3400.81. HRMS calcd. for $C_{12}H_{14}N_3O_2$: 232.1081 [M+H$^+$]; found (ESI+): 232.1077. Mp: 55-56 °C.

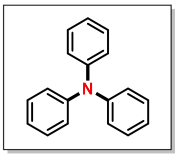

Compound 3a: triphenylamine. **Procedure:** The title compound was synthesized via *General Procedure A* from bromobenzene **1v** (78.0 mg, 0.50 mmol). The product was isolated in 86% yield (35.1 mg) via flash chromatography (petroleum ether). White solid. **Analytical data :** ^{1}H NMR (400 MHz, $CDCl_3$) δ 7.24 (t, *J* = 7.7 Hz, 6H), 7.09 (d, *J* = 8.0 Hz, 6H), 7.04 – 6.93 (m, 3H); ^{13}C NMR (100 MHz, $CDCl_3$) δ 147.84, 129.18, 124.15, 122.64. IR (neat, cm^{-1}) 571.86, 613.83, 620.93, 691.03, 746.63, 1026.44, 1074.03, 1172.20, 1274.07, 1313.77, 1327.36, 1396.08, 1489.29, 1582.50. HRMS calcd. for $C_{18}H_{16}N$: 246.1277 [M+H$^+$]; found (ESI+): 246.1273. Mp: 124-125 °C. The spectroscopic data match a literature report.[1]

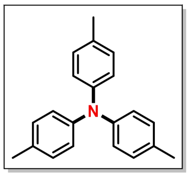

Compound 3b: tri-*p*-tolylamine. **Procedure:** The title compound was synthesized via *General Procedure A* from 4-bromotoluene **1w** (1.0 equiv., 0.50 mmol, 0.0855 g). The product was isolated in 76% yield (36.4 mg) via flash chromatography (petroleum ether). White solid. **Analytical data :** ^{1}H NMR (400 MHz, $CDCl_3$) δ 7.05 (d, *J* = 8.1 Hz, 6H), 6.98 (d, *J* = 8.1 Hz, 6H), 2.32 (s, 9H); ^{13}C NMR (100 MHz, $CDCl_3$) δ 145.69, 131.73, 129.70, 123.84, 20.73. IR (neat, cm^{-1}) 562.44, 715.51, 780.07, 809.63, 917.54, 1017.84, 1105.40, 1182.92, 1271.18, 1290.46, 1317.14, 1503.18, 1579.08, 1607.01, 2917.63, 3023.86. HRMS calcd. For $C_{21}H_{22}N$: 288.1747 [M+H$^+$]; found (ESI+): 288.1746. Mp: 113-114 °C. The spectroscopic data match a literature report.[4]

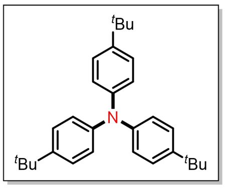

Compound 3c: tris(4-(tert-butyl)phenyl)amine. **Procedure:** The title compound was synthesized via *General Procedure A* from 1-bromo-4-tert-butylbenzene **1x** (1.0 equiv., 0.50 mmol, 0.1066 g). The product was isolated in 64% yield (44.1 mg) via flash chromatography (petroleum ether). White solid. **Analytical data :** ^{1}H NMR (400 MHz, $CDCl_3$) δ 7.24 (d, *J* = 8.1 Hz, 6H), 7.01 (d, *J* = 8.2 Hz, 6H), 1.31 (s, 27H); ^{13}C NMR (100 MHz, $CDCl_3$) δ 145.36, 144.99, 125.85, 123.36, 34.20, 31.44. IR (neat, cm^{-1}) 551.64, 576.45, 701.95, 731.22, 739.54, 759.51, 827.84, 1018.22, 1117.04, 1191.69, 1267.46, 1277.45, 1325.48, 1363.81, 1392.53, 1508.28, 1601.46, 2860.91, 2957.67. HRMS calcd. for $C_{30}H_{40}N$: 414.3155 [M+H$^+$]; found (ESI+): 414.3157. Mp: 281-283 °C. The spectroscopic data match a literature report.[1]

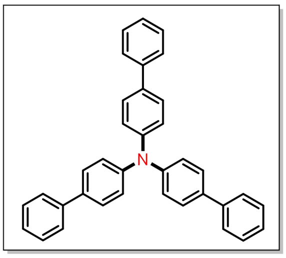

Compound 3d: tri([1,1'-biphenyl]-4-yl)amine. **Procedure:** The title compound was synthesized via *General Procedure A* from 4-bromo-1,1'-biphenyl **1y** (1.0 equiv., 0.50 mmol, 0.1160 g). The product was isolated in 82% yield (64.7 mg) via flash chromatography (petroleum ether/ethyl acetate = 100 : 1). White solid. **Analytical data :** ^{1}H NMR (400 MHz, $CDCl_3$) δ 7.60 (d, J = 7.7 Hz, 6H), 7.53 (d, J = 8.2 Hz, 6H), 7.44 (t, J = 7.5 Hz, 6H), 7.32 (t, J = 7.4 Hz, 3H), 7.24 (d, J = 7.5 Hz, 6H); ^{13}C NMR (100 MHz, $CDCl_3$) δ 146.83, 140.59, 135.63, 128.76, 127.90, 126.90, 126.70, 124.42. IR (neat, cm^{-1}) 551.64, 576.65, 615.93, 693.39, 720.29, 740.92, 761.68, 833.94, 908.98, 1006.61, 1038.83, 1074.37, 1113.33, 1154.83, 1187.67, 1263.70, 1278.27, 1292.76, 1315.88, 1364.55, 1447.21, 1481.68, 1513.15, 1596.32, 1660.58, 2850.35, 2919.66, 3032.42. HRMS calcd. for $C_{36}H_{28}N$: 474.2216 [M+H$^+$]; found (ESI+): 474.2219. Mp: 256-258 °**C**. The spectroscopic data match a literature report.[4]

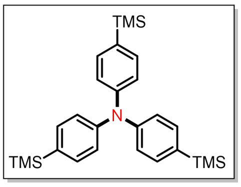

Compound 3e: tris(4-(trimethylsilyl)phenyl)amine. **Procedure:** The title compound was synthesized via *General Procedure A* from 1-bromo-4-(trimethylsilyl)benzene **1z** (1.0 equiv., 0.50 mmol, 0.1146 g). The product was isolated in 60% yield (46.1 mg) via flash chromatography (petroleum ether). White solid. **Analytical data :** ^{1}H NMR (400 MHz, $CDCl_3$) δ 7.40 (d, J = 8.5 Hz, 6H), 7.10 (d, J = 8.3 Hz, 6H), 0.28 (s, 27H); ^{13}C NMR (100 MHz, $CDCl_3$) δ 147.95, 134.26, 129.24, 123.40, -1.00. IR (KBr, cm^{-1}) 417.5, 444.5, 522.5, 532.6, 621.2, 635.9, 648.1, 687.2, 722.7, 756.9, 818.5, 839.5, 919.2, 1017.8, 1113.8, 1188.8, 1247.8, 1262.8, 1283.0, 1326.7, 1407.5, 1500.2, 1563.6, 1586.2, 1909.2, 2892.5, 2952.2, 3019.3, 3072.3. HRMS calcd. for $C_{27}H_{40}NSi_3$: 462.2463 [M+H$^+$]; found (ESI+): 462.2458. Mp: 241-242 °**C**.

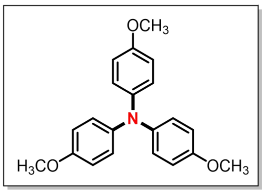

Compound 3f: tris(4-methoxyphenyl)amine. **Procedure:** The title compound was synthesized via *General Procedure A* from 4-bromoanisole **1aa** (1.0 equiv., 0.50 mmol, 0.0935 g). The product was isolated in 65% yield (36.3 mg) via flash chromatography (petroleum ether/ethyl acetate = 100 : 1). White solid. **Analytical data :** ^{1}H NMR (400 MHz, $CDCl_3$) δ 7.06 – 6.89 (m, 6H), 6.86 – 6.68 (m, 6H), 3.77 (s, 9H); ^{13}C NMR (100 MHz, $CDCl_3$) δ 154.89, 142.01, 124.79, 114.49, 55.49. IR (neat, cm^{-1}) 570.42, 630.61, 661.36, 686.36, 715.77, 777.62, 801.19, 822.89, 911.49, 943.80, 970.66, 1032.75, 1105.62, 1177.44, 1234.35, 1264.14, 1316.08, 1342.95, 1439.95, 1461.94, 1498.52, 1601.11, 2832.77, 2930.32. HRMS calcd. for $C_{21}H_{22}NO_3$: 336.1594 [M+H$^+$]; found (ESI+): 336.1591. Mp: 95-97 °**C**. The spectroscopic data match a literature report.[5]

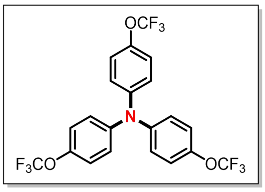

Compound 3g: tris(4-(trifluoromethoxy)phenyl)amine. **Procedure:** The title compound was synthesized via *General Procedure A* from 1-bromo-4-(trifluoromethoxy)benzene **1ab** (1.0 equiv., 0.50 mmol, 0.1205 g). The product was isolated in 48% yield (39.7 mg) via flash chromatography (petroleum ether/ethyl acetate = 100 : 1). Colorless oil. **Analytical data :** ^{1}H NMR (400 MHz, $CDCl_3$) δ 7.15 (d, *J* = 8.8 Hz, 6H), 7.12 – 7.06 (m, 6H); ^{13}C NMR (100 MHz, $CDCl_3$) δ 145.79, 144.74, 125.00, 123.22 (q, *J* = 256 Hz), 122.33; ^{19}F NMR (377 MHz, $CDCl_3$) δ -58.12. IR (KBr, cm^{-1}) 418.3, 529.5, 549.5, 629.9, 671.7, 722.6, 810.9, 845.0, 920.5, 1015.3, 1107.3, 1165.3, 1204.3, 1255.7, 1505.0, 1602.3, 2850.3, 2922.0, 3052.4, 3360.1. HRMS calcd. for $C_{21}H_{13}F_9NO_3$: 497.0673 $[M+H]^+$; found (ESI+): 497.0663.

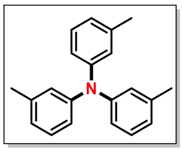

Compound 3h: tri-*m*-tolylamine. **Procedure:** The title compound was synthesized via *General Procedure A* from 1-bromo-3-methylbenzene **1ac** (1.0 equiv., 0.50 mmol, 0.0850 g). The product was isolated in 74% yield (35.4 mg) via flash chromatography (petroleum ether). White solid. **Analytical data :** ^{1}H NMR (400 MHz, $CDCl_3$) δ 7.15 (t, *J* = 7.7 Hz, 3H), 6.97 – 6.87 (m, 6H), 6.85 (d, *J* = 7.5 Hz, 3H), 2.29 (s, 9H); ^{13}C NMR (100 MHz, $CDCl_3$) δ 147.97, 138.92, 128.89, 124.80, 123.35, 121.38, 21.40. IR (neat, cm^{-1}) 633.23, 690.93, 700.24, 759.05, 772.74, 873.58, 964.33, 997.75, 1031.68, 1089.94, 1163.39, 1181.54, 1277.77, 1297.77, 1309.68, 1320.02, 1378.82, 1446.10, 1482.07, 1578.53, 1596.22, 2918.74, 3030.97. HRMS calcd. for $C_{21}H_{22}N$: 288.1747 $[M+H^+]$; found (ESI+): 288.1743. Mp: 58-59 °C. The spectroscopic data match a literature report.[1]

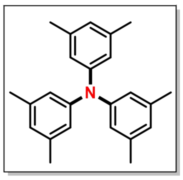

Compound 3i: tris(3,5-dimethylphenyl)amine. **Procedure:** The title compound was synthesized via *General Procedure A* from 3,5-dimethylphenyl bromide **1ad** (1.0 equiv., 0.50 mmol, 0.0925 g). The product was isolated in 58% yield (31.8 mg) via flash chromatography (petroleum ether). White solid. **Analytical data :** ^{1}H NMR (400 MHz, $CDCl_3$) δ 6.72 (d, *J* = 1.6 Hz, 6H), 6.68 (s, 3H), 2.25 (s, 18H); ^{13}C NMR (100 MHz, $CDCl_3$) δ 148.10, 138.59, 124.23, 122.09, 21.29. IR (neat, cm^{-1}) 568.85, 578.37, 677.53, 691.17, 735.40, 836.47, 876.82, 949.17, 1037.15, 1066.31, 1162.52, 1192.40, 1326.55, 1378.74, 1454.04, 1587.42, 2859.20, 2914.78, 3015.24. HRMS calcd. for $C_{24}H_{28}N$: 330.2216 $[M+H^+]$; found (ESI+): 330.2215. Mp: 198-200 °C. The spectroscopic data match a literature report.[6]

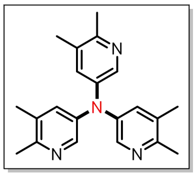

Compound 3j: tris(5,6-dimethylpyridin-3-yl)amine. **Procedure:** The title compound was synthesized via *General Procedure A* from 5-bromo-2,3-dimethylpyridine **1ae** (1.0 equiv., 0.50 mmol, 0.0925 g). The product was isolated in 57% yield (31.7 mg) via flash

chromatography (petroleum ether/ethyl acetate = 20 : 1). R_f = 0.4. Yellow solid. **Analytical data :** ^{1}H NMR (400 MHz, $CDCl_3$) 8.05 (d, *J* = 2.6 Hz, 3H), 7.09 (d, *J* = 2.6 Hz, 3H), 2.46 (s, 9H), 2.19 (s, 9H); ^{13}C NMR (100 MHz, $CDCl_3$) δ 151.95, 141.84, 141.14, 132.19, 131.79, 21.93, 19.22. IR (KBr, cm^{-1}) 418.6, 489.2, 549.1, 618.3, 700.9, 723.2, 825.4, 885.5, 972.8, 1023.5, 1075.8, 1121.8, 1146.0, 1180.3, 1233.9, 1261.5, 1320.0, 432.3, 1474.4, 1590.4, 1646.1, 2853.5, 2919.4, 3396.8. HRMS calcd. for $C_{21}H_{25}N_4$: 333.2074 [$M+H^+$]; found (ESI+): 333.2071. Mp: 124-126 °C.

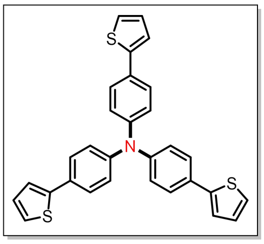

Compound 3k: tris(4-(thiophen-2-yl)phenyl)amine. **Procedure:** The title compound was synthesized via *General Procedure A* from 2-(4-bromophenyl)thiophene **1af** (1.0 equiv., 0.50 mmol, 0.1190 g). The product was isolated in 72% yield (58.9 mg) via flash chromatography (petroleum ether/ethyl acetate = 100 : 1). Yellow solid. **Analytical data :** ^{1}H NMR (400 MHz, $CDCl_3$) δ 7.54 – 7.48 (m, 6H), 7.27 – 7.23 (m, 6H), 7.16 – 7.10 (m, 6H), 7.06 (dd, *J* = 5.1, 3.6 Hz, 3H); ^{13}C NMR (100 MHz, $CDCl_3$) δ 146.49, 144.07, 129.28, 128.01, 126.87, 124.37, 124.22, 122.45. IR (neat, cm^{-1}) 581.41, 689.15, 728.65, 813.80, 834.95, 848.25, 958.08, 1049.94, 1078.81, 1111.13, 1182.13, 1208.15, 1271.02, 1289.64, 1319.15, 1430.44, 1496.09, 1531.95, 1596.78. HRMS calcd. for $C_{30}H_{22}NS_3$: 492.0909 [$M+H^+$]; found (ESI+): 492.0909. Mp: 180-182 °C. The spectroscopic data match a literature report.[7]

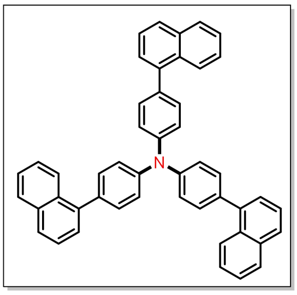

Compound 3l: tris(4-(naphthalen-1-yl)phenyl)amine. **Procedure:** The title compound was synthesized via *General Procedure A* from 1-(4-bromophenyl)naphthalene **1ag** (1.0 equiv., 0.50 mmol, 0.1410 g). The product was isolated in 81% yield (84.1 mg) via flash chromatography (petroleum ether/ethyl acetate = 100 : 1). White solid. **Analytical data :** ^{1}H NMR (400 MHz, $CDCl_3$) δ 8.16 – 8.03 (m, 3H), 8.00 – 7.90 (m, 3H), 7.87 (d, *J* = 8.1 Hz, 3H), 7.61 – 7.46 (m, 18H), 7.45 – 7.35 (m, 6H); ^{13}C NMR (100 MHz, $CDCl_3$) δ 146.86, 139.88, 135.26, 133.90, 131.67, 131.04, 128.32, 127.46, 126.93, 126.08, 125.98, 125.76, 125.45, 124.01. IR (neat, cm^{-1}) 569.40, 622.42, 650.64, 694.81, 733.40, 773.72, 791.55, 798.30, 839.27, 862.97, 963.00, 1019.29, 1059.43, 1115.45, 1178.67, 1245.24, 1266.59, 1286.53, 1315.78, 1394.23, 1458.35, 1499.31, 1600.32, 1736.12, 2922.90, 3041.42. HRMS calcd. for $C_{48}H_{34}N$: 624.2686 [$M+H^+$]; found (ESI+): 624.2668. Mp: 109-111 °C. The spectroscopic data match a literature report.[8]

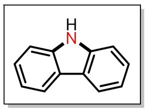

Compound 5a: 9H-carbazole. **Procedure:** The title compound was synthesized via *General Procedure A* from 2,2'-dibromo-1,1'-biphenyl **4a** (1.0 equiv., 0.50 mmol, 0.1549 g), Ad_2P^nBu (0.10 equiv., 0.05 mmol, 0.0178 g) was used instead of Ruphos (0.10 equiv., 0.05 mmol, 0.0233 g). The product was isolated in

78% yield (65.2 mg) via flash chromatography (petroleum ether/ethyl acetate = 20 : 1). R_f = 0.4. Yellow solid. **Analytical data :** ^{1}H NMR (400 MHz, DMSO-d$_6$) δ 11.23 (s, 1H), 8.10 (d, *J* = 7.8 Hz, 2H), 7.49 (d, *J* = 8.1 Hz, 2H), 7.39 – 7.34 (m, 2H), 7.16 – 7.12 (m, 2H); ^{13}C NMR (100 MHz, DMSO-d$_6$) δ 139.69, 125.48, 122.36, 120.13, 118.45, 110.90. IR (neat, cm-1) 572.90, 618.14, 655.38, 721.40, 745.76, 756.11, 840.88, 857.73, 927.66, 994.70, 1010.16, 1107.13, 1139.67, 1158.92, 1205.49, 1234.14, 1324.57, 1335.72, 1393.10, 1449.45, 1492.10, 1598.04, 1625.59, 1934.88, 3049.26, 3415.48. HRMS calcd. for $C_{12}H_{10}N$: 168.0808 [M+H$^+$]; found (ESI+): 168.0807. Mp: 242-244 °C. The spectroscopic data match a literature report.[9]

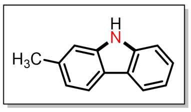

Compound 5b: 2-methyl-9H-carbazole. **Procedure:** The title compound was synthesized via *General Procedure A* from 2,2'-dibromo-4-methyl-1,1'-biphenyl[10] **4b** (1.0 equiv., 0.50 mmol, 0.1620 g), Ad_2P^nBu (0.10 equiv., 0.05 mmol, 0.0178 g) was used instead of Ruphos (0.10 equiv., 0.05 mmol, 0.0233 g). The product was isolated in 71% yield (64.3 mg) via flash chromatography (petroleum ether/ethyl acetate = 20 : 1). R_f = 0.4. Yellow solid. **Analytical data :** ^{1}H NMR (400 MHz, DMSO-d$_6$) δ 11.08 (s, 1H), 8.01 (d, *J* = 7.8 Hz, 1H), 7.94 (d, *J* = 7.9 Hz, 1H), 7.42 (d, *J* = 8.1 Hz, 1H), 7.33 – 7.29 (m, 1H), 7.25 (s, 1H), 7.15 – 7.05 (m, 1H), 6.95 (d, *J* = 8.0 Hz, 1H), 2.45 (s, 3H); ^{13}C NMR (100 MHz, DMSO-d$_6$) 140.18, 139.69, 134.97, 124.93, 122.47, 120.14, 120.02, 119.84, 119.77, 118.35, 110.88, 110.78, 21.69. IR (neat, cm-1) 571.86, 657.42, 724.88, 744.23, 765.02, 805.74, 863.62, 928.36, 997.79, 1016.28, 1109.82, 1130.67, 1149.17, 1204.62, 1240.87, 1324.67, 1374.52, 1439.43, 1459.02, 1488.10, 1604.01, 1631.68, 2855.31, 2914.01, 3048.21, 3396.69. HRMS calcd. for $C_{13}H_{12}N$: 182.0964 [M+H$^+$]; found (ESI+): 182.0963. Mp: 220-222 °C. The spectroscopic data match a literature report.[11]

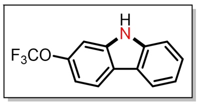

Compound 5c: 2-(trifluoromethoxy)-9H-carbazole. **Procedure:** The title compound was synthesized via *General Procedure A* from 2,2'-dibromo-4-(trifluoromethoxy)-1,1'-biphenyl[10] **4c** (1.0 equiv., 0.50 mmol, 0.1969 g), Ad_2P^nBu (0.10 equiv., 0.05 mmol, 0.0178 g) was used instead of Ruphos (0.10 equiv., 0.05 mmol, 0.0233 g). The product was isolated in 60% yield (75.3 mg) via flash chromatography (petroleum ether/ethyl acetate = 20 : 1). R_f = 0.4. Yellow solid. **Analytical data :** ^{1}H NMR (400 MHz, DMSO-d$_6$) δ 11.47 (s, 1H), 8.19 (d, *J* = 8.5 Hz, 1H), 8.17 – 8.09 (m, 1H), 7.51 (dt, *J* = 8.2, 0.9 Hz, 1H), 7.46 – 7.34 (m, 2H), 7.18 (ddd, *J* = 8.0, 7.1, 1.0 Hz, 1H), 7.10 (ddt, *J* = 8.6, 2.0, 1.0 Hz, 1H); ^{13}C NMR (100 MHz, DMSO-d$_6$) δ 146.44, 140.49, 139.77, 126.04, 121.63, 121.50, 121.42, 120.44, 119.19, 111.69, 111.23, 103.57; ^{19}F NMR (377 MHz, DMSO-d$_6$) δ -56.60. IR (neat, cm-1) 569.14, 608.86, 626.27, 669.57, 696.91, 726.94, 750.46, 765.50, 821.25, 855.82, 886.64, 937.51, 970.81, 1000.18, 1145.04, 1205.60, 1336.73, 1444.48, 1458.77, 1490.37, 1604.82, 3414.58. HRMS calcd. for $C_{13}H_9F_3NO$: 252.0631 [M+H$^+$]; found (ESI+): 252.0629. Mp: 178-180 °C.

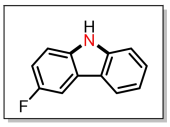

Compound 5d: 3-fluoro-9H-carbazole. **Procedure:** The title compound was synthesized via *General Procedure A* from 2,2'-dibromo-5-fluoro-1,1'-biphenyl[10] **4d** (1.0 equiv., 0.50 mmol, 0.1639 g), Ad_2P^nBu (0.10 equiv., 0.05 mmol, 0.0178 g) was used instead of Ruphos (0.10 equiv., 0.05 mmol, 0.0233 g). The product was isolated in 67% yield (62.0 mg) via flash chromatography (petroleum ether/ethyl acetate = 20 : 1). R_f = 0.4. Yellow solid. **Analytical data** : ^{1}H NMR (400 MHz, DMSO-d_6) δ 11.27 (s, 1H), 8.10 (d, *J* = 7.8 Hz, 1H), 7.93 (dd, *J* = 9.4, 2.6 Hz, 1H), 7.51 – 7.43 (m, 2H), 7.38 (ddd, *J* = 8.2, 7.0, 1.2 Hz, 1H), 7.21 (td, *J* = 9.2, 2.6 Hz, 1H), 7.13 (ddd, *J* = 8.1, 7.0, 1.0 Hz, 1H); ^{13}C NMR (100 MHz, DMSO-d_6) δ 156.38 (d, *J* = 230 Hz), 140.77, 136.17, 126.12, 122.83 (d, *J* = 10 Hz), 122.16 (d, *J* = 4.0 Hz), 120.65, 118.44, 113.15 (d, *J* = 25 Hz), 111.77 (d, *J* = 9.0 Hz), 111.21, 105.72 (d, *J* = 23 Hz). ^{19}F NMR (377 MHz, DMSO-d_6) δ -120.46. IR (neat, cm-1) 568.95, 597.42, 621.54, 635.42, 722.81, 744.63, 763.87, 805.65, 842.77, 864.16, 914.27, 1005.14, 1056.65, 1066.02, 1145.74, 1166.14, 1198.70, 1247.26, 1274.50, 1290.81, 1320.82, 1336.55, 1392.89, 1408.48, 1454.91, 1468.26, 1492.74, 1585.13, 1607.28, 2900.94, 2987.12, 3416.49, 3666.37. HRMS calcd. for $C_{12}H_9FN$: 186.0714 [M+H$^+$]; found (ESI+): 186.0712. Mp: 205-206 **°C**. The spectroscopic data match a literature report.[12]

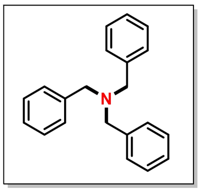

Compound 7: tribenzylamine. **Procedure:** The title compound was synthesized via *General Procedure A* from benzyl bromide **6** (85.5 mg, 0.50 mmol), without Pd catalyst. The product was isolated in 81% yield (38.8 mg) via flash chromatography (petroleum ether/ethyl acetate = 100 : 1). White solid. **Analytical data :** ^{1}H NMR (400 MHz, $CDCl_3$) δ 7.45 – 7.38 (m, 6H), 7.32 (dd, *J* = 8.4, 6.8 Hz, 6H), 7.26 – 7.19 (m, 3H), 3.56 (s, 6H); ^{13}C NMR (100 MHz, $CDCl_3$) δ 139.62, 128.71, 128.19, 126.83, 57.88. IR (neat, cm-1) 591.34, 624.43, 695.56, 740.94, 824.22, 879.10, 903.42, 971.83, 988.24, 1027.95, 1069.90, 1120.11, 1245.63, 1307.02, 1365.27, 1377.64, 1450.81, 1492.62, 1601.76, 2798.65, 3025.22. HRMS calcd. for $C_{20}H_{22}N$: 288.1747 [M+H$^+$]; found (ESI+): 288.1747. Mp: 90-92 **°C**. The spectroscopic data match a literature report.[13]

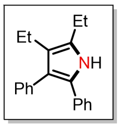

Compound 9: 2,3-diethyl-4,5-diphenyl-1H-pyrrole. **Procedure:** The title compound was synthesized via *General Procedure A* from ((1Z,3Z)-1,4-dibromo-3-ethylhexa-1,3-diene-1,2-diyl)dibenzene[14] **8** (209 mg, 0.50 mmol). The product was isolated in 83% yield (114.2 mg) via flash chromatography (petroleum ether/ethyl acetate = 50 : 1). R_f = 0.4. Brown oil. **Analytical data :** ^{1}H NMR (400 MHz, $CDCl_3$) δ 7.90 (s, 1H), 7.34 (d, *J* = 6.9 Hz, 1H), 7.32 – 7.29 (m, 2H), 7.28 – 7.23 (m, 2H), 7.22 – 7.17 (m, 2H), 7.17 – 7.13 (m, 2H), 7.13 – 7.07 (m, 1H), 2.72 (q, *J* = 7.6 Hz, 2H), 2.44 (q, *J* = 7.5 Hz, 2H), 1.32 (t, *J* = 7.6 Hz, 3H), 1.00 (t, *J* = 7.5 Hz, 3H); ^{13}C NMR (100 MHz, $CDCl_3$) δ 136.85, 133.35, 130.48, 130.14, 128.37, 128.17, 126.27, 126.04, 125.90, 125.55, 122.30, 121.69, 19.13, 17.39, 16.33, 14.43. IR (neat, cm-1) 553.57, 565.14, 572.67, 588.11, 614.66, 658.11, 694.92, 763.45, 799.65, 841.66, 853.37, 864.09, 871.76, 881.92, 914.61, 955.13, 1022.22, 1071.25, 1097.62, 1261.28, 1445.07, 1493.24, 1502.14, 1529.77,

1551.49, 1600.48, 1626.25, 1641.20, 1649.76, 1680.12, 1692.04, 1949.49, 2359.65, 2963.28, 3056.49, 3288.34. HRMS calcd. for $C_{20}H_{22}N$: 276.1747 [M+H$^+$]; found (ESI+): 276.1748.

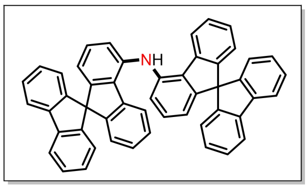

Compound 11: di(9,9'-spirobi[fluoren]-4-yl)amine. **Procedure:** The title compound was synthesized via *General Procedure A* from 4-bromo-9,9'-spirobi[fluorene] **10** (1.0 equiv., 0.50 mmol, 0.1970 g). The product was isolated in 63% yield (101.6 mg) via flash chromatography (petroleum ether/ethyl acetate = 20 : 1). R_f = 0.3. White solid. **Analytical data :** ^{1}H NMR (400 MHz, $CDCl_3$) δ 8.02 (d, *J* = 7.7 Hz, 2H), 7.86 (d, *J* = 7.6 Hz, 4H), 7.38 (t, *J* = 7.5 Hz, 4H), 7.31 (t, *J* = 7.6 Hz, 2H), 7.16 (t, *J* = 7.5 Hz, 4H), 7.13 – 7.07 (m, 4H), 7.04 (t, *J* = 7.6 Hz, 2H), 6.85 (d, *J* = 7.6 Hz, 4H), 6.78 (d, *J* = 7.5 Hz, 2H), 6.51 (s, 1H), 6.45 (d, *J* = 7.3 Hz, 2H); ^{13}C NMR (100 MHz, $CDCl_3$) δ 150.73, 148.90, 148.76, 141.76, 141.04, 139.21, 132.07, 128.81, 127.89, 127.82, 127.75, 127.16, 124.03, 122.44, 120.03, 119.25, 118.15, 66.00. IR (KBr, cm^{-1}) 418.1, 480.8, 595.2, 618.9, 652.9, 666.9, 724.5, 750.2, 783.0, 801.1, 906.7, 947.8, 1004.1, 1031.0, 1094.4, 1108.2, 1154.0, 1246.4, 1282.8, 1322.4, 1349.6, 1390.6, 1444.8, 1484.8, 1508.7, 1582.0, 1603.0, 1711.2, 1955.2, 2922.7, 3015.7, 3062.8, 3485.8. HRMS calcd. for $C_{50}H_{32}N$: 646.2529 [M+H$^+$]; found (ESI+): 646.2532. Mp: 180-182 **°C**.

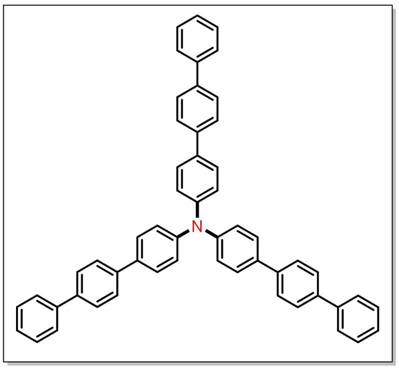

Compound 13: tri([1,1':4',1''-terphenyl]-4-yl)amine. **Procedure:** The title compound was synthesized via *General Procedure A* from 4-bromo-1,1':4',1''-terphenyl **12** (1.0 equiv., 0.50 mmol, 0.1540 g). The product was isolated in 89% yield (104.0 mg) via flash chromatography (petroleum ether/ethyl acetate = 100 : 1). White solid. **Analytical data :** ^{1}H NMR (400 MHz, $CDCl_3$) δ 7.67 (d, *J* = 15.9 Hz, 18H), 7.60 (d, *J* = 8.1 Hz, 6H), 7.47 (t, *J* = 7.5 Hz, 6H), 7.37 (d, *J* = 7.4 Hz, 3H), 7.28 (d, *J* = 8.4 Hz, 6H); ^{13}C NMR (100 MHz, $CDCl_3$) δ 146.87, 140.71, 139.71, 139.49, 135.13, 128.81, 127.82, 127.50, 127.29, 127.01, 124.49. IR (neat, cm^{-1}) 566.80, 614.57, 632.63, 693.90, 732.70, 763.75, 825.84, 1003.60, 1044.44, 1239.41, 1291.25, 1319.92, 1372.14, 1481.53, 1502.20, 1596.28, 1735.56, 3383.96. HRMS calcd. for $C_{54}H_{40}N$: 702.3155 [M+H$^+$]; found (ESI+): 702.3187. Mp: 262-264**°C**.

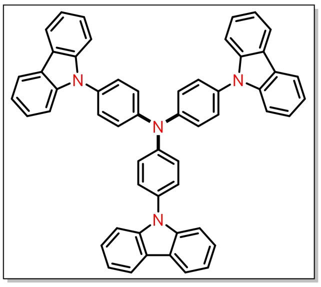

Compound 15: tris(4-(9H-carbazol-9-yl)phenyl)amine. **Procedure:** 9-(4-bromophenyl)-9H-carbazole **14** was prepared via literature method[15] from **5a.** The title compound was synthesized via *General Procedure A* from 9-(4-bromophenyl)-9H-carbazole **14** (1.0 equiv., 0.50 mmol, 0.1605 g). The product was isolated in 77% yield (95.1 mg) via flash chromatography (petroleum ether/ethyl acetate = 100 : 1). White solid. **Analytical data :** ^{1}H NMR (400 MHz, $CDCl_3$) δ 8.18 (d, *J* = 7.8 Hz, 6H), 7.63 – 7.56 (m, 6H), 7.56 – 7.50 (m, 12H), 7.46 (ddd, *J* = 8.2,

7.0, 1.2 Hz, 6H), 7.32 (ddd, *J* = 8.0, 7.0, 1.1 Hz, 6H); ^{13}C NMR (100 MHz, $CDCl_3$) δ 146.40, 140.96, 132.81, 128.26, 125.94, 125.34, 123.33, 120.35, 119.94, 109.79. IR (neat, cm^{-1}) 564.68, 611.53, 618.13, 637.69, 719.54, 744.08, 770.98, 814.05, 829.32, 839.06, 912.43, 928.90, 966.89, 1014.91, 1026.52, 1118.75, 1146.15, 1183.55, 1224.37, 1267.58, 1307.93, 1334.19, 1361.36, 1448.26, 1477.70, 1504.29, 1597.95, 1623.41, 3043.60. HRMS calcd. for $C_{54}H_{37}N_4$: 741.3013 [$M+H^+$]; found (ESI+): 741.2992. Mp: 297-298°**C**.

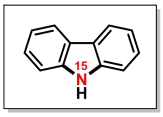

Compound ^{15}N-5a: 10*H*-carbazole-$^{15}N_2$. **Procedure:** The title compound was synthesized via *General Procedure B* from 2,2'-dibromo-1,1'-biphenyl **4a** (1.0 equiv., 0.50 mmol, 0.1549 g). The product was isolated in 76% yield (63.9 mg) via flash chromatography (petroleum ether/ethyl acetate = 20 : 1). R_f = 0.4. Yellow solid. **Analytical data :** ^{1}H NMR (400 MHz, DMSO-d_6) δ 11.24 (d, *J* = 96 Hz, 1H), 8.11 (dd, *J* = 7.8, 1.1 Hz, 2H), 7.48 (dq, *J* = 8.2, 1.0 Hz, 2H), 7.38 (ddd, *J* = 8.0, 7.1, 0.9 Hz, 2H), 7.15 (ddd, *J* = 7.9, 7.1, 1.1 Hz, 2H); ^{13}C NMR (100 MHz, DMSO-d_6) δ 139.59, 125.49, 122.36, 120.14, 118.46, 110.91; ^{15}N NMR (51 MHz, DMSO-d_6) δ 117.30 (d, *J* = 97.92 Hz). IR (neat, cm-1) 564.13, 572.05, 618.01, 630.12, 649.08, 723.51, 746.18, 797.71, 836.67, 846.85, 927.32, 995.27, 1010.01, 1105.04, 1139.38, 1158.78, 1231.21, 1260.48, 1324.84, 1335.13, 1385.12, 1448.19, 1488.96, 1597.50, 2916.76, 3407.34. HRMS calcd. for $C_{12}H_{10}{}^{15}N$: 169.0778 [$M+H^+$]; found (ESI+): 169.0779. Mp: 242-245°**C**.

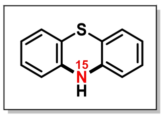

Compound ^{15}N-17: 10*H*-phenothiazine-$^{15}N_2$. **Procedure:** The title compound was synthesized via *General Procedure B* from 1-bromo-2-[(2-bromophenyl)thio]benzene[16] **16** (171 mg, 0.50 mmol), Ad_2P^nBu (0.10 equiv., 0.05 mmol, 0.0178 g) was used instead of Ruphos (0.10 equiv., 0.05 mmol, 0.0233 g). The product was isolated in 63% yield (62.9 mg) via flash chromatography (petroleum ether/ethyl acetate = 40 : 1). R_f = 0.3. Yellow solid. **Analytical data :** ^{1}H NMR (400 MHz, DMSO-d_6) δ 8.58 (d, *J* = 88 Hz, 1H), 6.98 (td, *J* = 7.6, 1.5 Hz, 2H), 6.91 (dd, *J* = 7.6, 1.4 Hz, 2H), 6.75 (td, *J* = 7.5, 1.3 Hz, 2H), 6.71 – 6.63 (m, 2H); ^{13}C NMR (100 MHz, DMSO-d_6) δ 142.16, 127.56, 126.25, 121.78, 116.31, 114.42; ^{15}N NMR (51 MHz, DMSO-d_6) δ 117.31 (d, *J* = 98.43 Hz). IR (neat, cm-1) 567.42, 617.34, 654.65, 688.68, 726.23, 737.38, 862.32, 879.23, 925.05, 1056.72, 1065.95, 1075.28, 1155.13, 1232.37, 1300.96, 1325.27, 1382.42, 1393.87, 1406.66, 1443.10, 1459.27, 1571.03, 1597.89, 2900.9, 2987.31, 3675.18. HRMS calcd. for $C_{12}H_{10}S^{15}N$: 201.0498 [$M+H^+$]; found (ESI+): 201.0499. Mp: 182-184 °**C.**

(V) NMR Spectral Data for Products

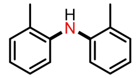

2a

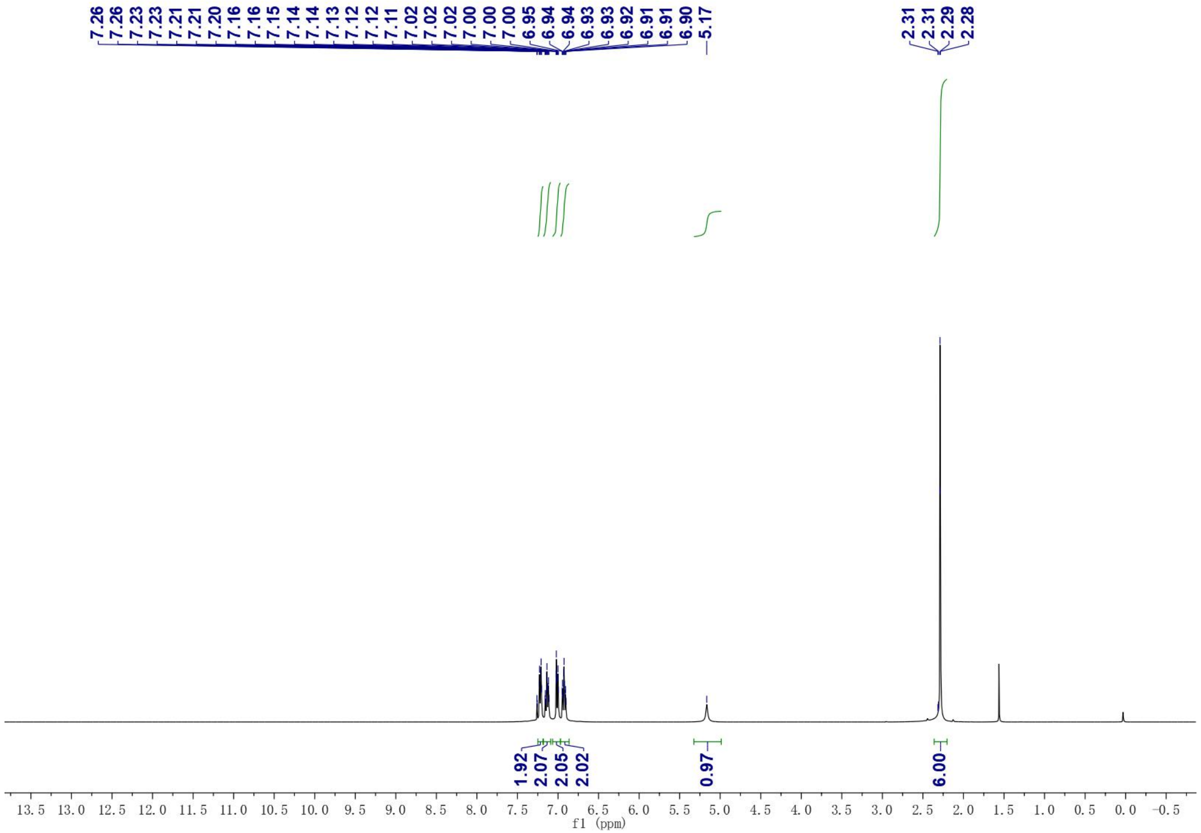

Supplementary Figure 1. ^{1}H NMR Spectrum of **2a**

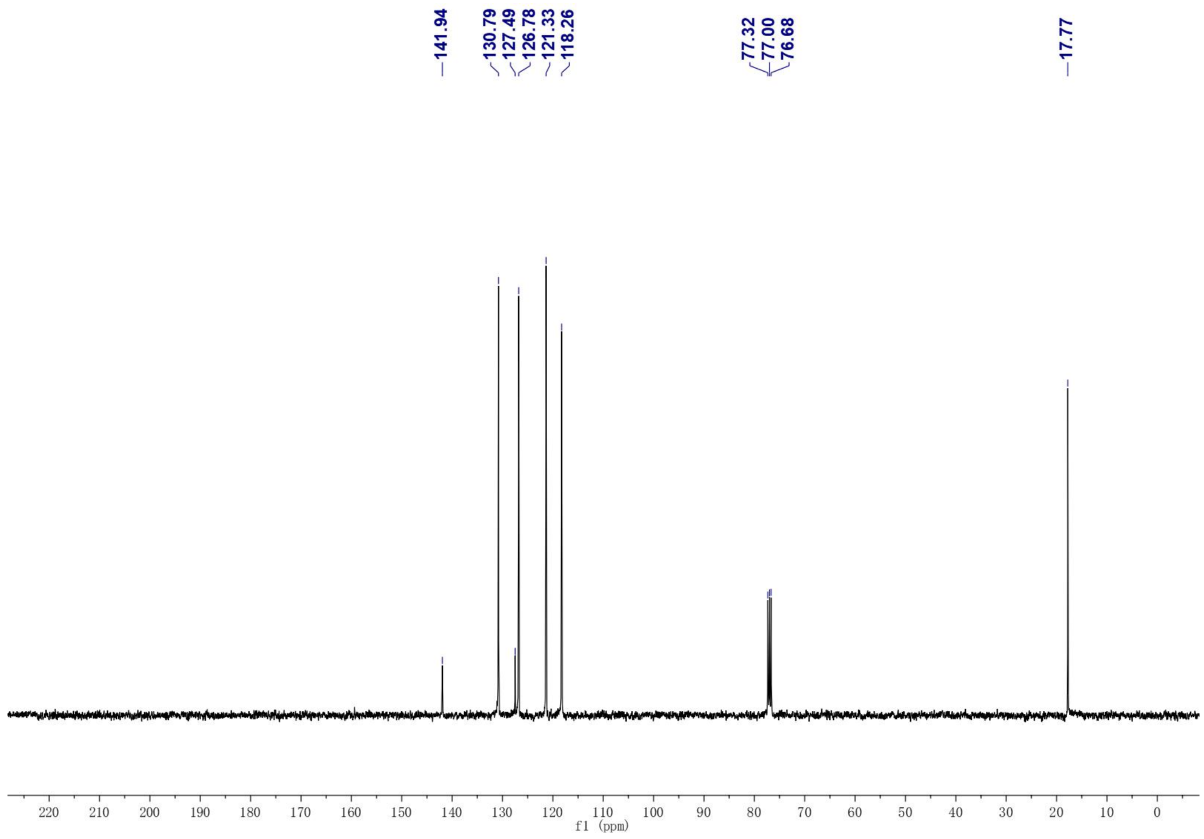

Supplementary Figure 2. ^{13}C NMR Spectrum of **2a**

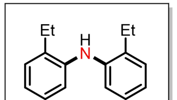

2b

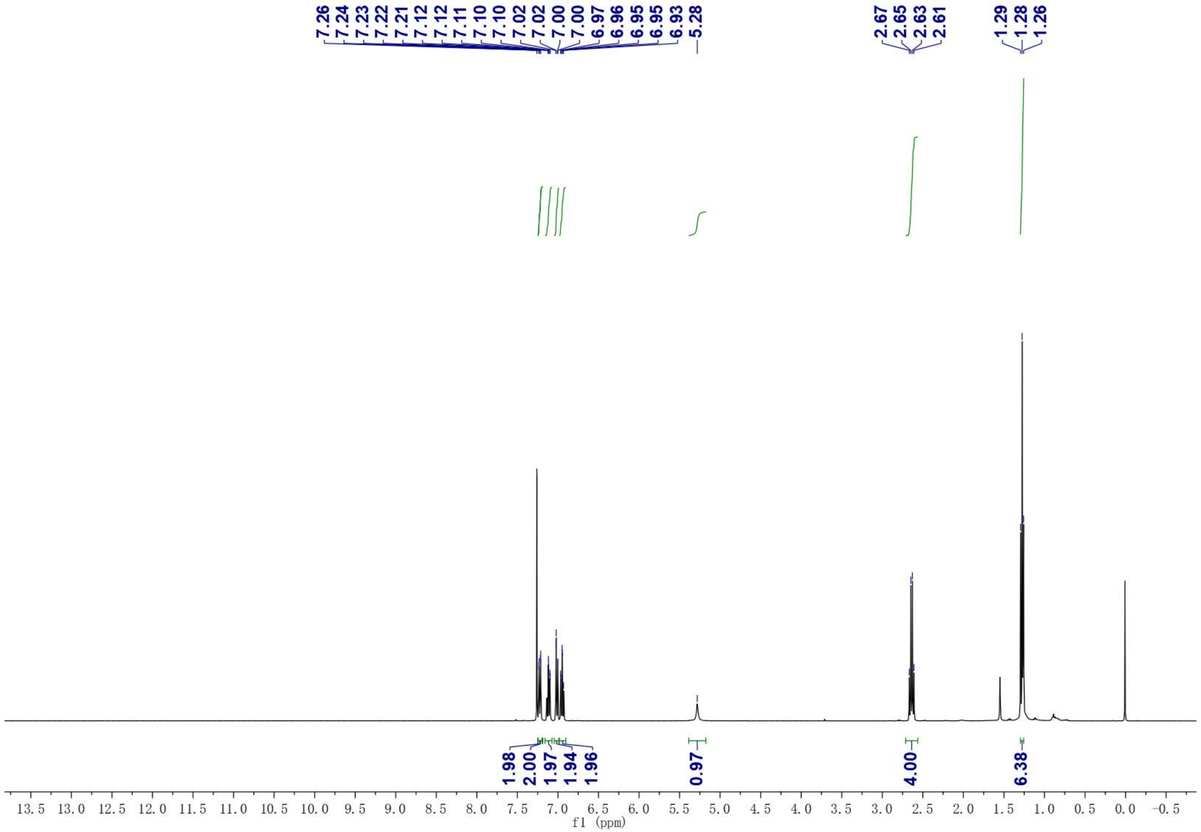

Supplementary Figure 3. ^{1}H NMR Spectrum of **2b**

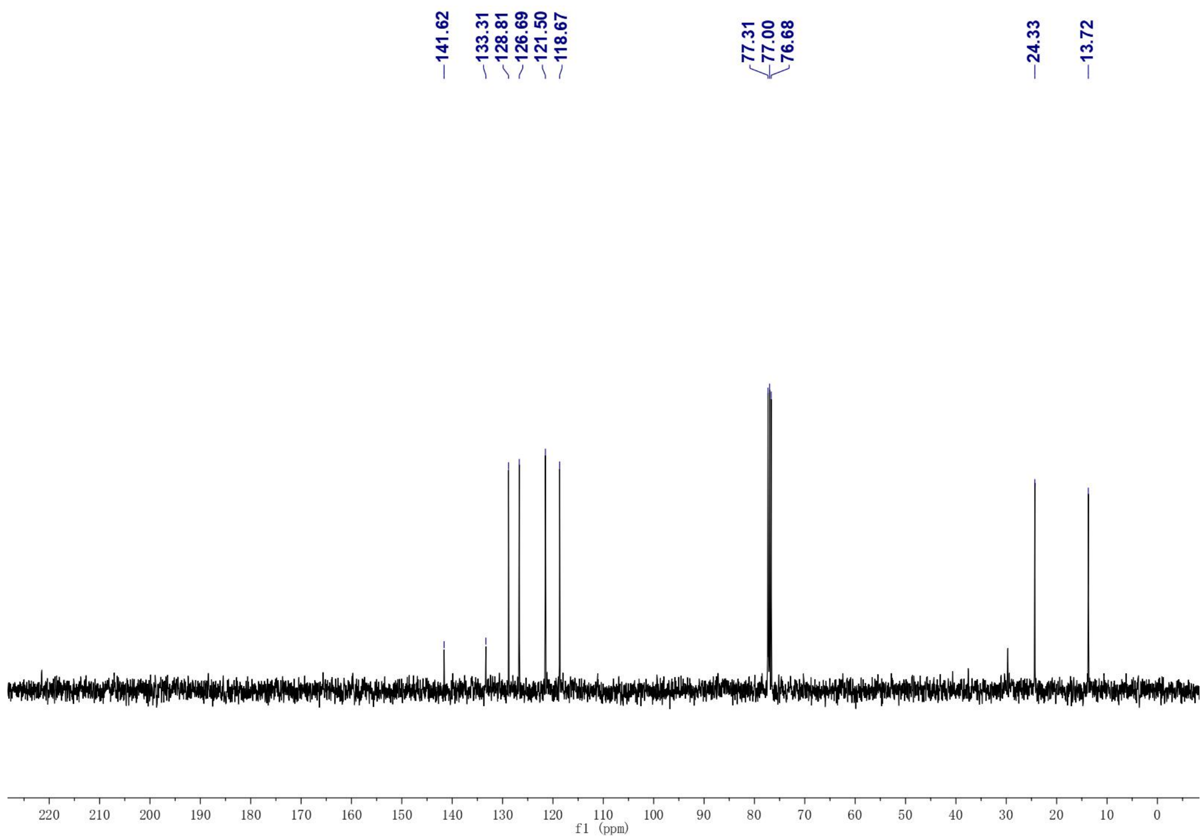

Supplementary Figure 4. ^{13}C NMR Spectrum of **2b**

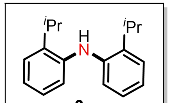

2c

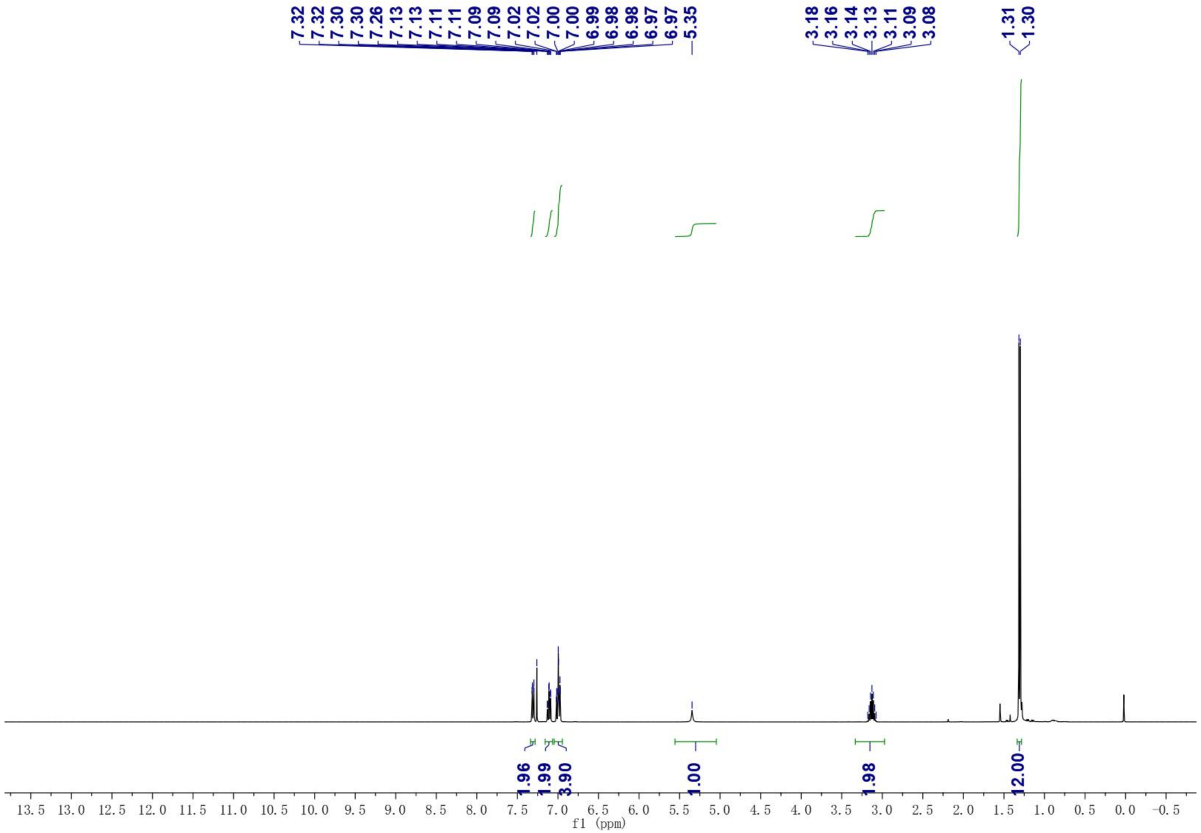

Supplementary Figure 5. ^{1}H NMR Spectrum of **2c**

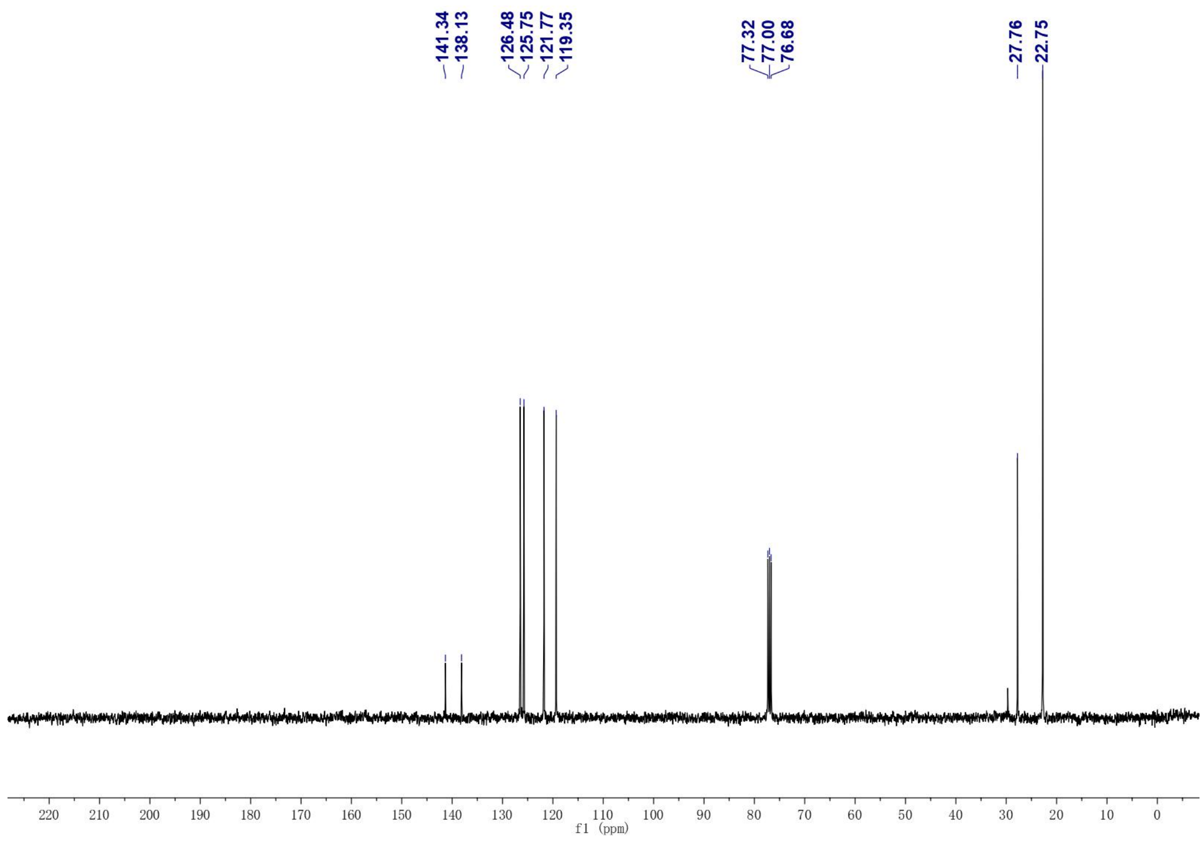

Supplementary Figure 6. ^{13}C NMR Spectrum of **2c**

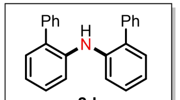

2d

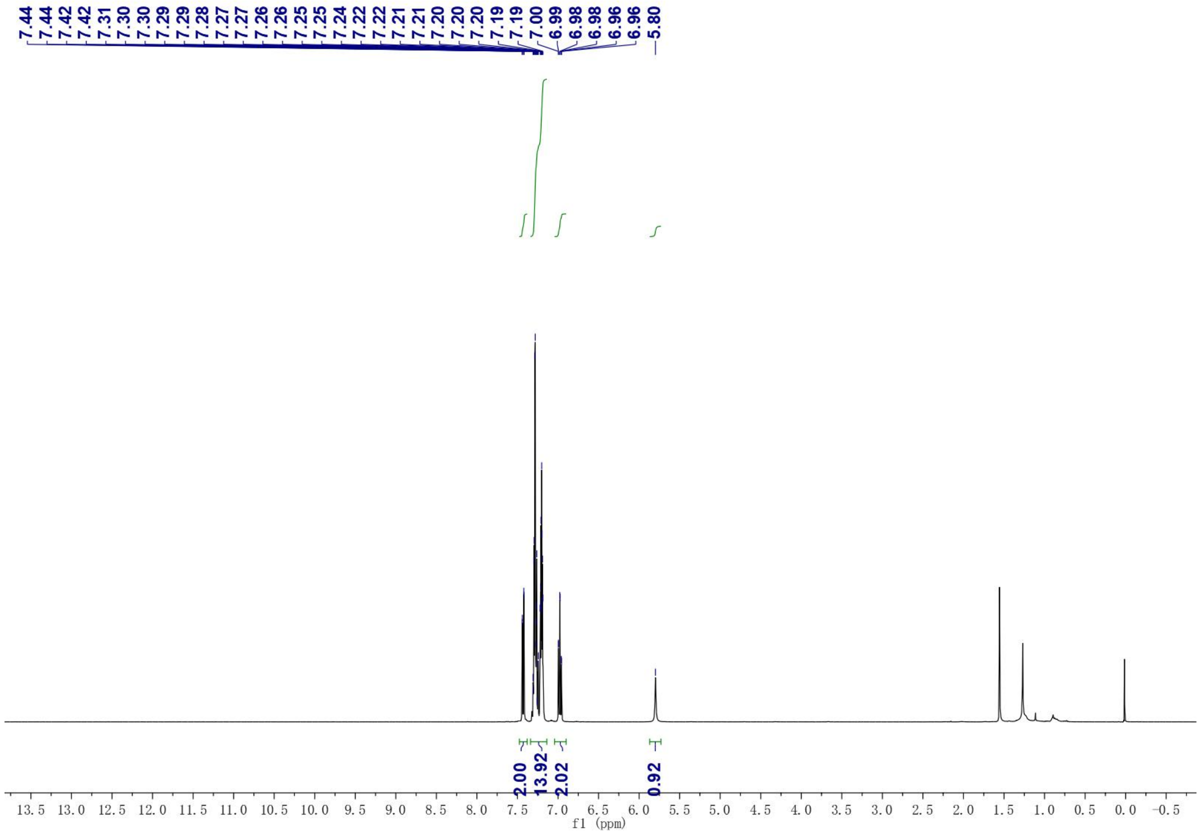

Supplementary Figure 7. ^{1}H NMR Spectrum of 2**d**

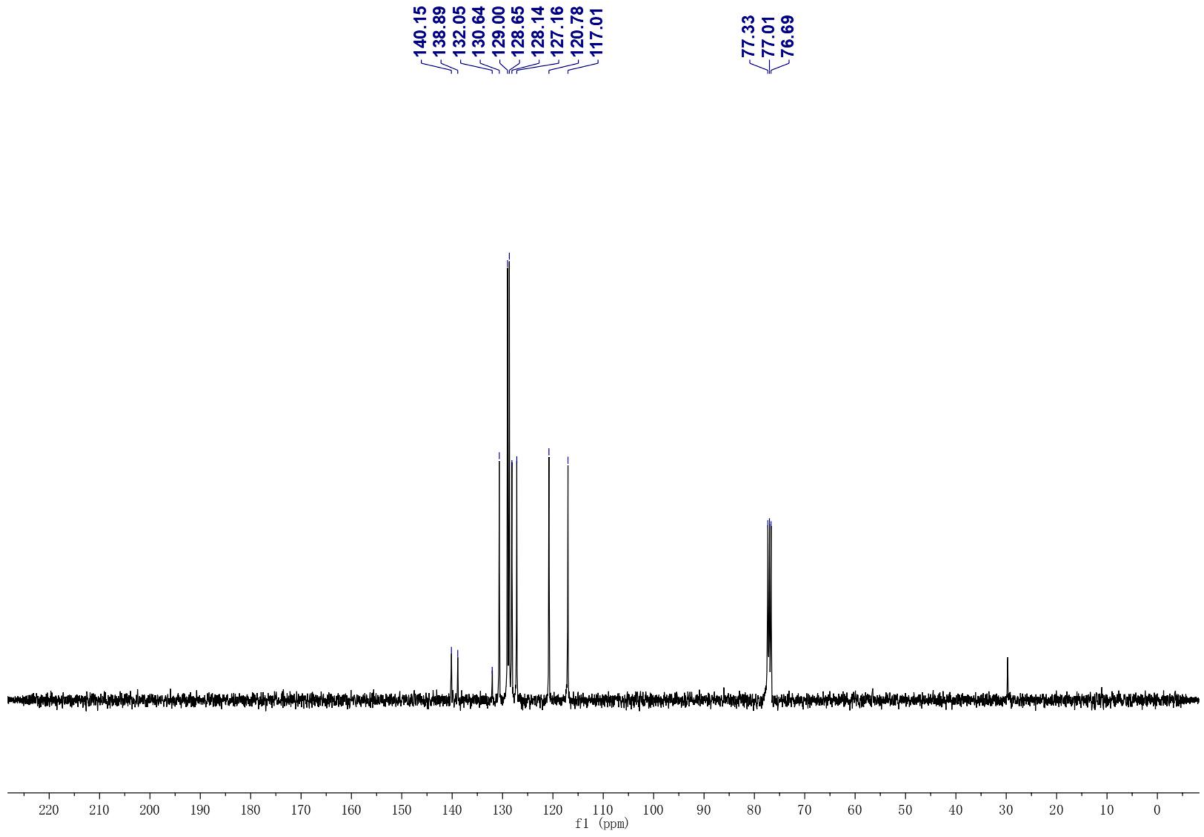

Supplementary Figure 8. ^{13}C NMR Spectrum of **2d**

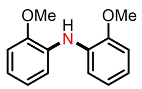

2e

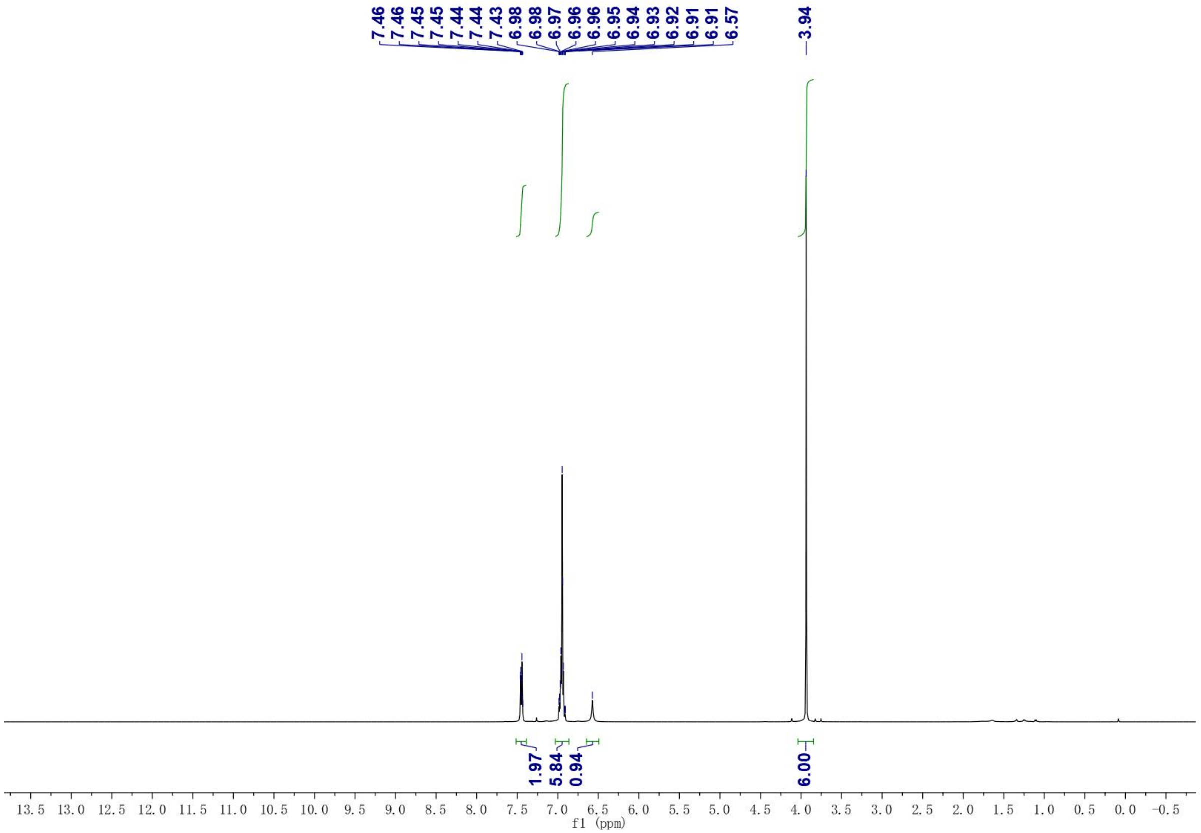

Supplementary Figure 9. ^{1}H NMR Spectrum of **2e**

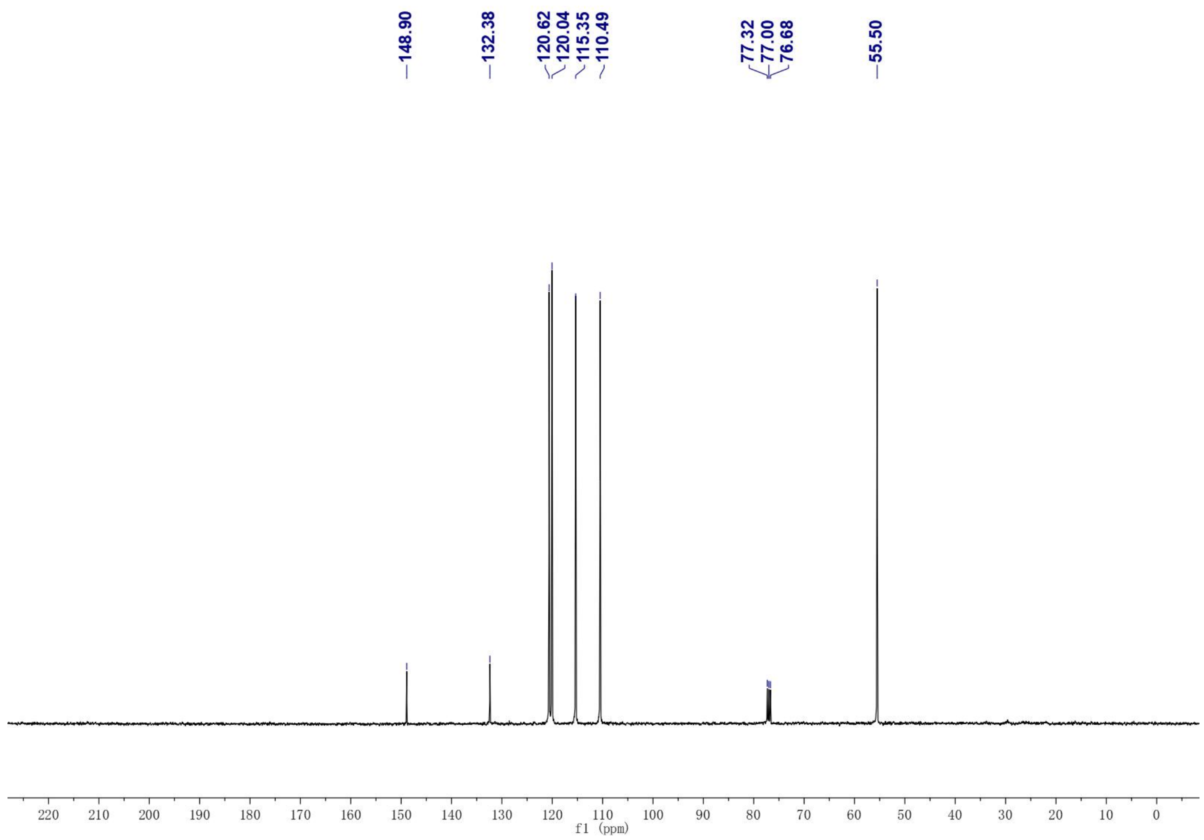

Supplementary Figure 10. ^{13}C NMR Spectrum of **2e**

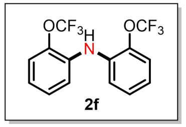

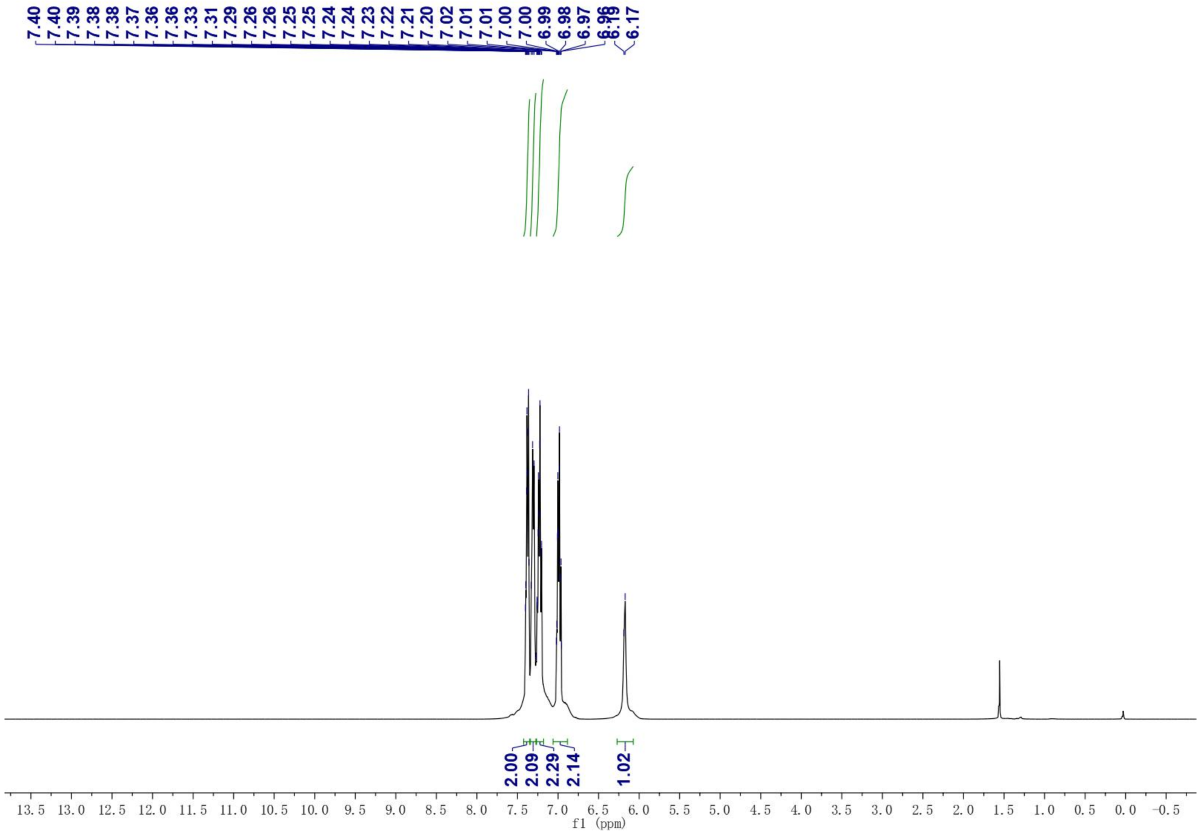

Supplementary Figure 11. ^{1}H NMR Spectrum of **2f**

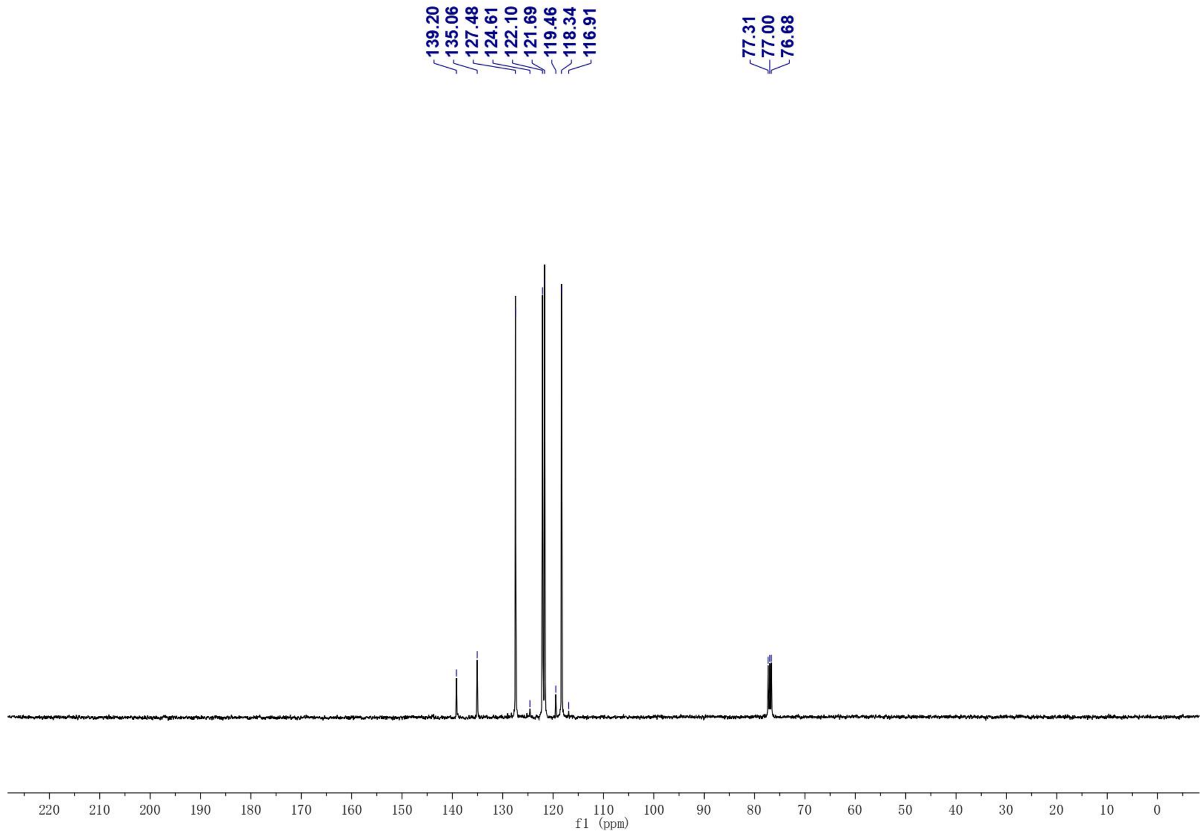

Supplementary Figure 12. ^{13}C NMR Spectrum of **2f**

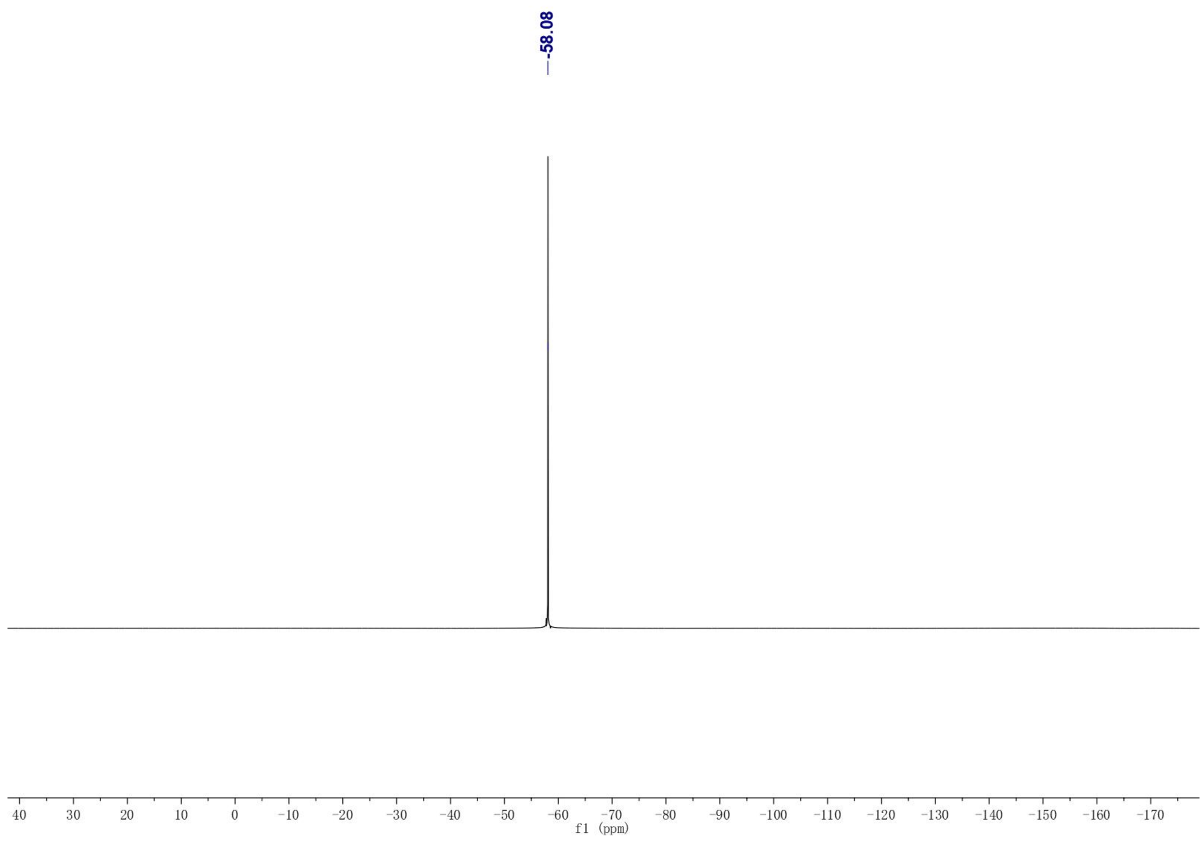

Supplementary Figure 13. ^{19}F NMR Spectrum of **2f**

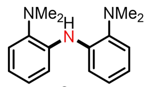

2g

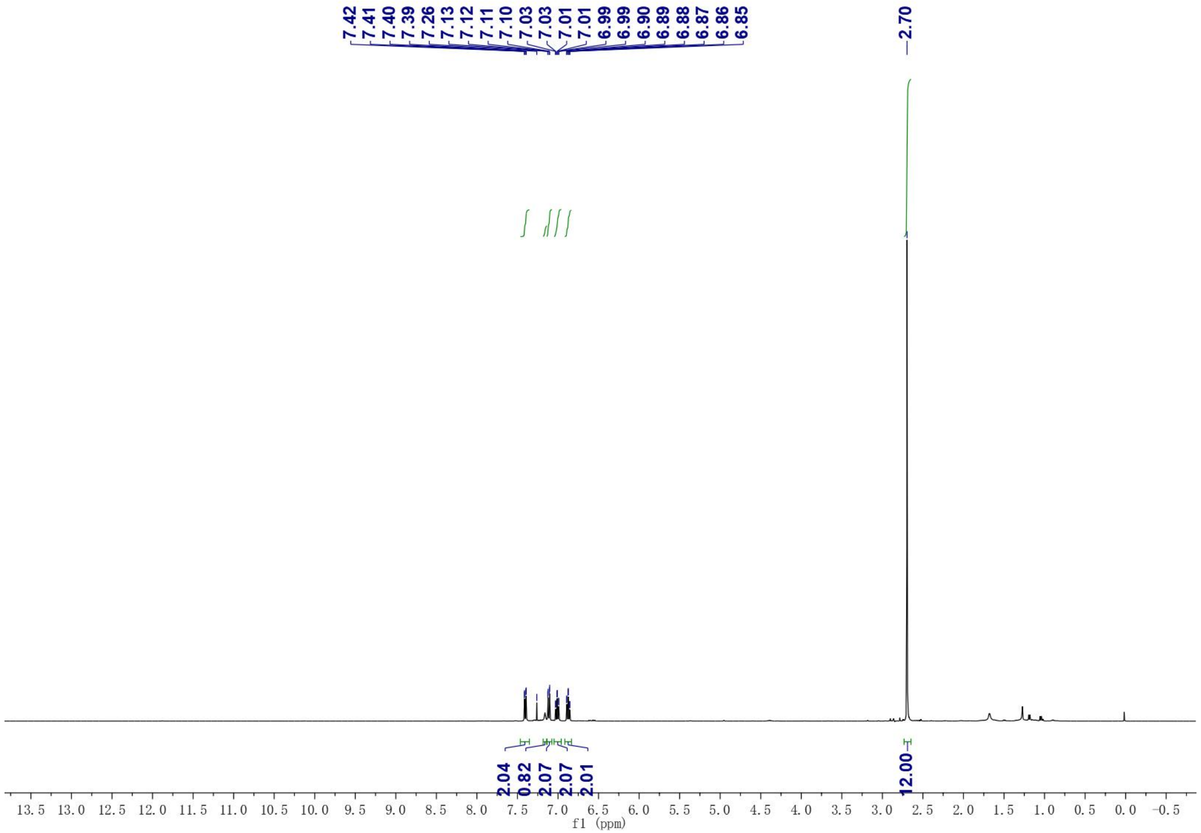

Supplementary Figure 14. ^{1}H NMR Spectrum of **2g**

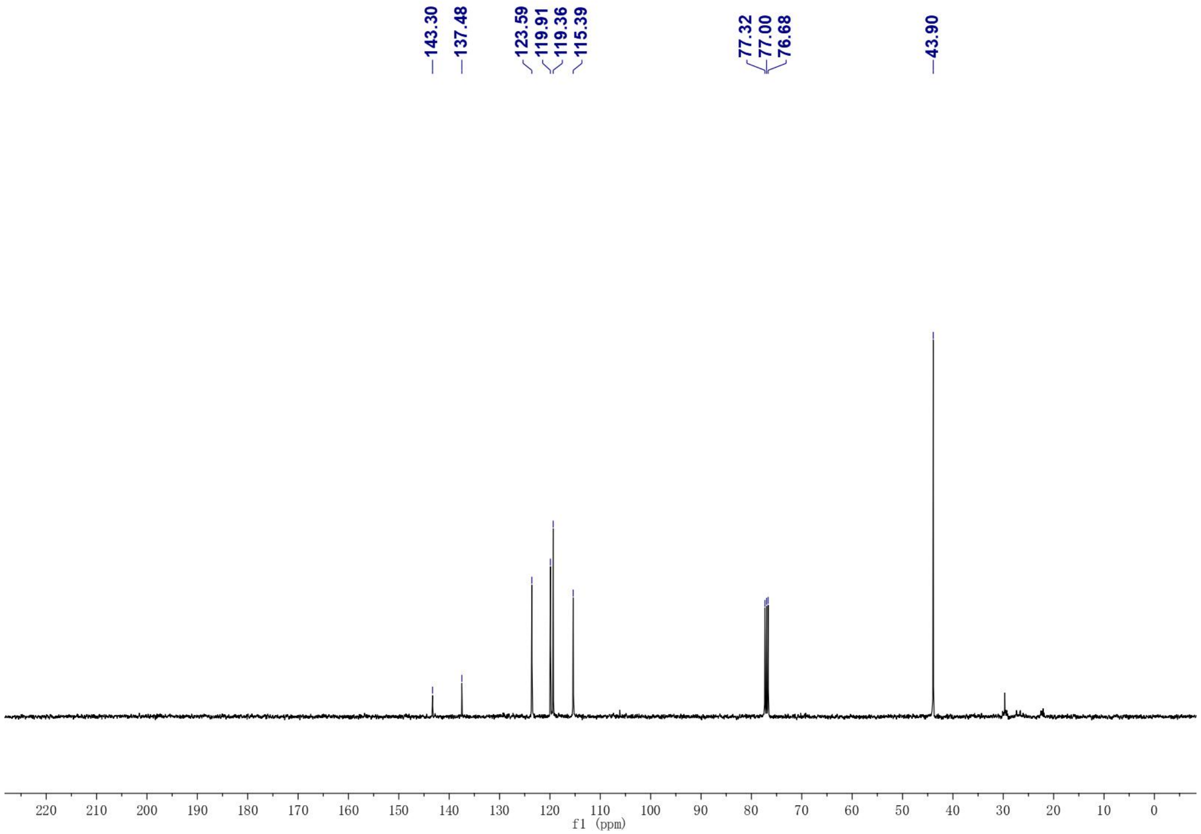

Supplementary Figure 15. ^{13}C NMR Spectrum of **2g**

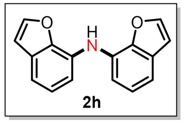

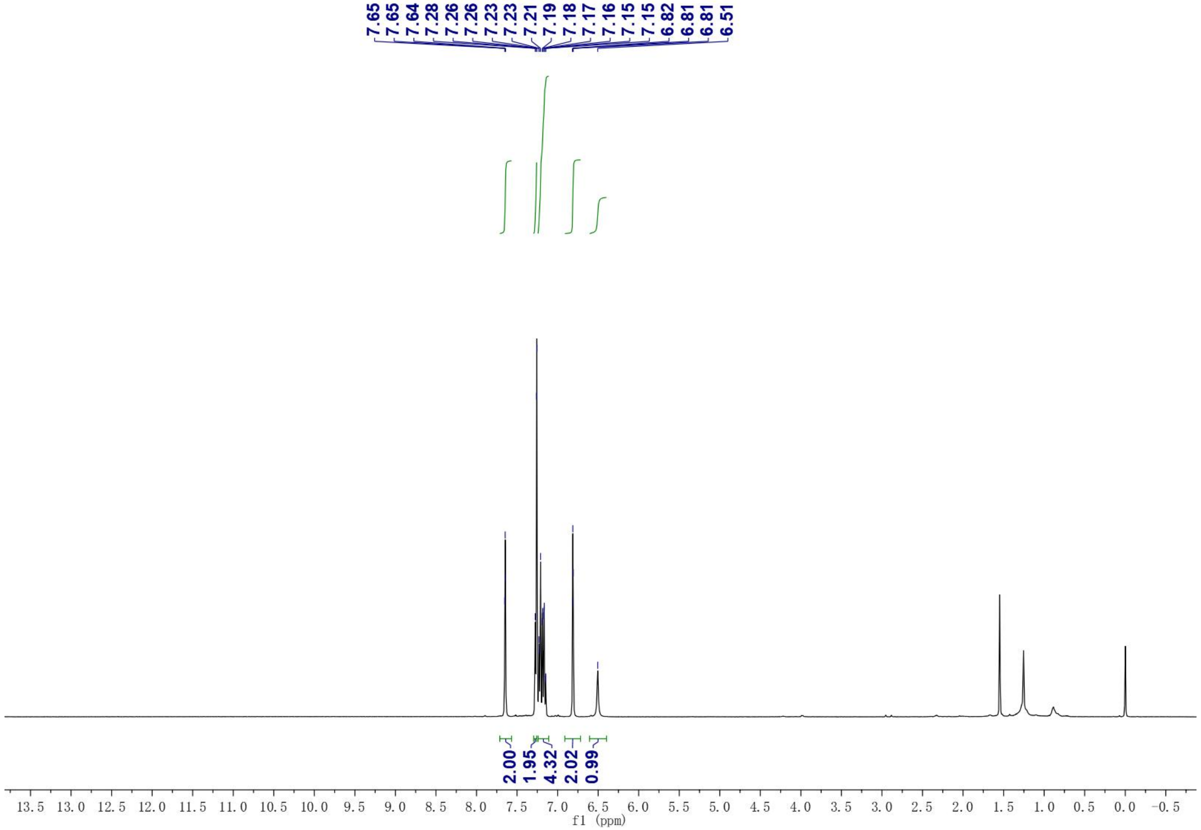

Supplementary Figure 16. ^{1}H NMR Spectrum of **2h**

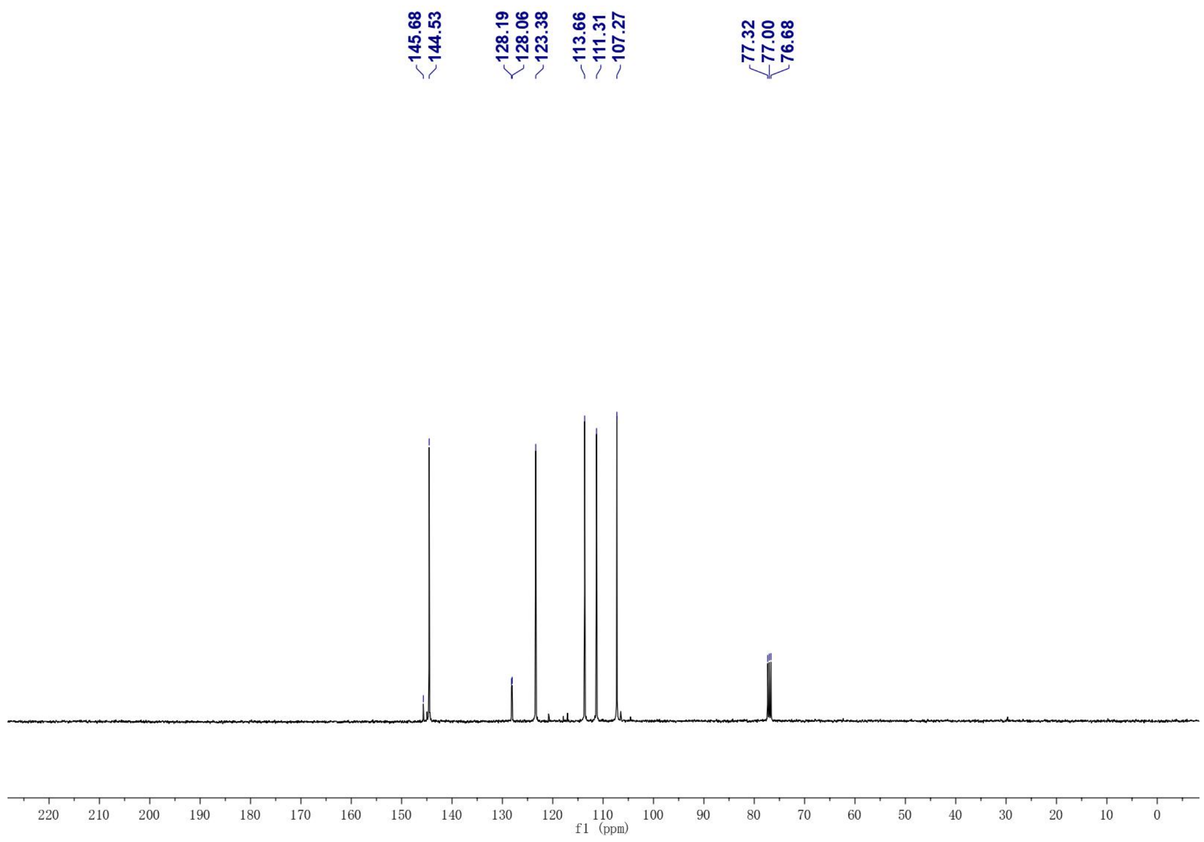

Supplementary Figure 17. ^{13}C NMR Spectrum of **2h**

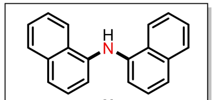

2i

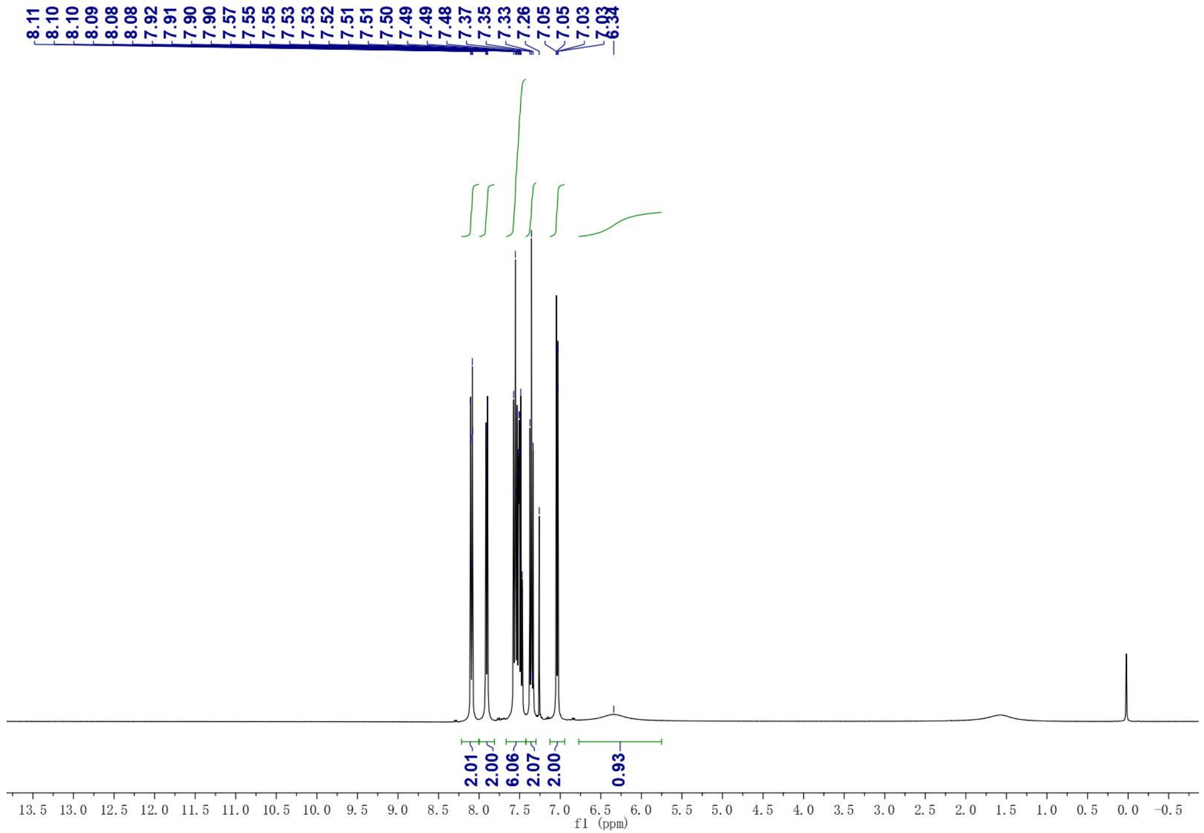

Supplementary Figure 18. ^{1}H NMR Spectrum of **2i**

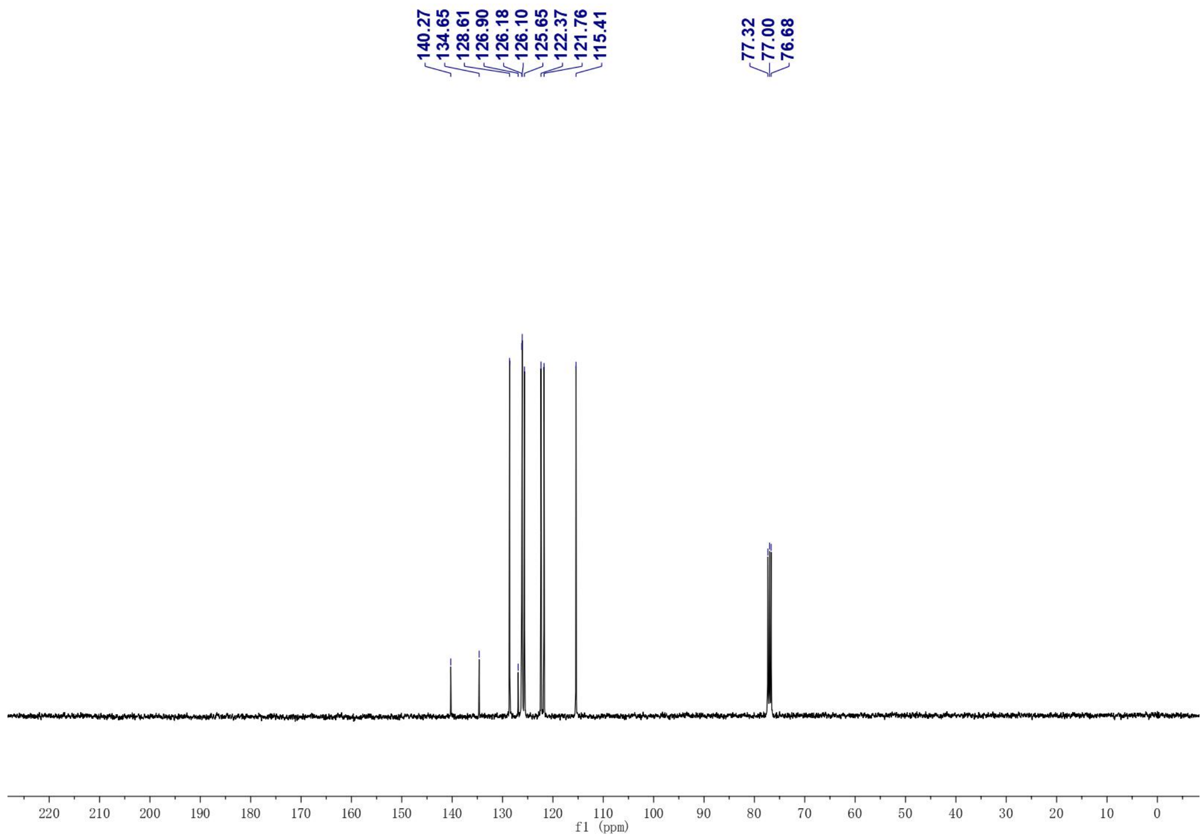

Supplementary Figure 19. ^{13}C NMR Spectrum of **2i**

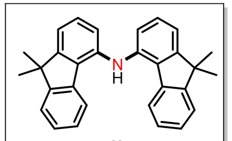

2j

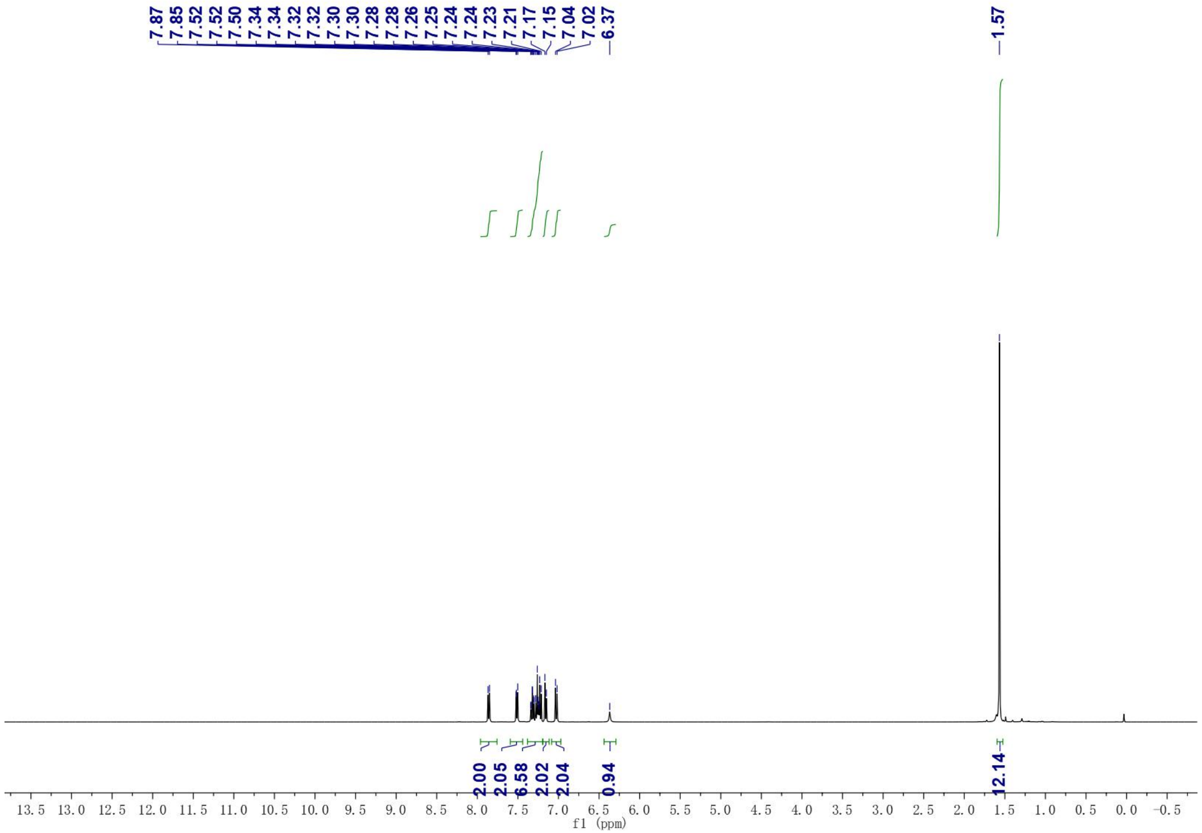

Supplementary Figure 20. ^{1}H NMR Spectrum of **2j**

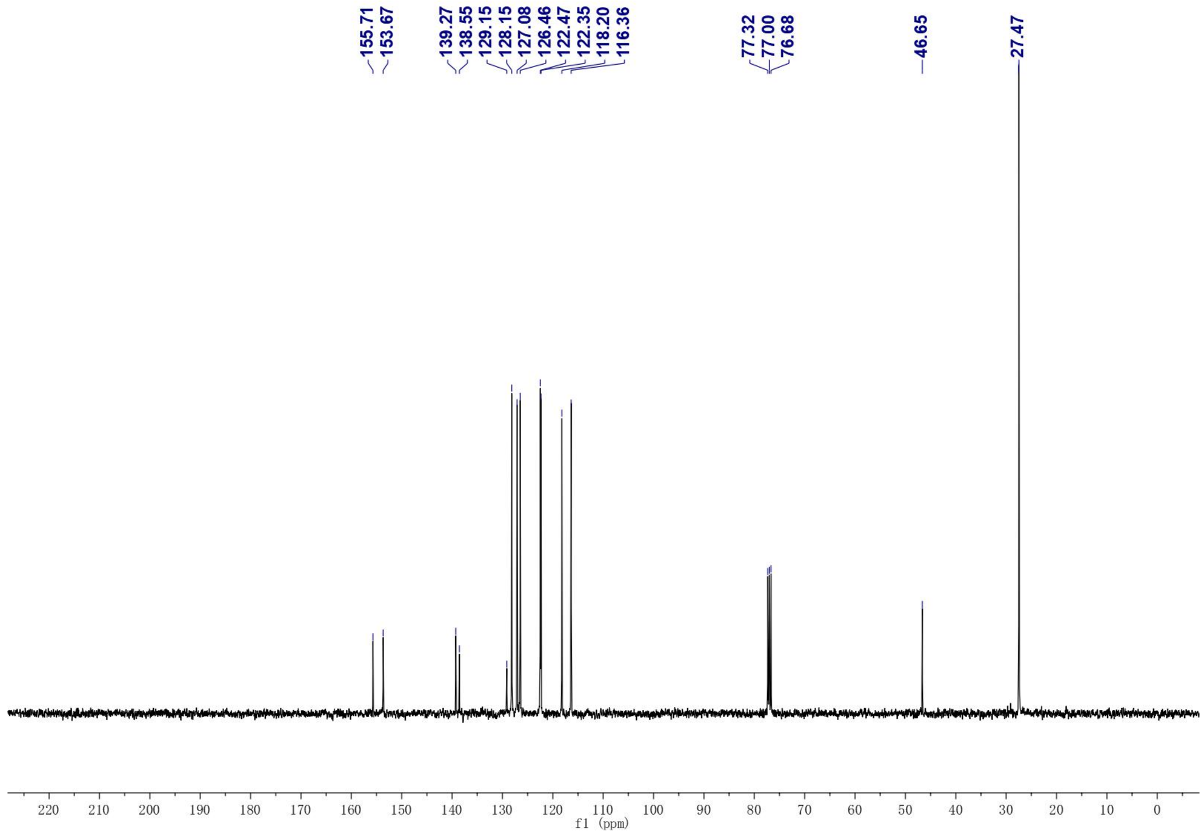

Supplementary Figure 21. ^{13}C NMR Spectrum of **2j**

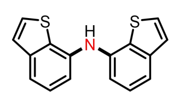

2k

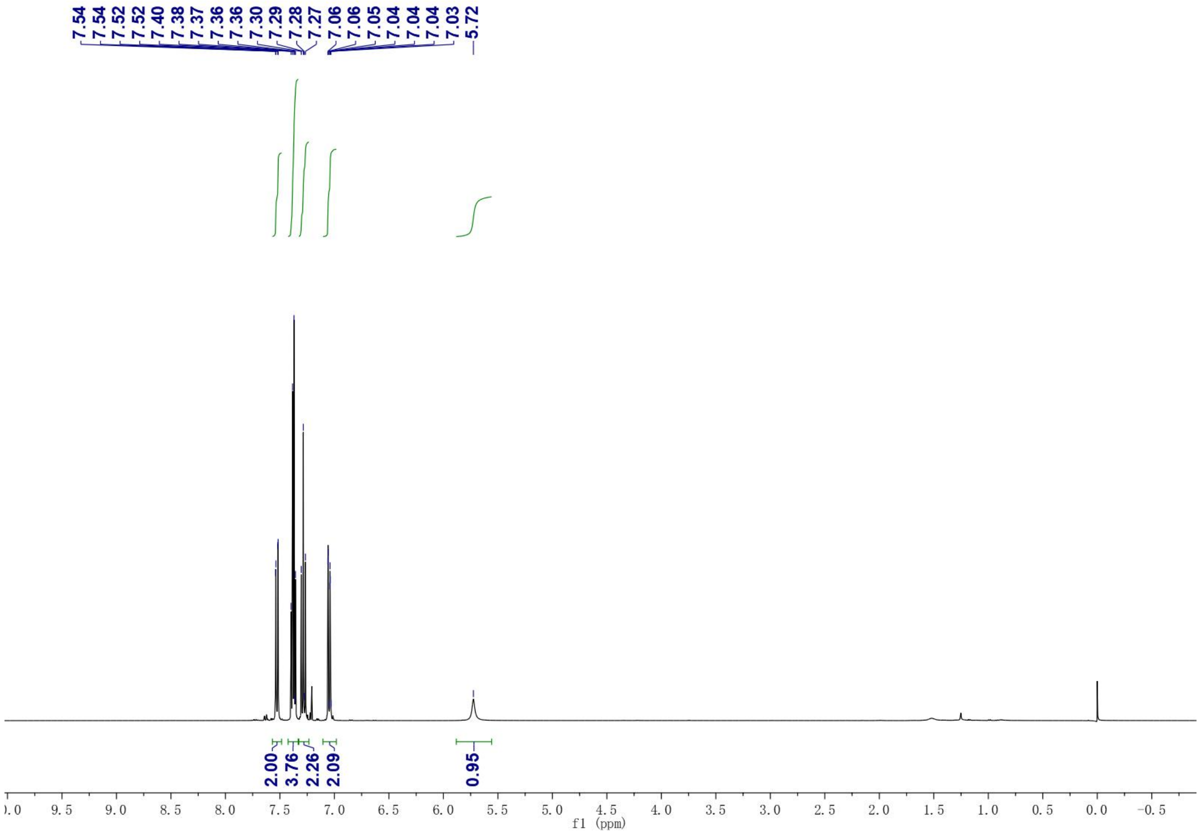

Supplementary Figure 22. ^{1}H NMR Spectrum of **2k**

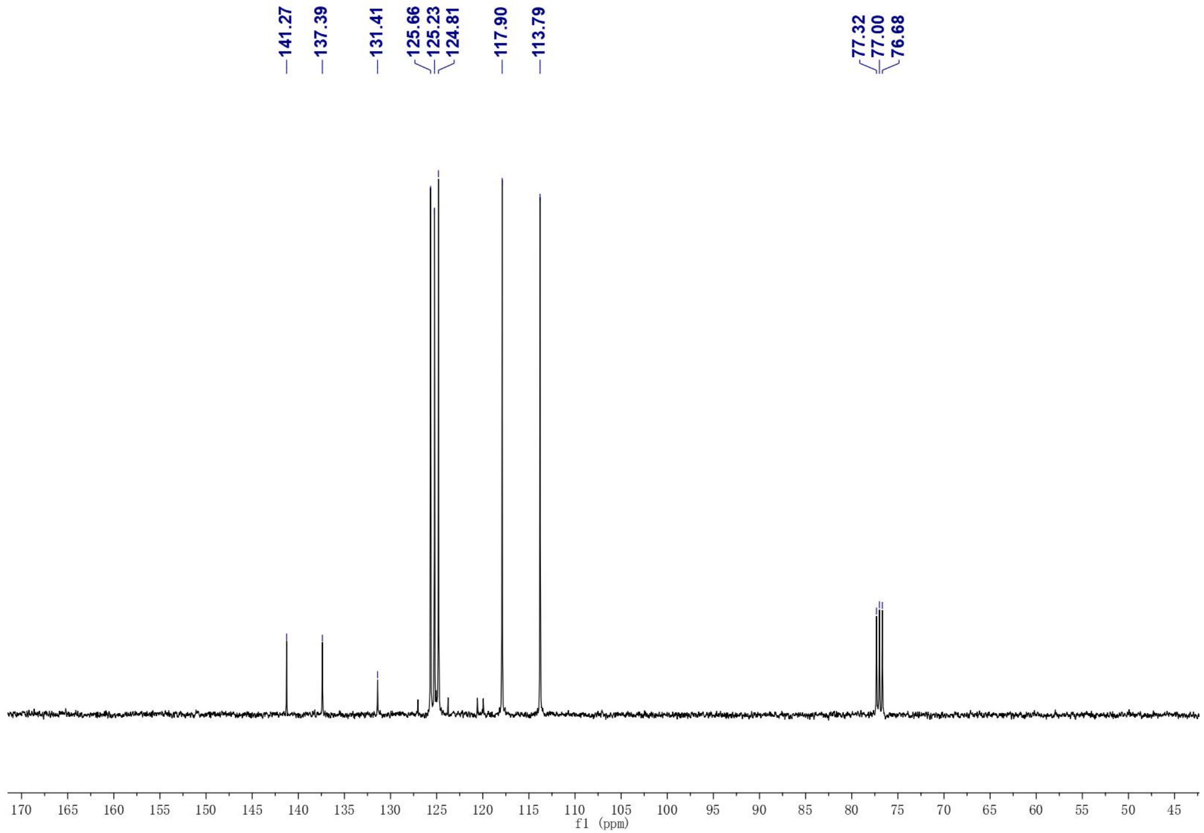

Supplementary Figure 23. ^{13}C NMR Spectrum of **2k**

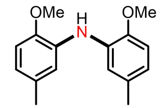

2l

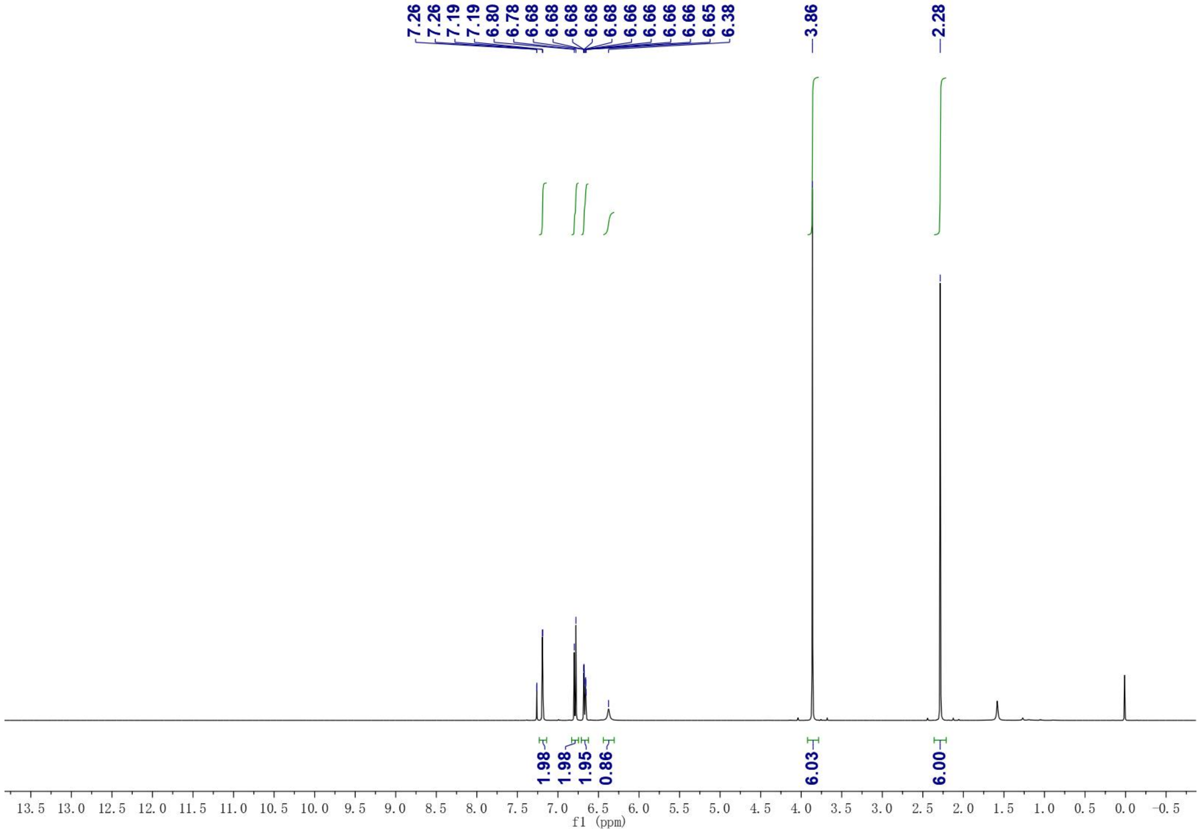

Supplementary Figure 24. ^{1}H NMR Spectrum of **2l**

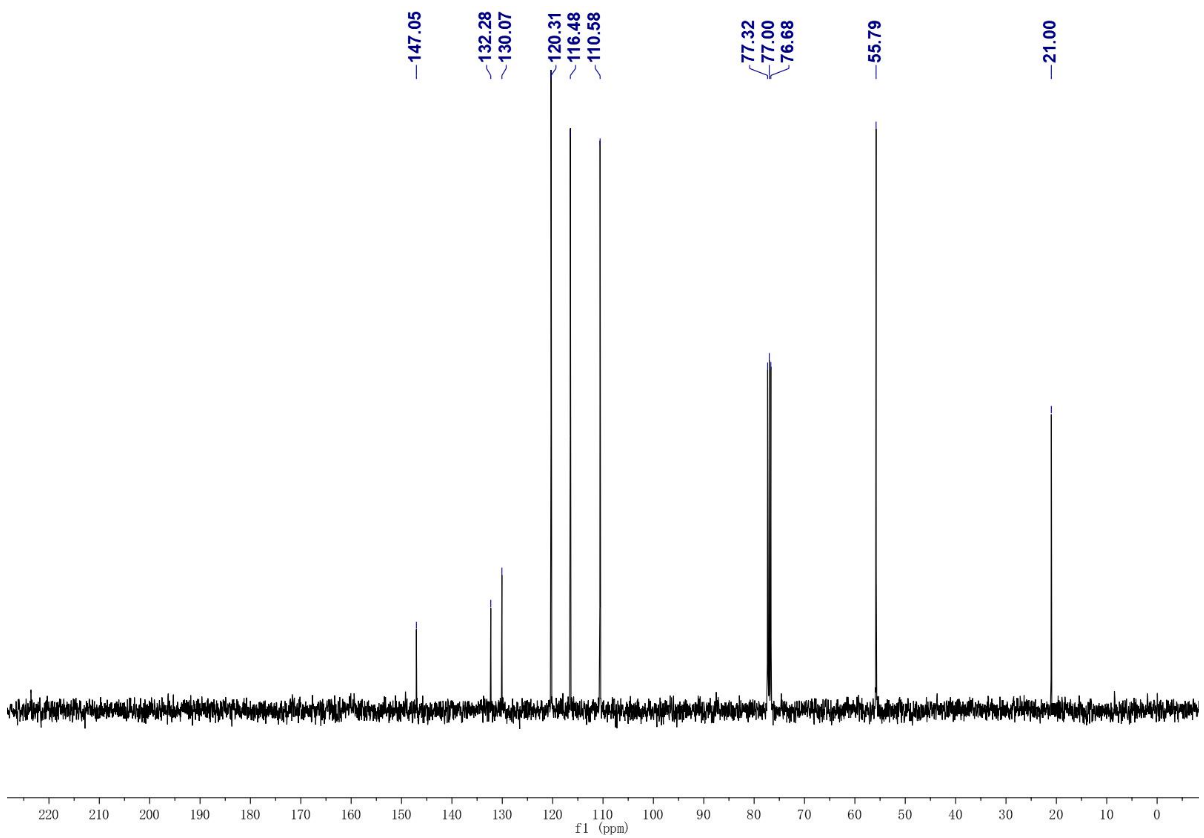

Supplementary Figure 25. ^{13}C NMR Spectrum of **2l**

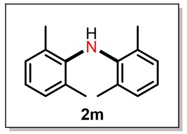

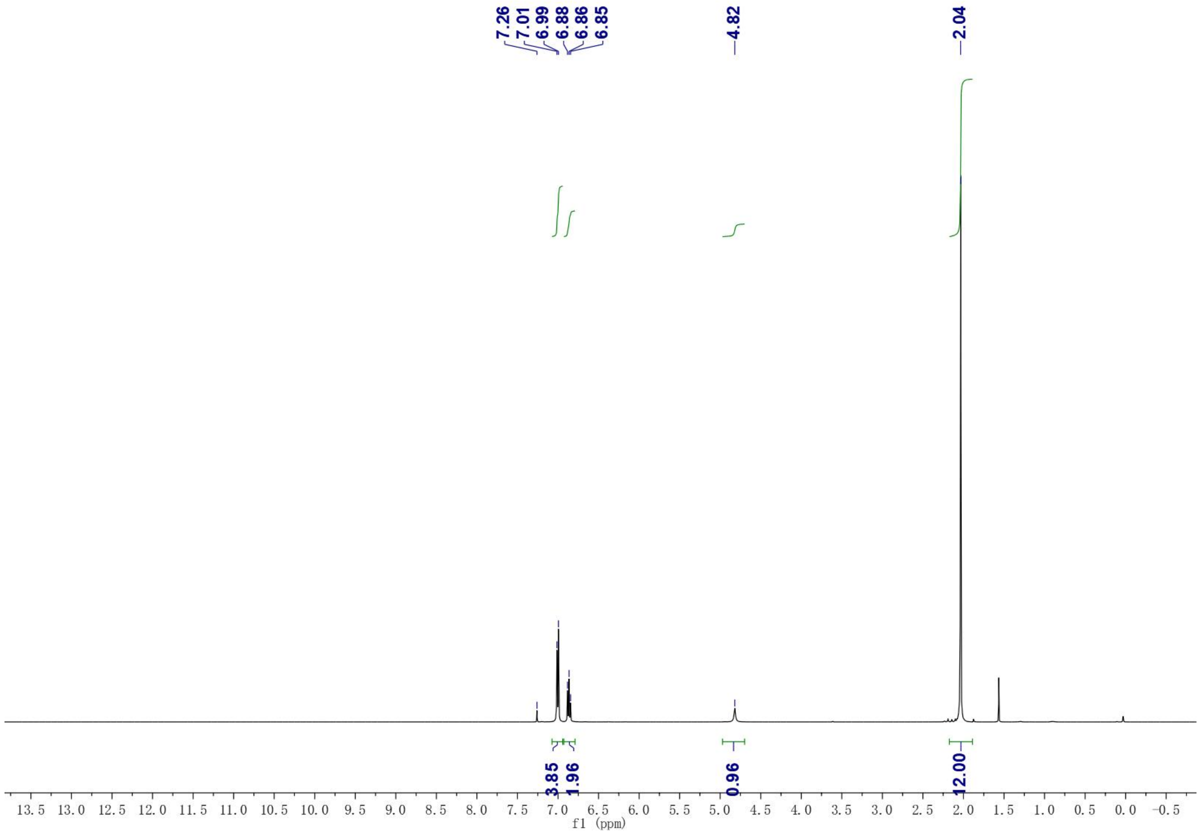

Supplementary Figure 26. ^{1}H NMR Spectrum of **2m**

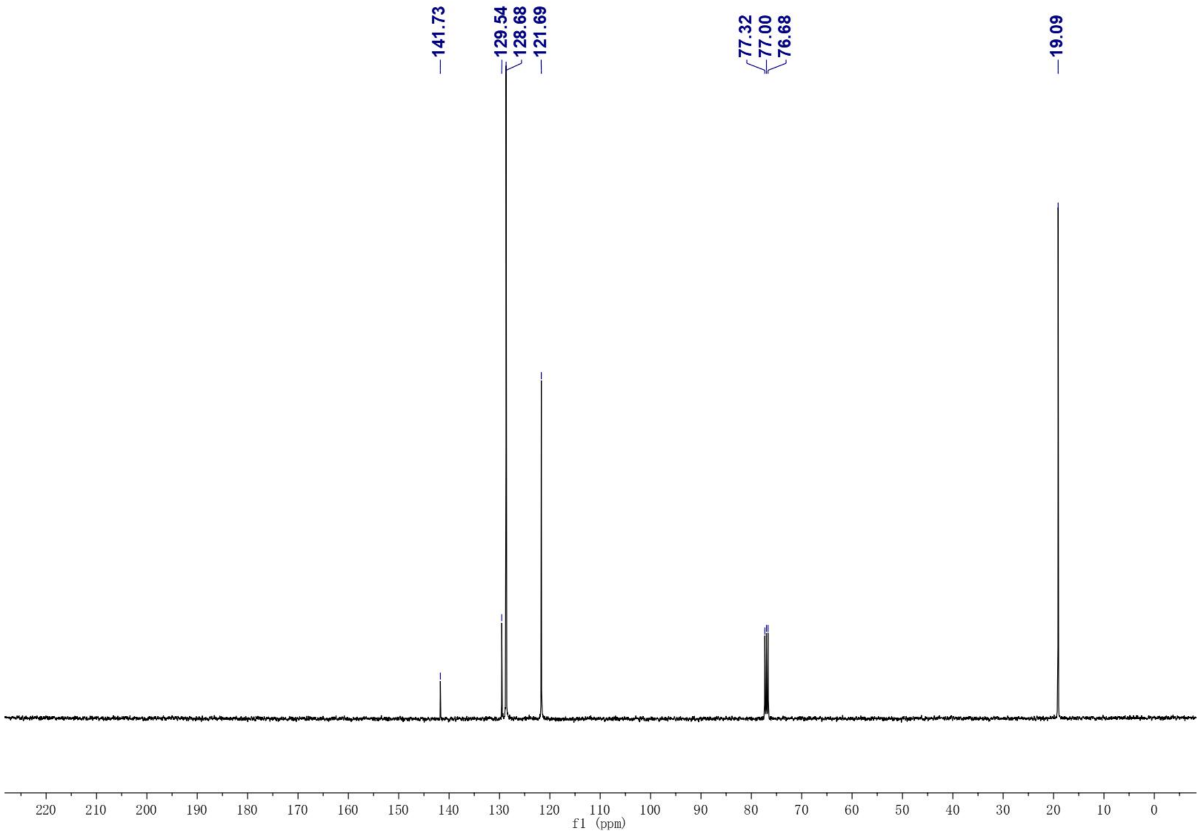

Supplementary Figure 27. ^{13}C NMR Spectrum of **2m**

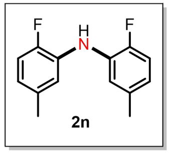

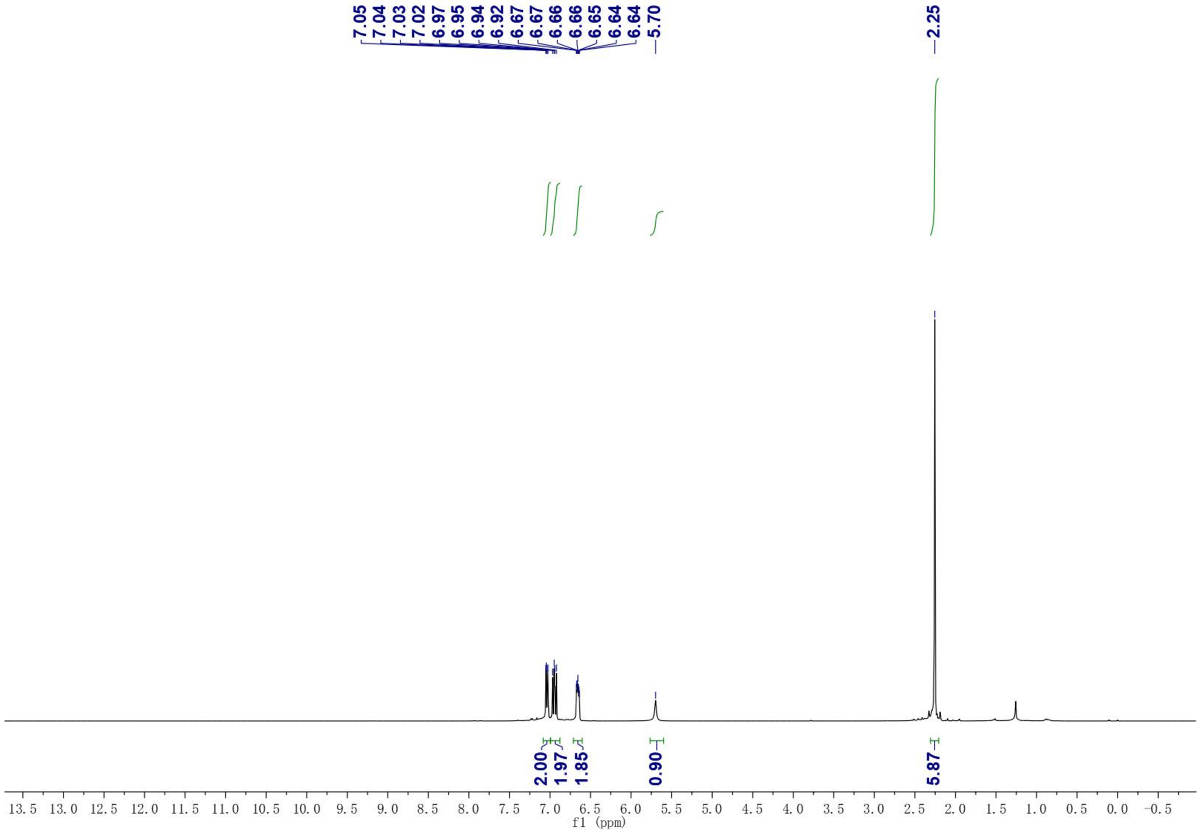

Supplementary Figure 28. ^{1}H NMR Spectrum of **2n**

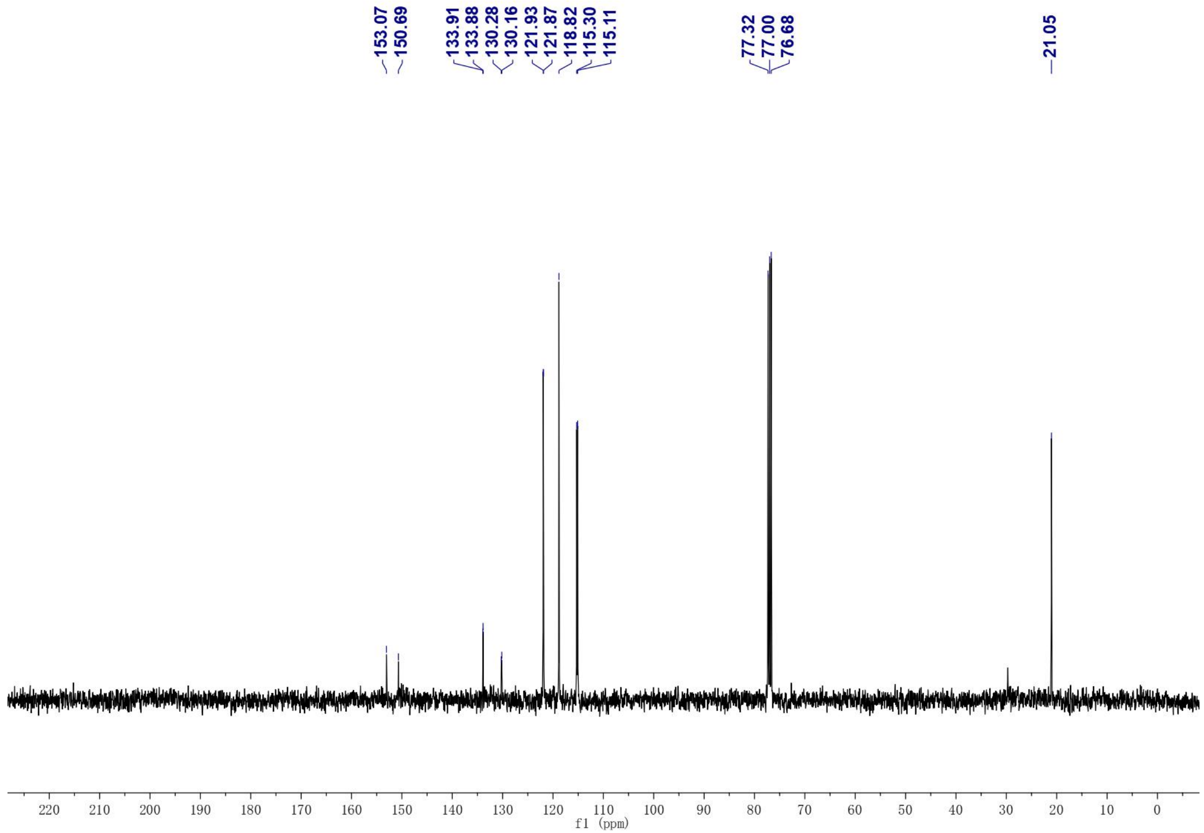

Supplementary Figure 29. ^{13}C NMR Spectrum of **2n**

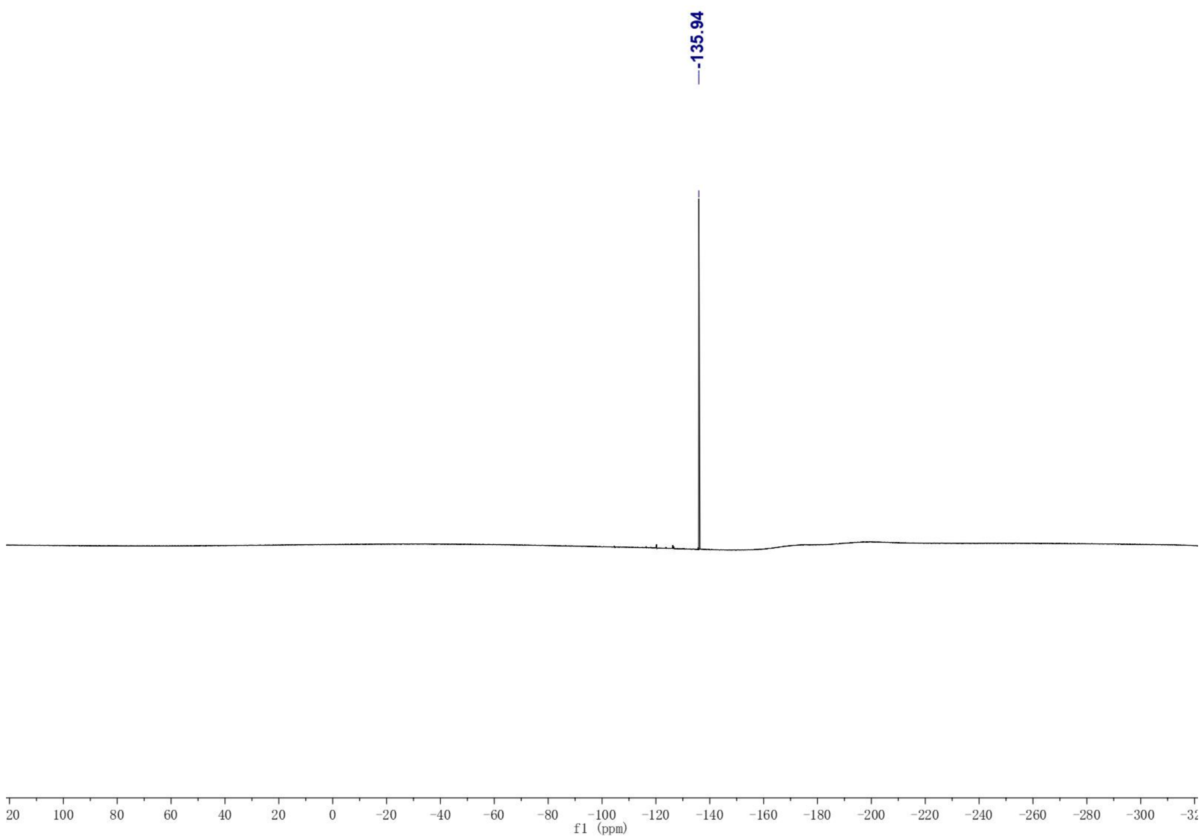

Supplementary Figure 30. ^{19}F NMR Spectrum of **2n**

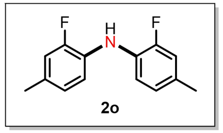

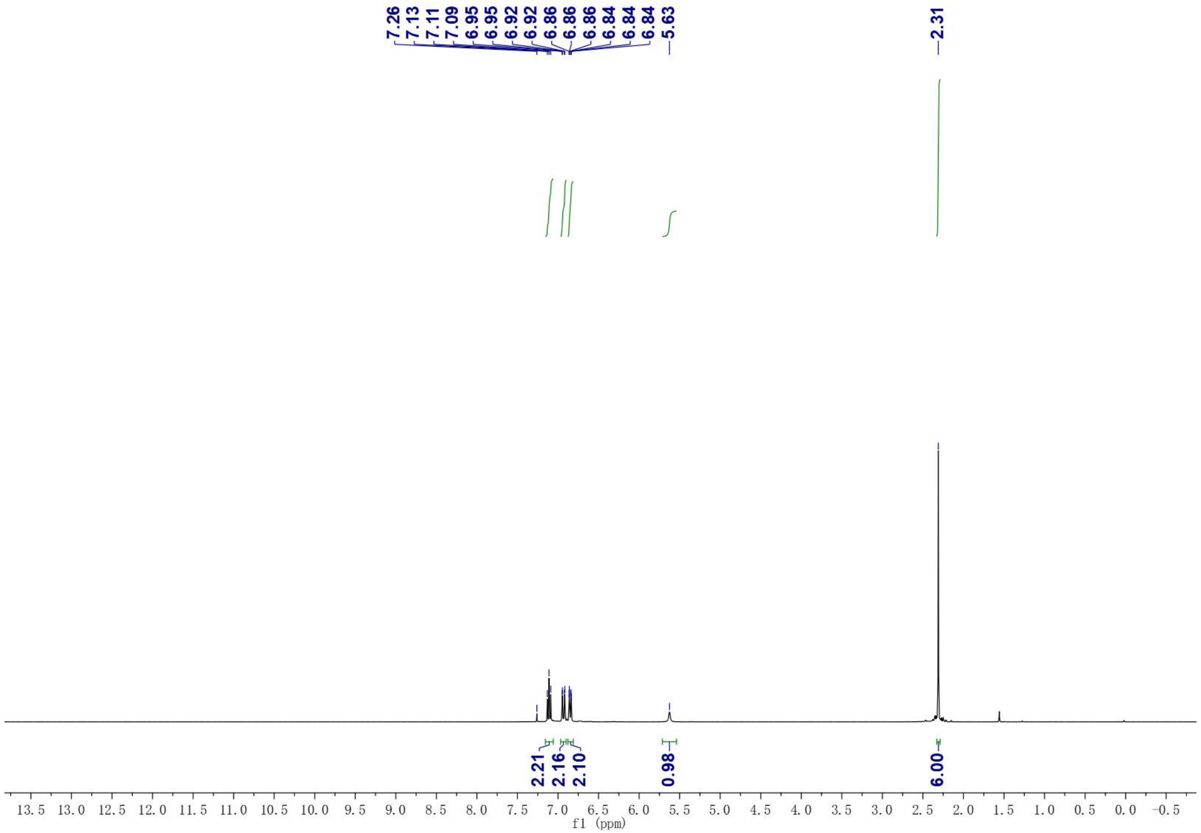

Supplementary Figure 31. ^{1}H NMR Spectrum of **2o**

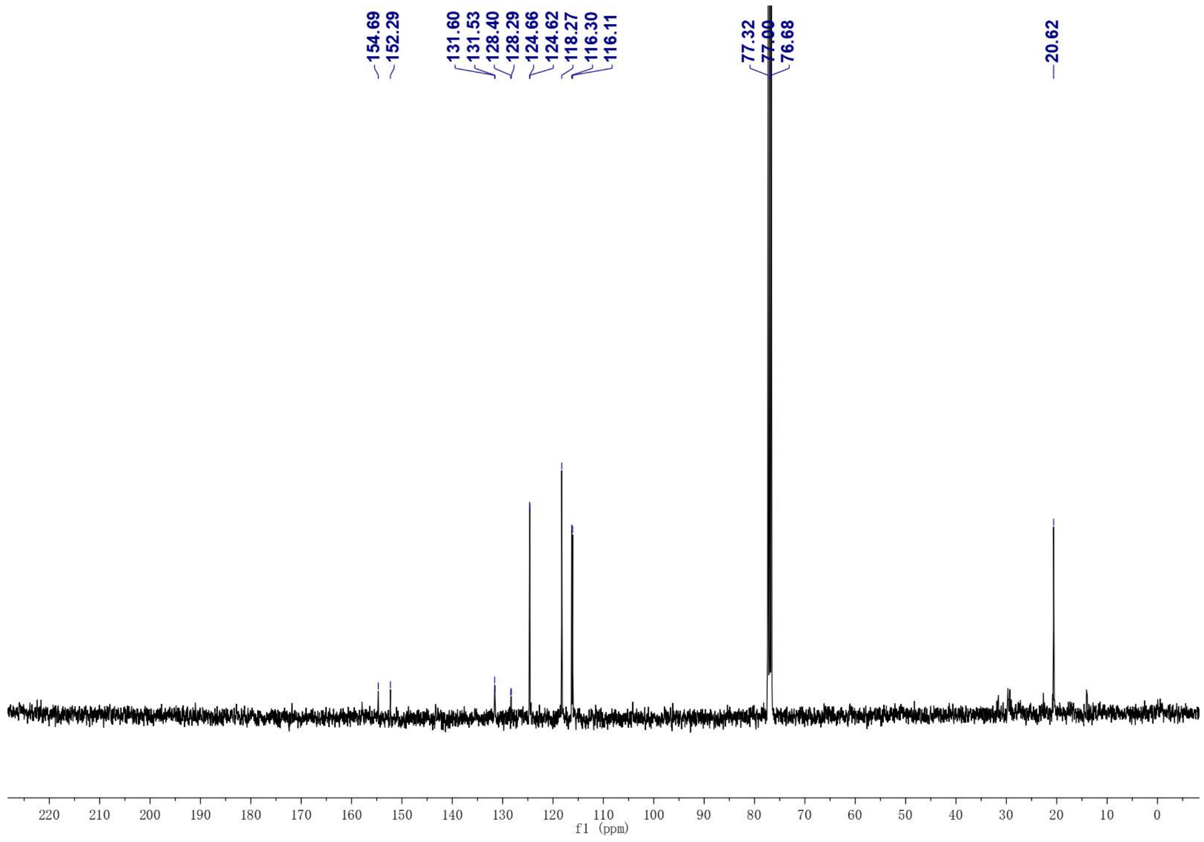

Supplementary Figure 32. ^{13}C NMR Spectrum of **2o**

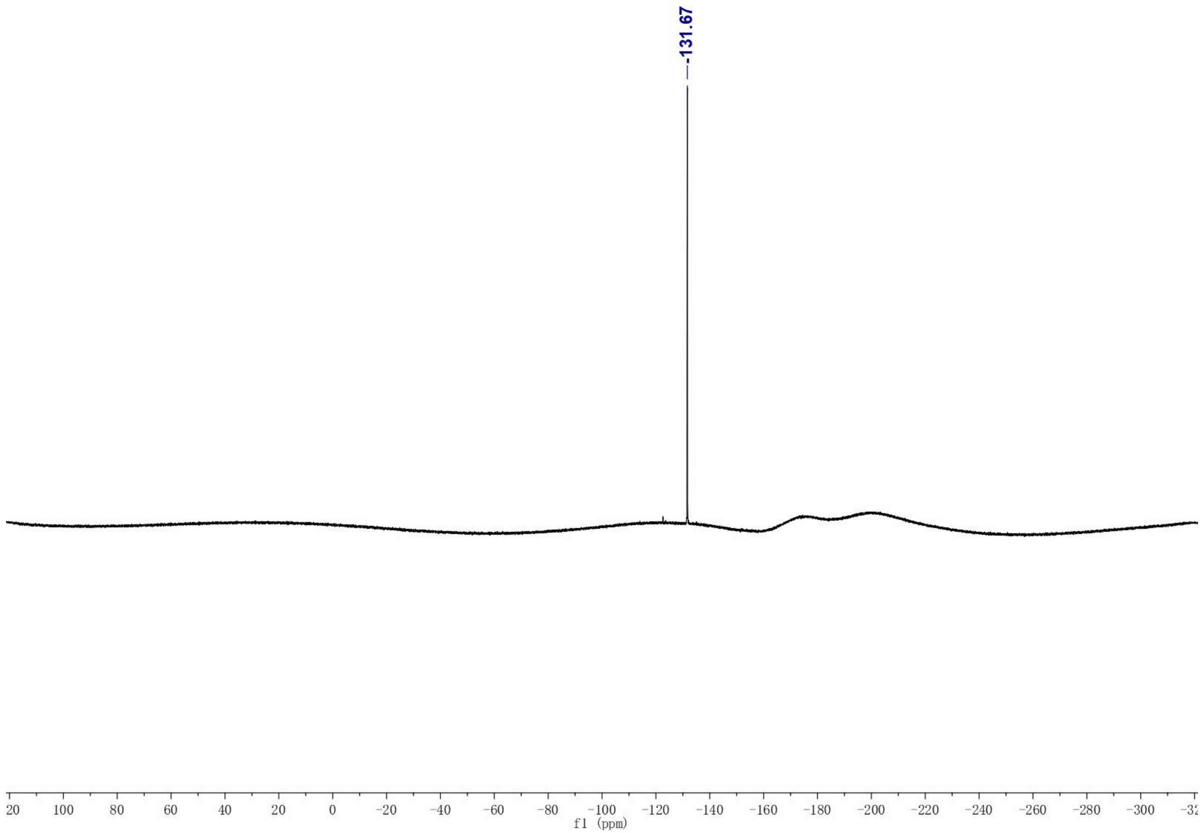

Supplementary Figure 33. ^{19}F NMR Spectrum of **2o**

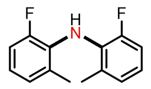

2p

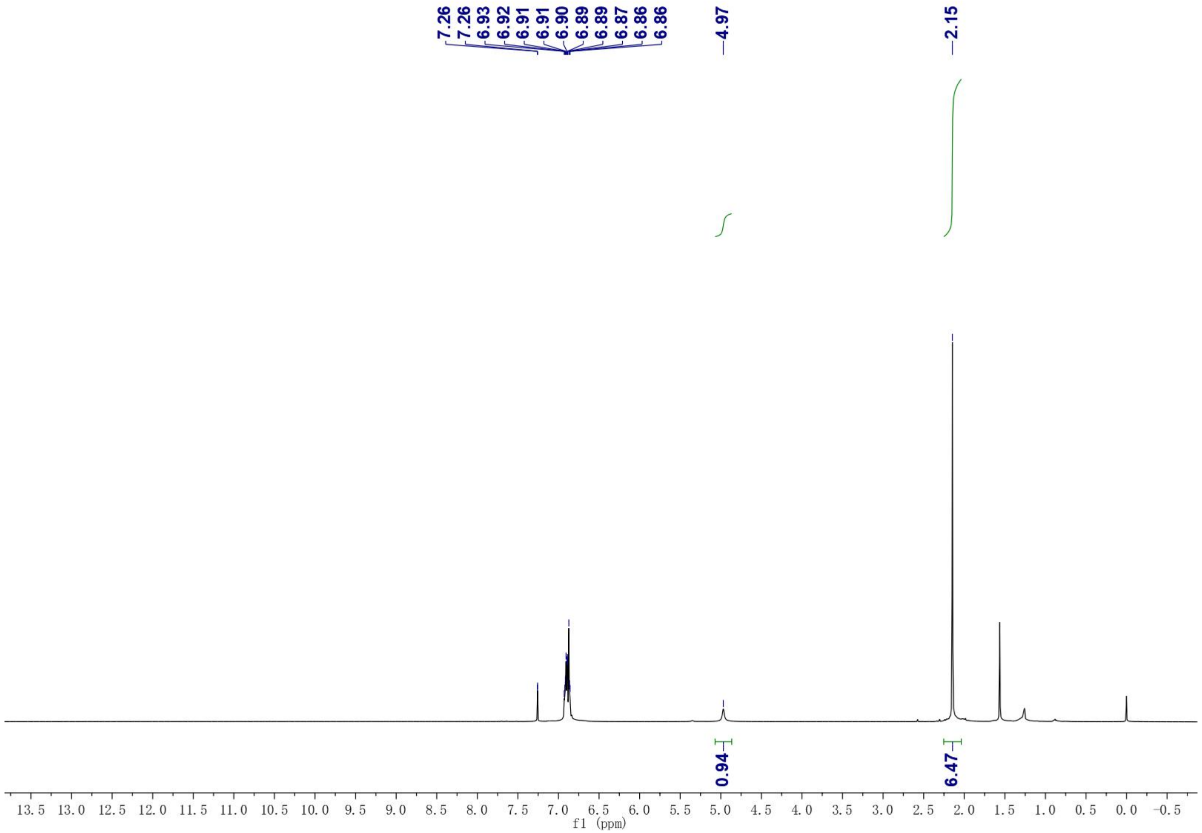

Supplementary Figure 34. ^{1}H NMR Spectrum of **2p**

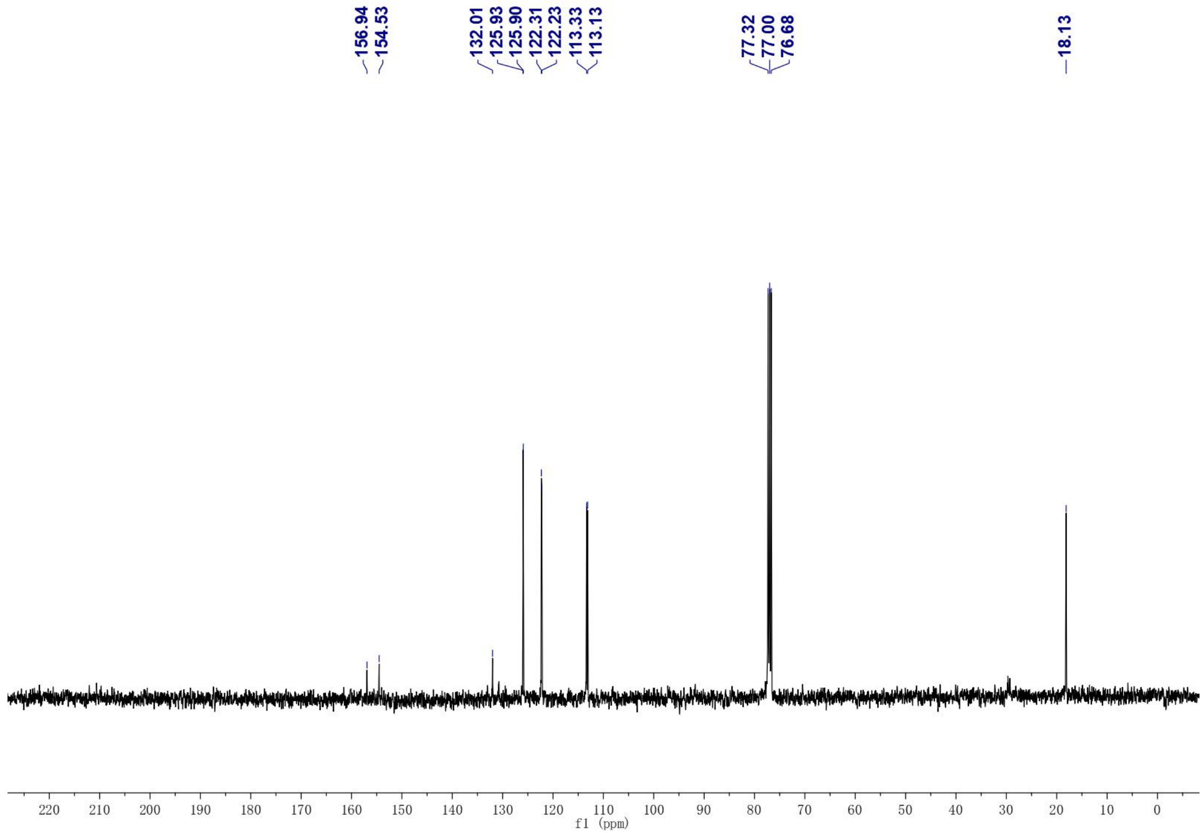

Supplementary Figure 35. ^{13}C NMR Spectrum of **2p**

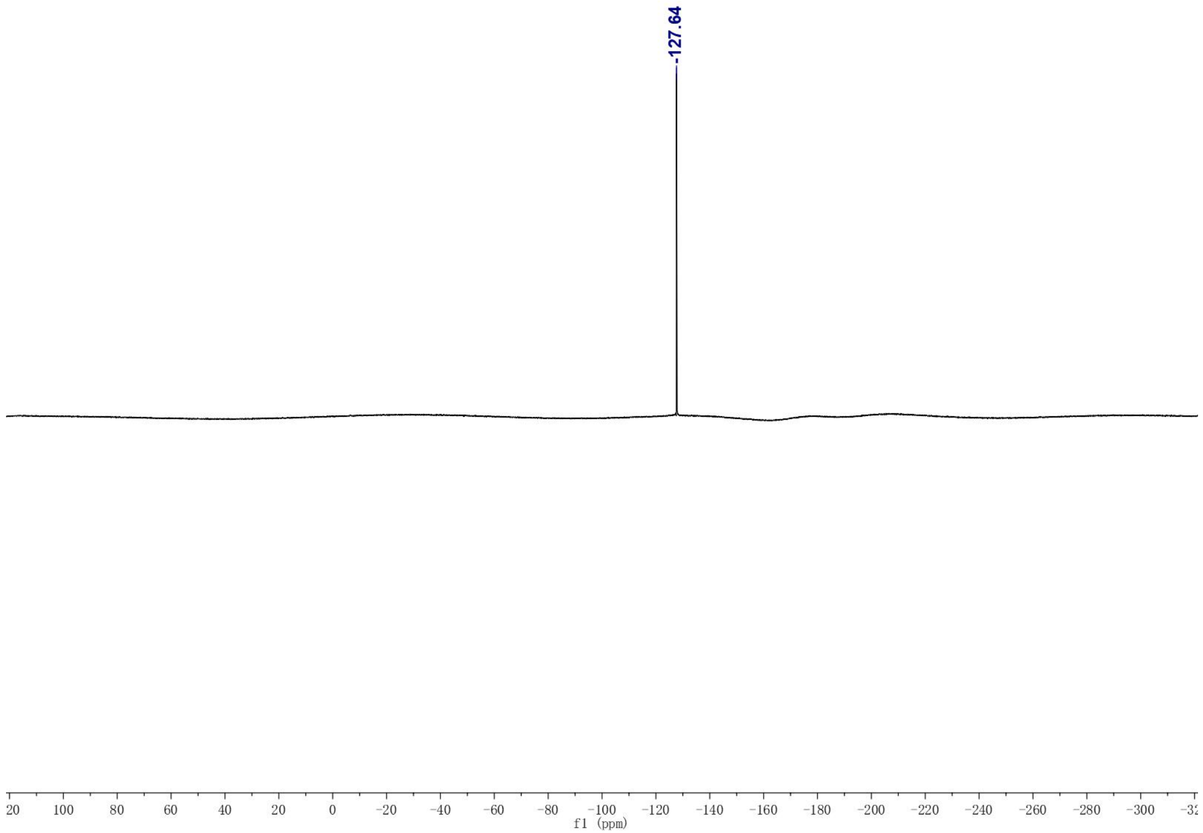

Supplementary Figure 36. ^{19}F NMR Spectrum of **2p**

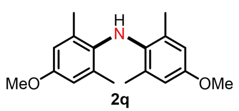

2q

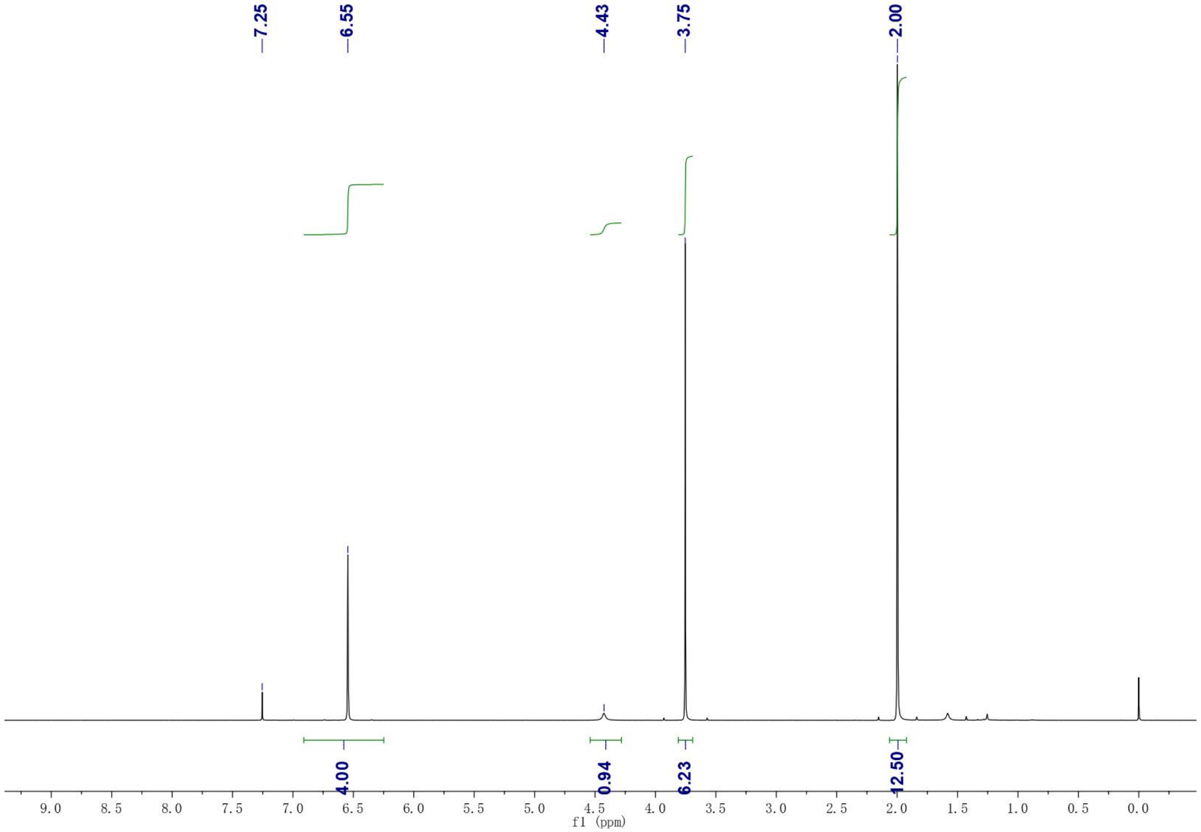

Supplementary Figure 37. ^{1}H NMR Spectrum of **2q**

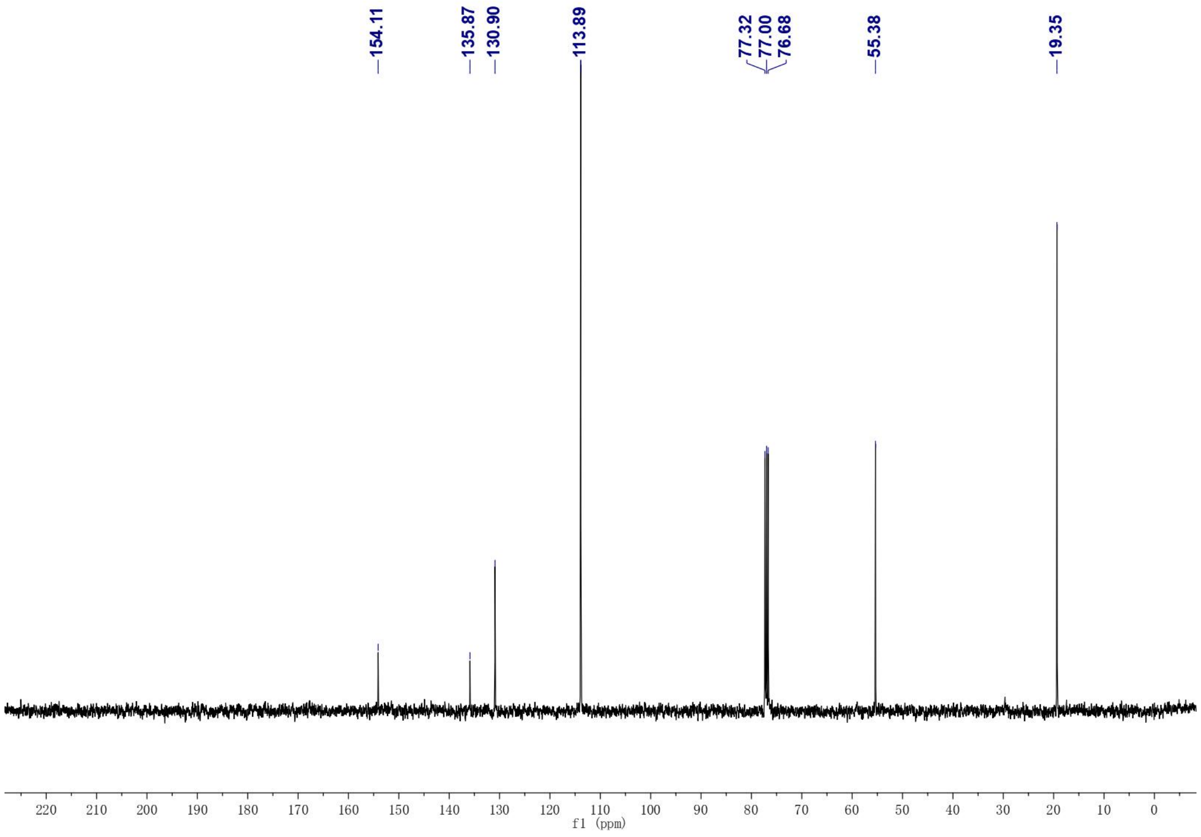

Supplementary Figure 38. ^{13}C NMR Spectrum of **2q**

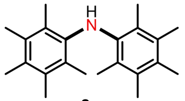

2r

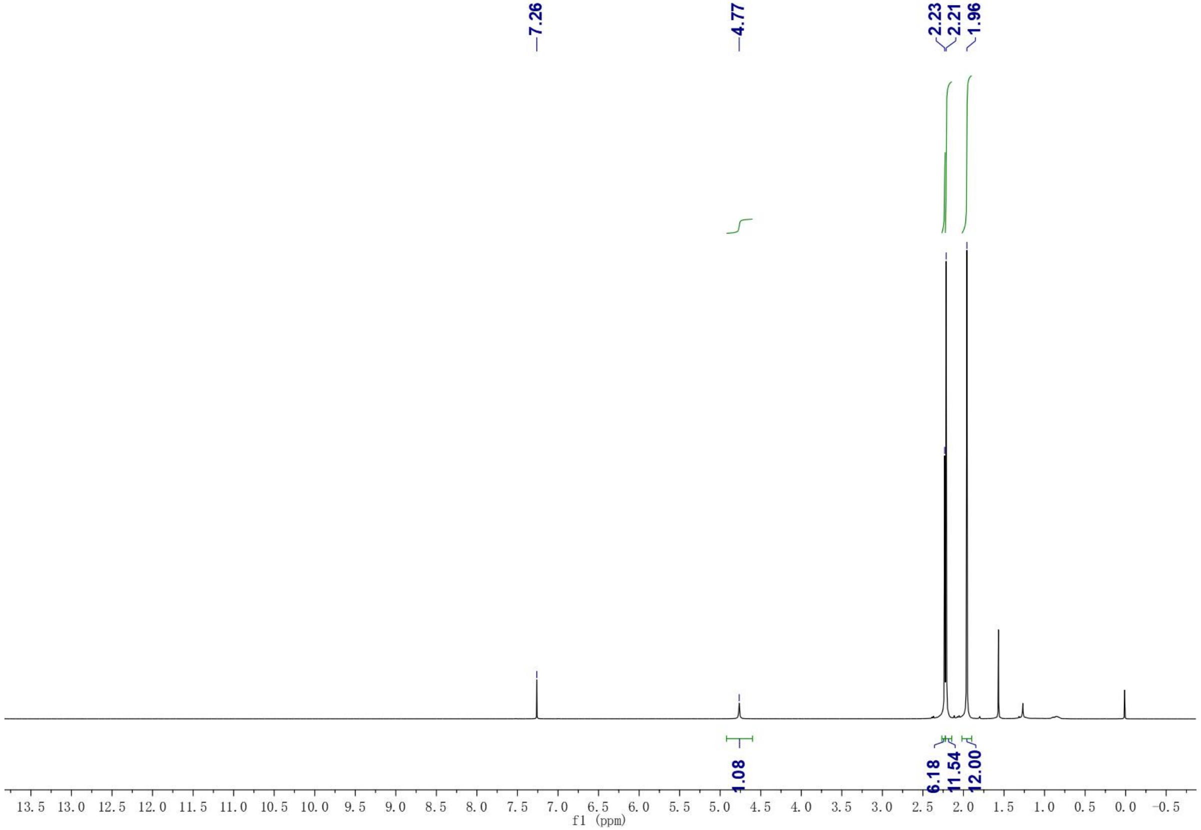

Supplementary Figure 39. ^{1}H NMR Spectrum of **2r**

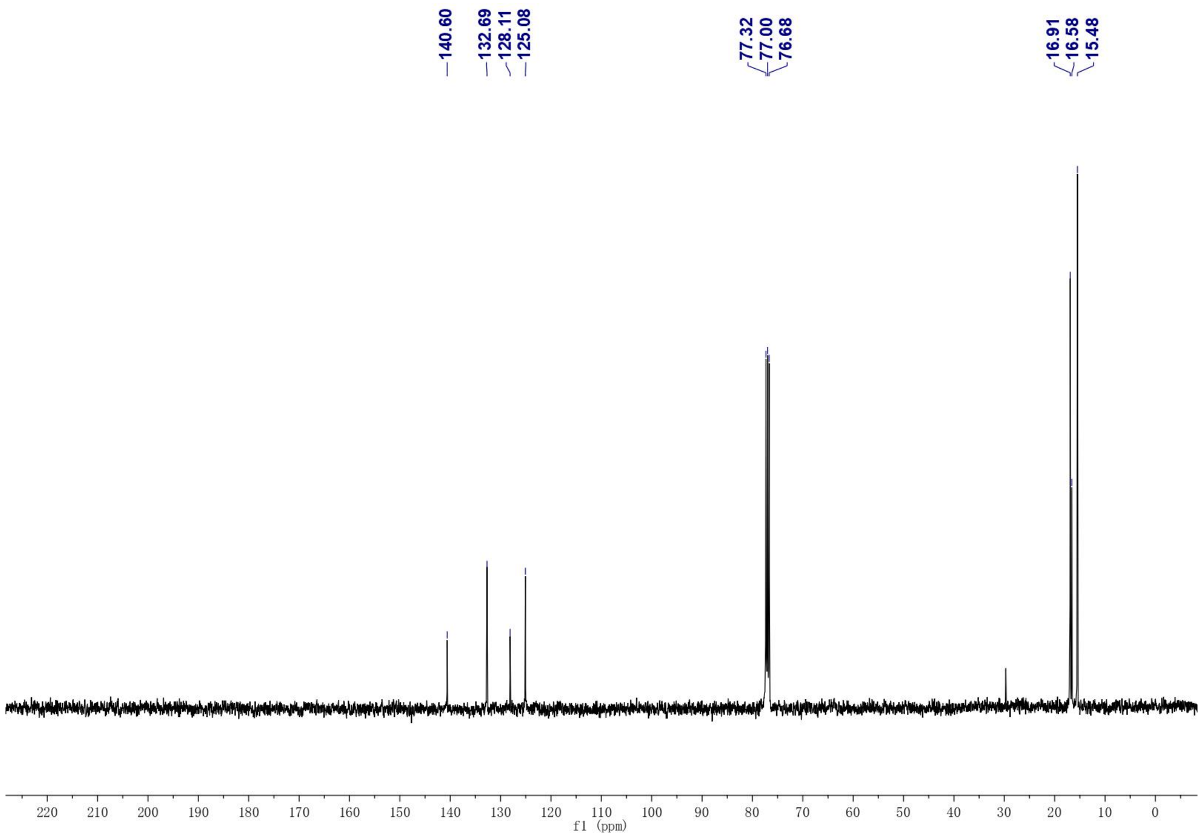

Supplementary Figure 40. ^{13}C NMR Spectrum of **2r**

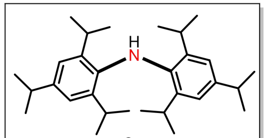

2s

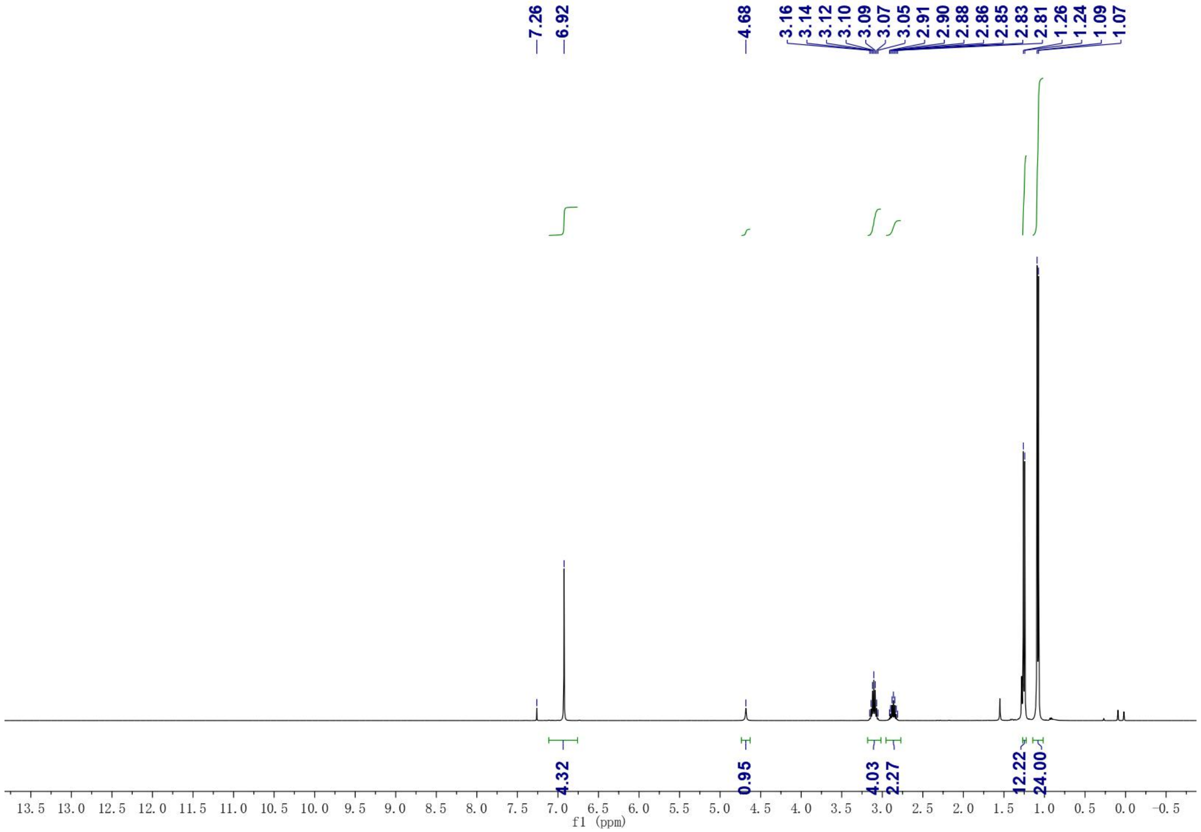

Supplementary Figure 41. ^{1}H NMR Spectrum of **2s**

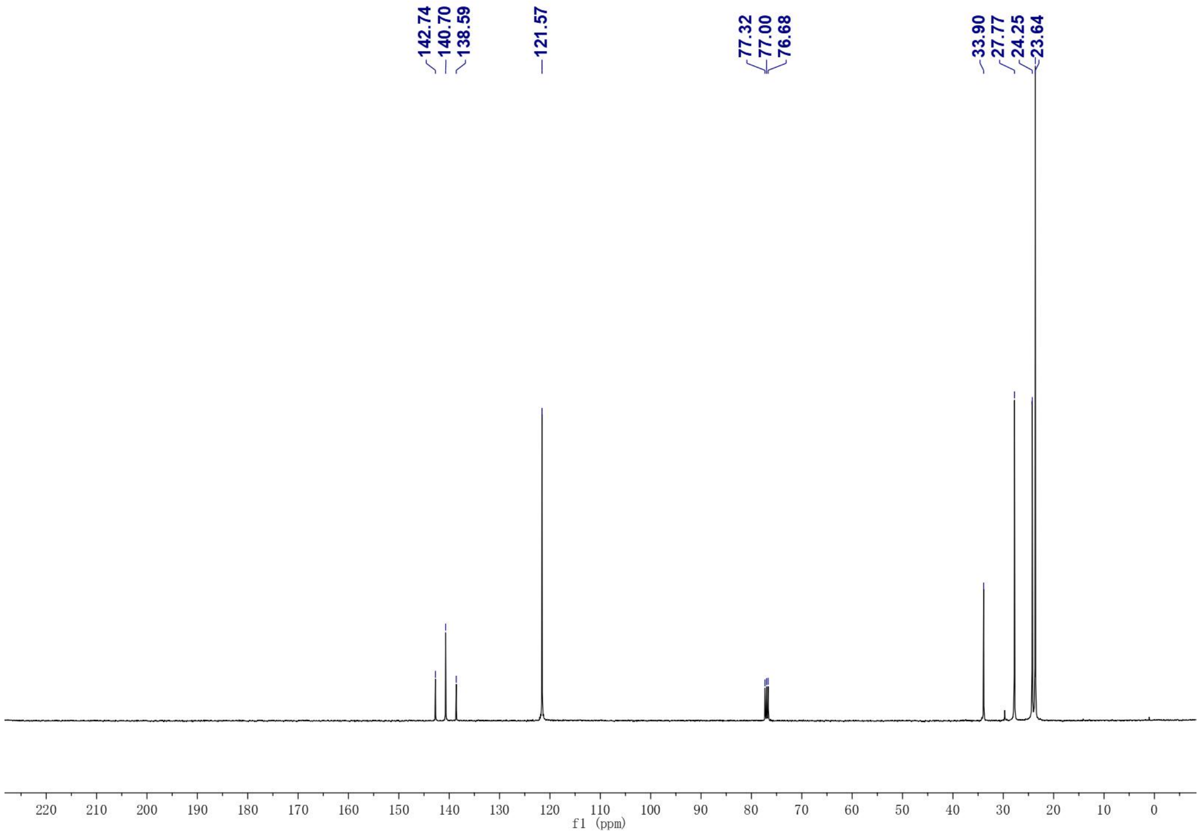

Supplementary Figure 42. ^{13}C NMR Spectrum of **2s**

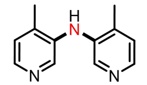

2t

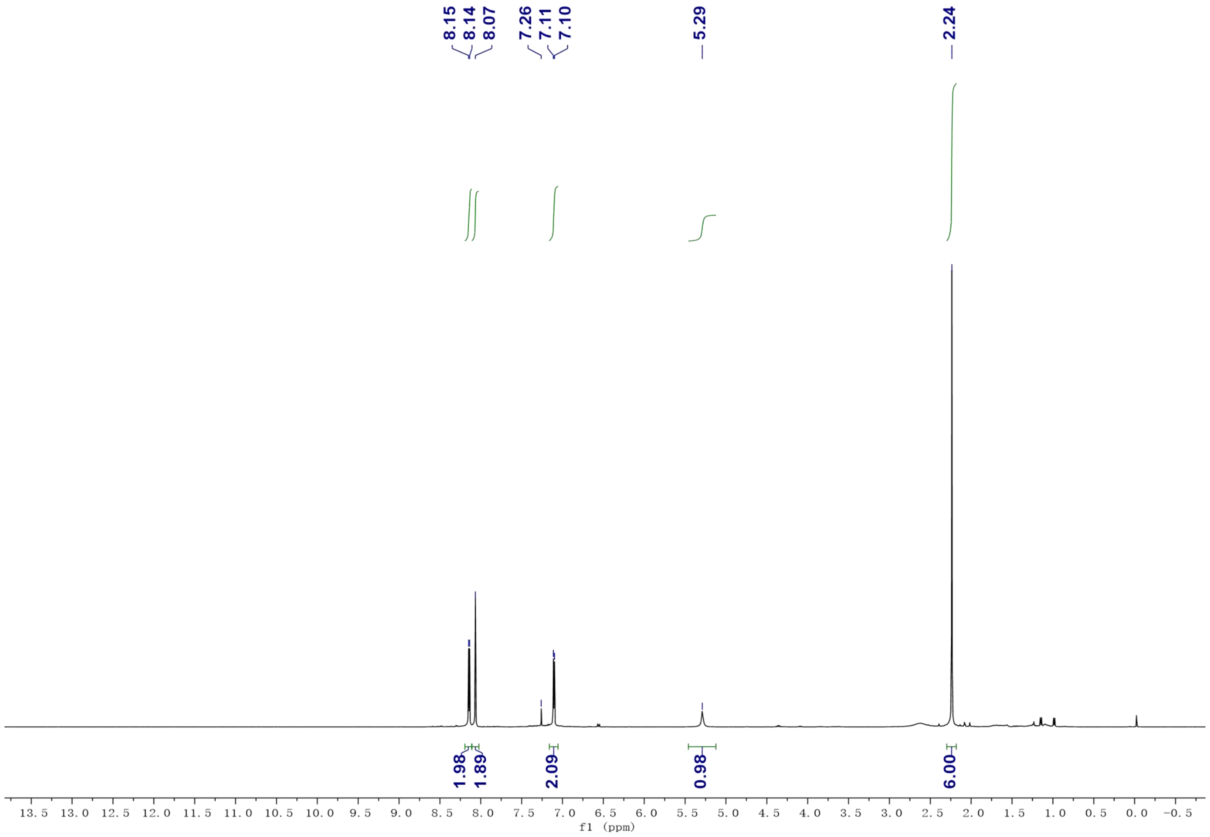

Supplementary Figure 43. ^{1}H NMR Spectrum of **2t**

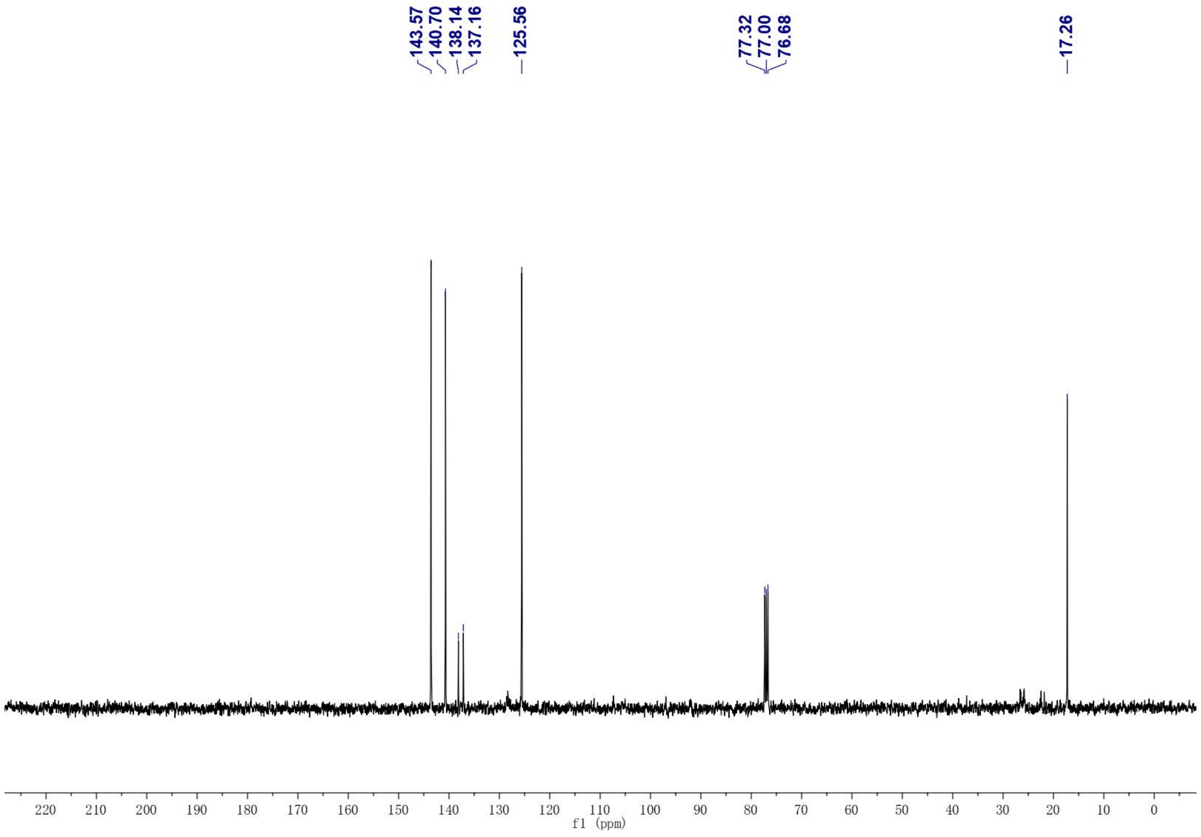

Supplementary Figure 44. ^{13}C NMR Spectrum of **2t**

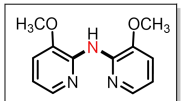

2u

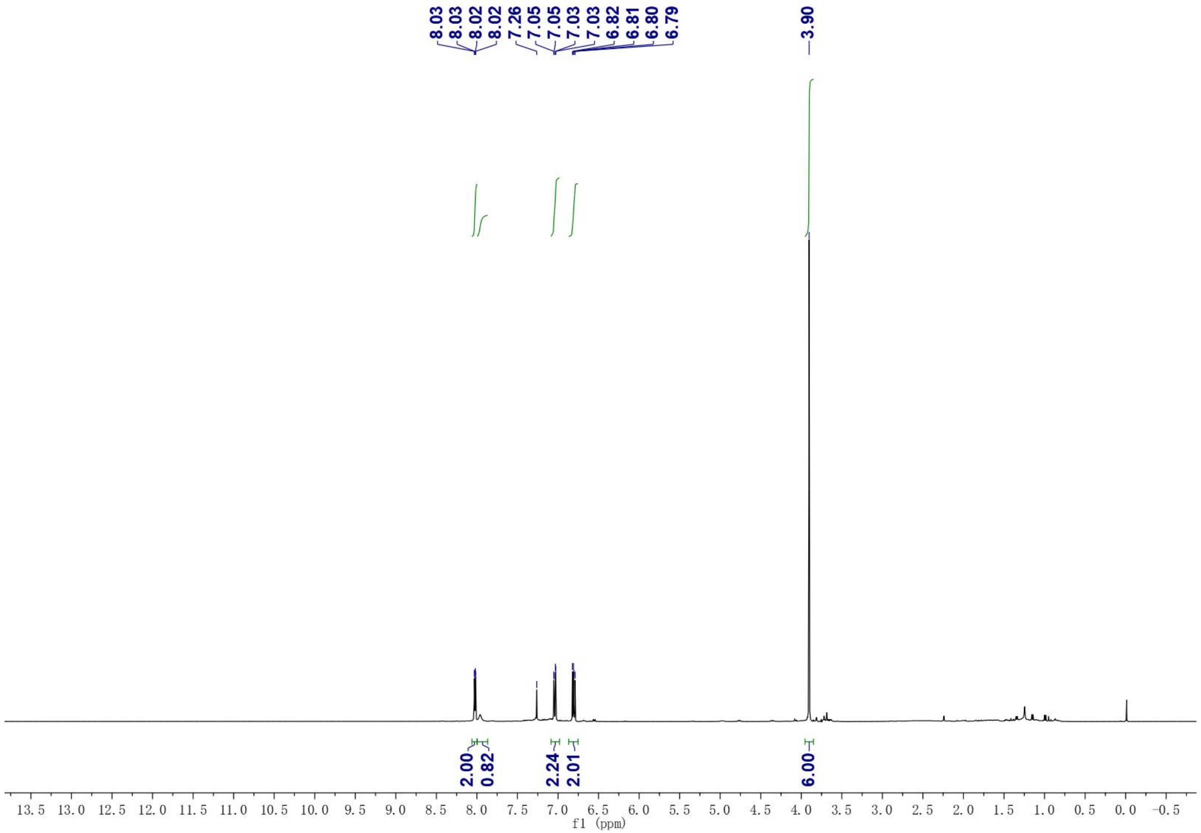

Supplementary Figure 45. ^{1}H NMR Spectrum of **2u**

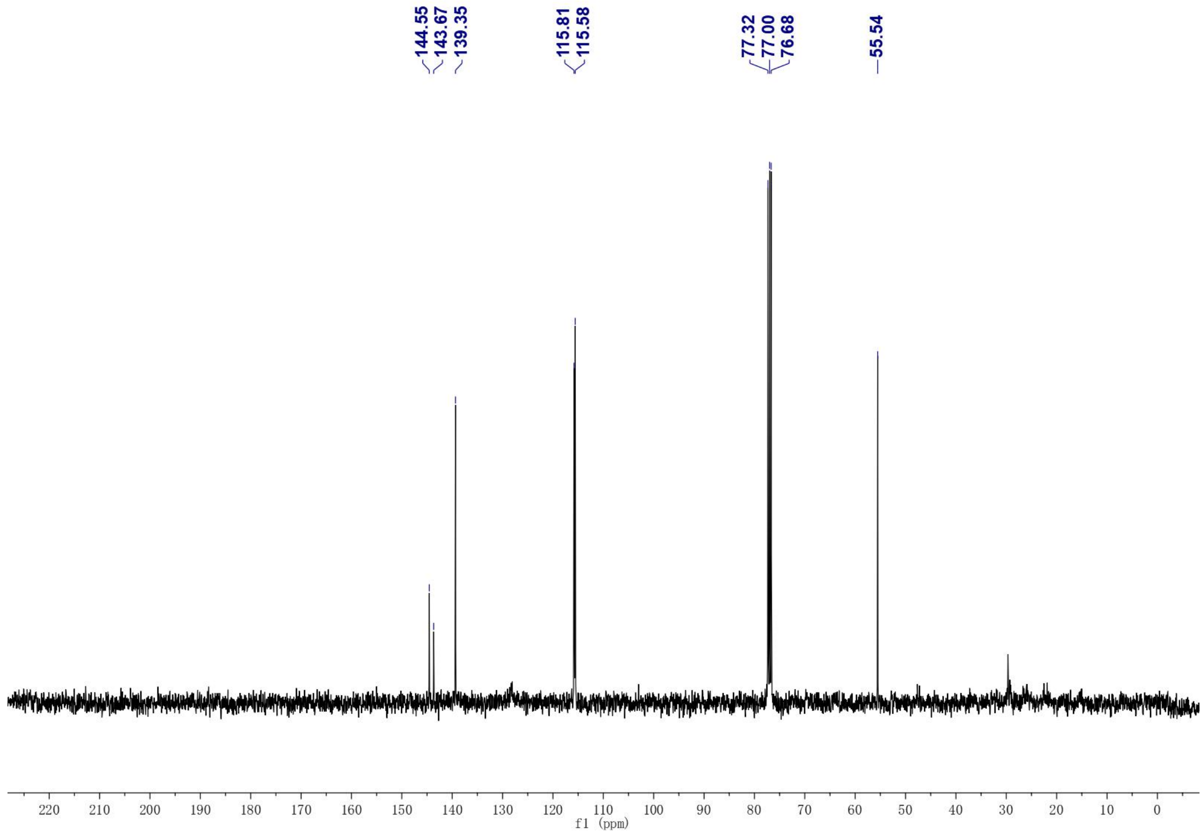

Supplementary Figure 46. ^{13}C NMR Spectrum of **2u**

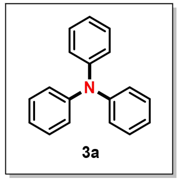

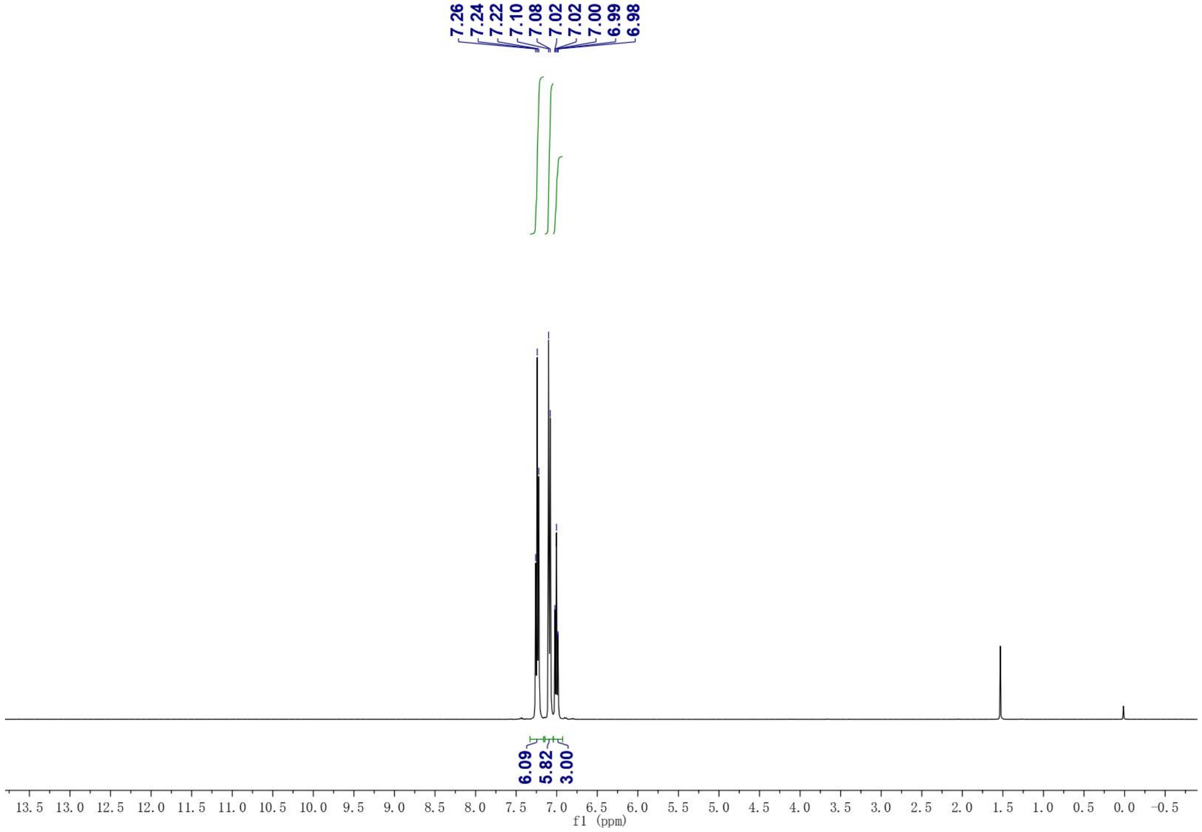

Supplementary Figure 47. ^{1}H NMR Spectrum of **3a**

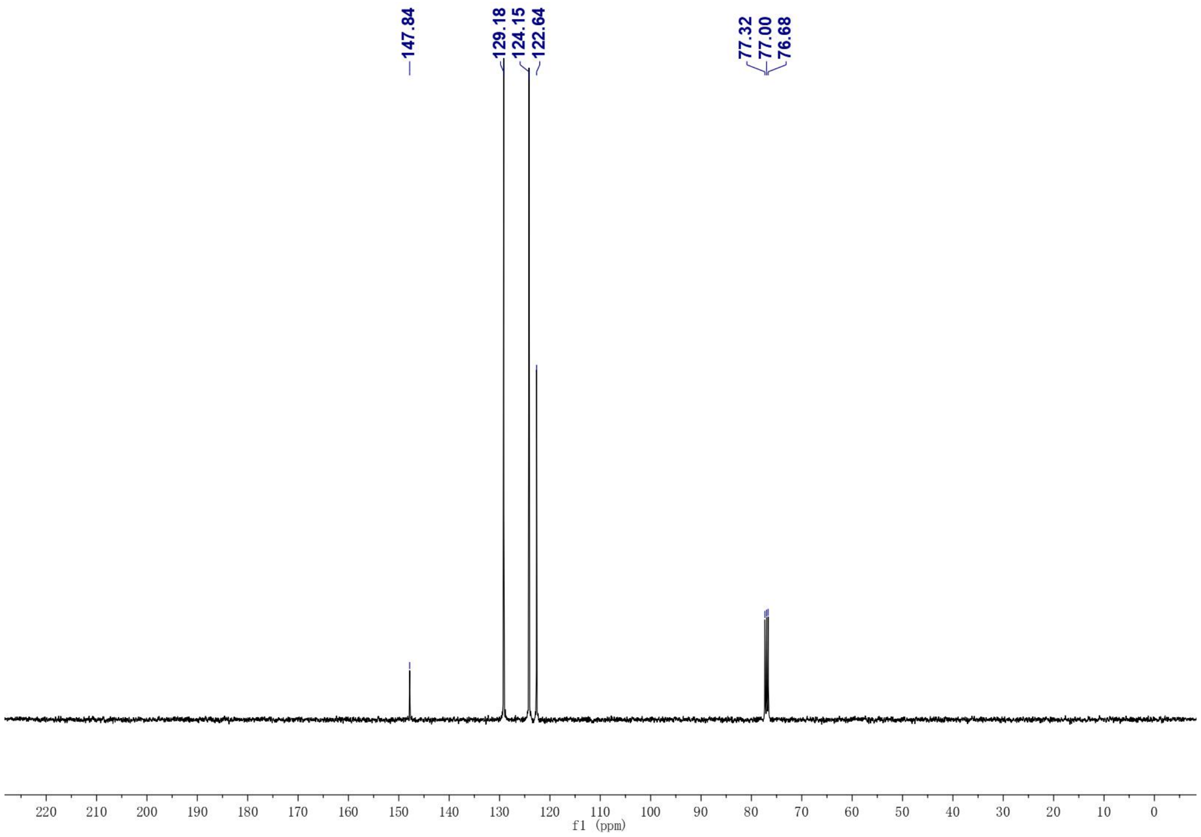

Supplementary Figure 48. ^{13}C NMR Spectrum of **3a**

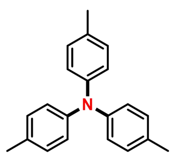

3b

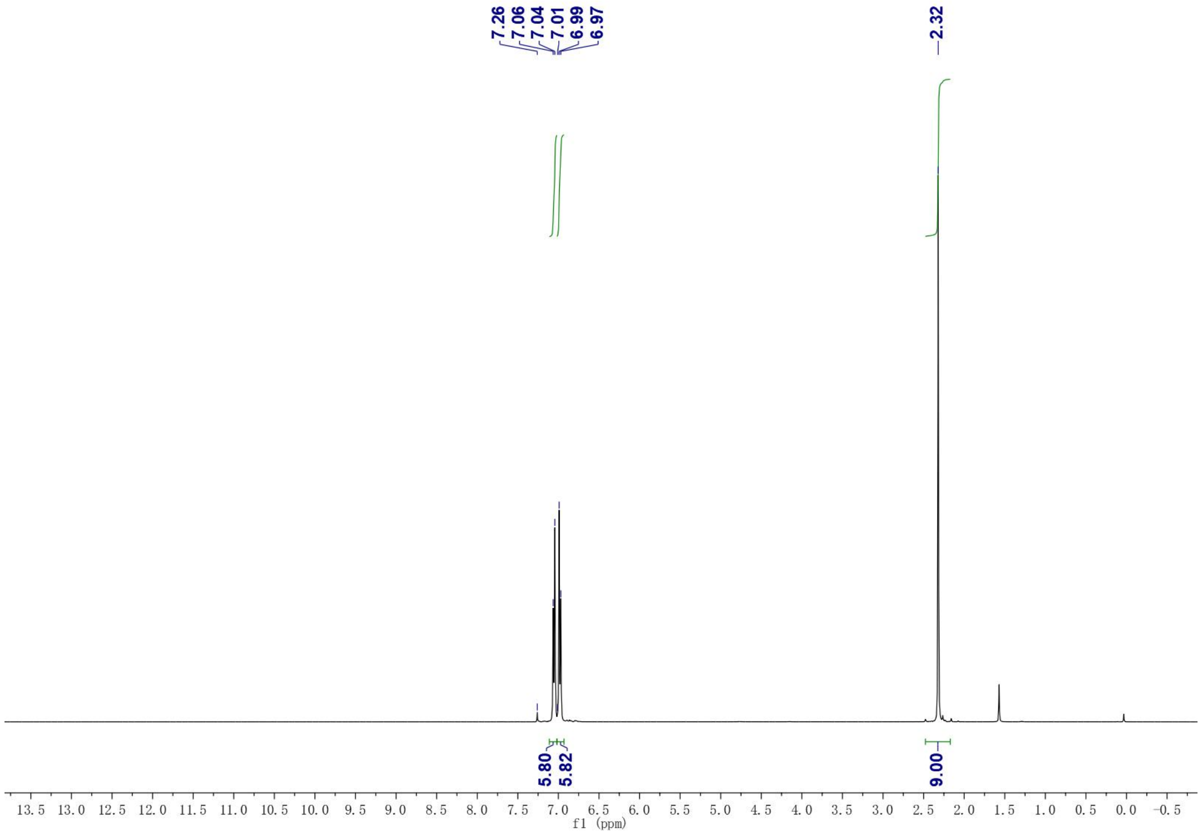

Supplementary Figure 49. ^{1}H NMR Spectrum of **3b**

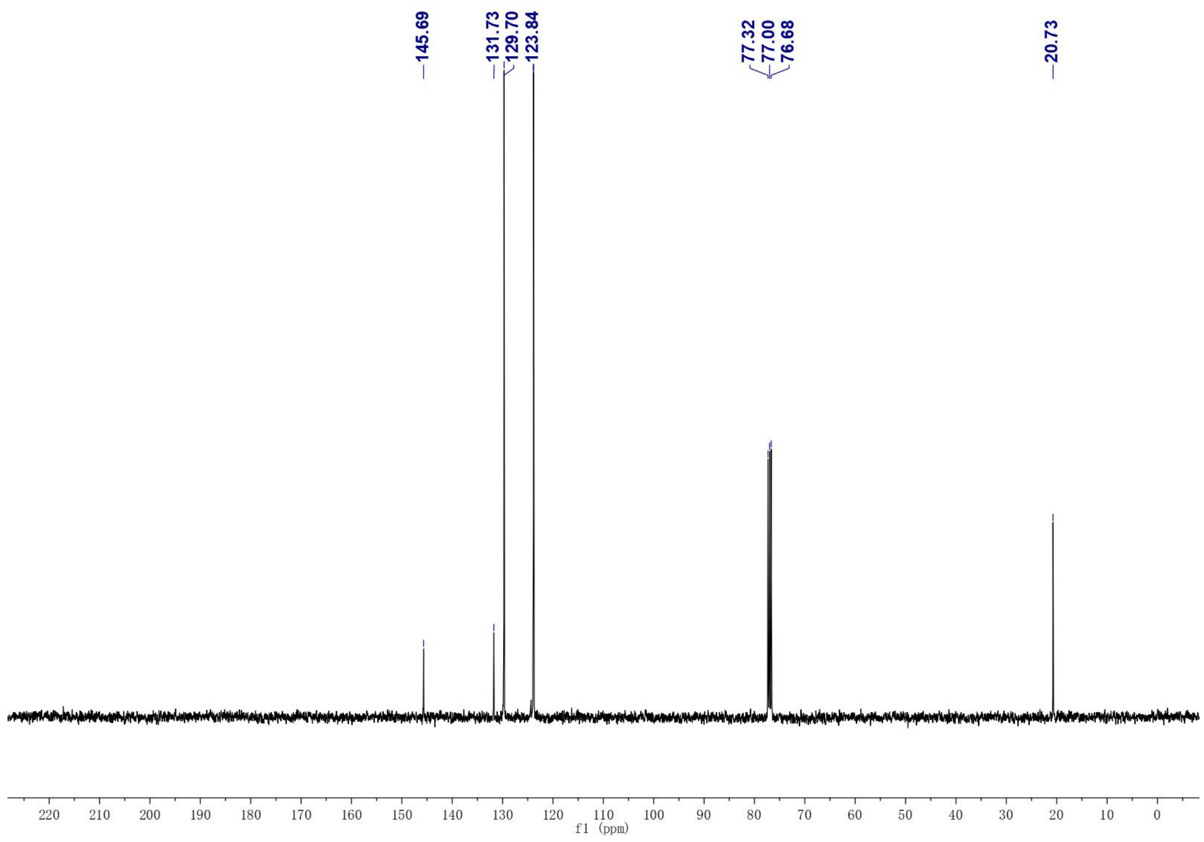

Supplementary Figure 50. ^{13}C NMR Spectrum of **3b**

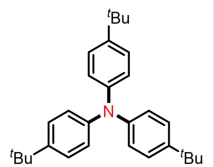

3c

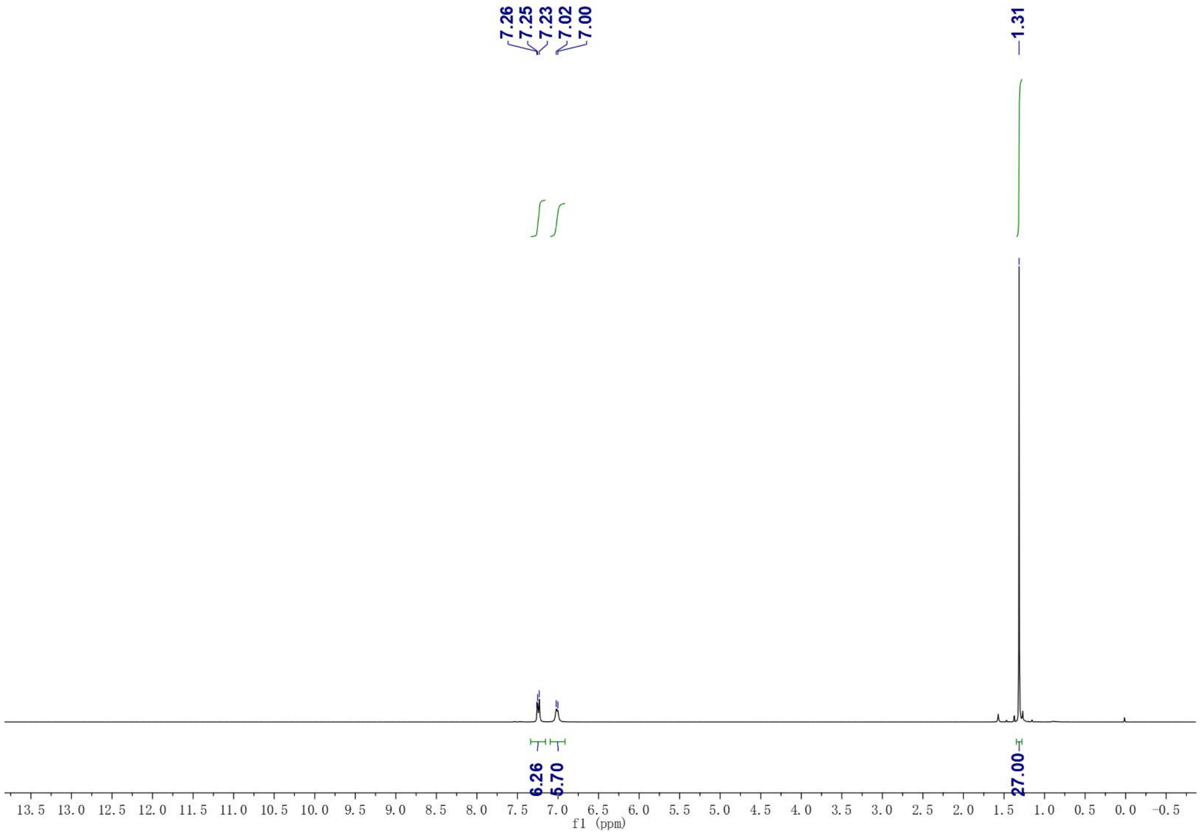

Supplementary Figure 51. ^{1}H NMR Spectrum of **3c**

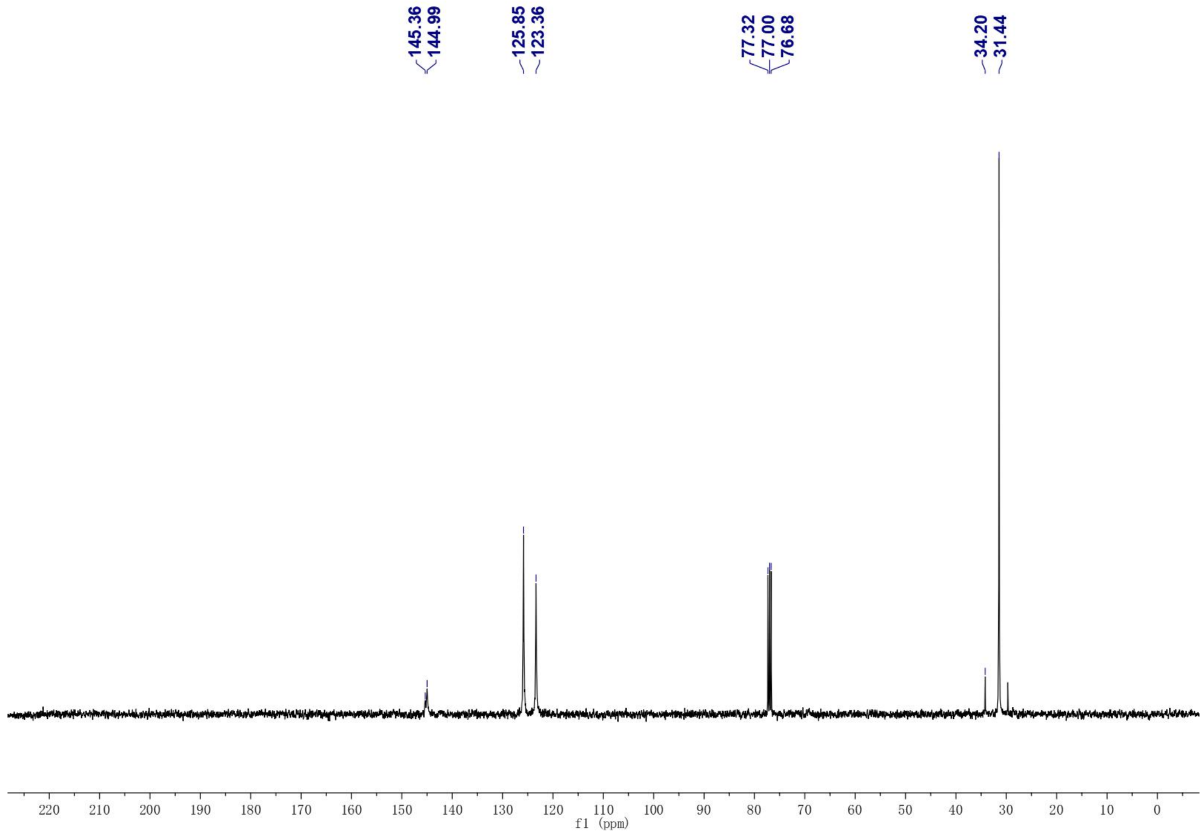

Supplementary Figure 52. ^{13}C NMR Spectrum of **3c**

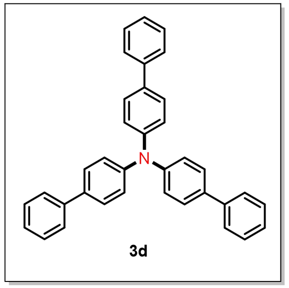

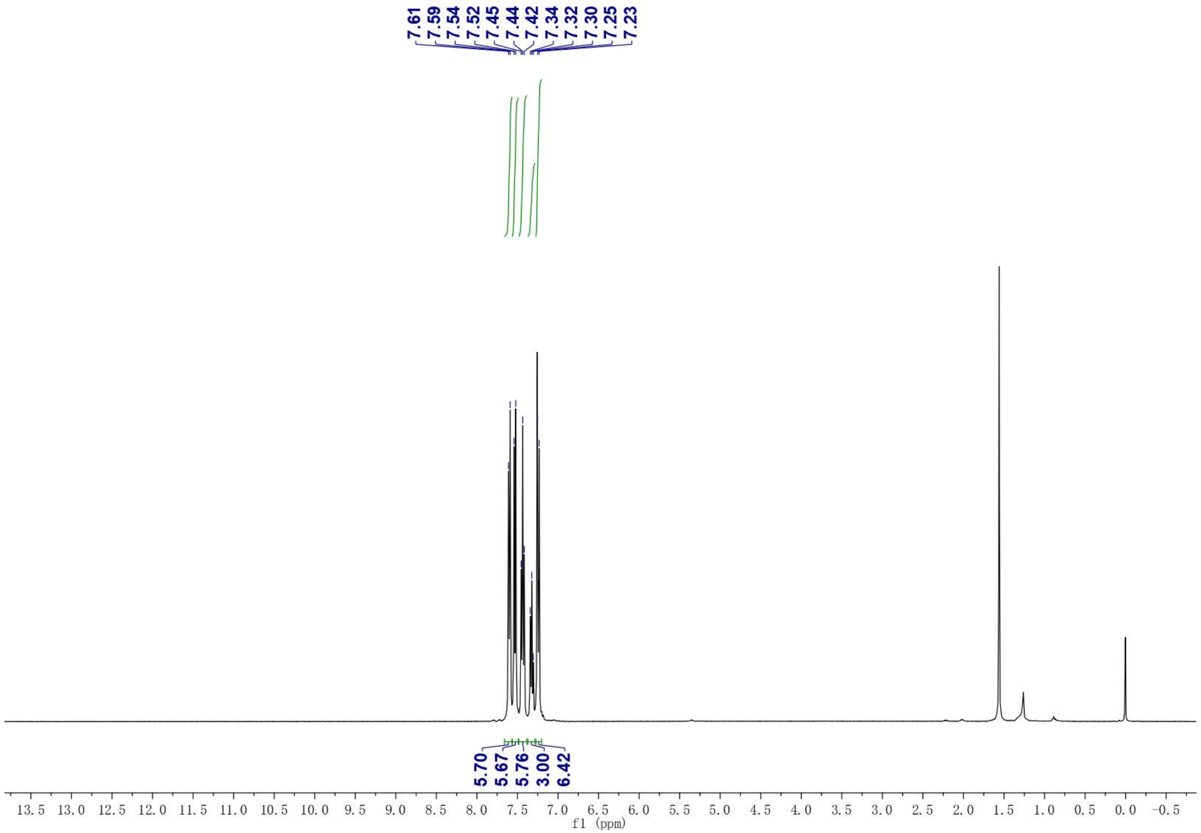

Supplementary Figure 53. ^{1}H NMR Spectrum of **3d**

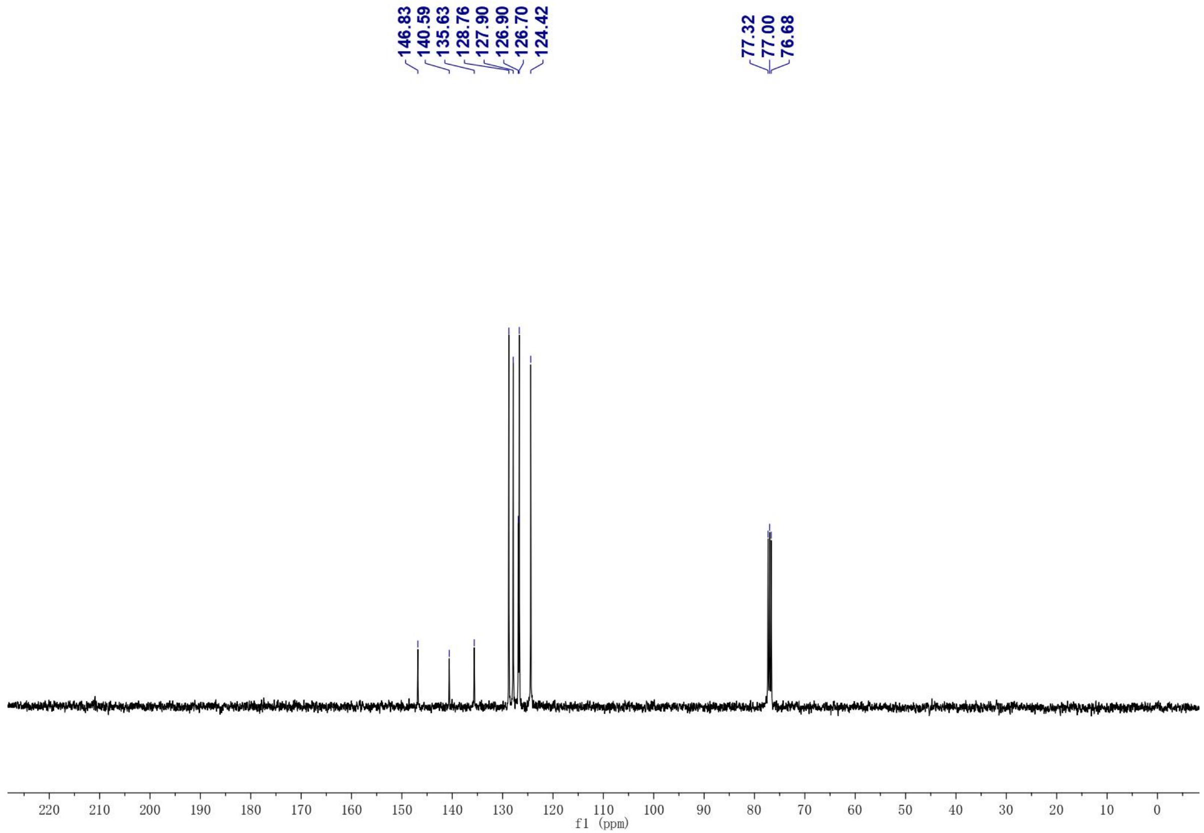

Supplementary Figure 54. ^{13}C NMR Spectrum of **3d**

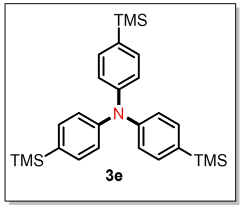

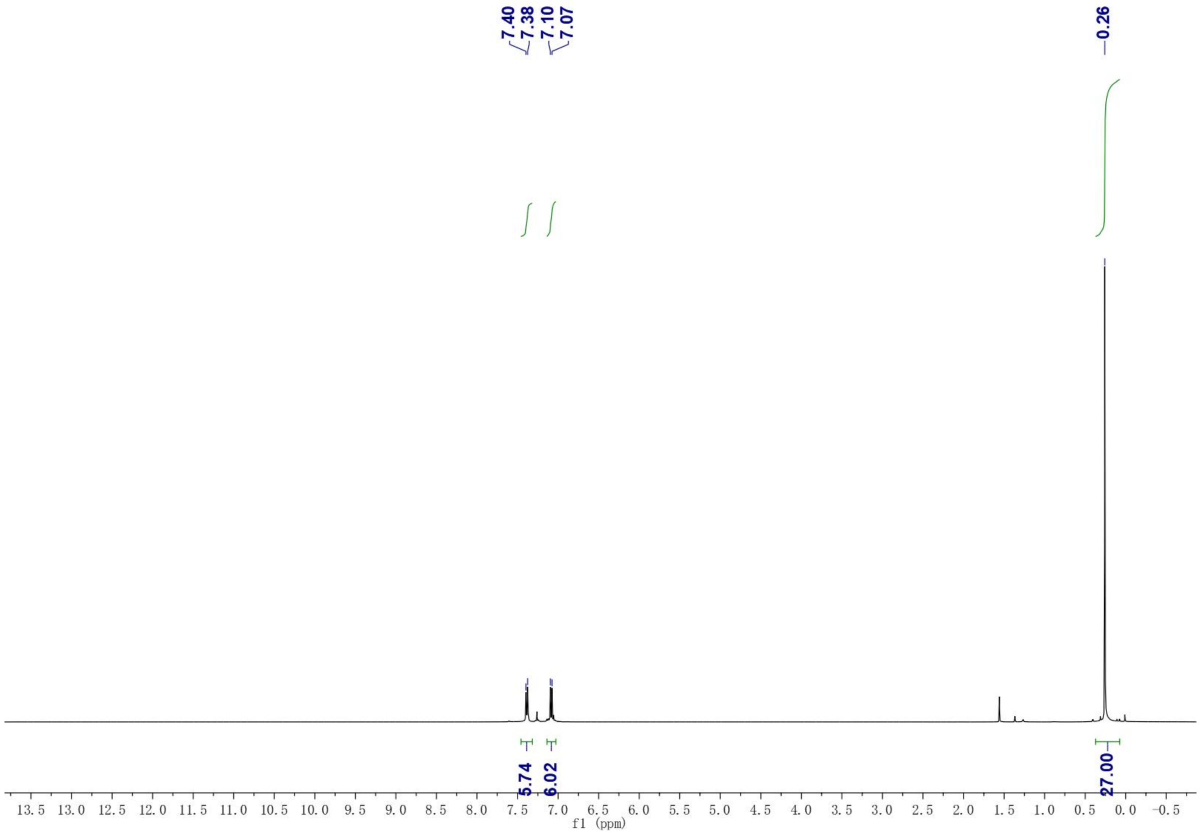

Supplementary Figure 55. ^{1}H NMR Spectrum of **3e**

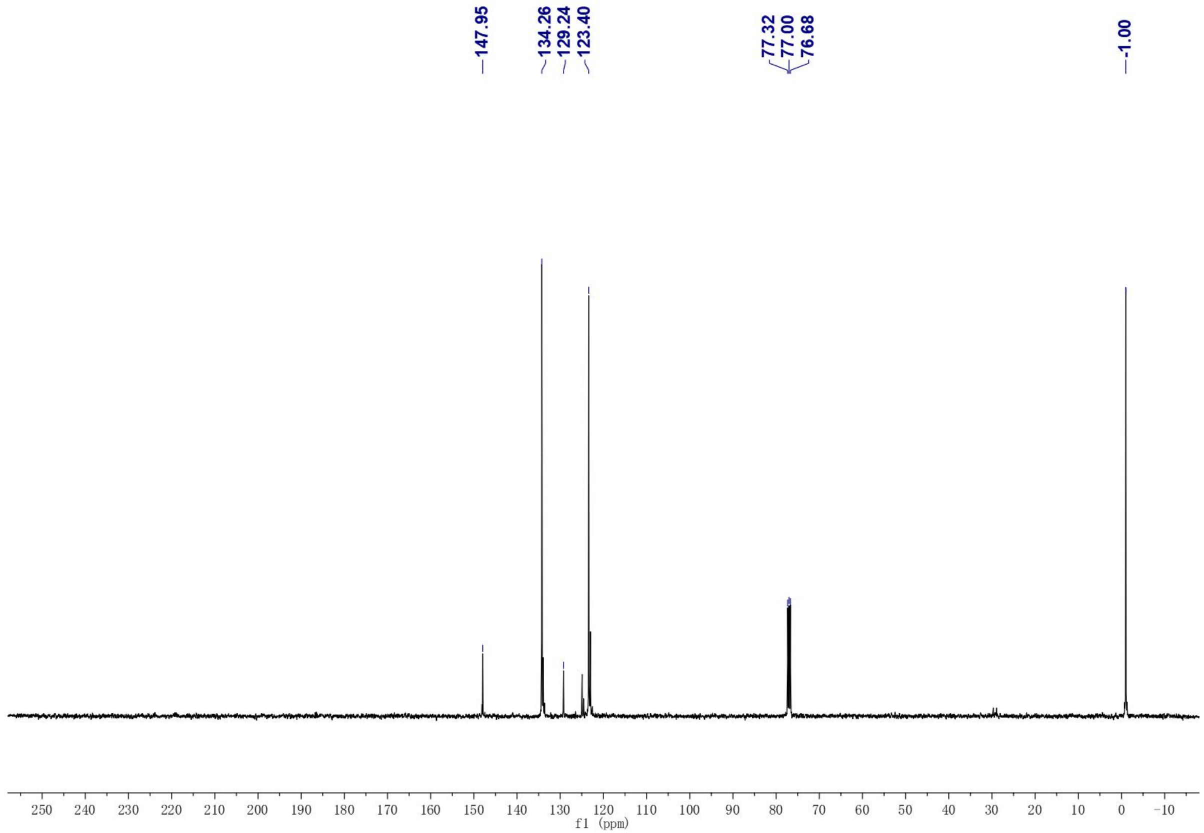

Supplementary Figure 56. ^{13}C NMR Spectrum of **3e**

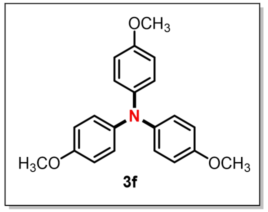

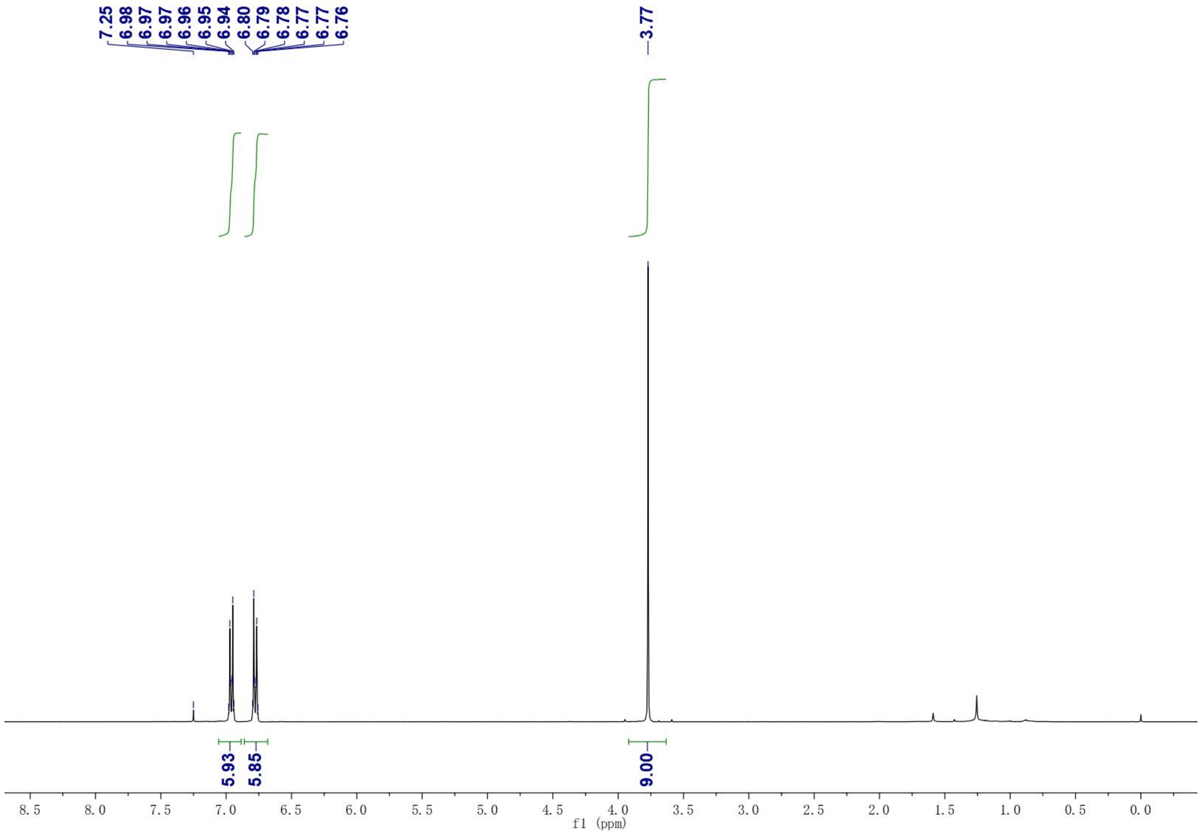

Supplementary Figure 57. ^{1}H NMR Spectrum of **3f**

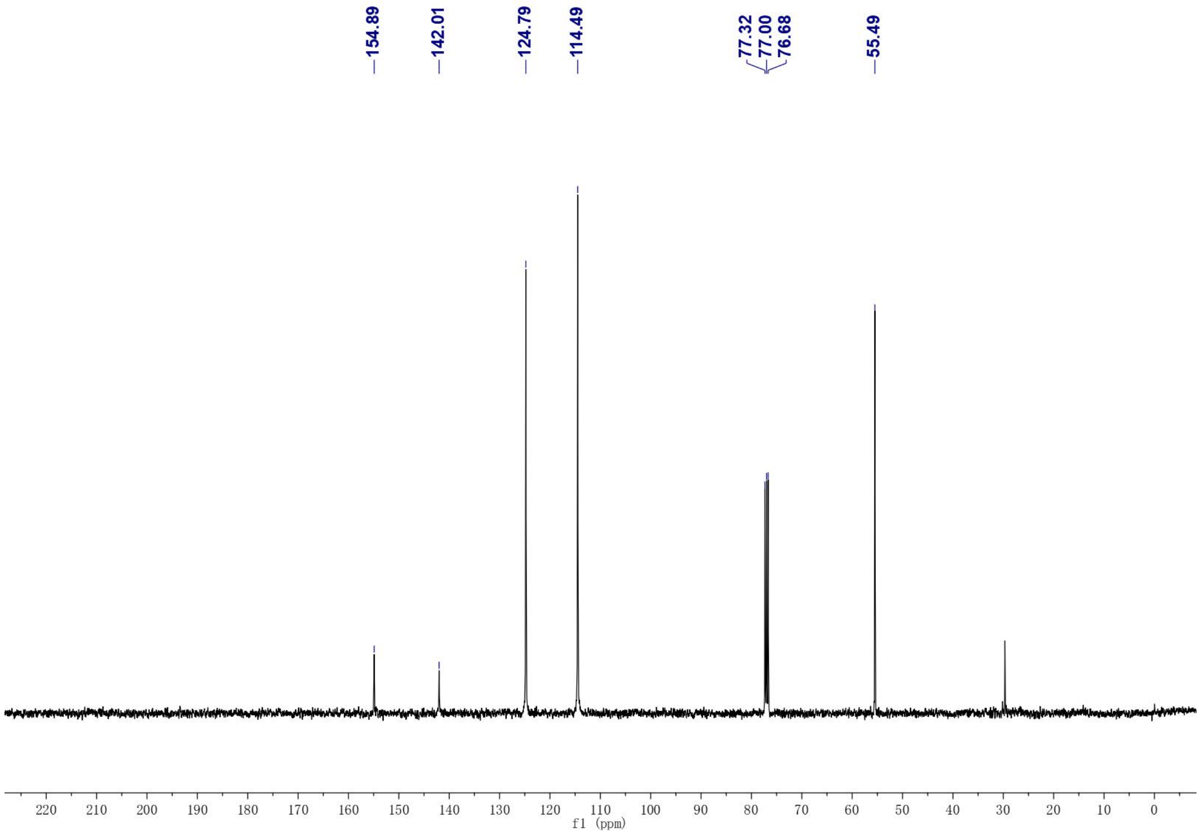

Supplementary Figure 58. ^{13}C NMR Spectrum of **3f**

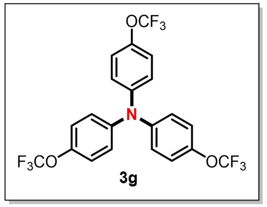

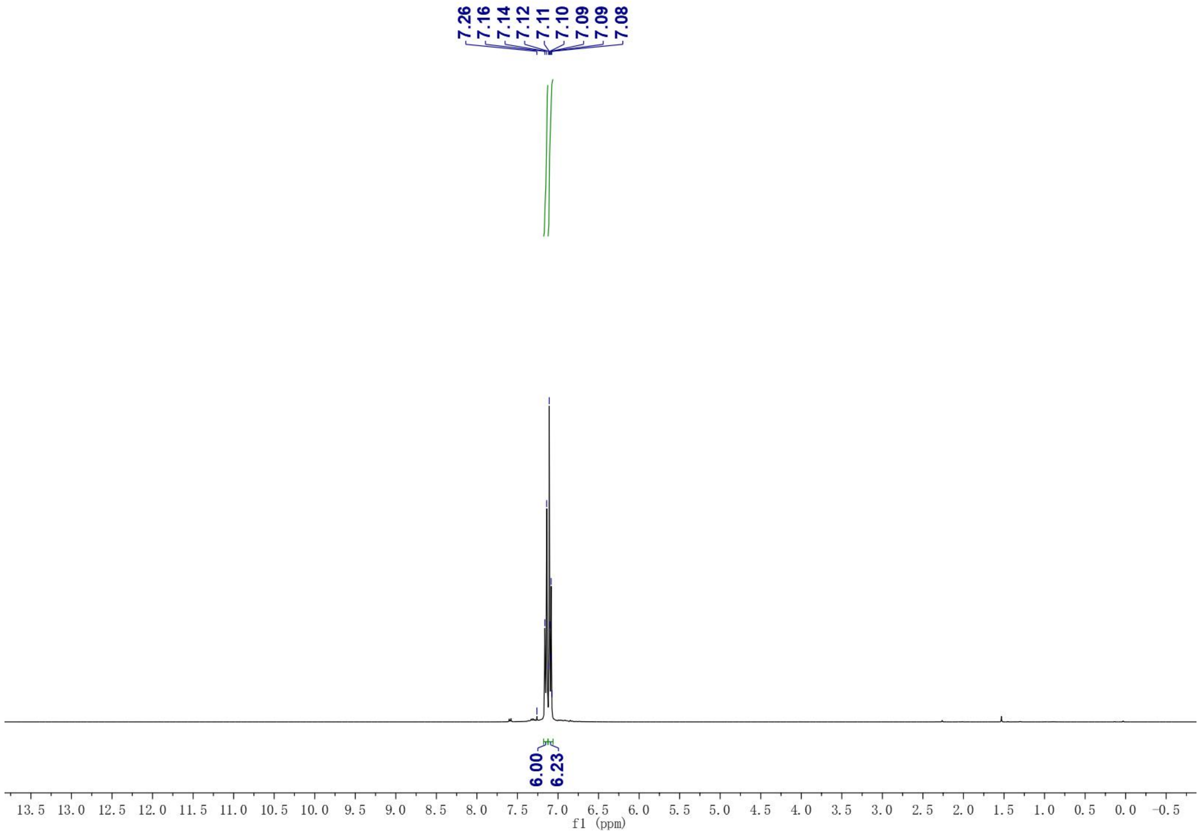

Supplementary Figure 59. ^{1}H NMR Spectrum of **3g**

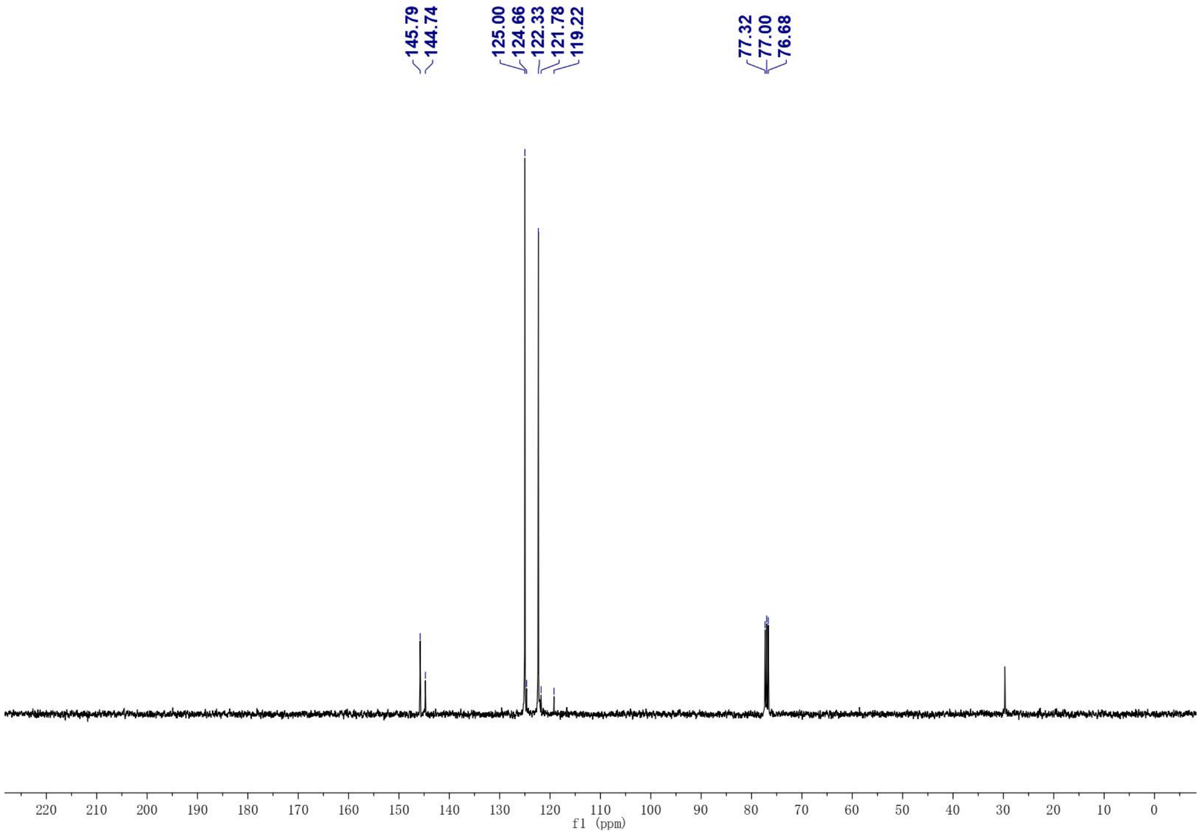

Supplementary Figure 60. ^{13}C NMR Spectrum of **3g**

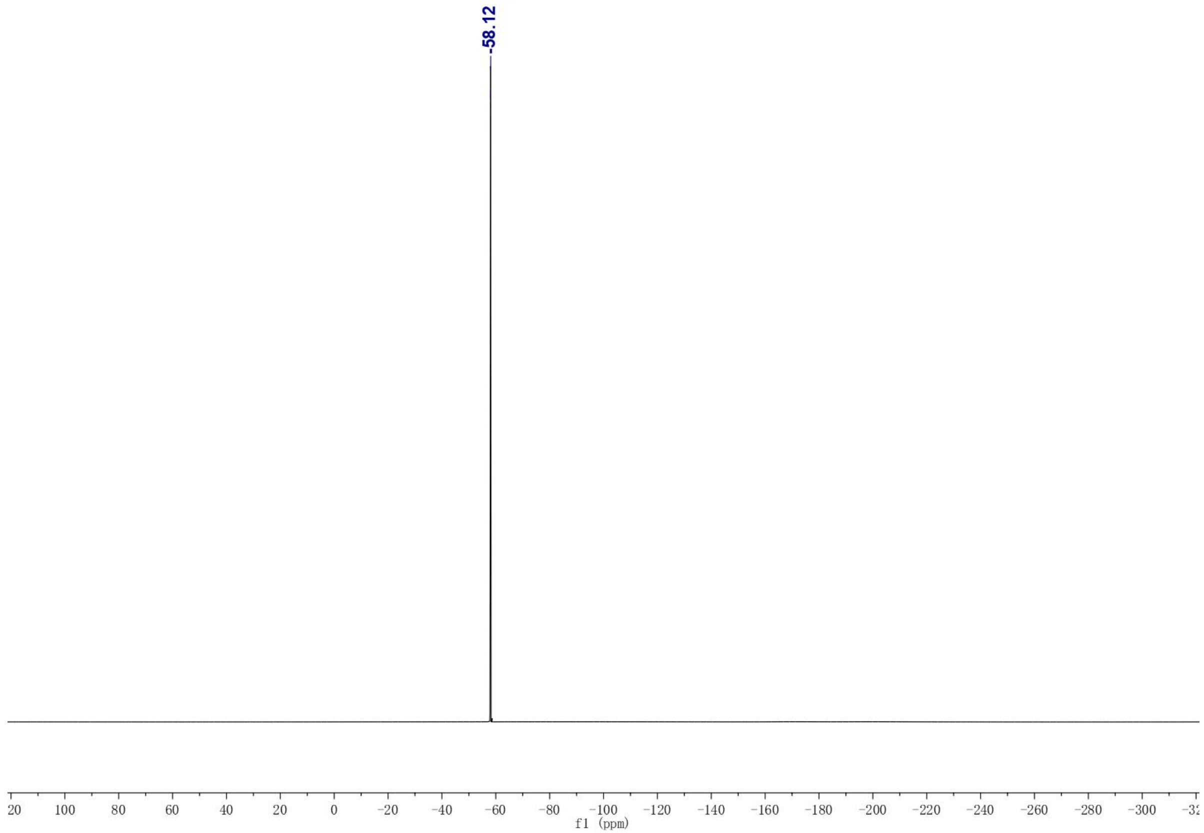

Supplementary Figure 61. ^{19}F NMR Spectrum of **3g**

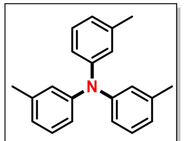

3h

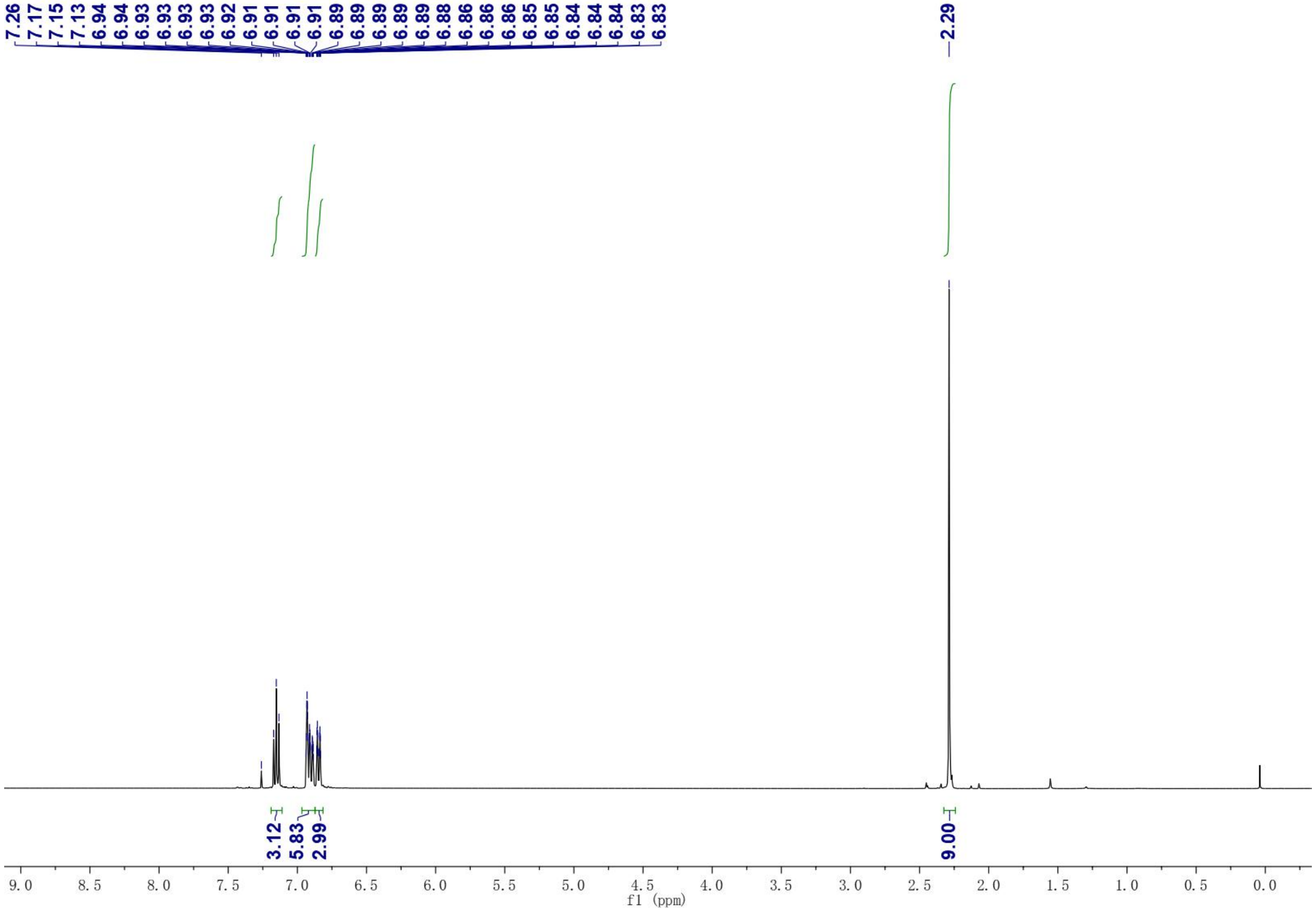

Supplementary Figure 62. ^{1}H NMR Spectrum of **3h**

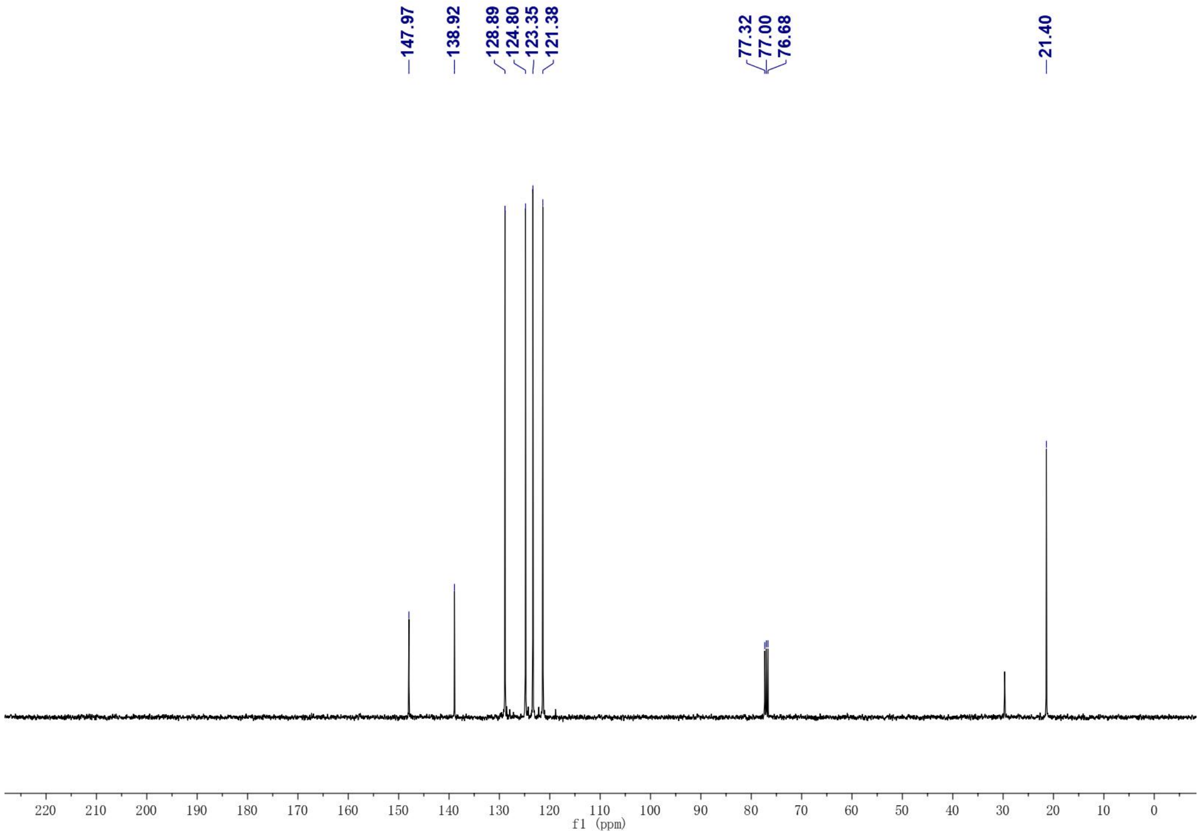

Supplementary Figure 63. ^{13}C NMR Spectrum of **3h**

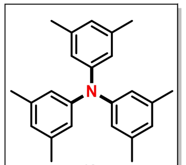

3i

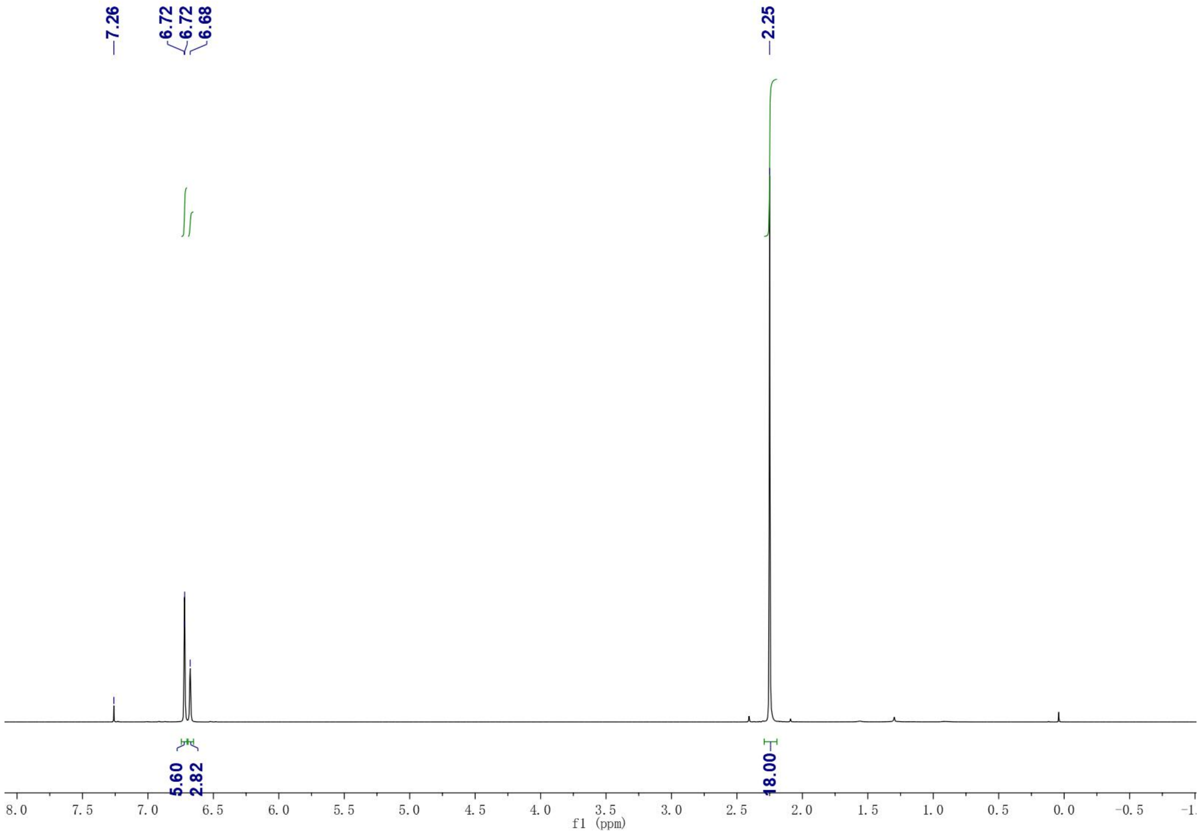

Supplementary Figure 64. ^{1}H NMR Spectrum of **3i**

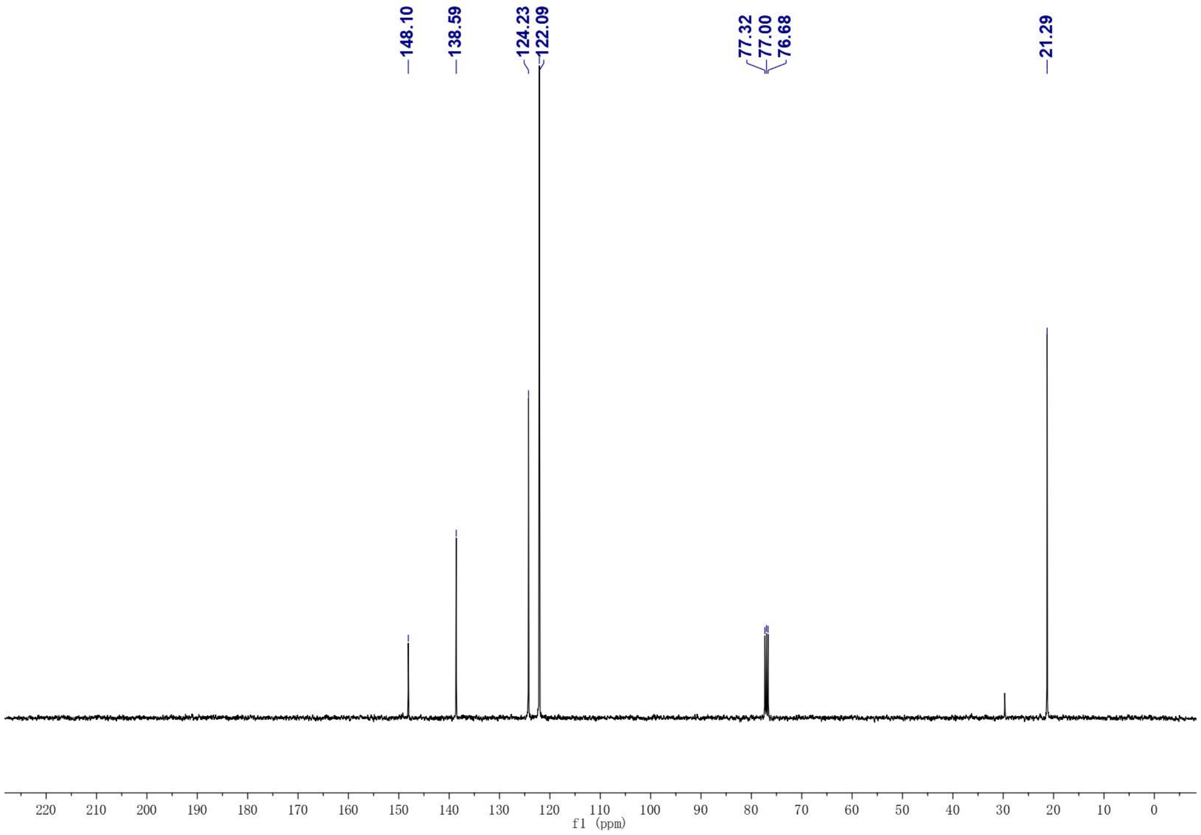

Supplementary Figure 65. ^{13}C NMR Spectrum of **3i**

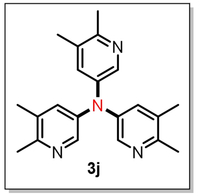

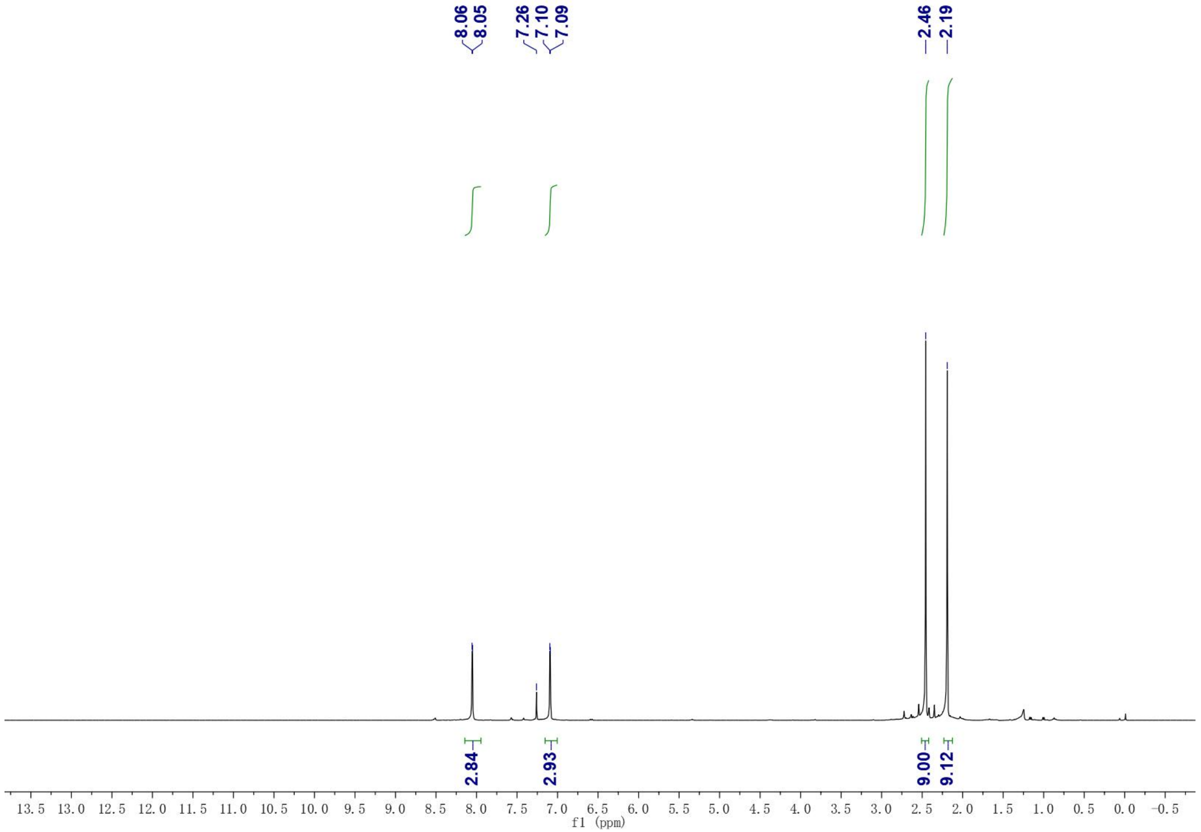

Supplementary Figure 66. ^{1}H NMR Spectrum of **3j**

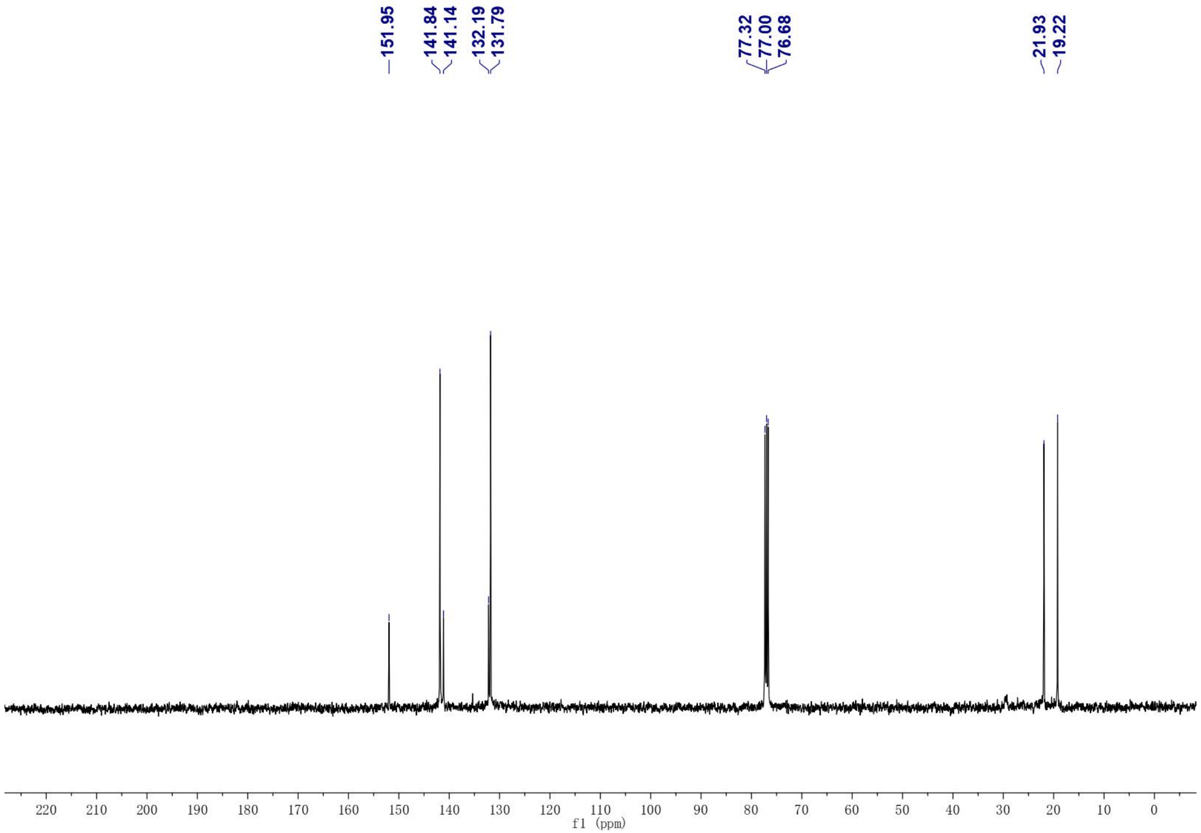

Supplementary Figure 67. ^{13}C NMR Spectrum of **3g**

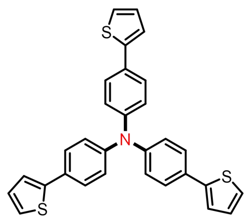

3k

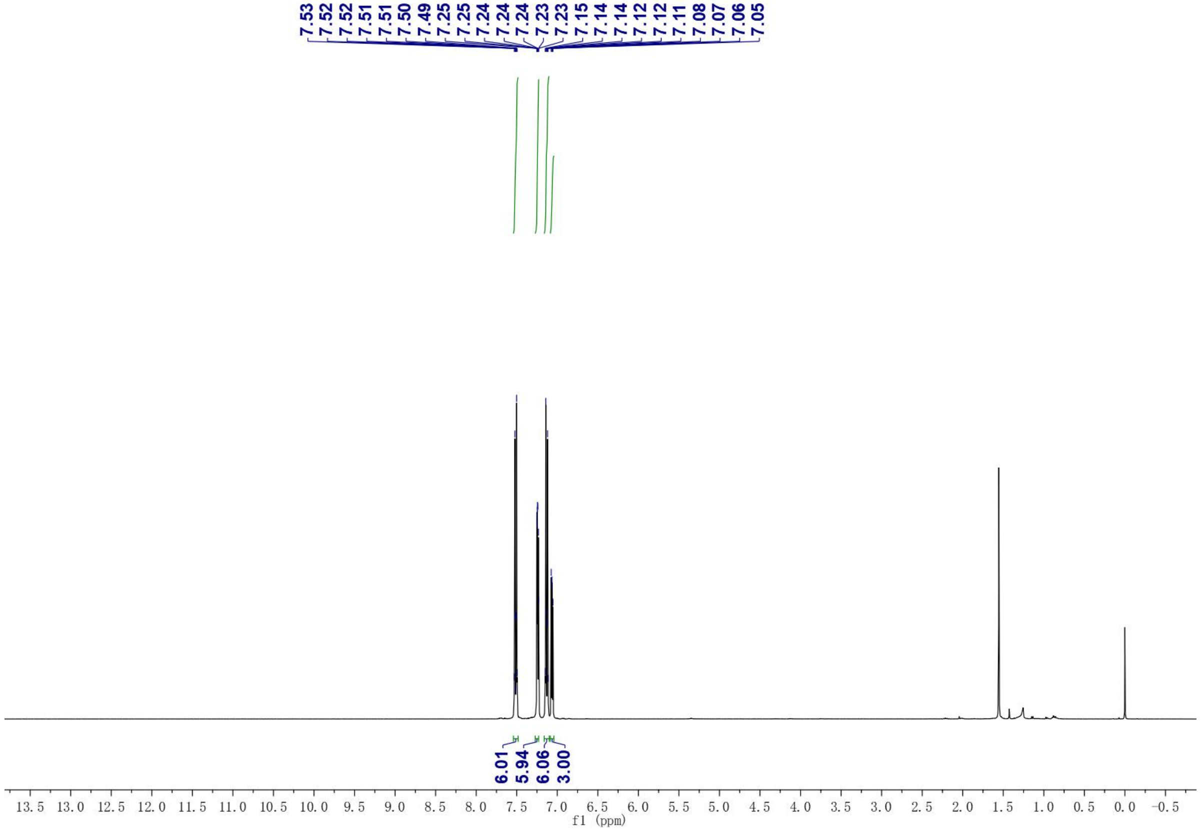

Supplementary Figure 68. ^{1}H NMR Spectrum of **3k**

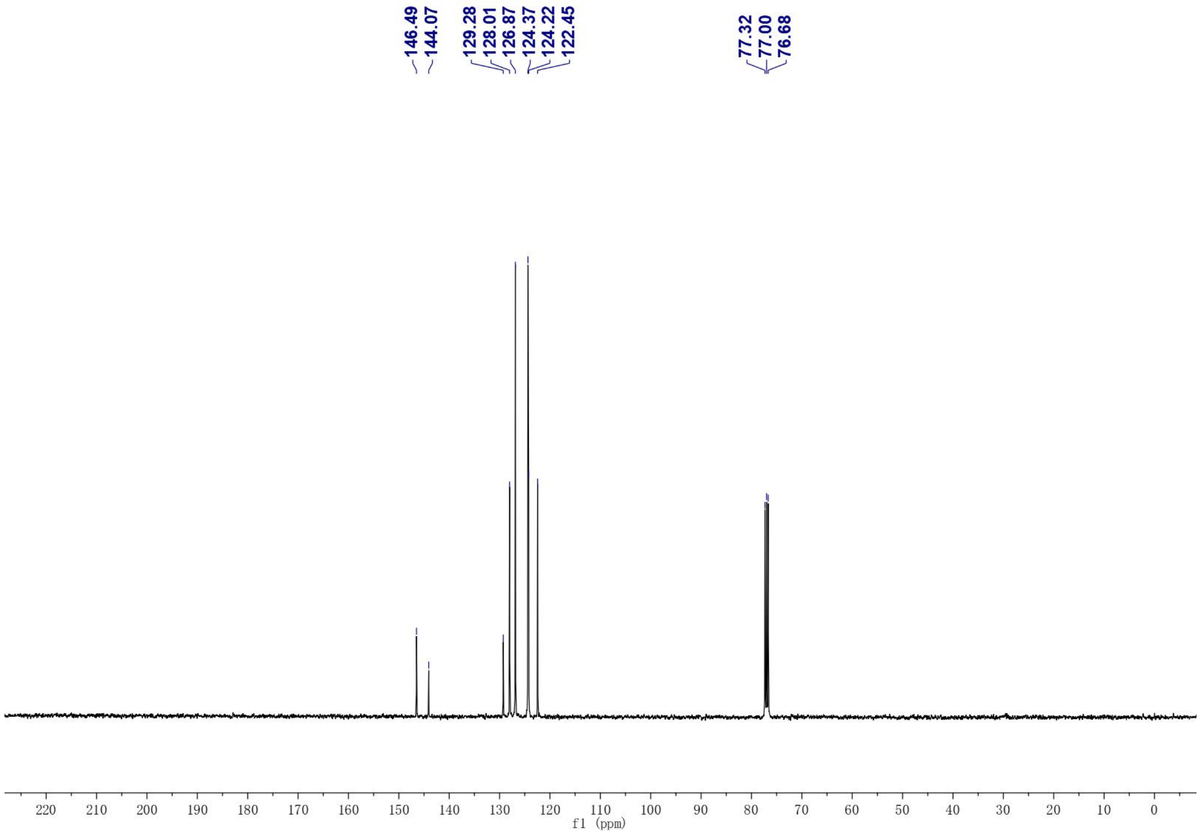

Supplementary Figure 69. ^{13}C NMR Spectrum of **3k**

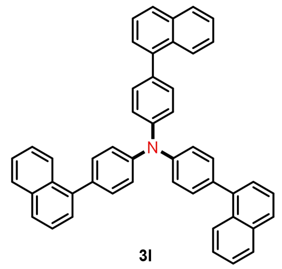

3l

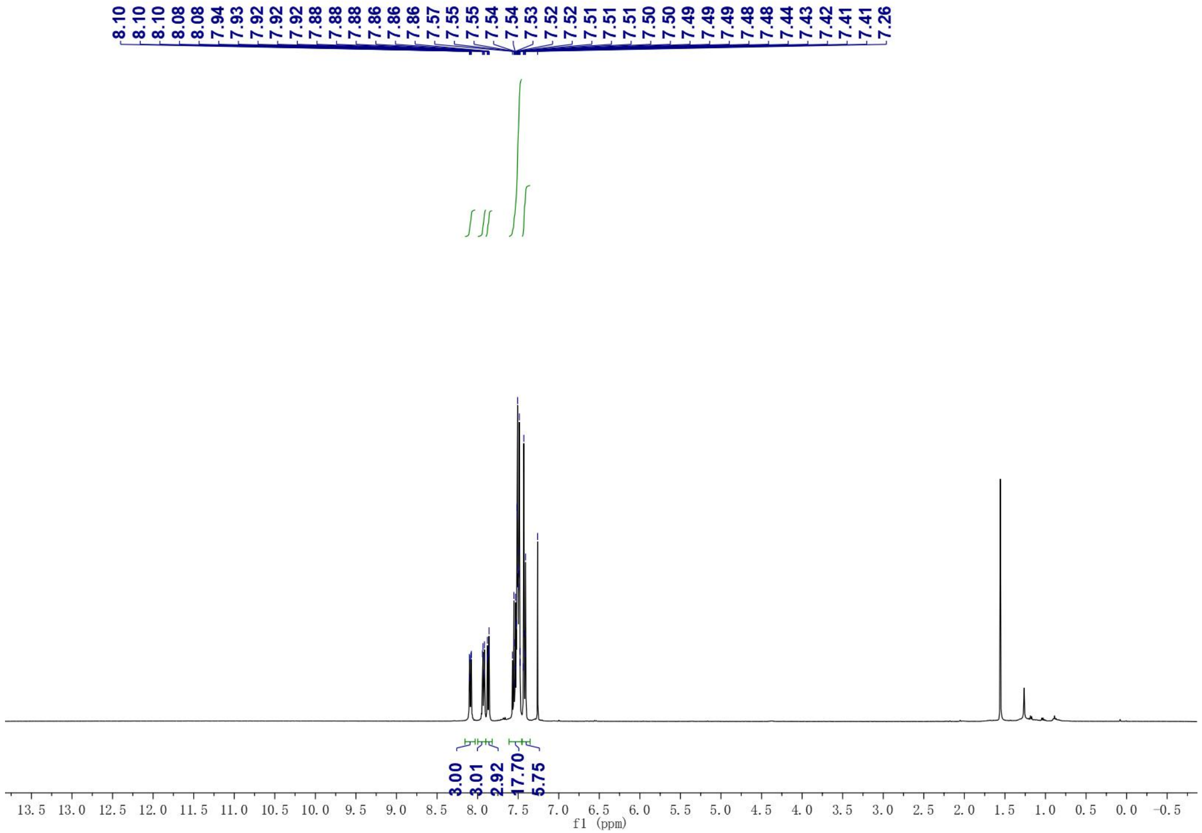

Supplementary Figure 70. ^{1}H NMR Spectrum of **3l**

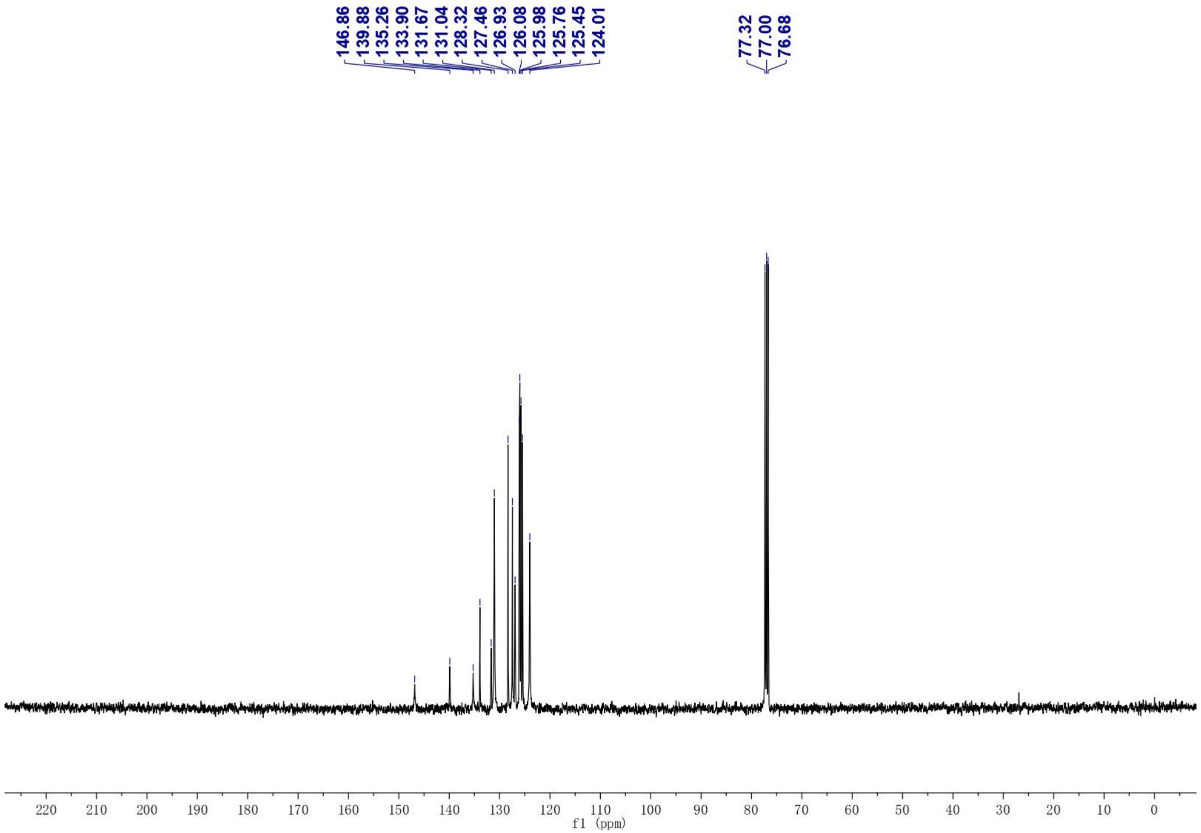

Supplementary Figure 71. ^{13}C NMR Spectrum of **3l**

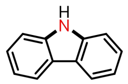

5a

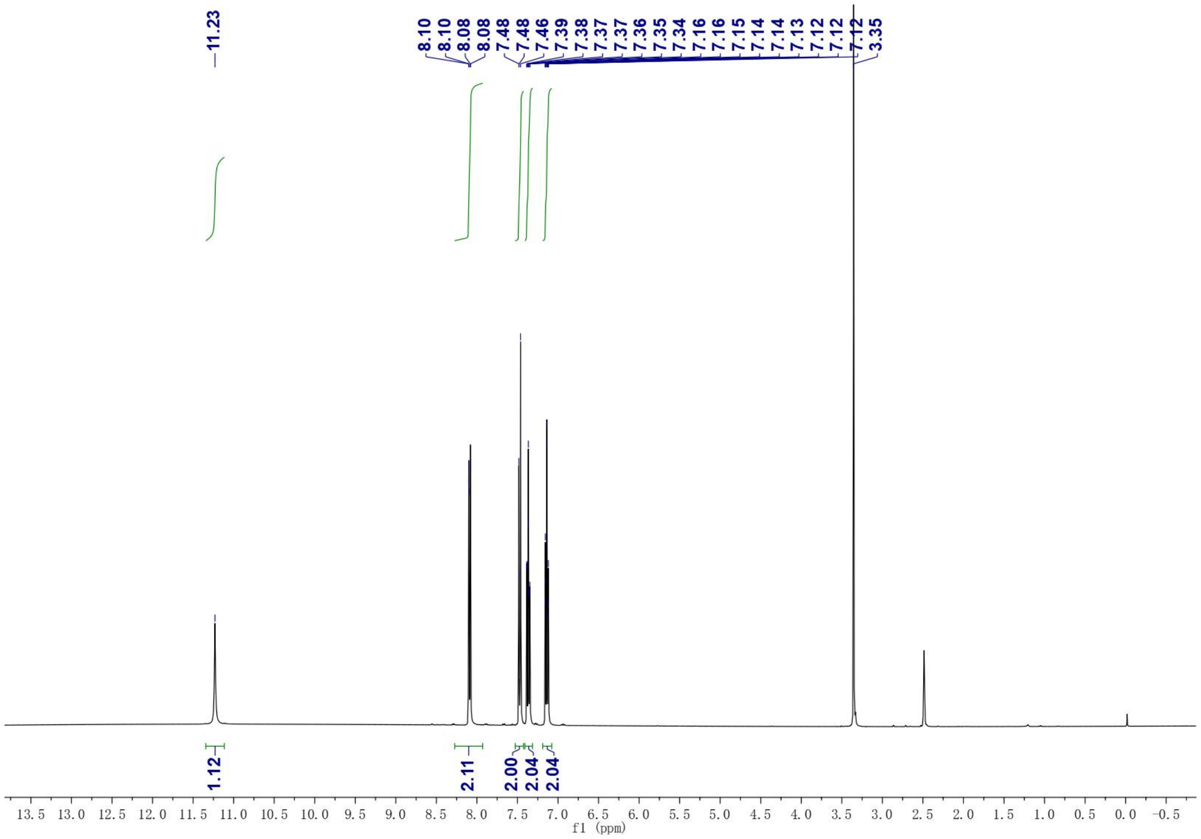

Supplementary Figure 72. ^{1}H NMR Spectrum of **5a**

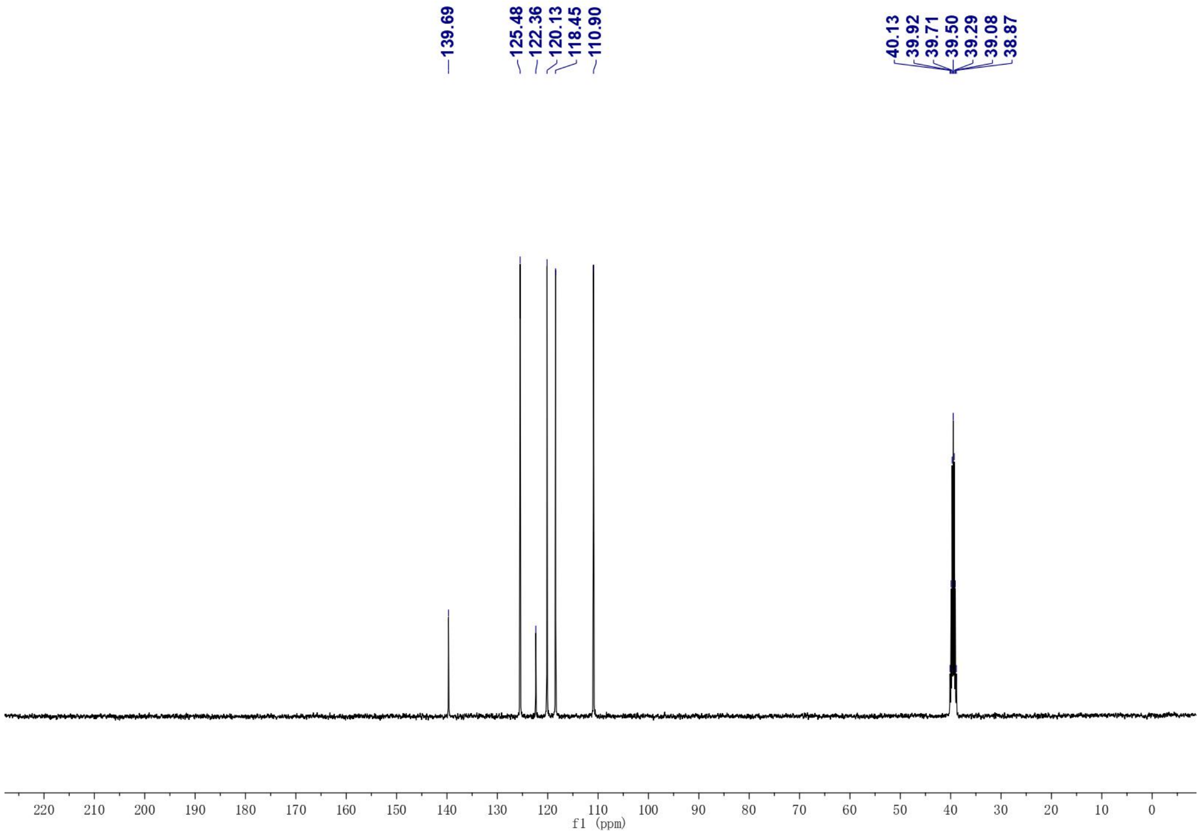

Supplementary Figure 73. ^{13}C NMR Spectrum of **5a**

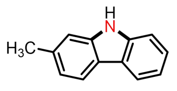

5b

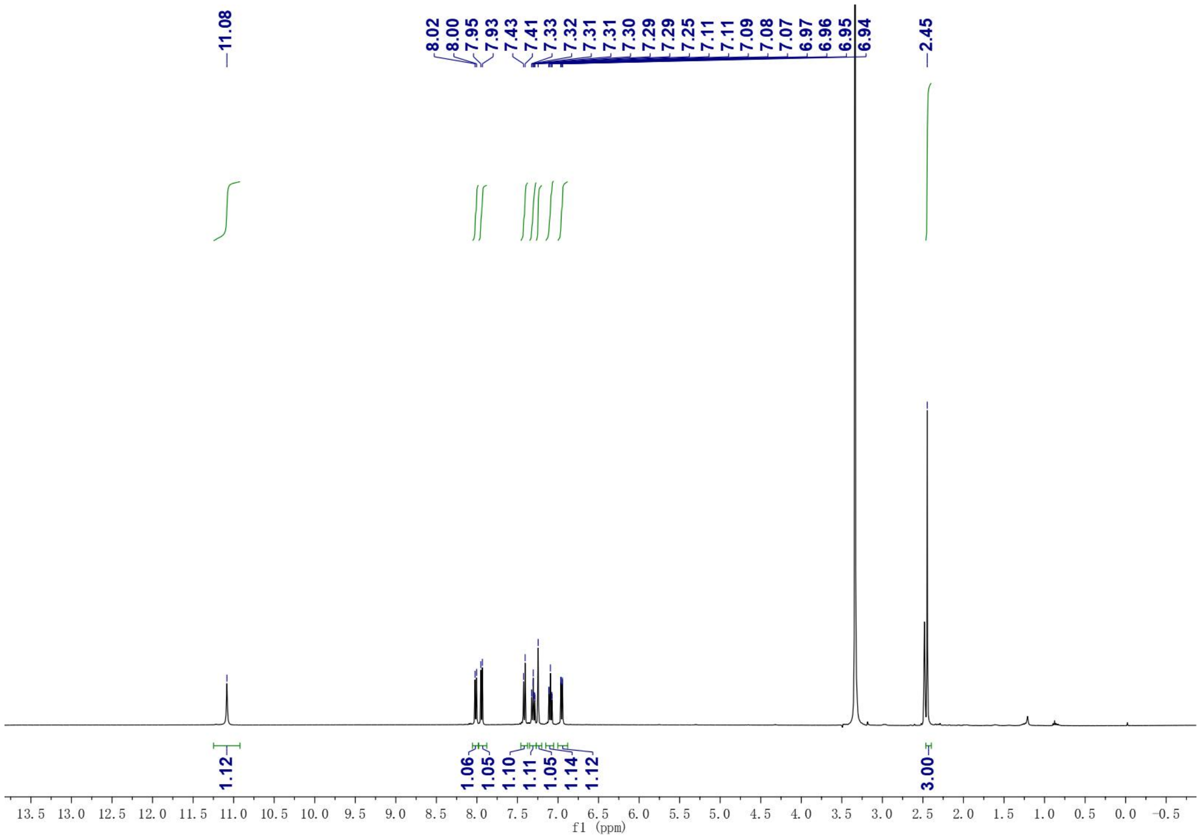

Supplementary Figure 74. ^{1}H NMR Spectrum of **5b**

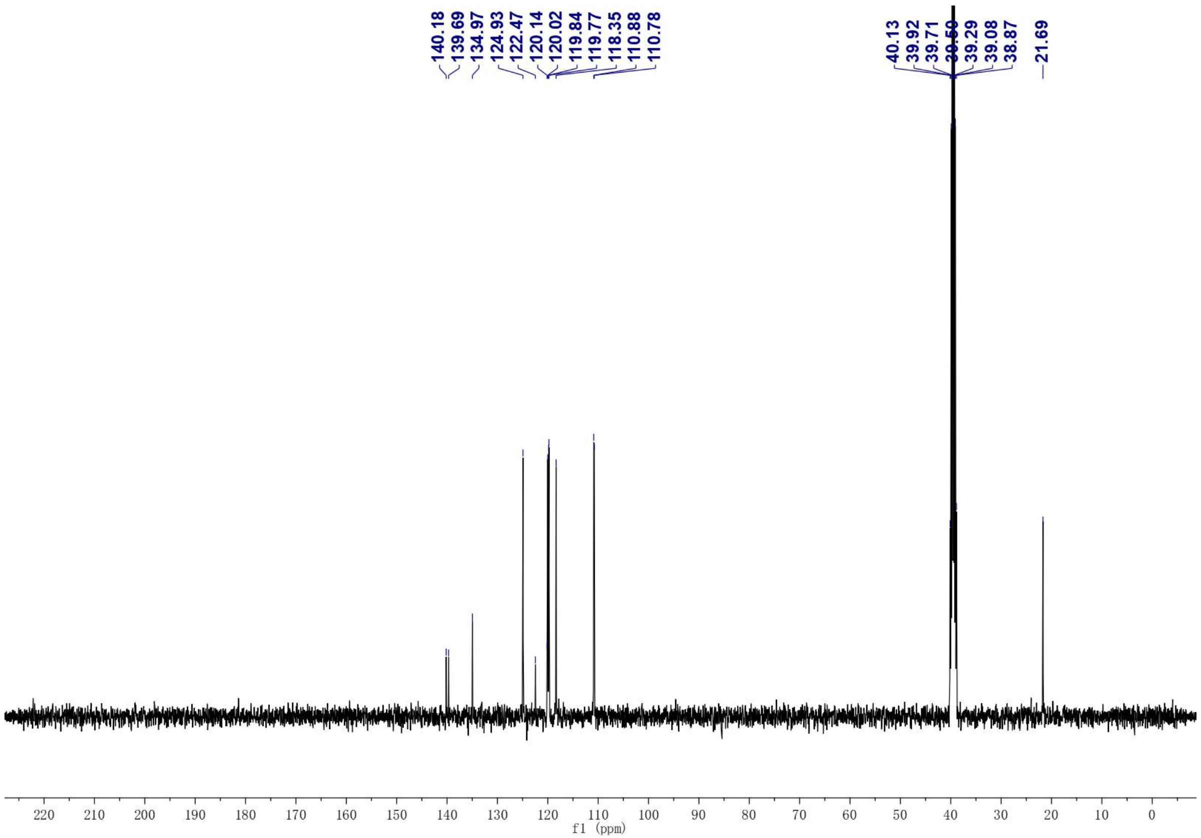

Supplementary Figure 75. ^{13}C NMR Spectrum of **5b**

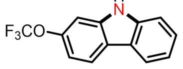

5c

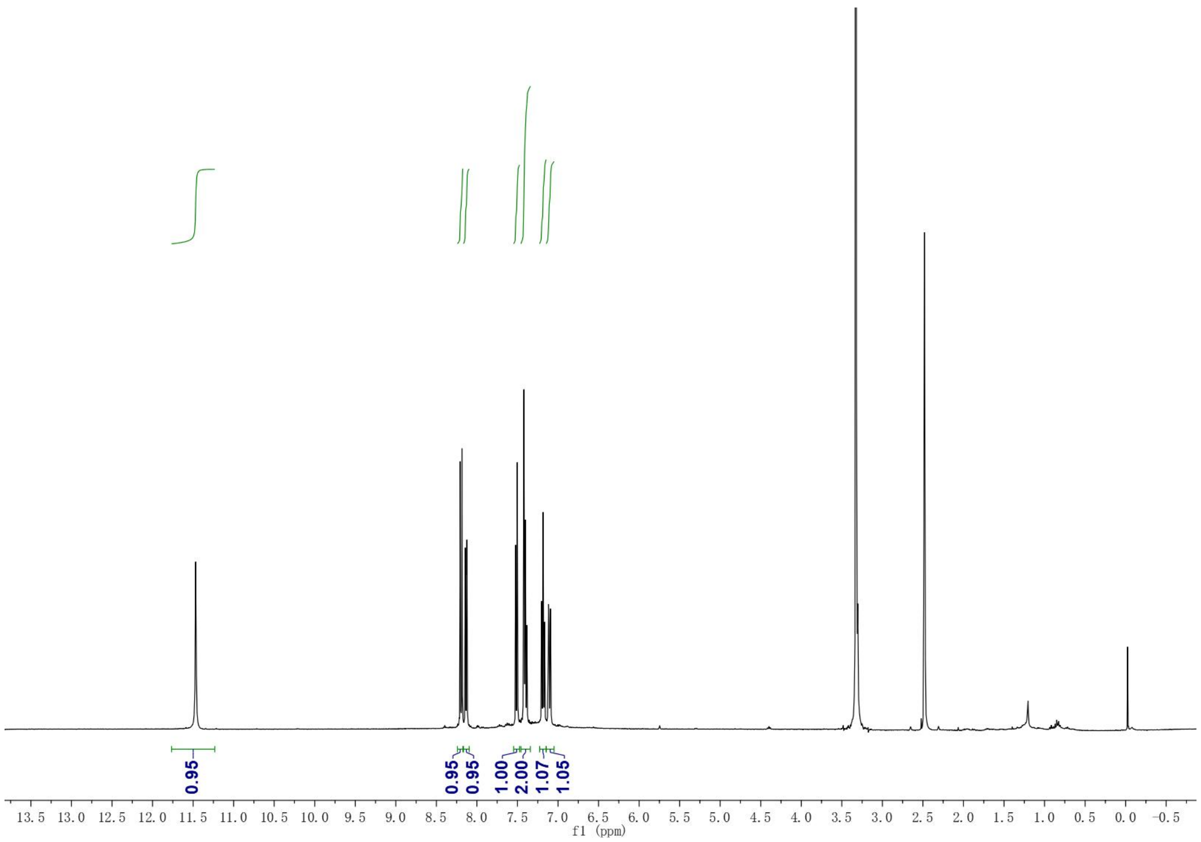

Supplementary Figure 76. ^{1}H NMR Spectrum of **5c**

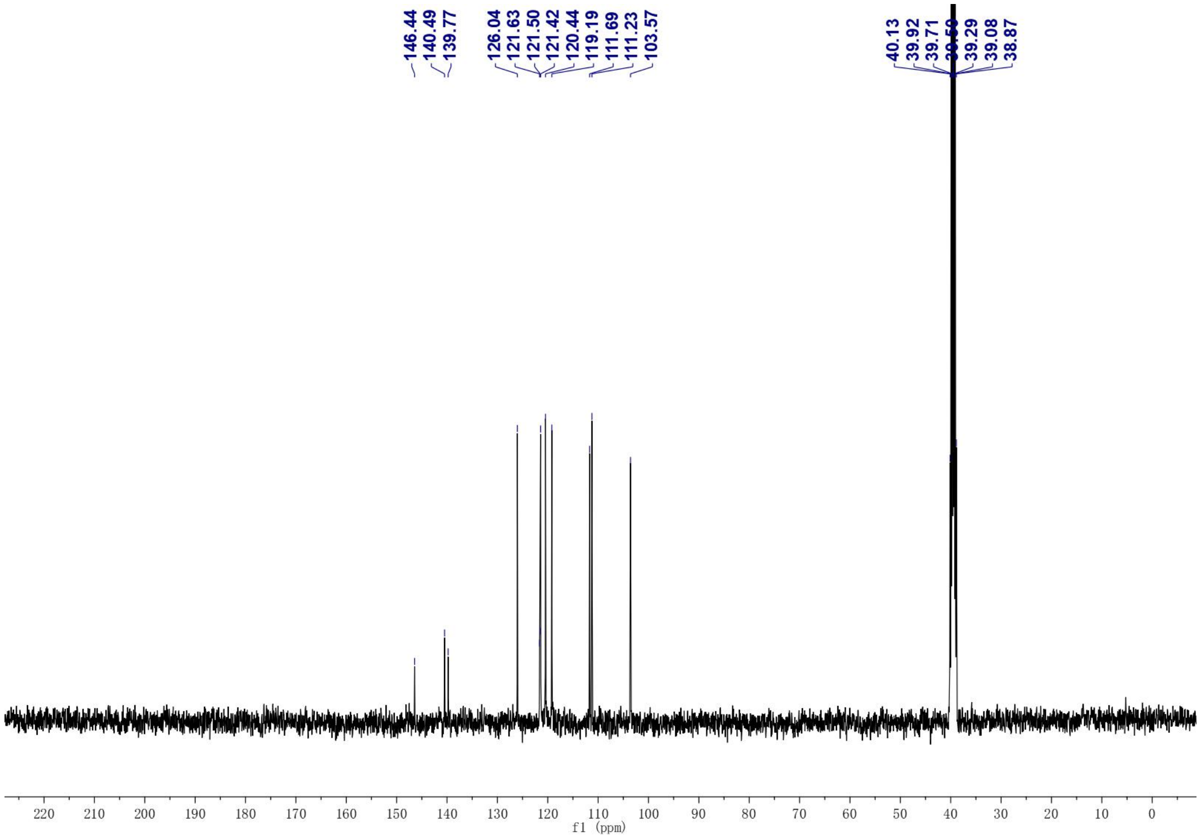

Supplementary Figure 77. ^{13}C NMR Spectrum of **5c**

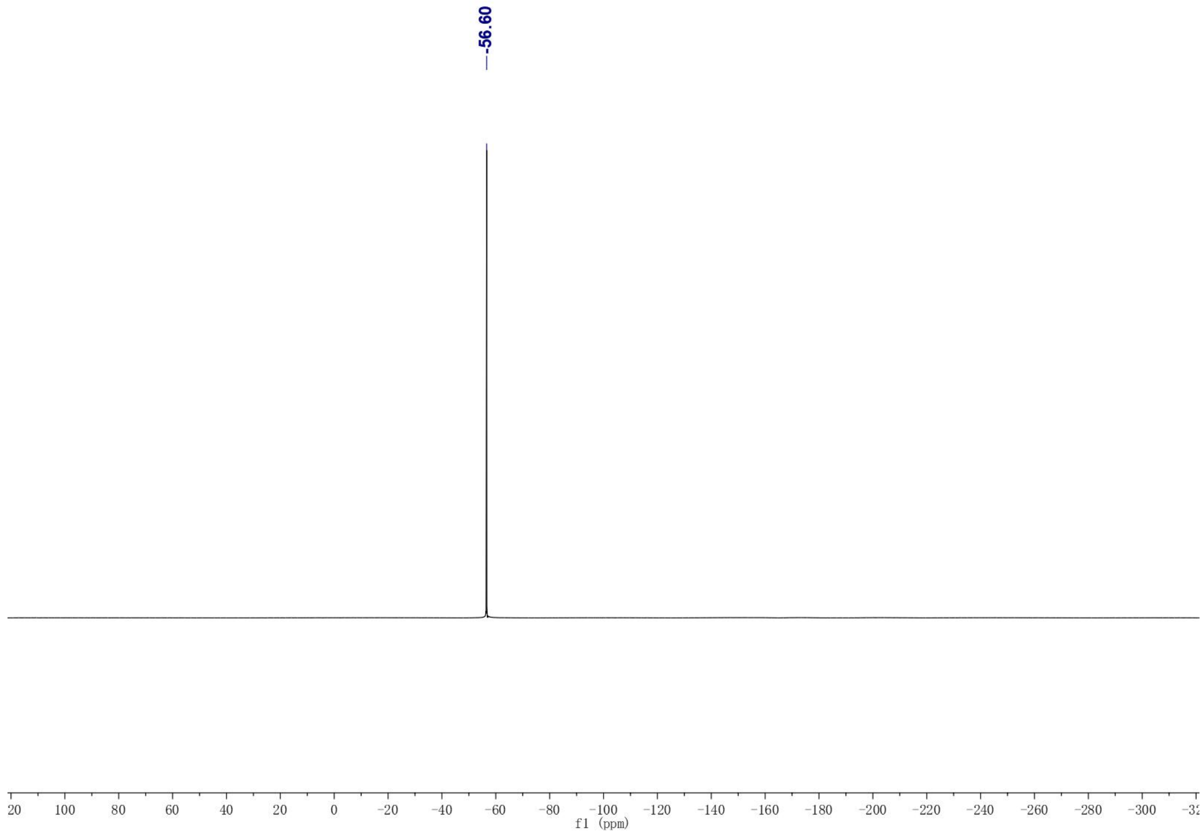

Supplementary Figure 78. ^{19}F NMR Spectrum of **5c**

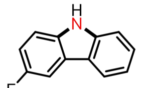

5d

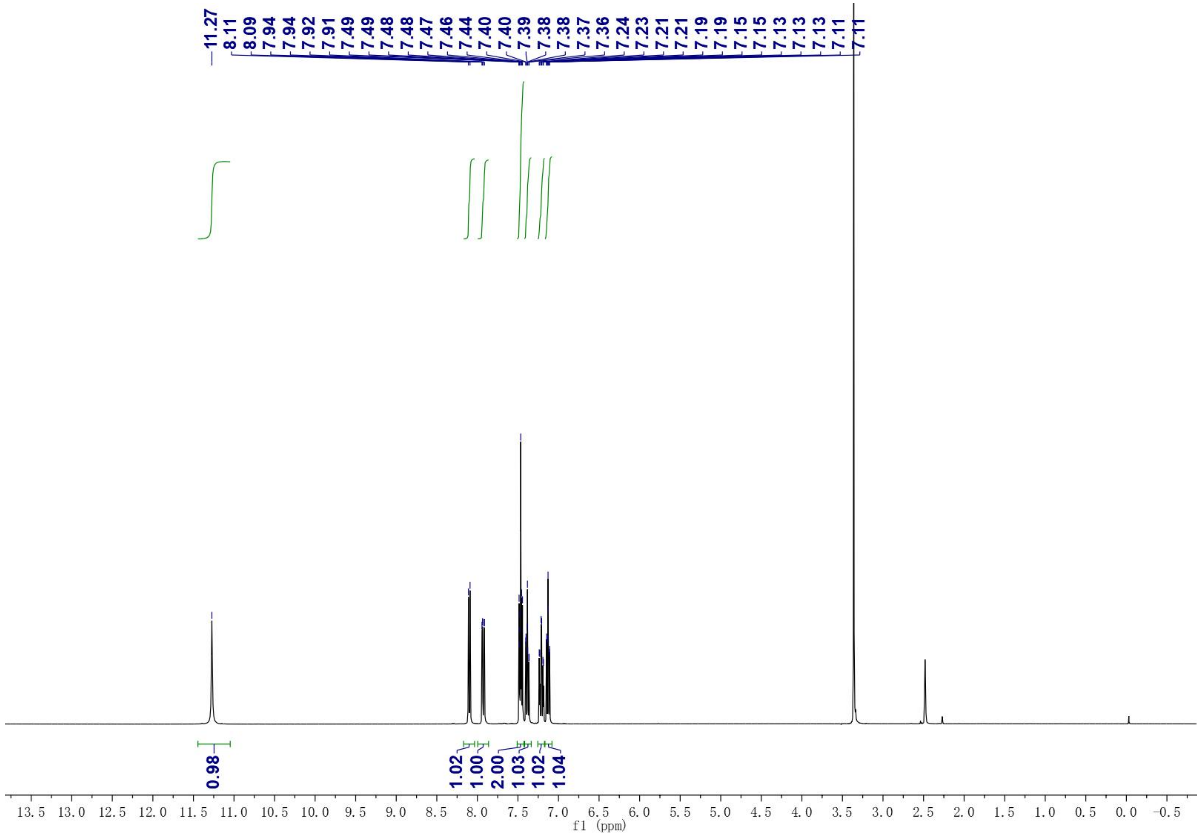

Supplementary Figure 79. ^{1}H NMR Spectrum of **5d**

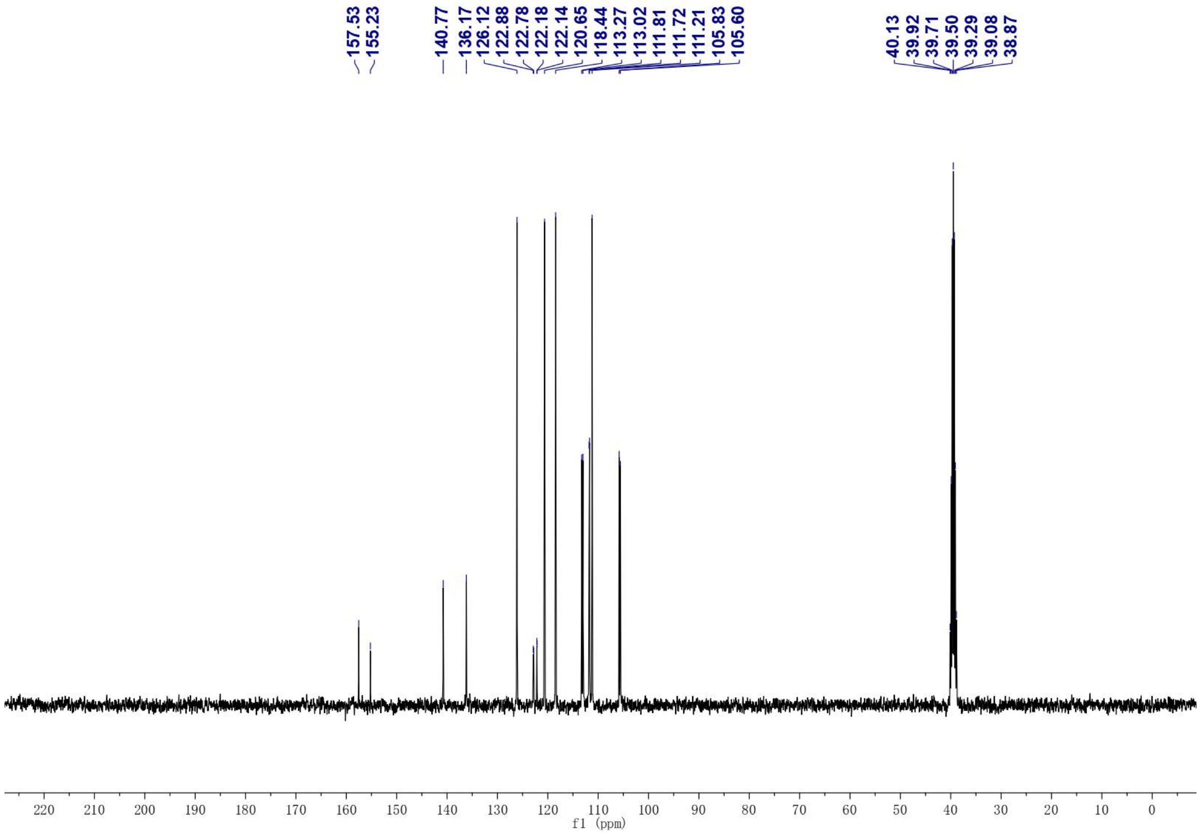

Supplementary Figure 80. ^{13}C NMR Spectrum of **5d**

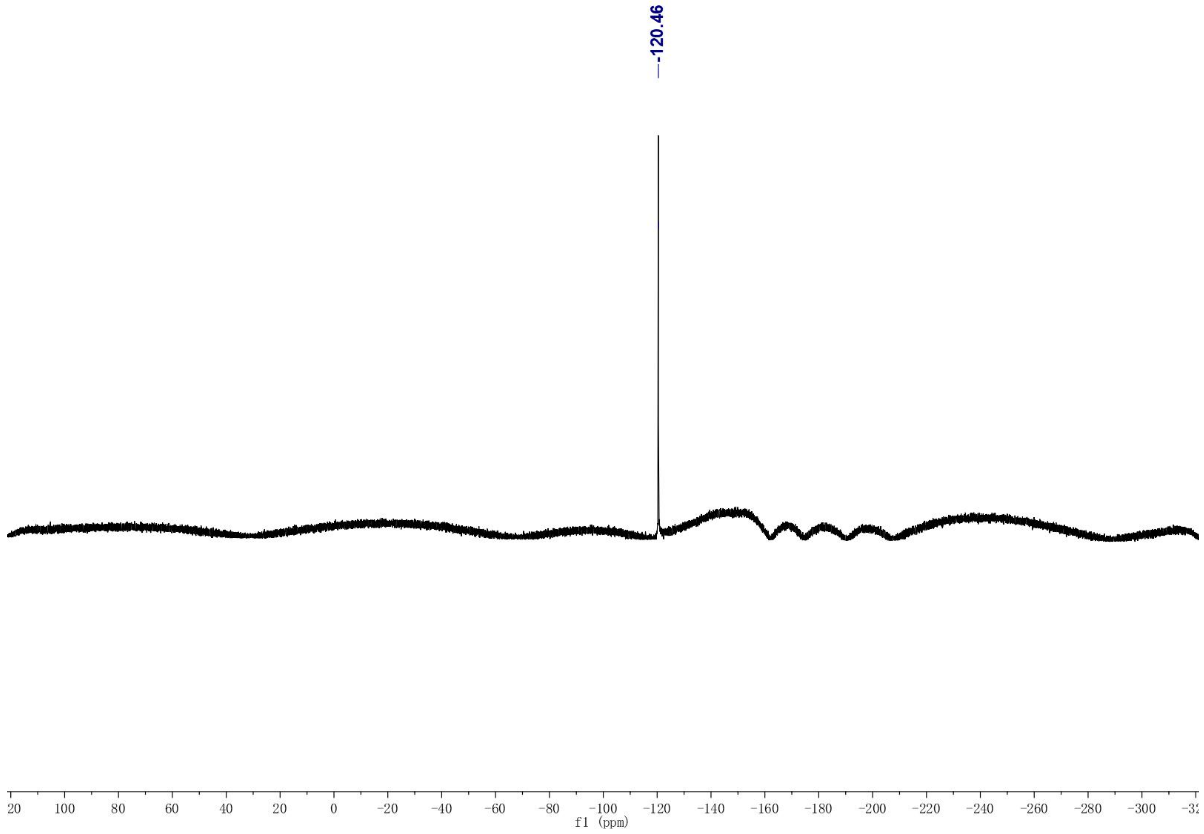

Supplementary Figure 81. ^{19}F NMR Spectrum of **5d**

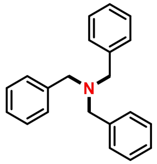

7

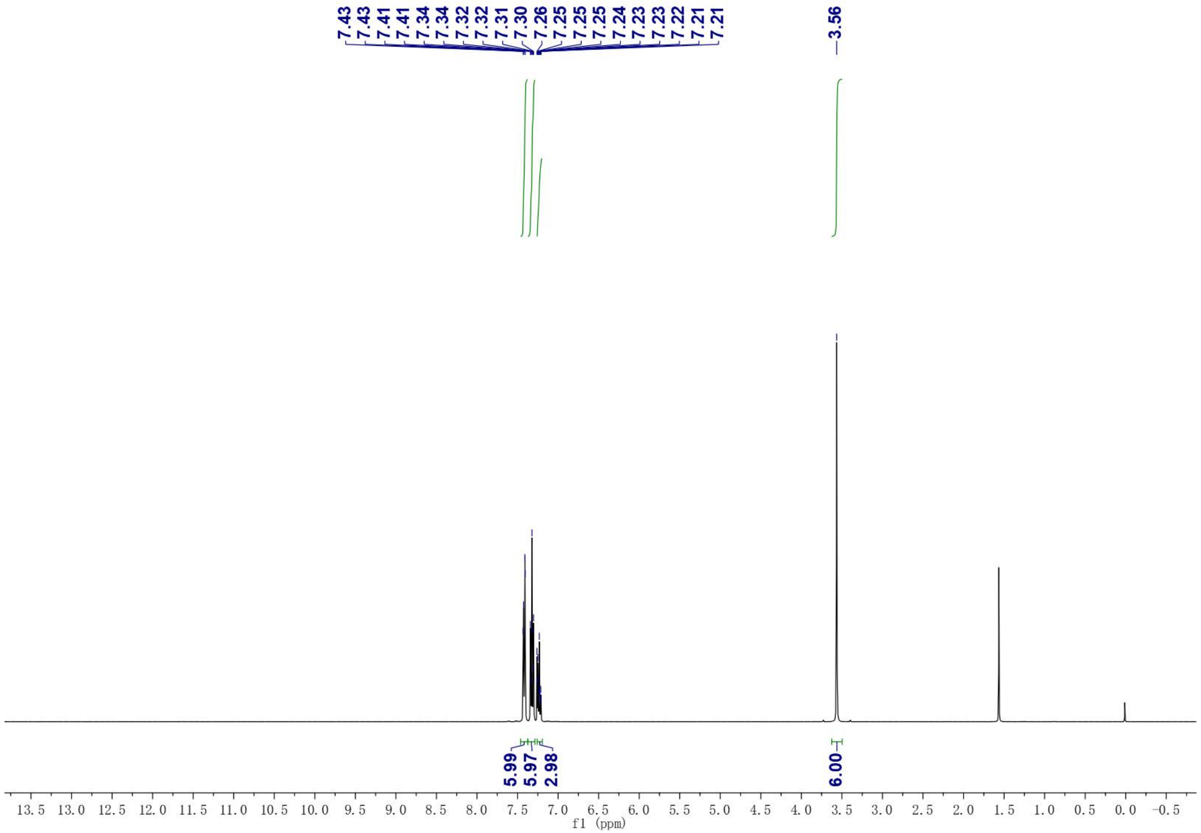

Supplementary Figure 82. ^{1}H NMR Spectrum of **7**

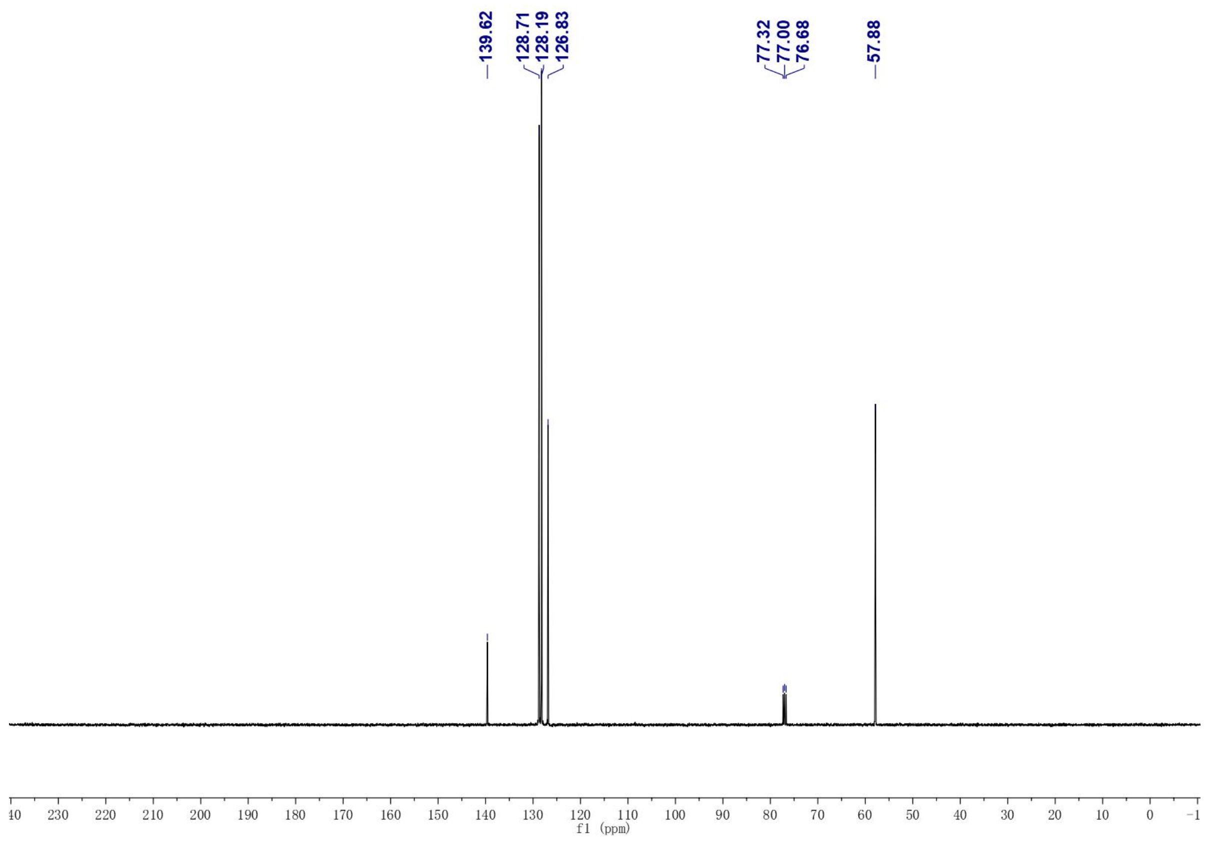

Supplementary Figure 83. ^{13}C NMR Spectrum of **7**

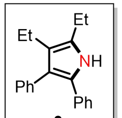

9

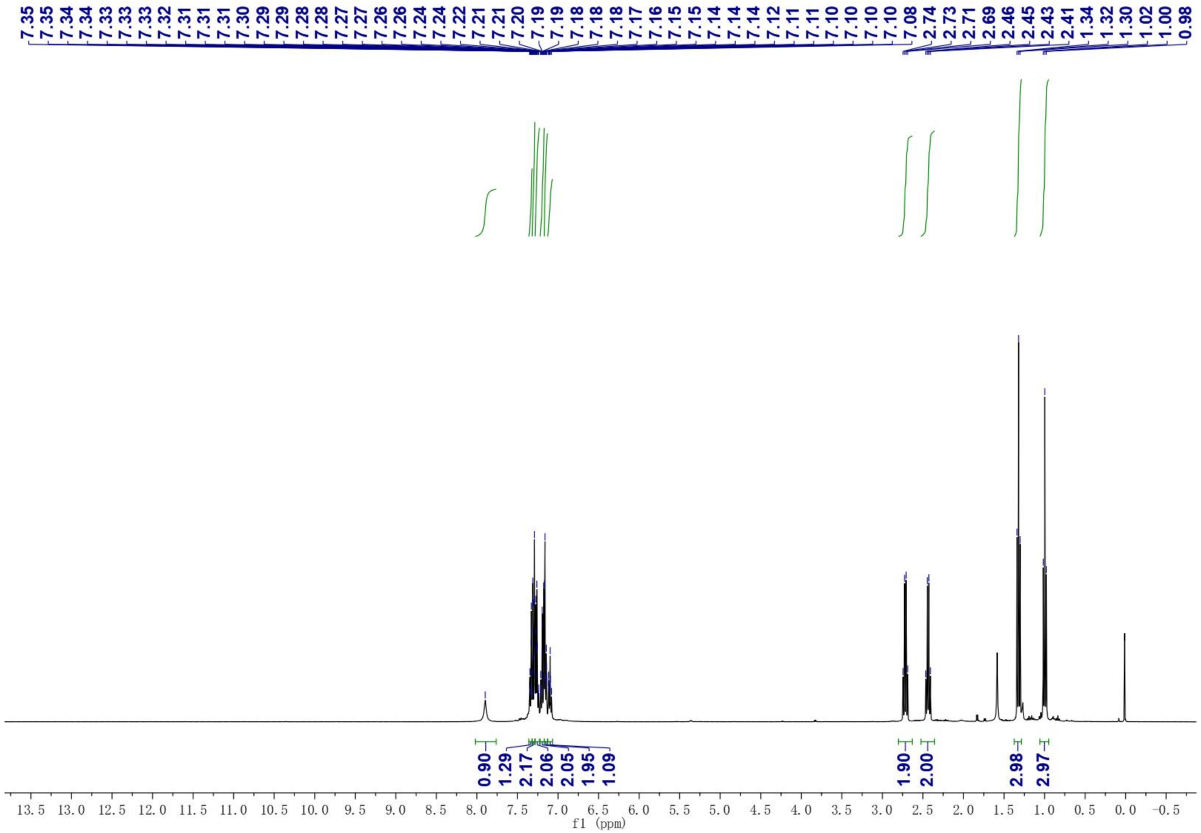

Supplementary Figure 84. ^{1}H NMR Spectrum of **9**

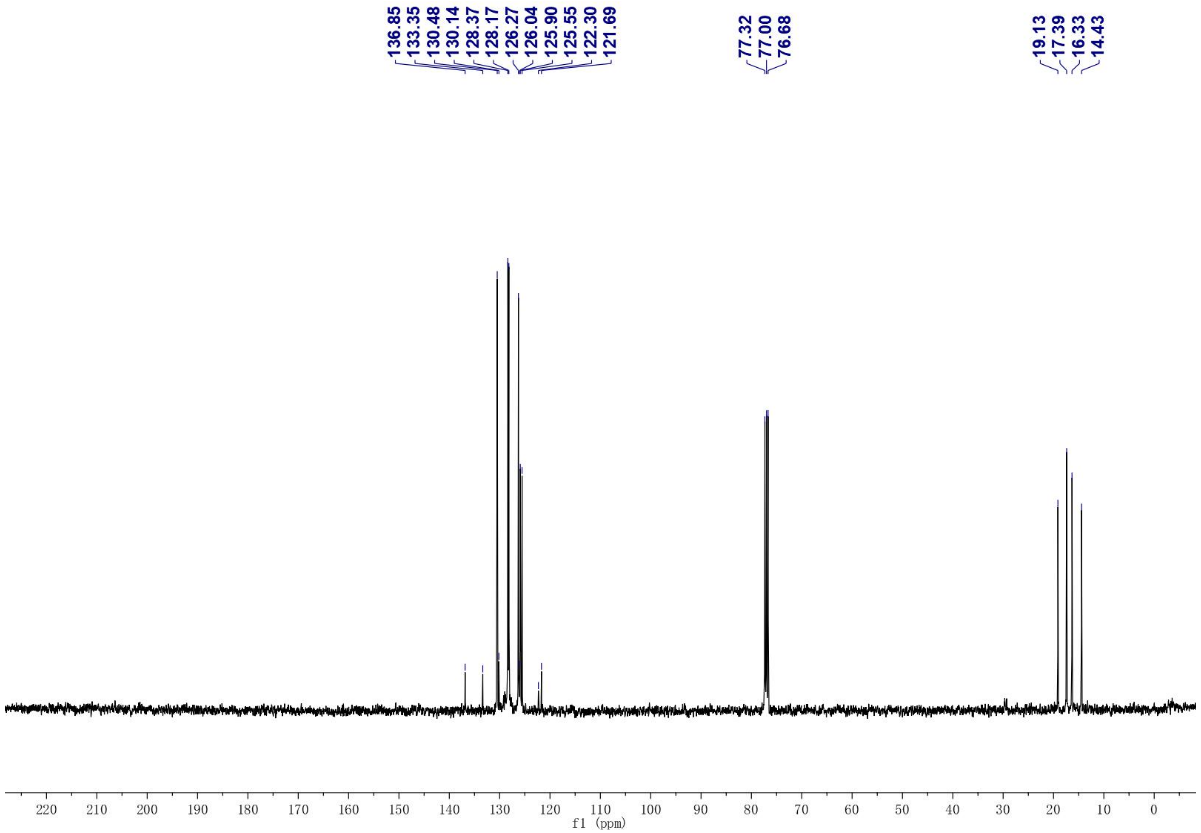

Supplementary Figure 85. ^{13}C NMR Spectrum of **9**

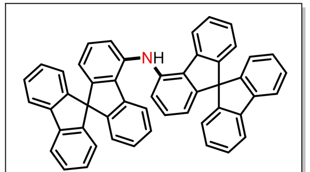

11

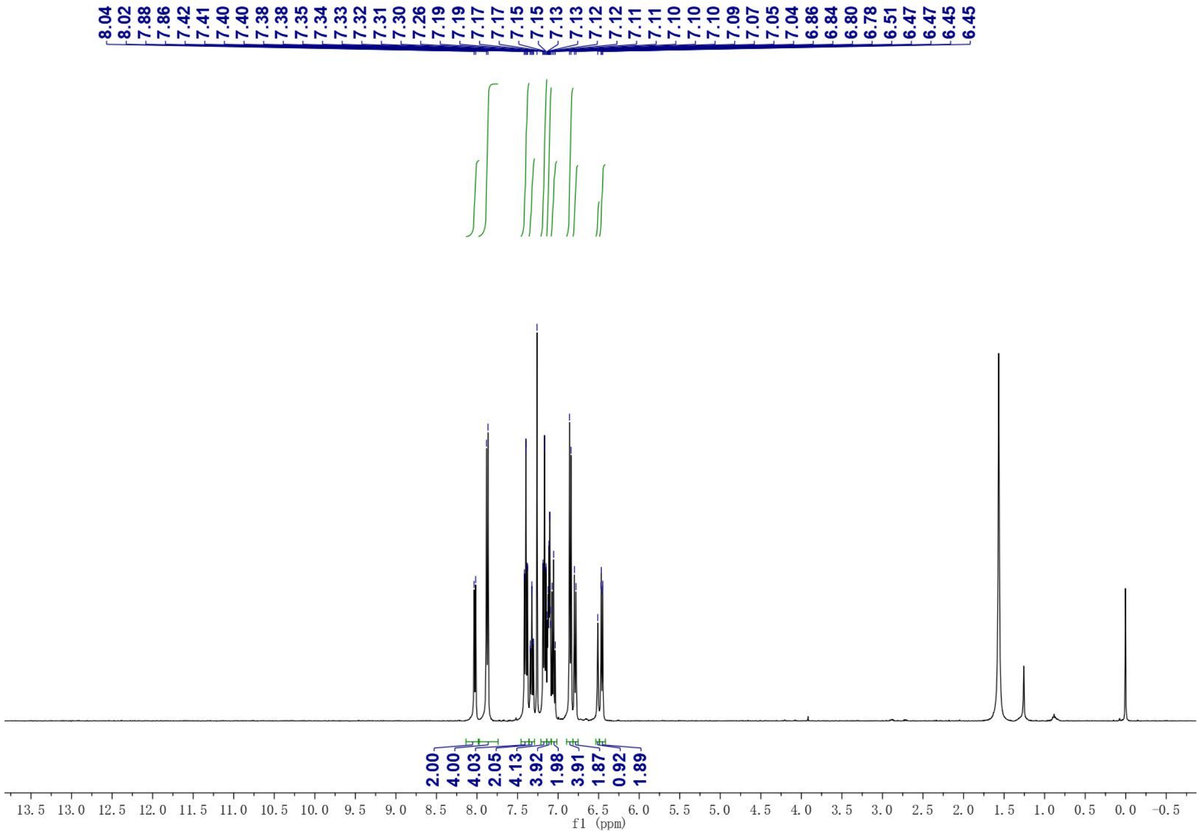

Supplementary Figure 86. ^{1}H NMR Spectrum of **11**

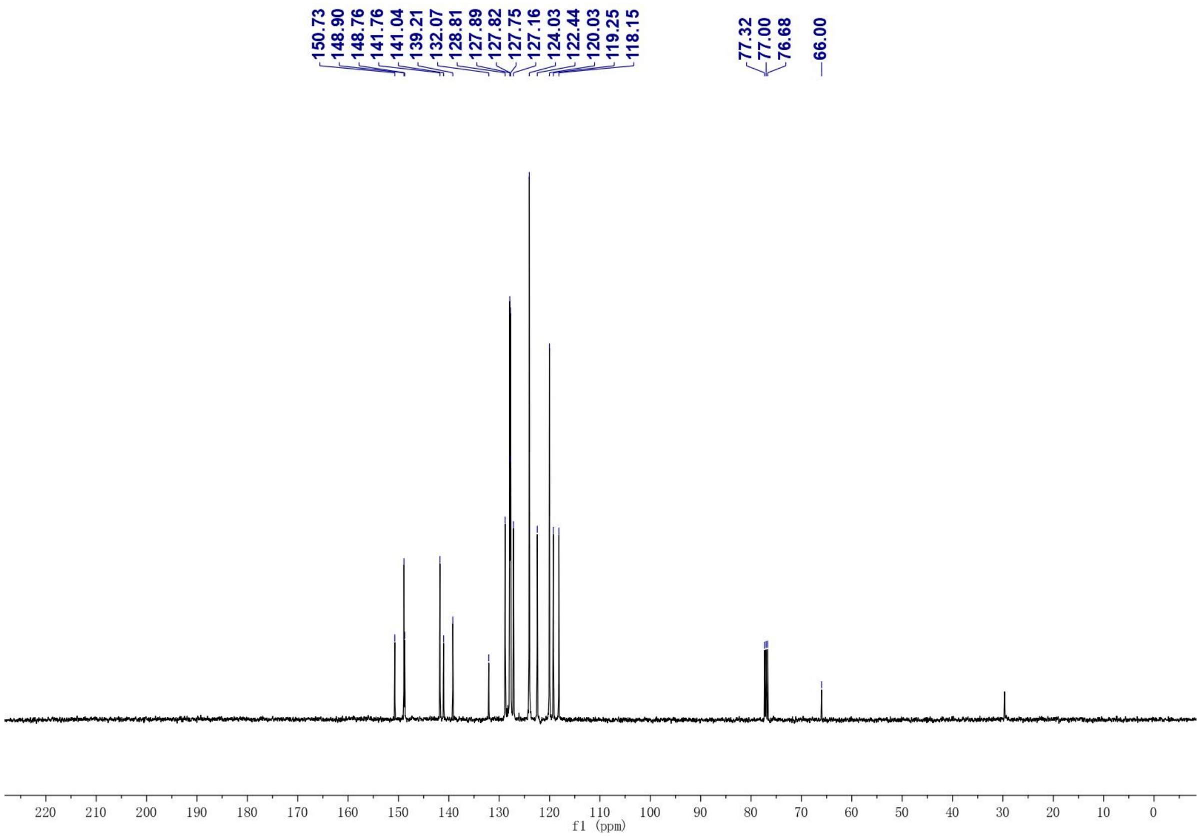

Supplementary Figure 87. ^{13}C NMR Spectrum of **11**

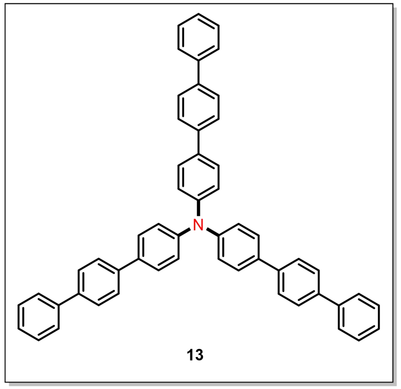

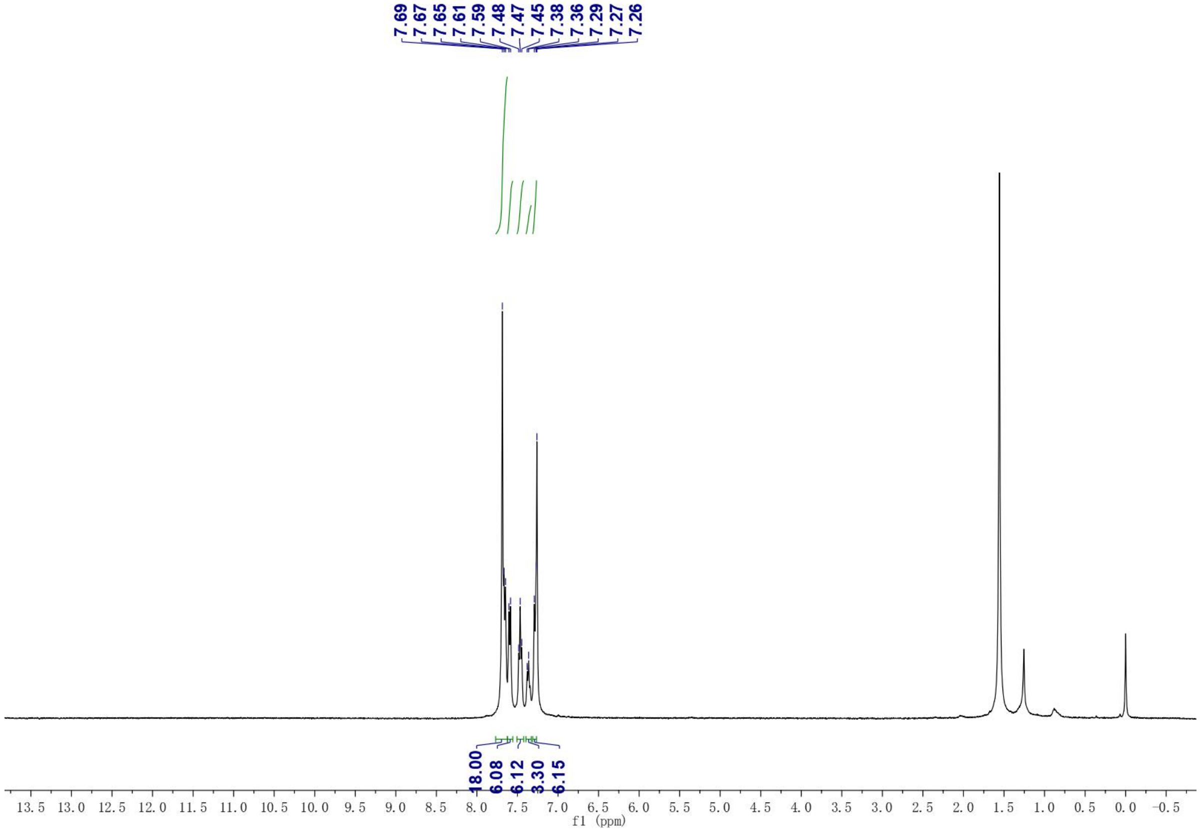

Supplementary Figure 88. ^{1}H NMR Spectrum of **13**

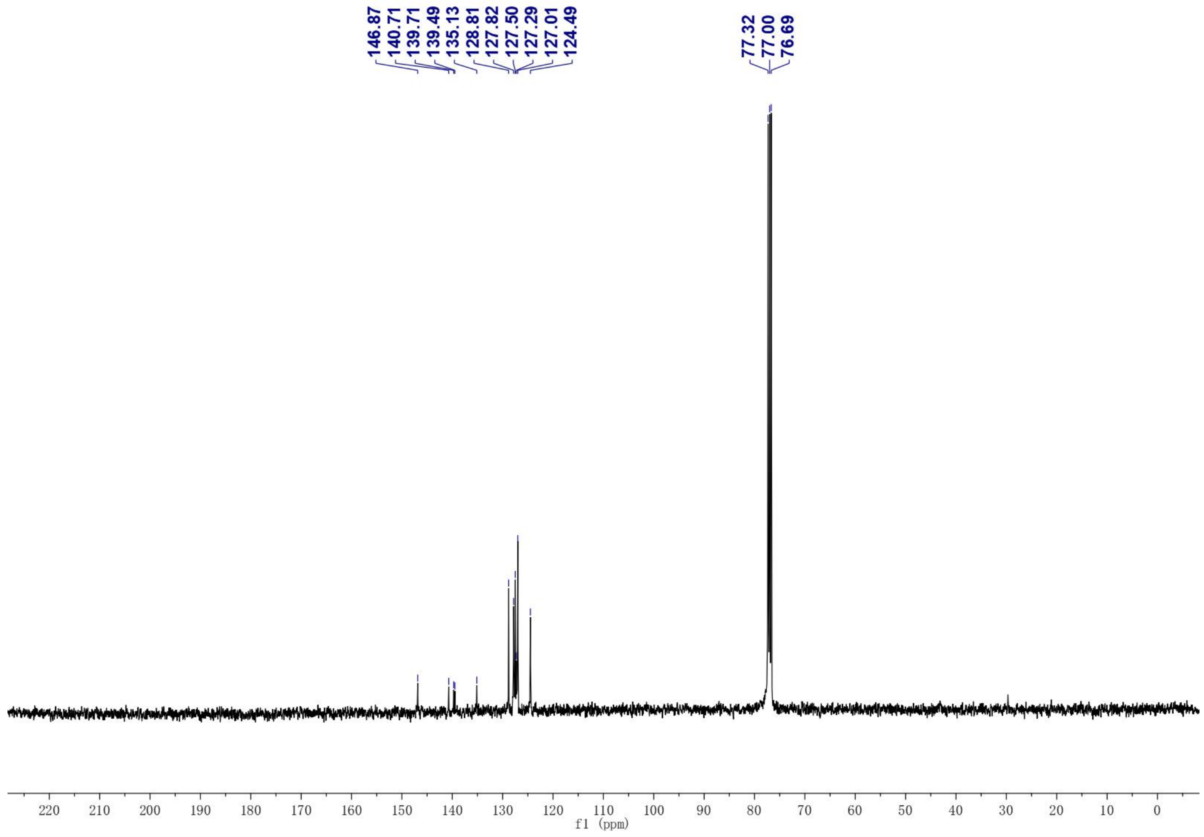

Supplementary Figure 89. ^{13}C NMR Spectrum of **13**

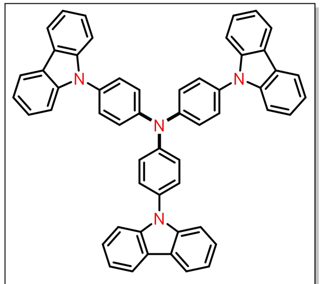

15

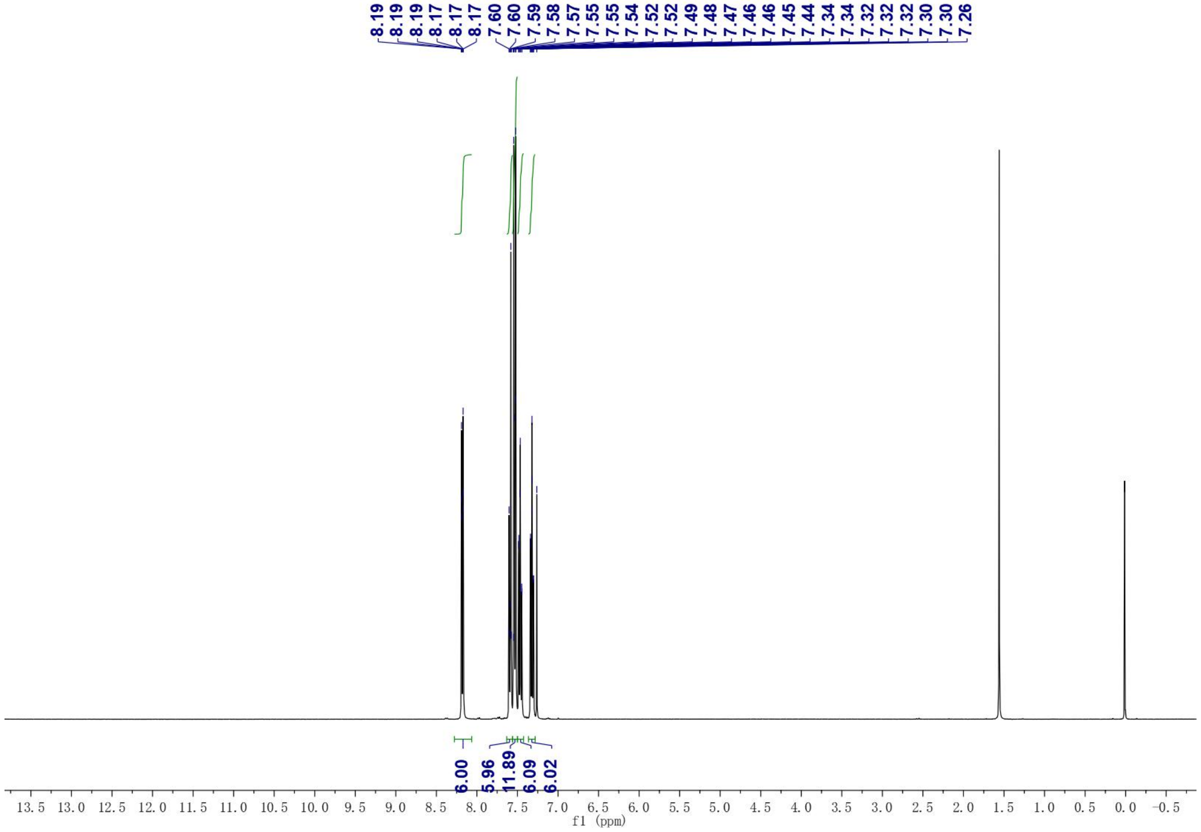

Supplementary Figure 90. ^{1}H NMR Spectrum of **15**

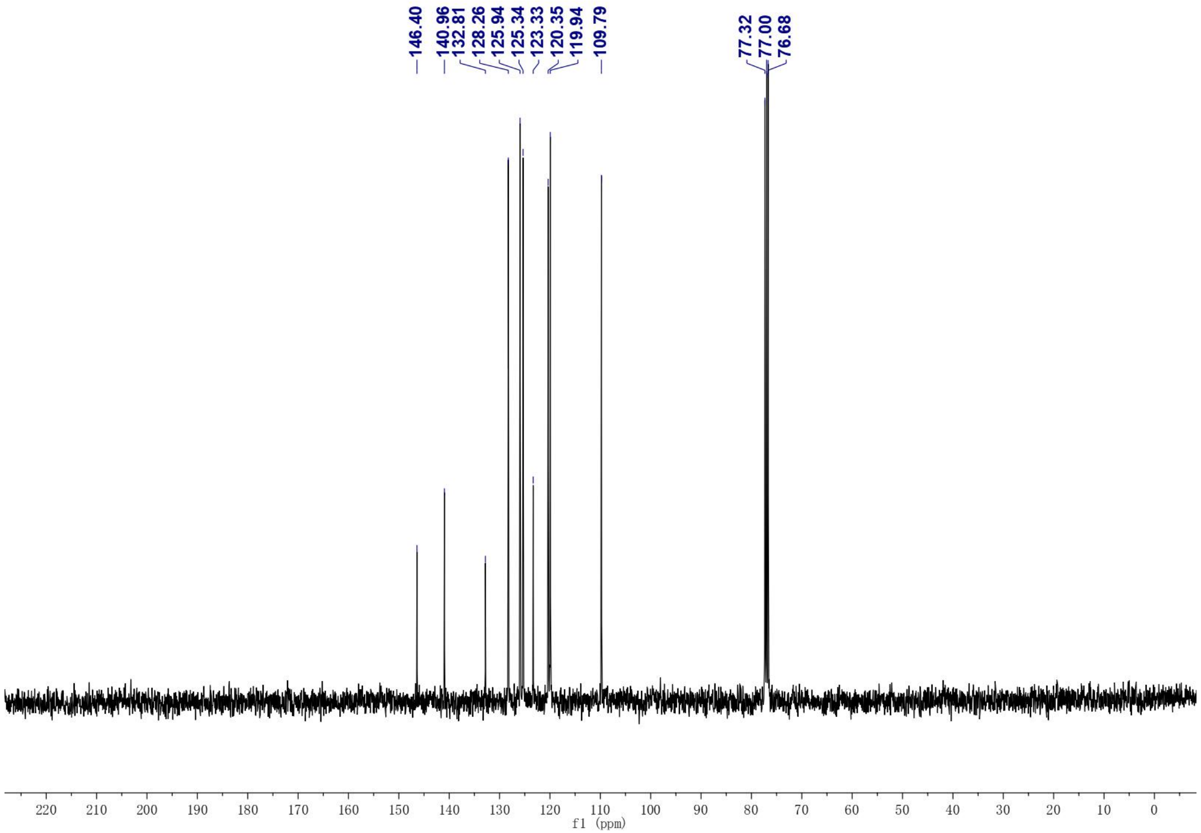

Supplementary Figure 91. ^{13}C NMR Spectrum of **15**

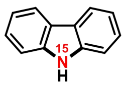

^{15}N-5a

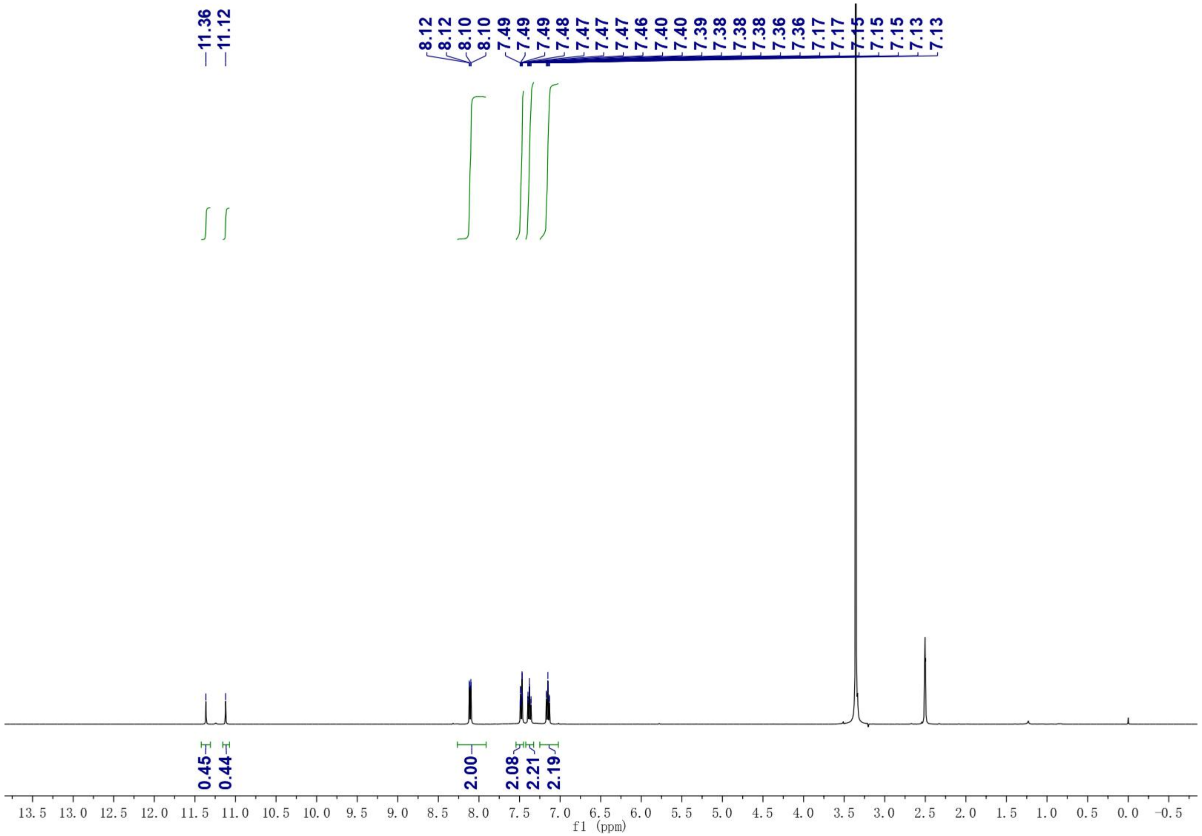

Supplementary Figure 92. ^{1}H NMR Spectrum of **^{15}N-5a**

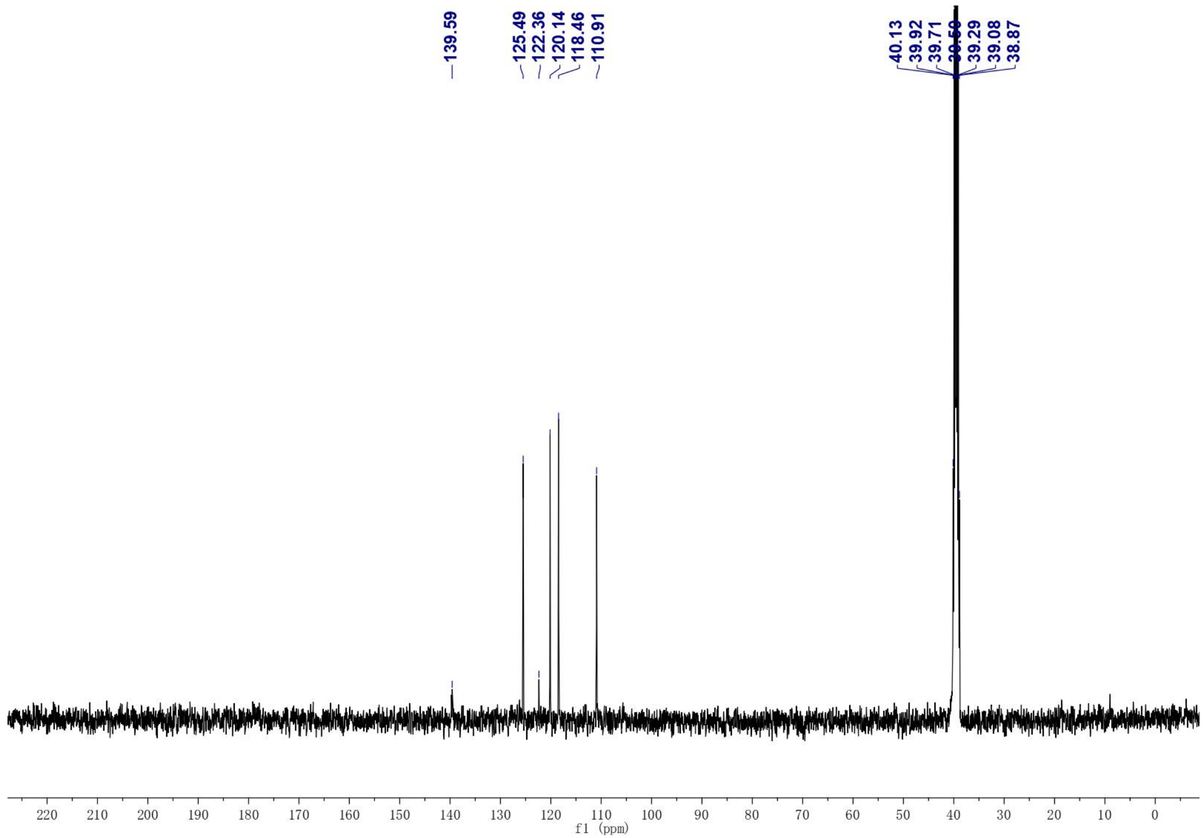

Supplementary Figure 93. ^{13}C NMR Spectrum of **^{15}N-5a**

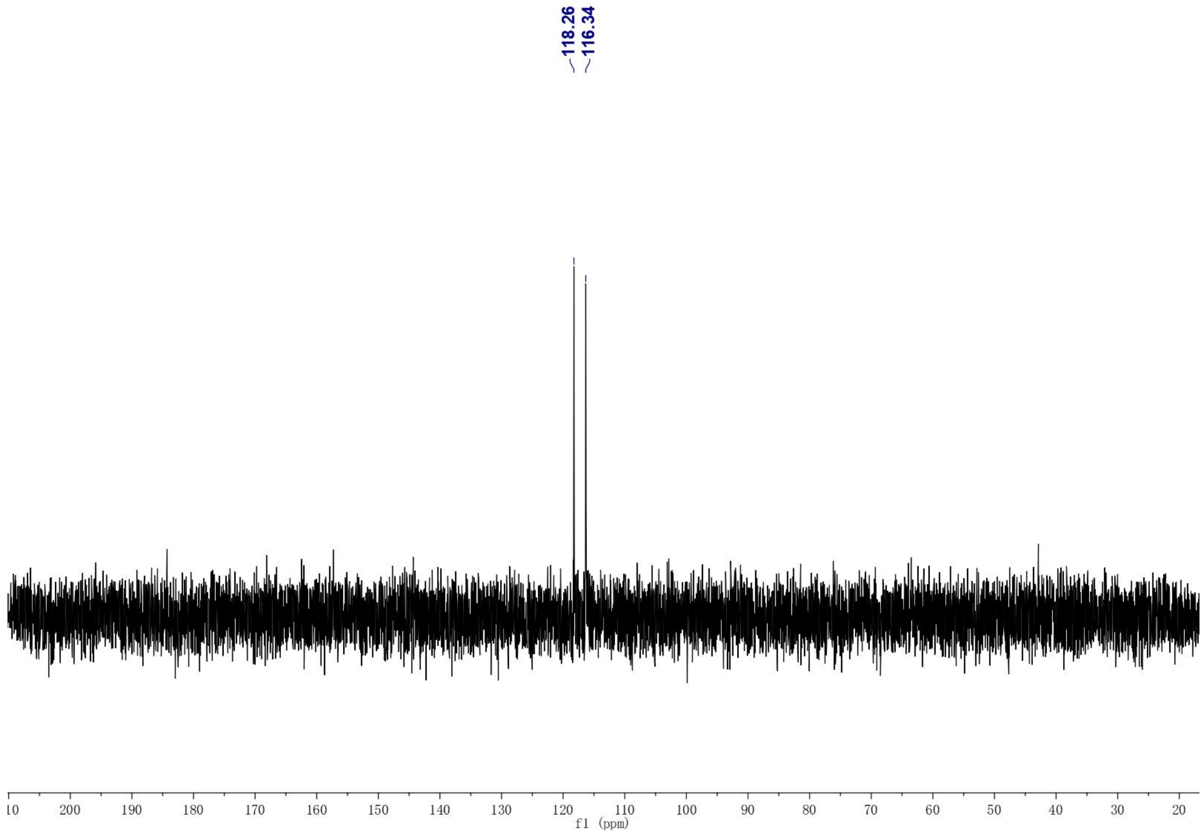

Supplementary Figure 94. ^{15}N NMR Spectrum of ^{15}**N-5a**

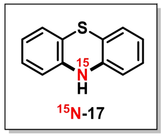

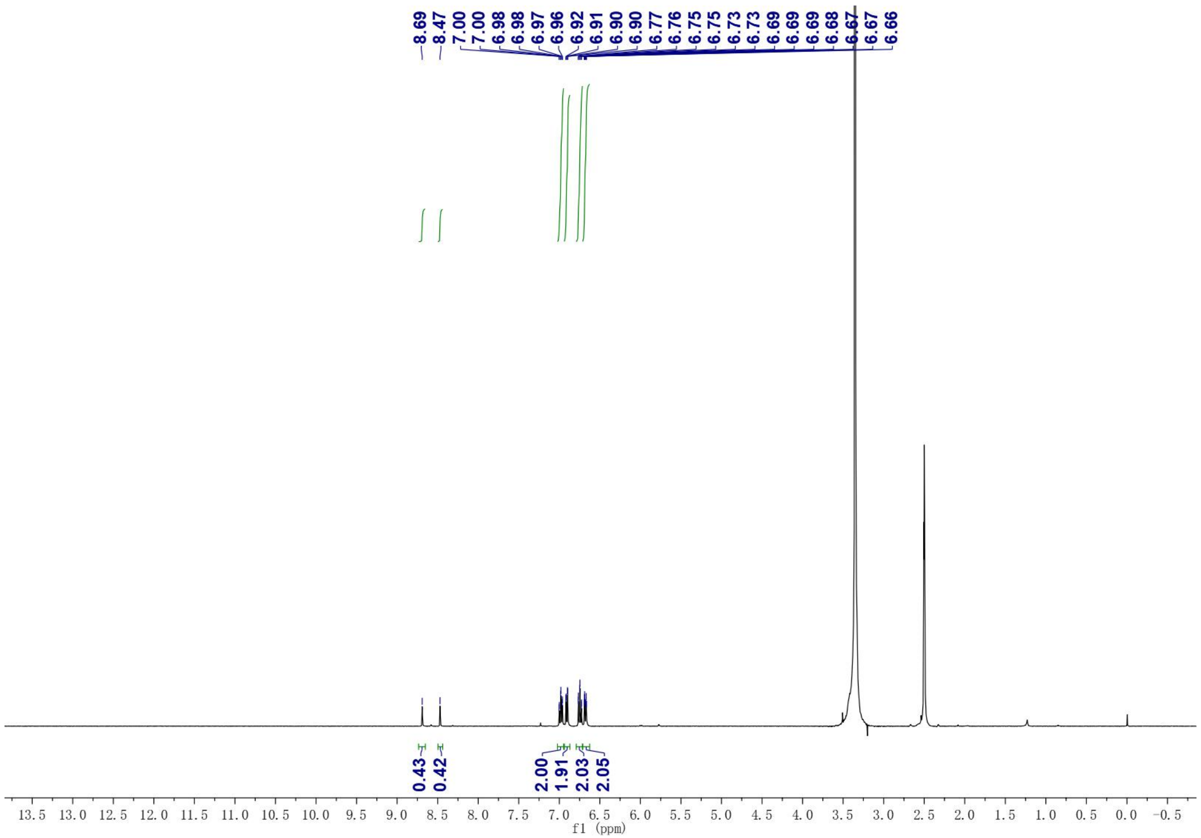

Supplementary Figure 95. ^{1}H NMR Spectrum of ^{15}**N-17**

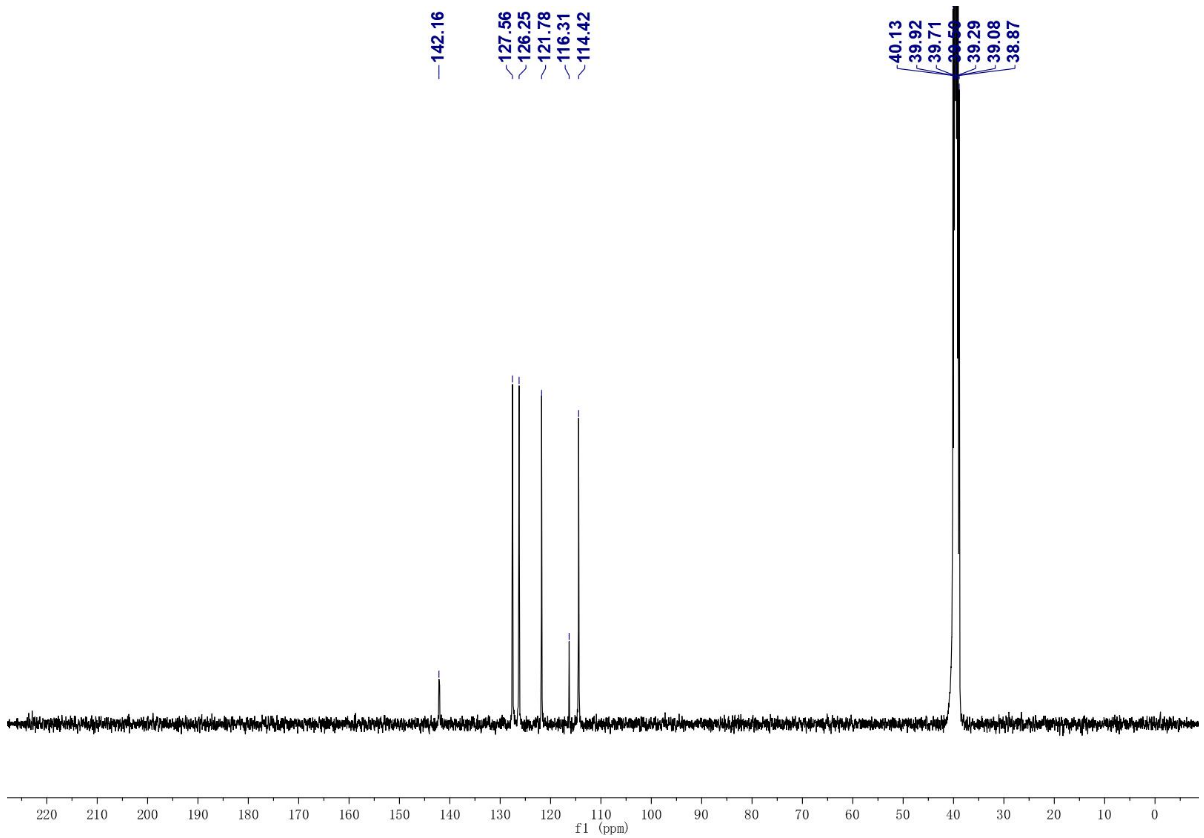

Supplementary Figure 96. ^{13}C NMR Spectrum of **^{15}N-17**

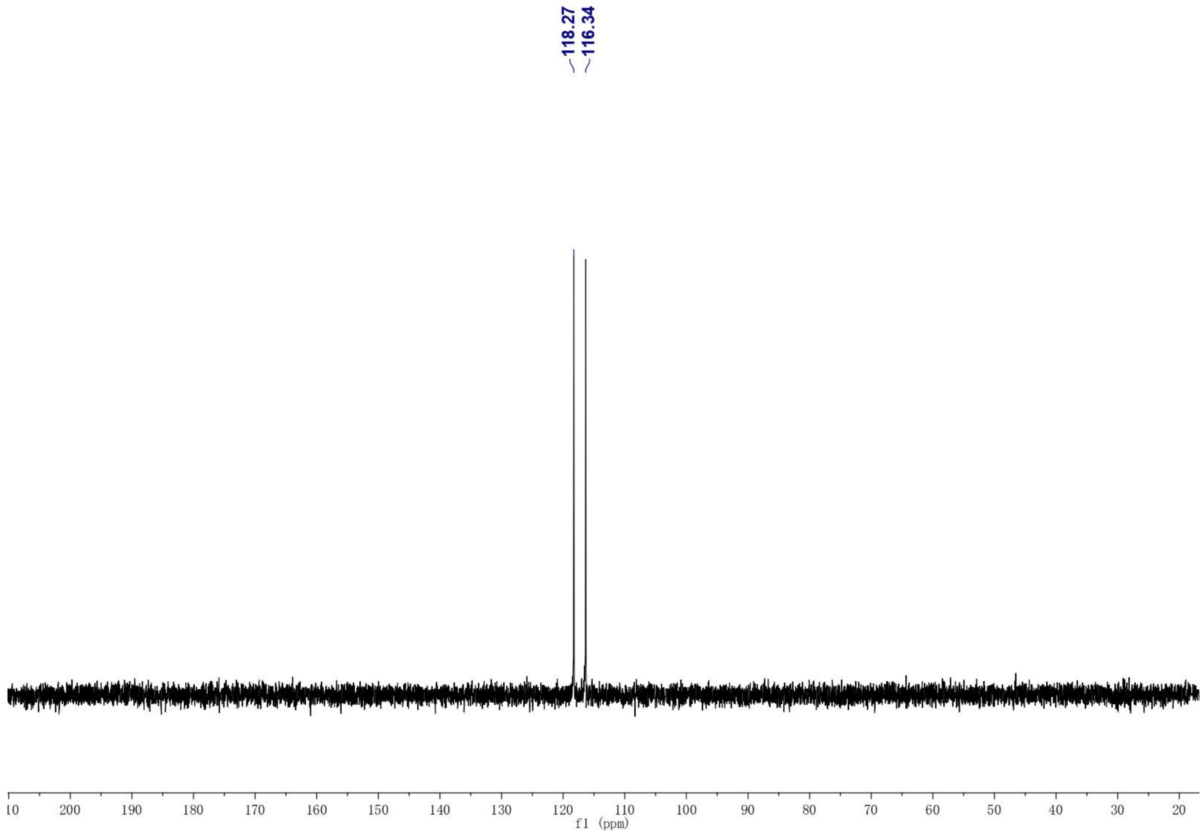

Supplementary Figure 97. ^{15}N NMR Spectrum of **^{15}N-17**

(VI) IR Spectral Data for Products

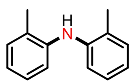

2a

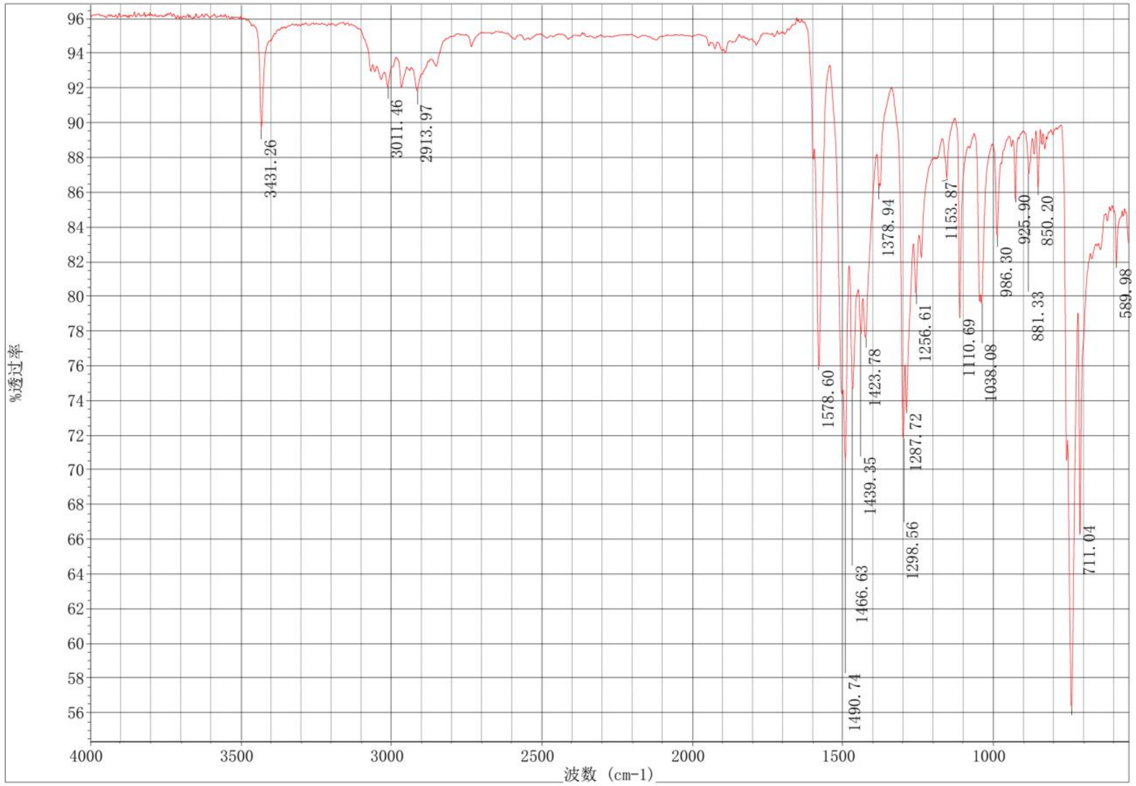

Supplementary Figure 98. IR Spectrum of **2a**

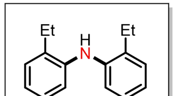

2b

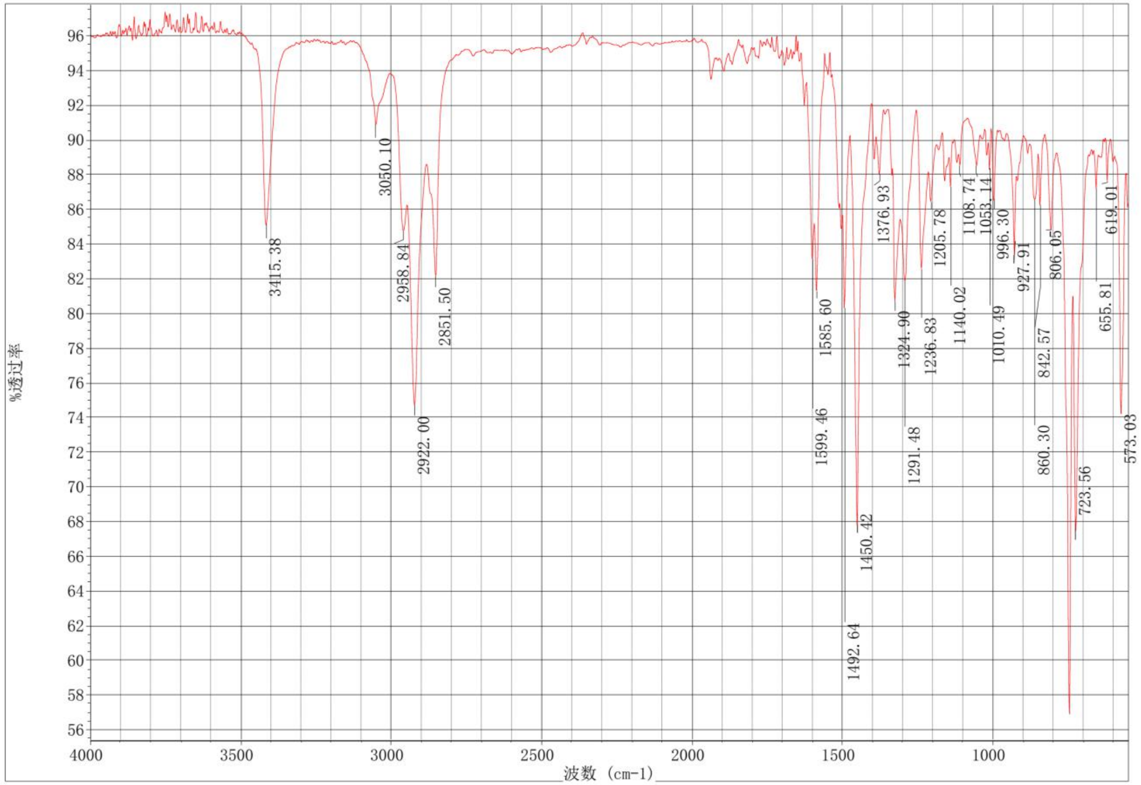

Supplementary Figure 99. IR Spectrum of **2b**

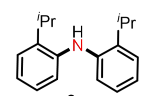

2c

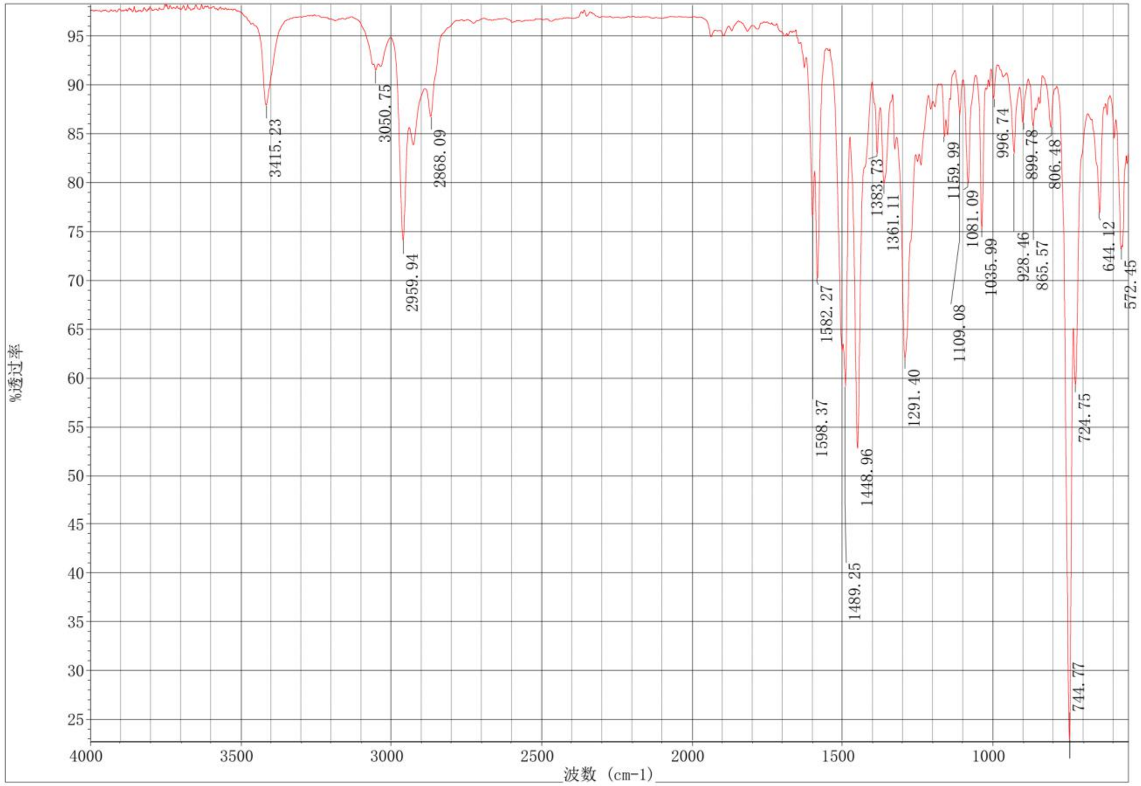

Supplementary Figure 100. IR Spectrum of **2c**

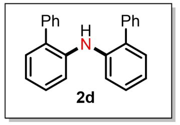

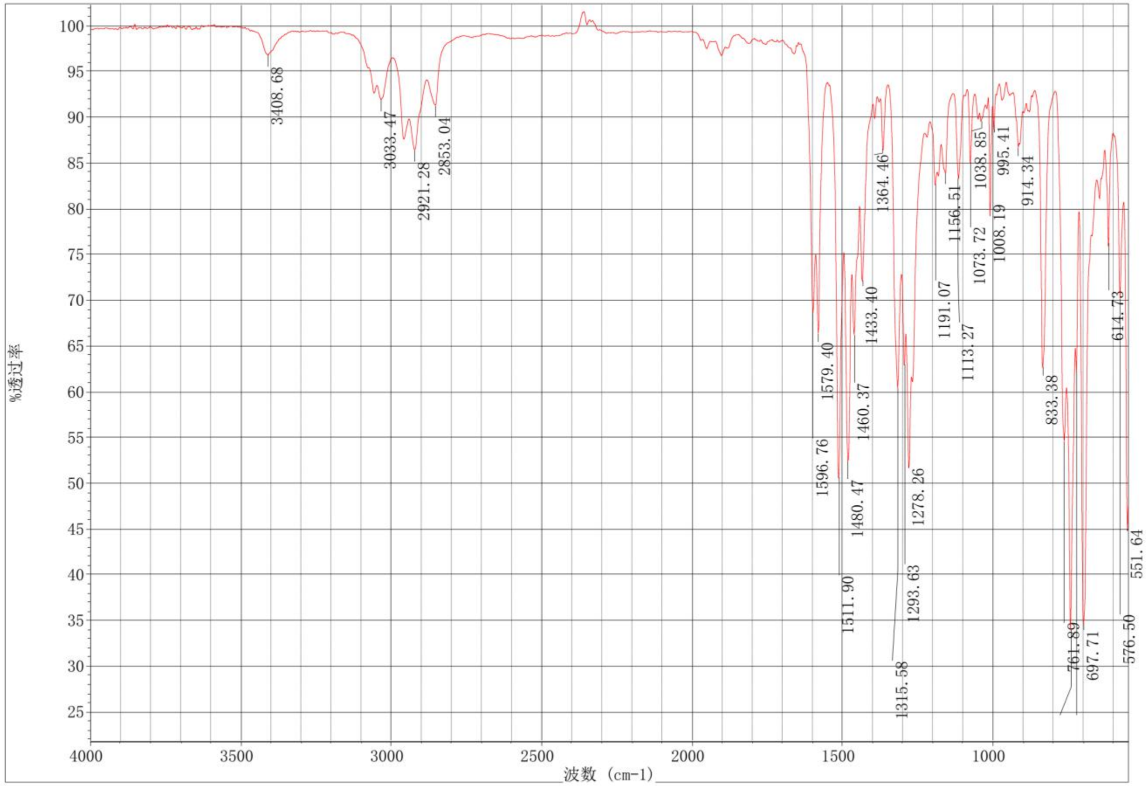

Supplementary Figure 101. IR Spectrum of **2d**

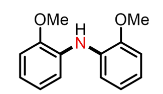

2e

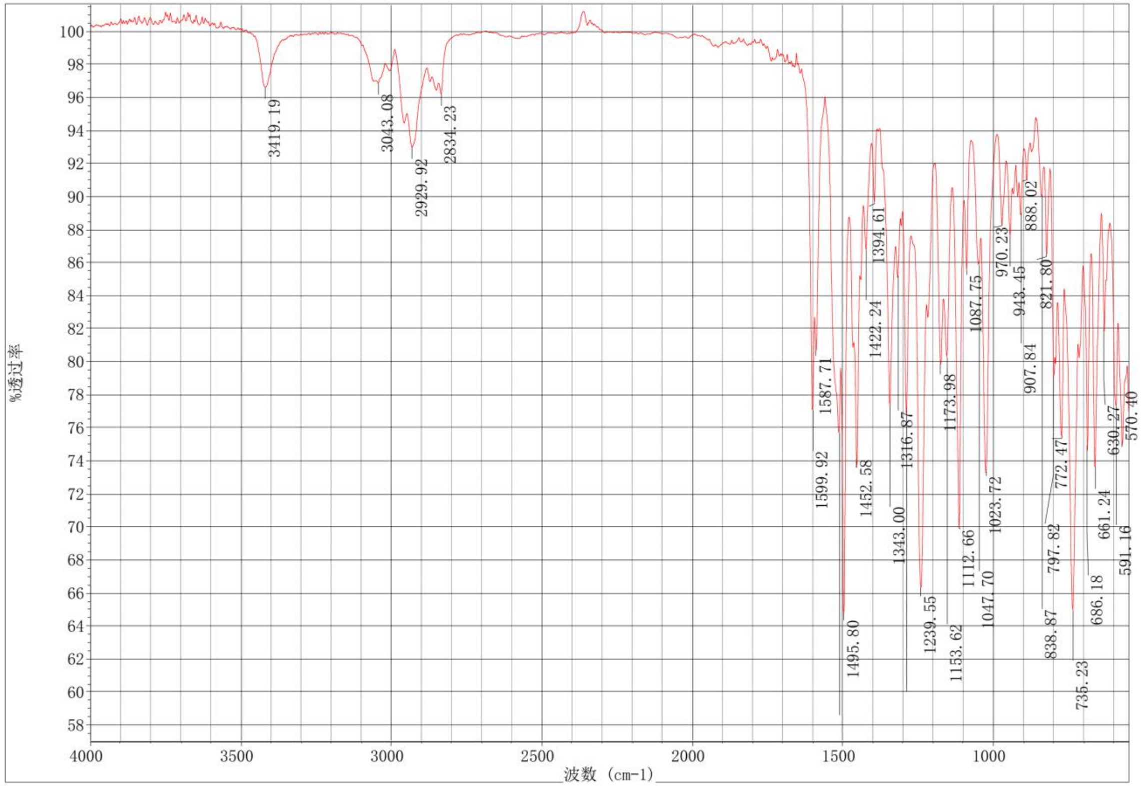

Supplementary Figure 102. IR Spectrum of **2e**

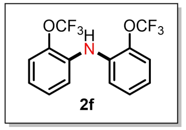

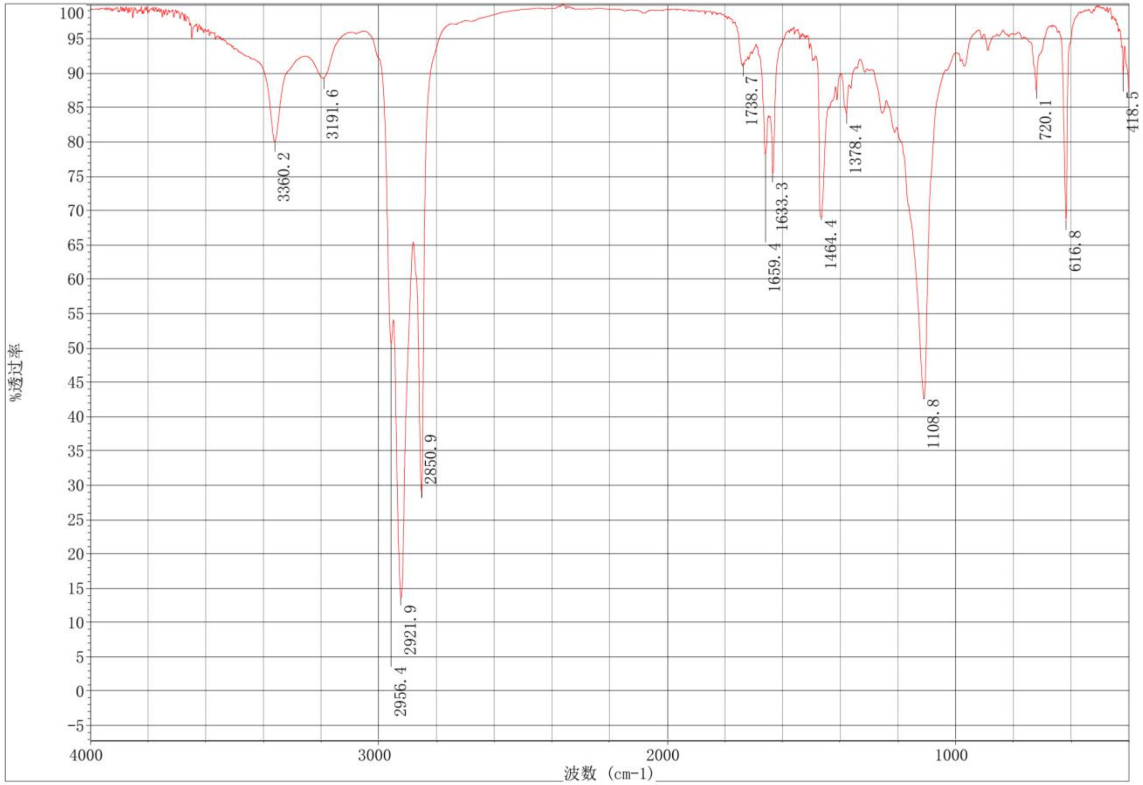

Supplementary Figure 103. IR Spectrum of **2f**

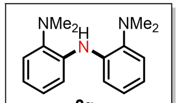

2g

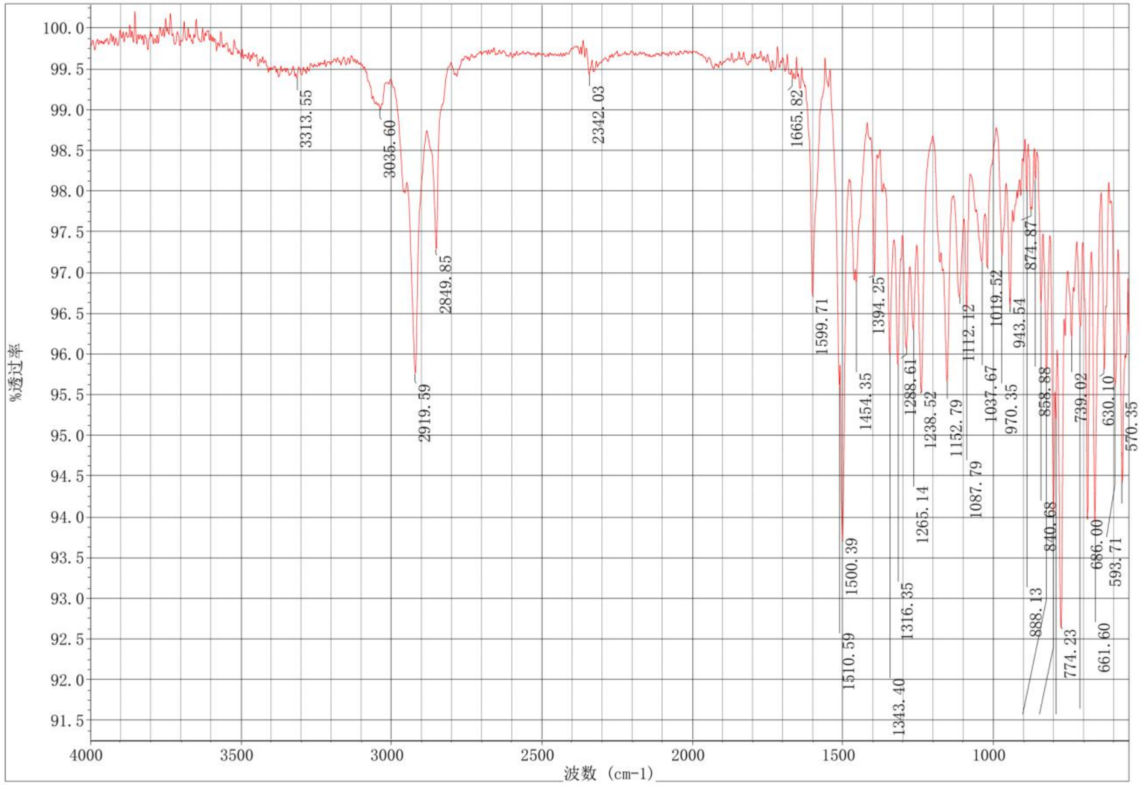

Supplementary Figure 104. IR Spectrum of **2g**

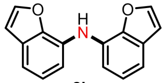

2h

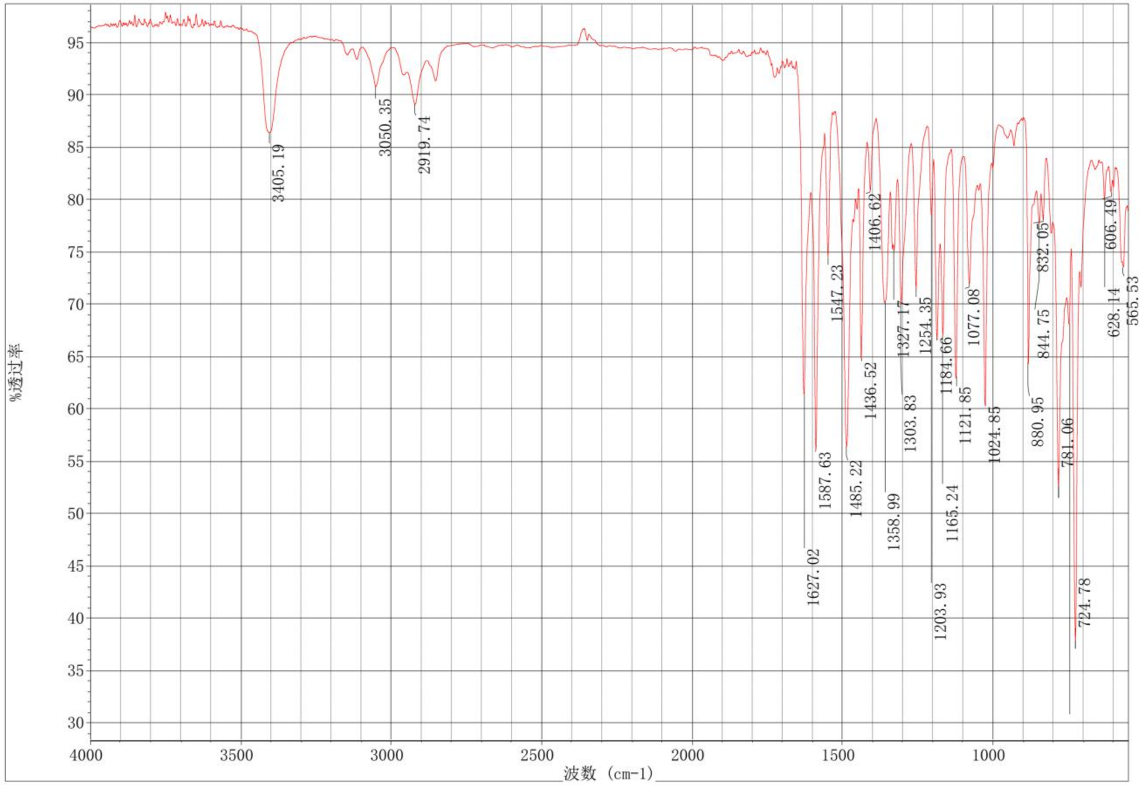

Supplementary Figure 105. IR Spectrum of **2h**

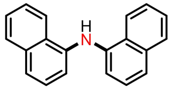

2i

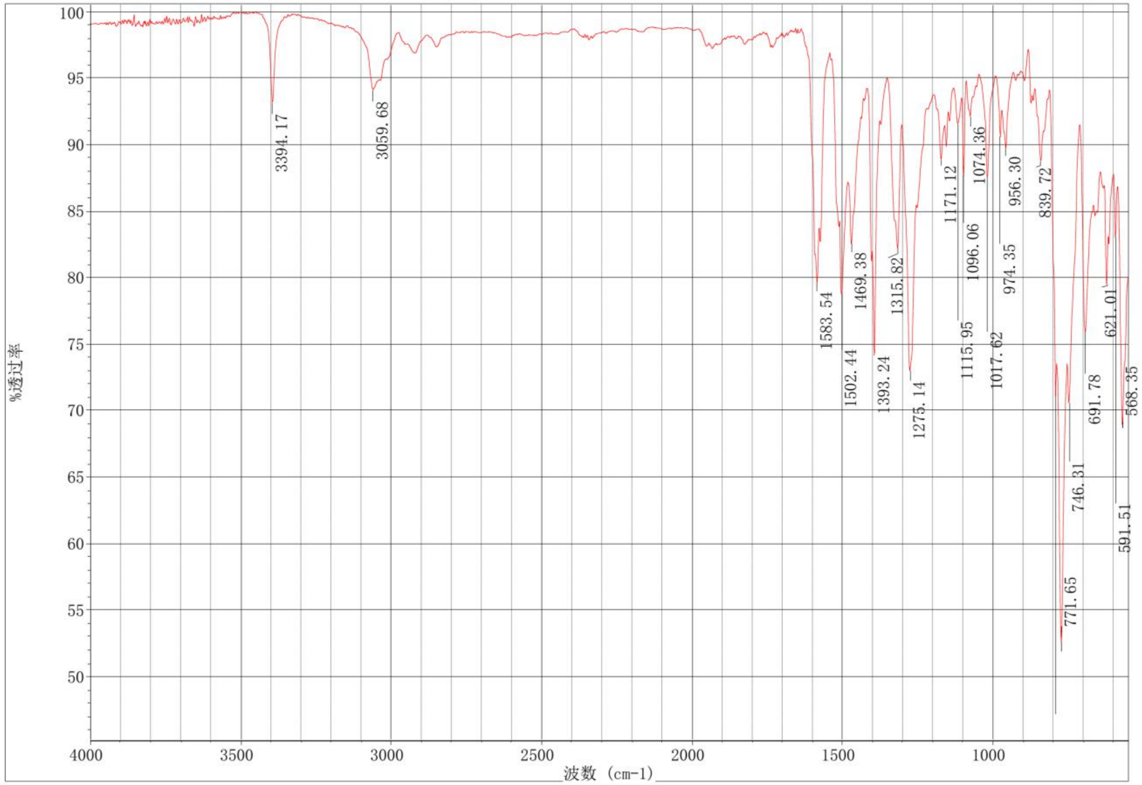

Supplementary Figure 106. IR Spectrum of **2i**

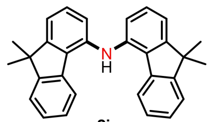

2j

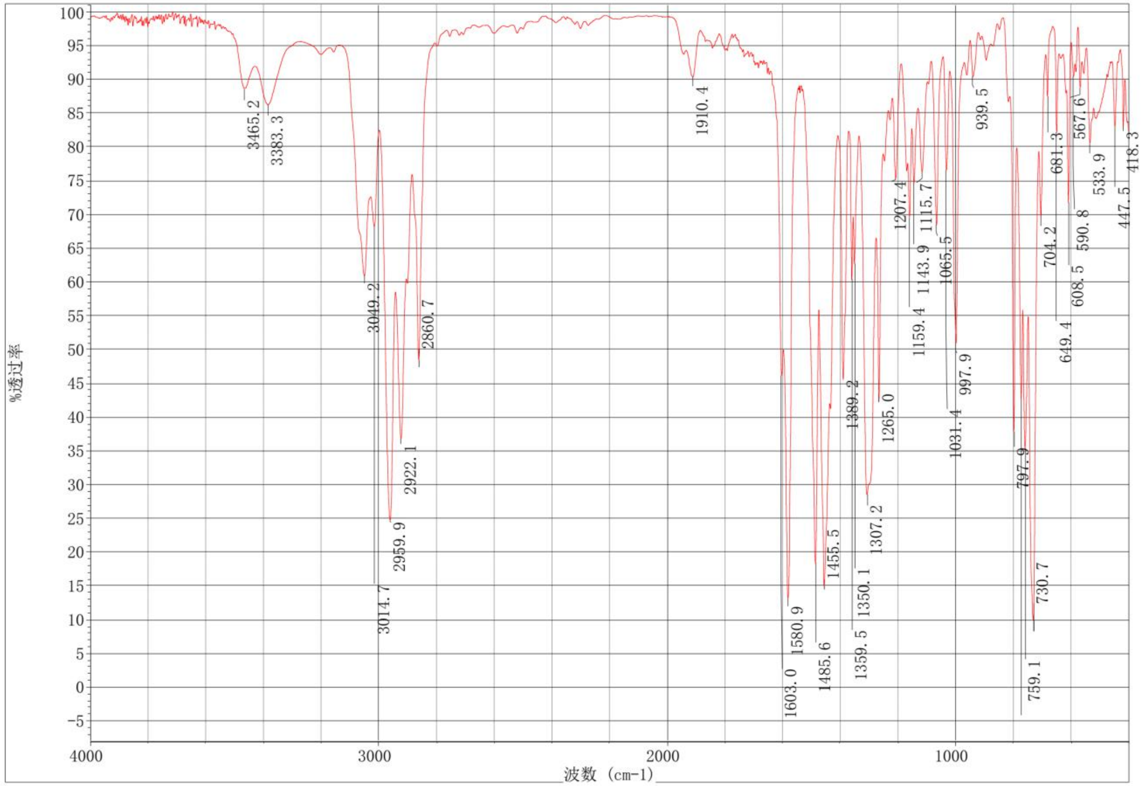

Supplementary Figure 107. IR Spectrum of **2j**

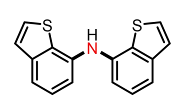

2k

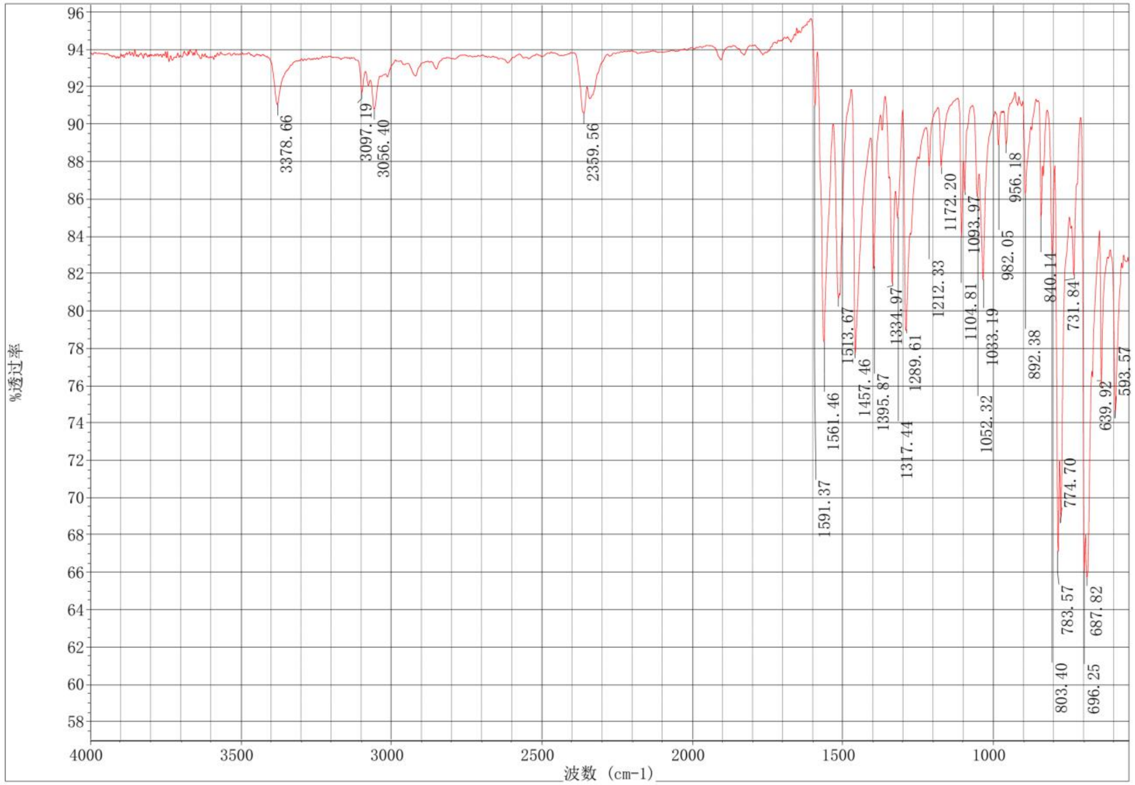

Supplementary Figure 108. IR Spectrum of **2k**

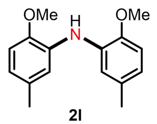

2l

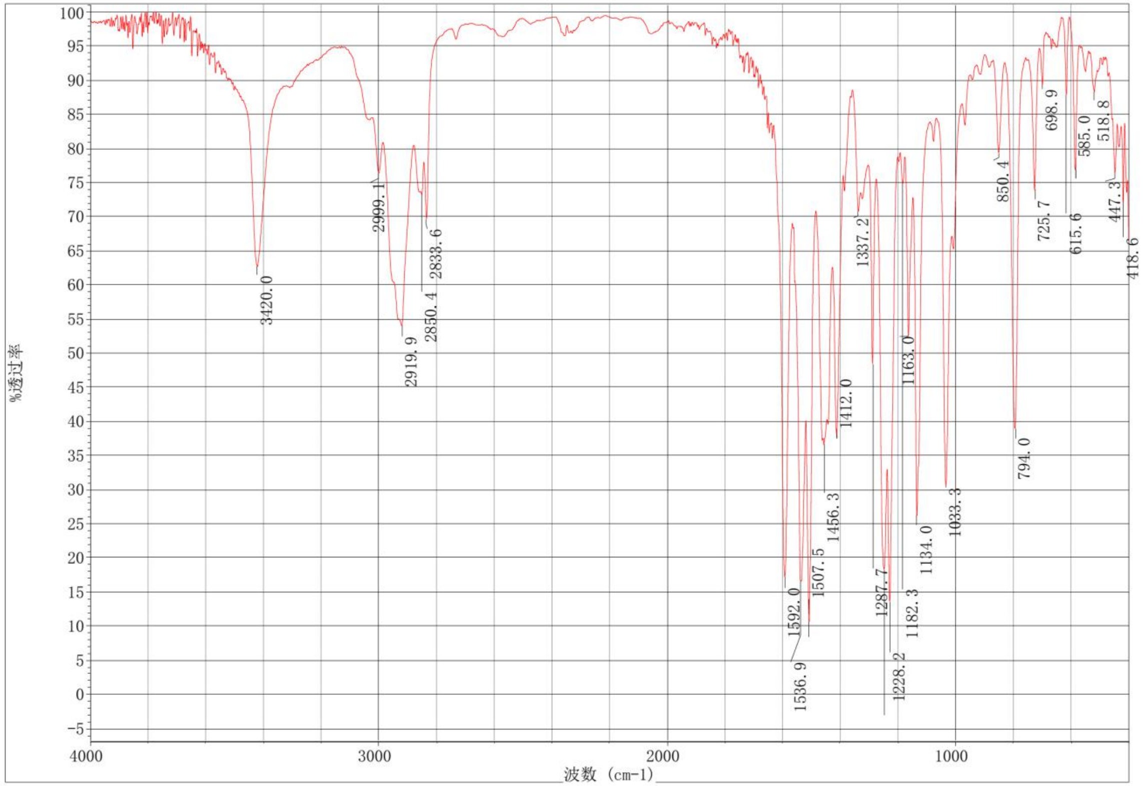

Supplementary Figure 109. IR Spectrum of **2l**

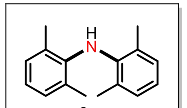

2m

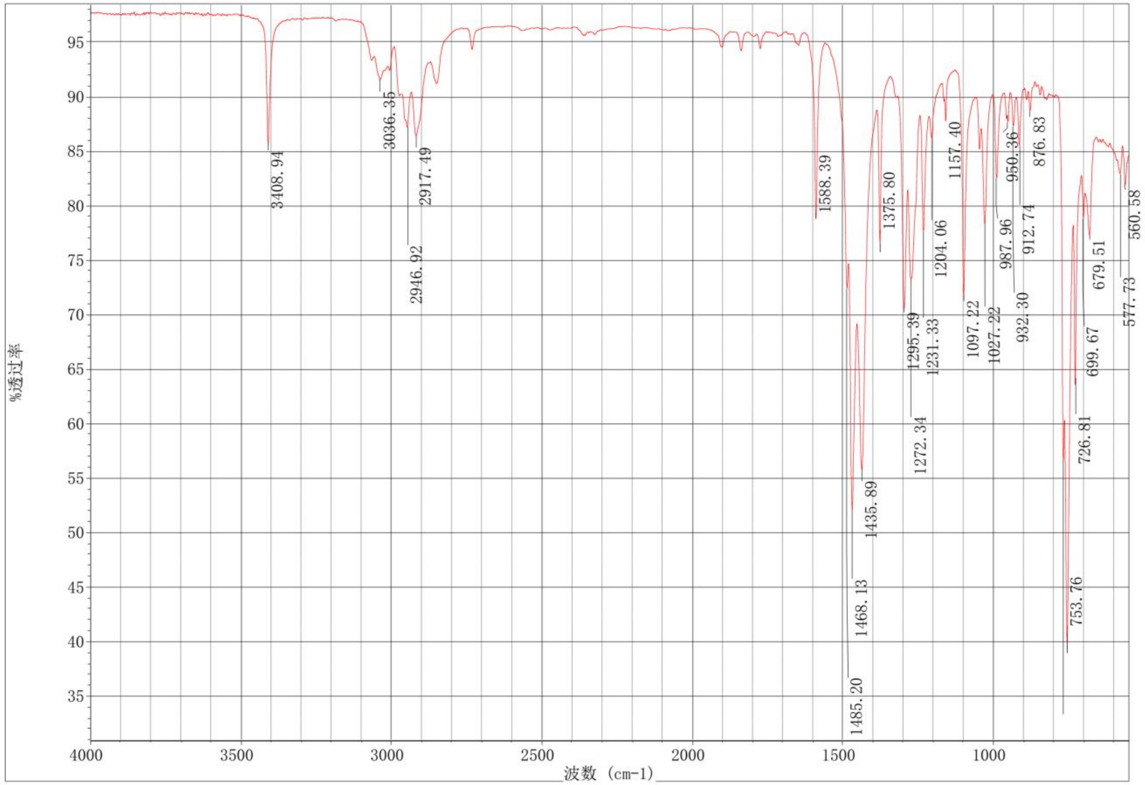

Supplementary Figure 110. IR Spectrum of **2m**

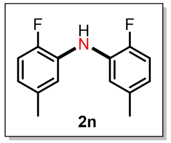

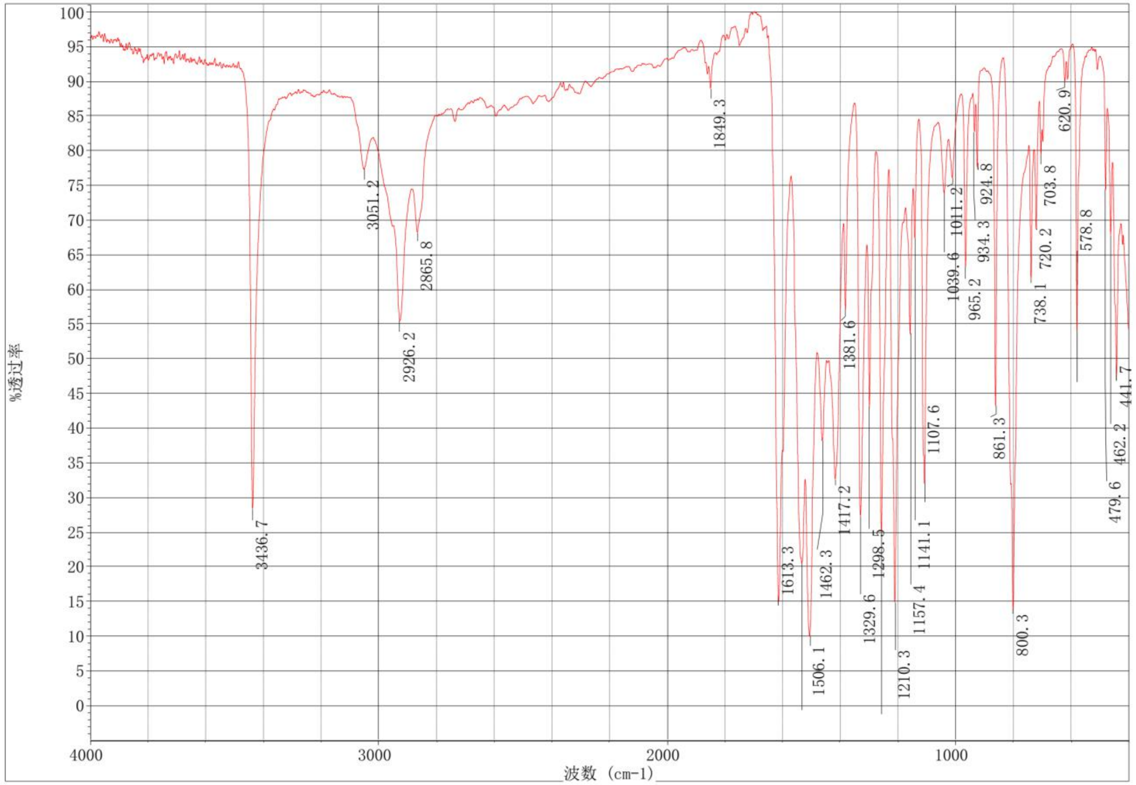

Supplementary Figure 111. IR Spectrum of **2n**

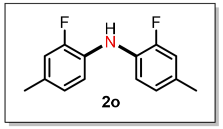

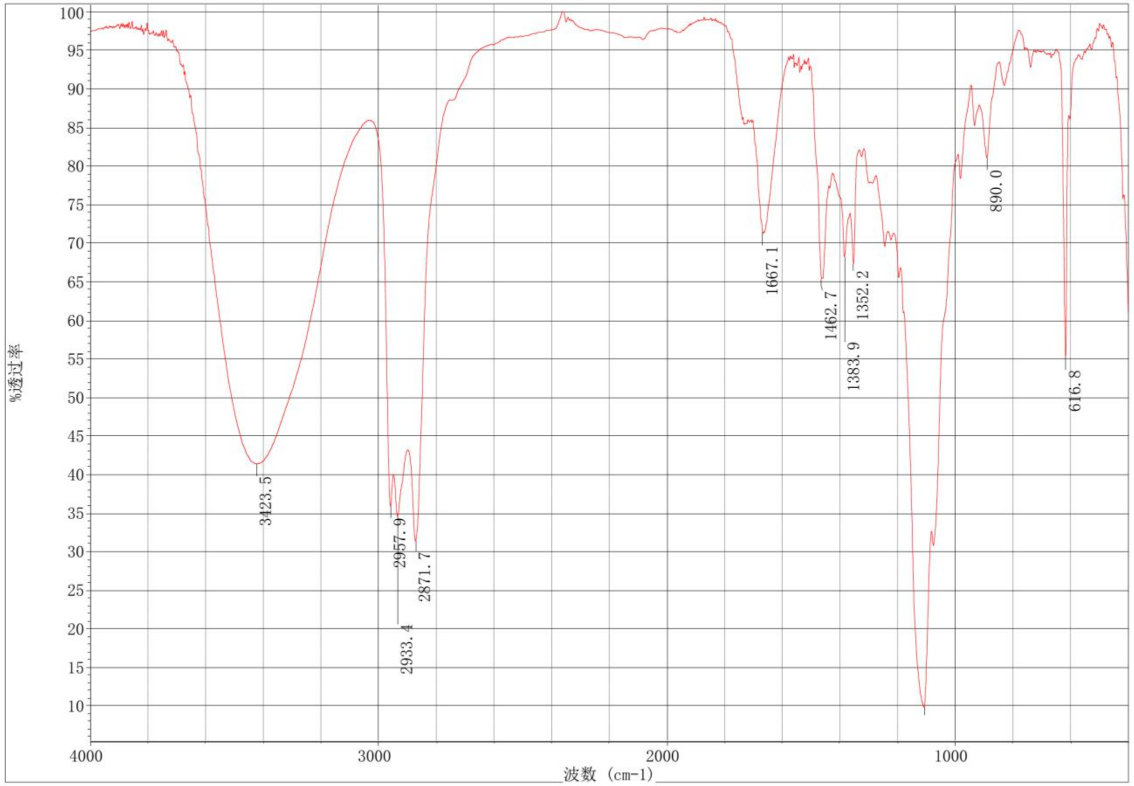

Supplementary Figure 112. IR Spectrum of **2o**

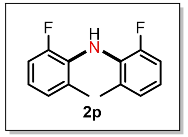

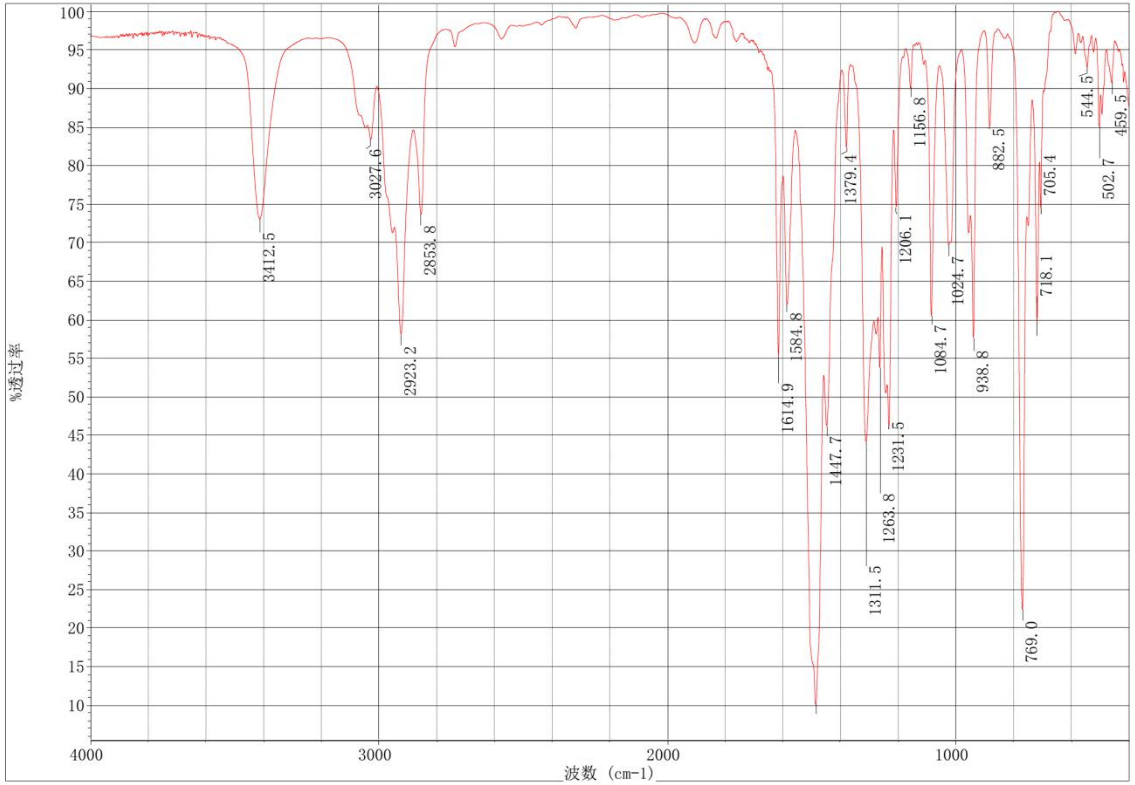

Supplementary Figure 113. IR Spectrum of **2p**

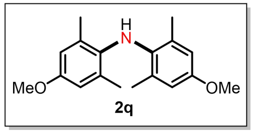

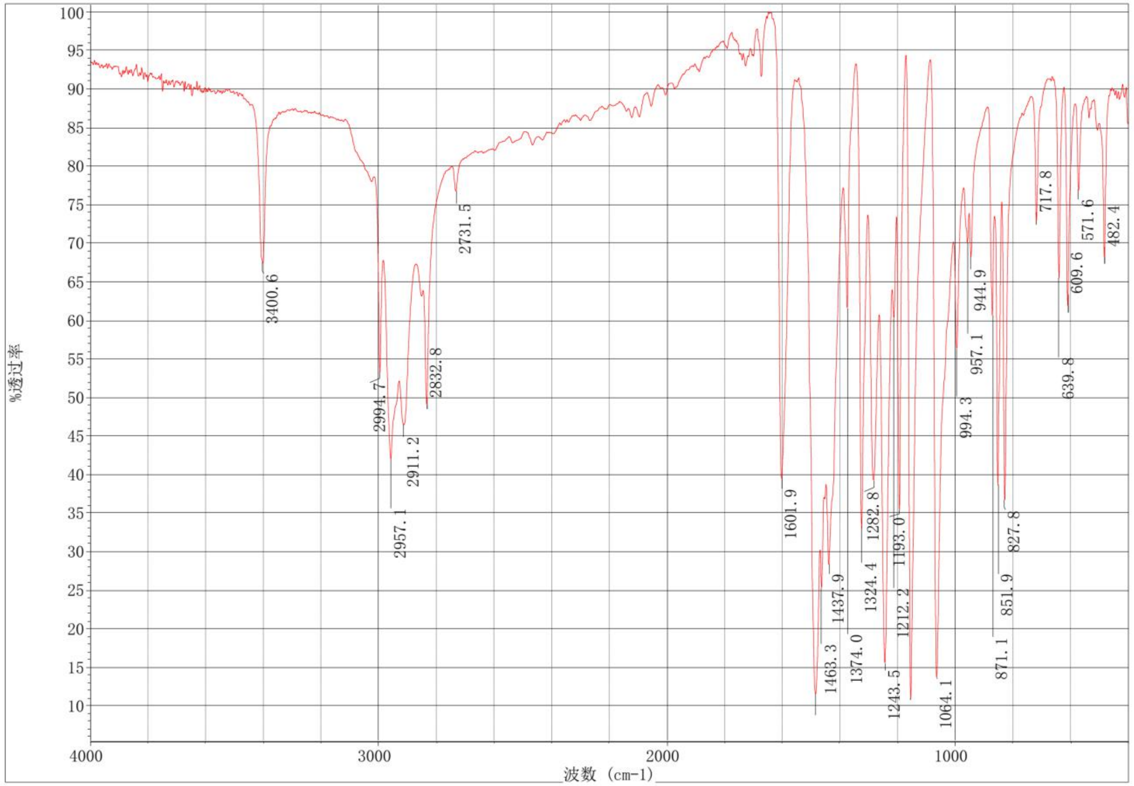

Supplementary Figure 114. IR Spectrum of **2q**

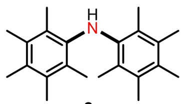

2r

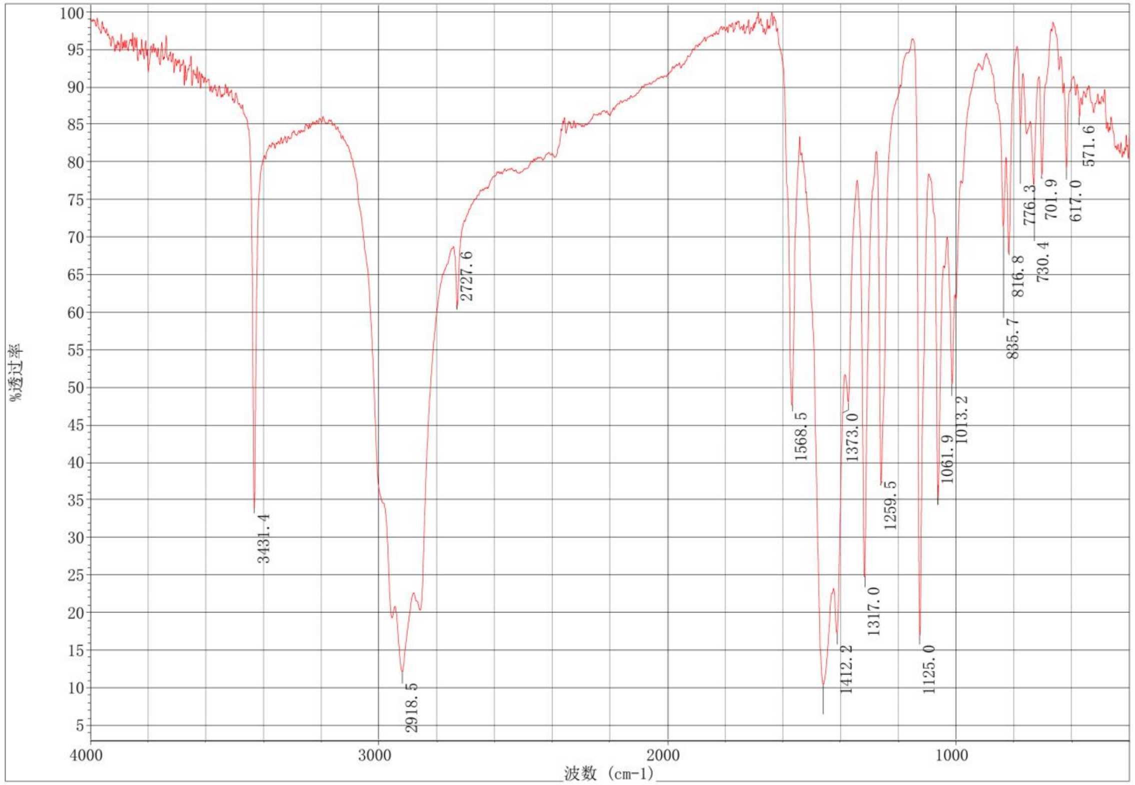

Supplementary Figure 115. IR Spectrum of **2r**

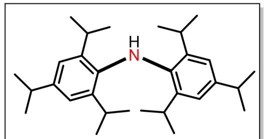

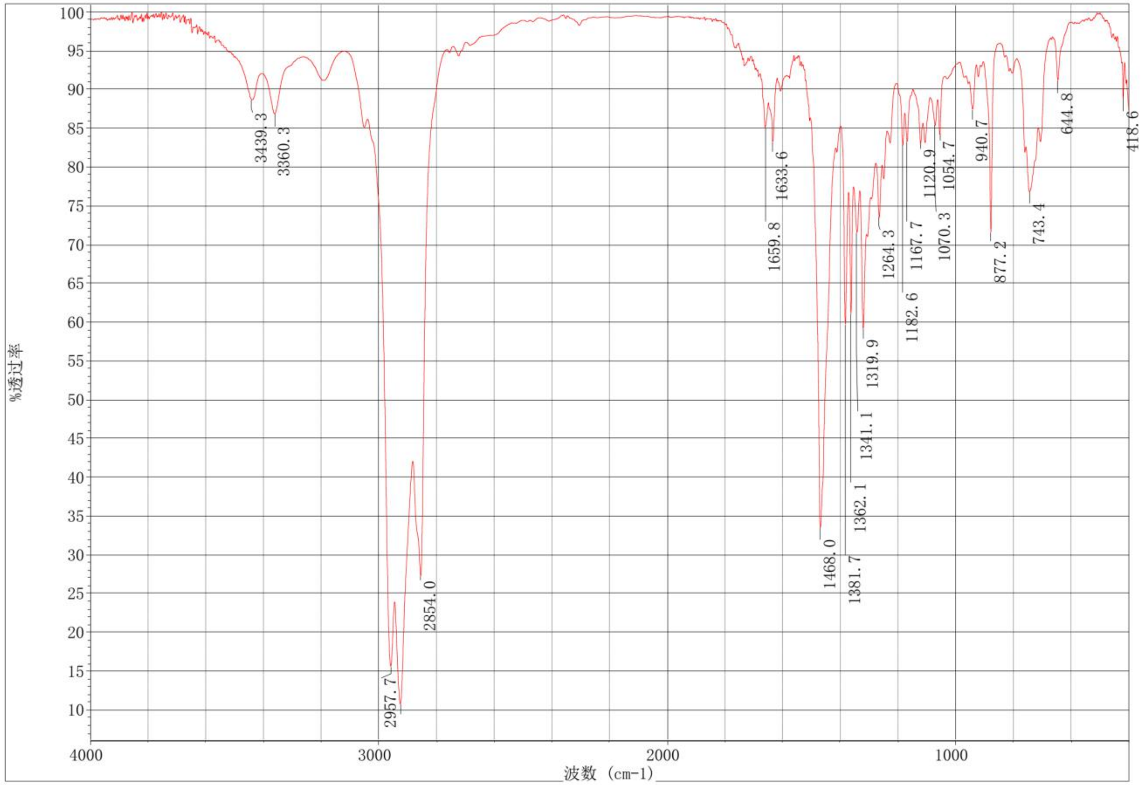

Supplementary Figure 116. IR Spectrum of **2s**

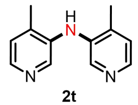

2t

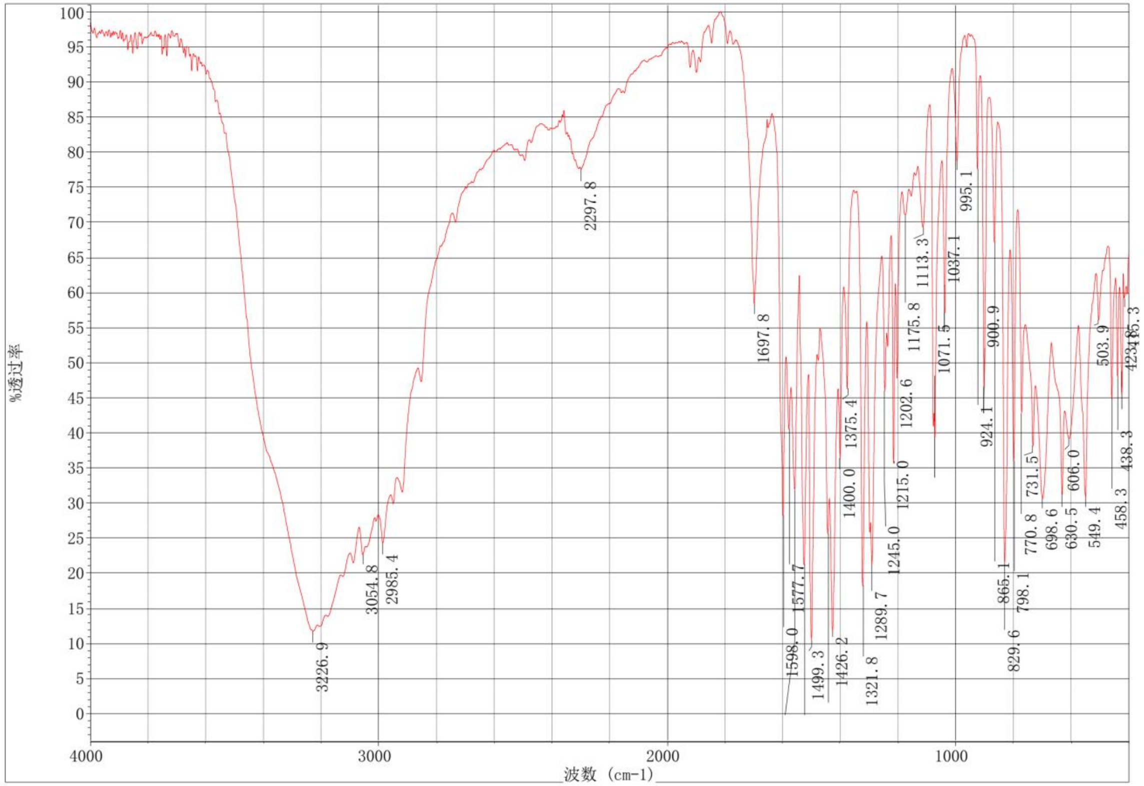

Supplementary Figure 117. IR Spectrum of **2t**

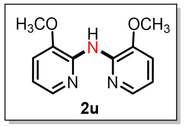

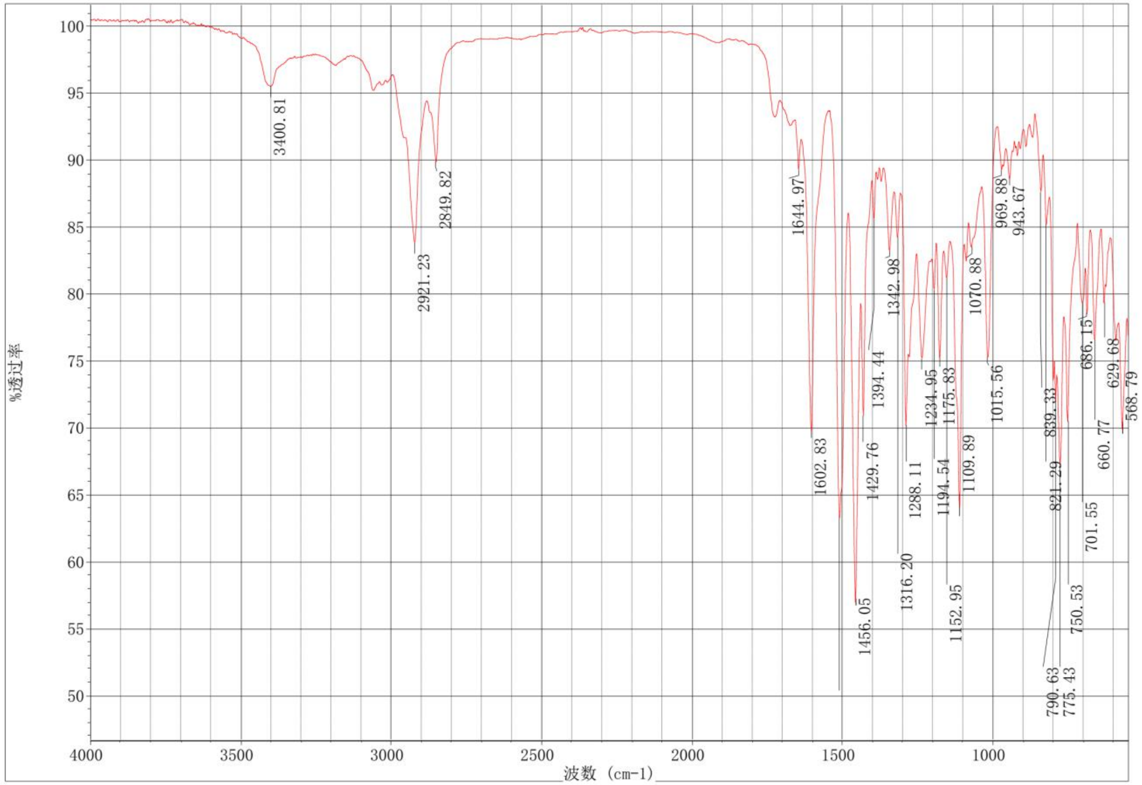

Supplementary Figure 118. IR Spectrum of **2u**

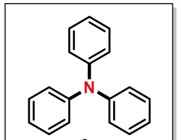

3a

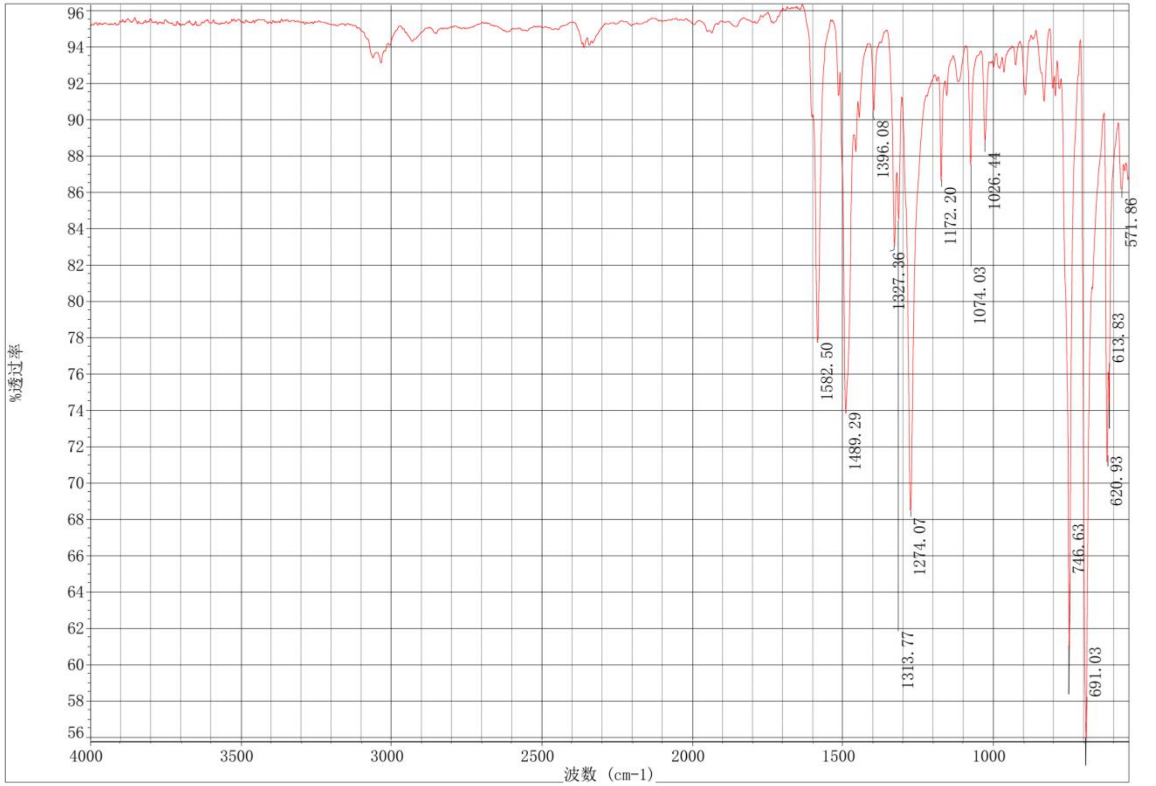

Supplementary Figure 119. IR Spectrum of **3a**

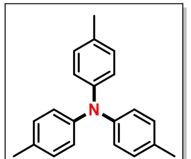

3b

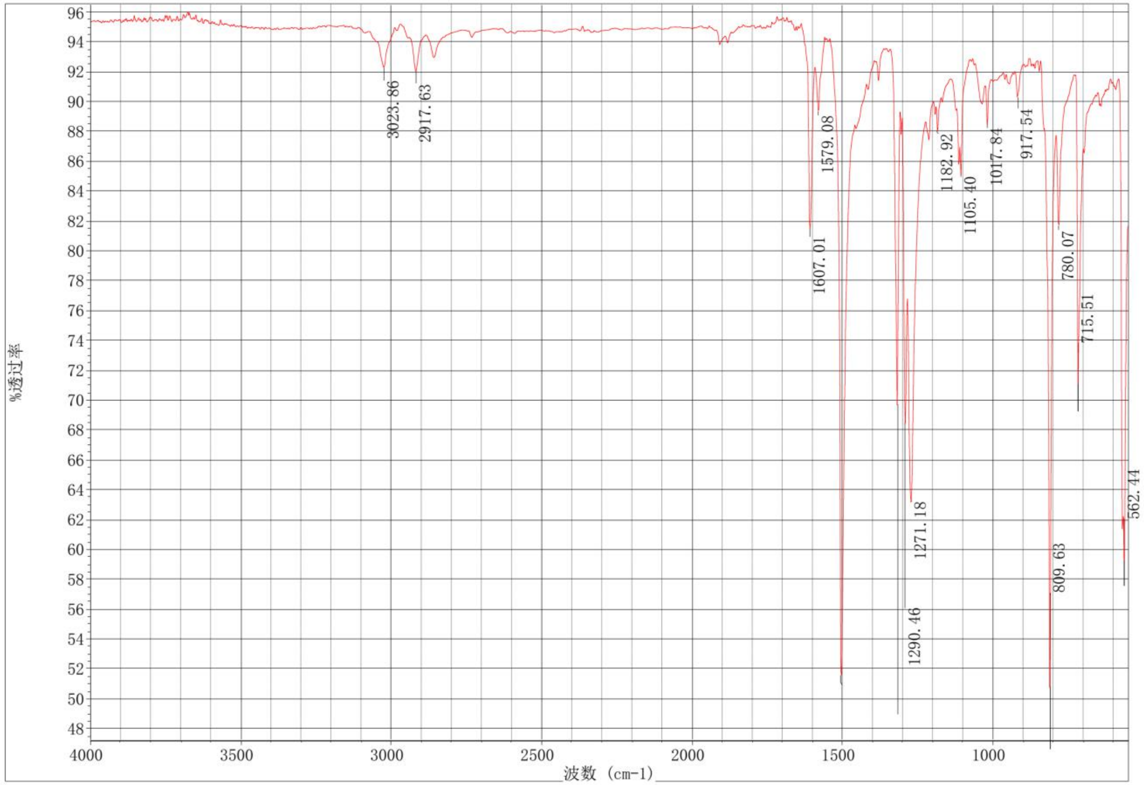

Supplementary Figure 120. IR Spectrum of **3b**

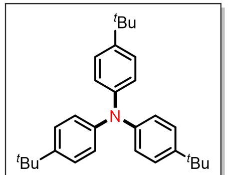

3c

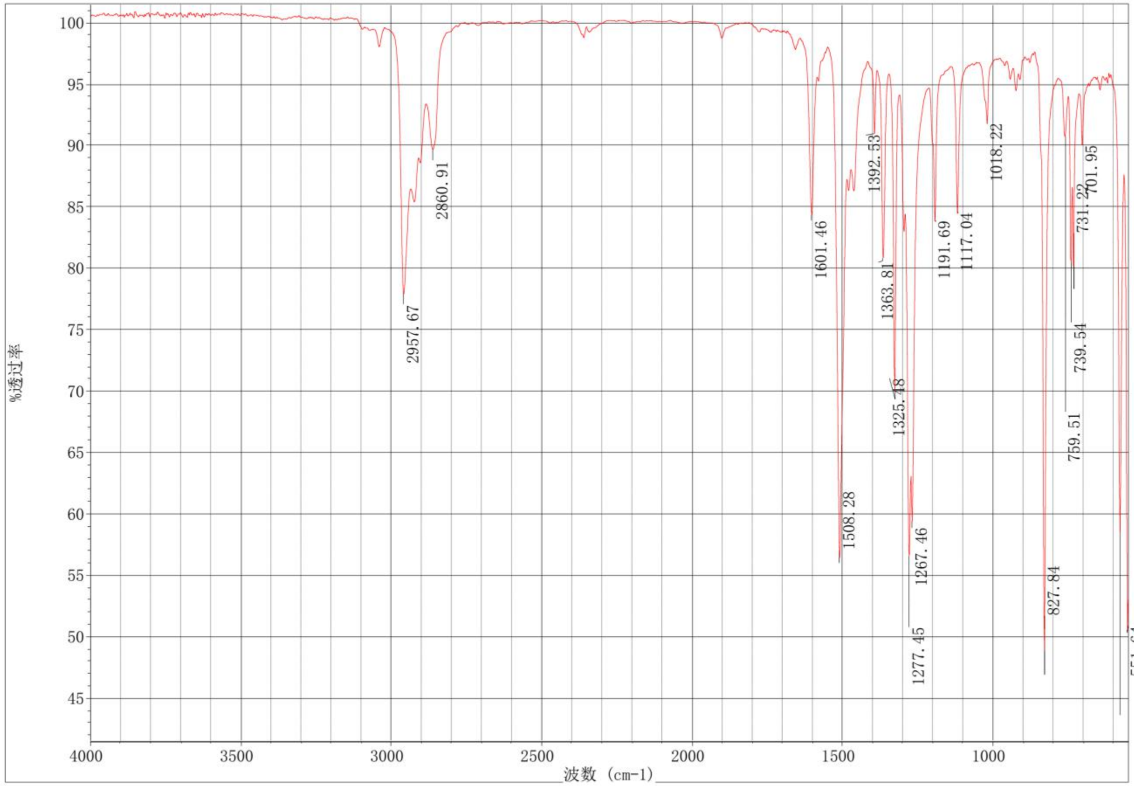

Supplementary Figure 121. IR Spectrum of **3c**

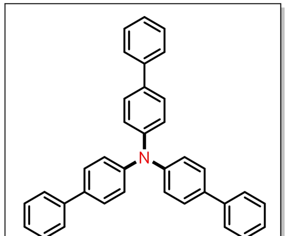

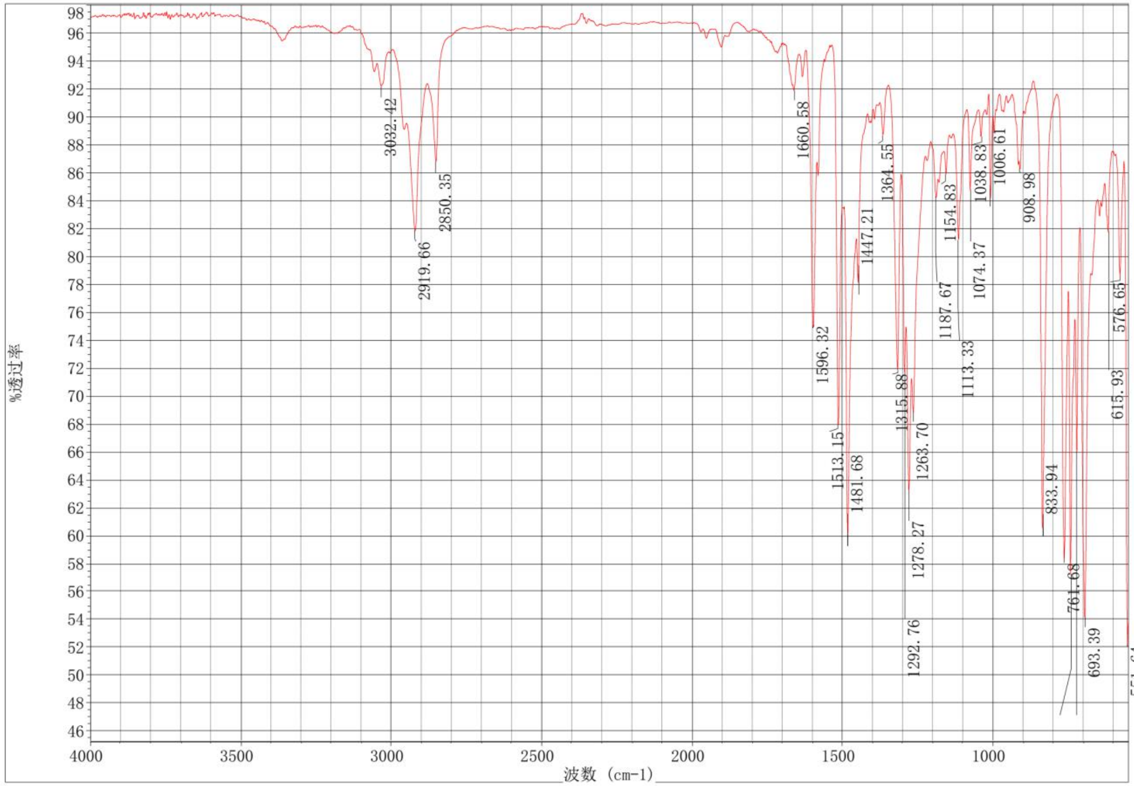

Supplementary Figure 122. IR Spectrum of **3d**

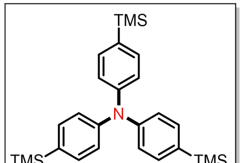

3e

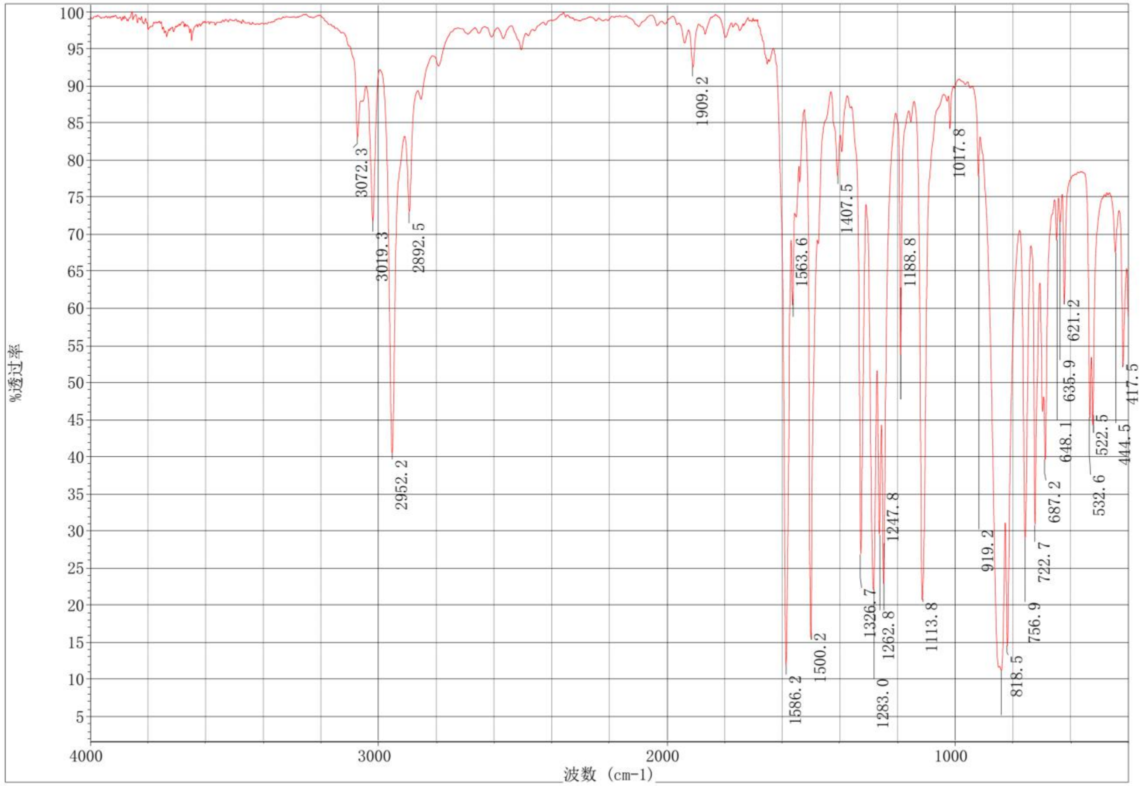

Supplementary Figure 123. IR Spectrum of **3e**

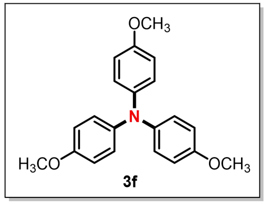

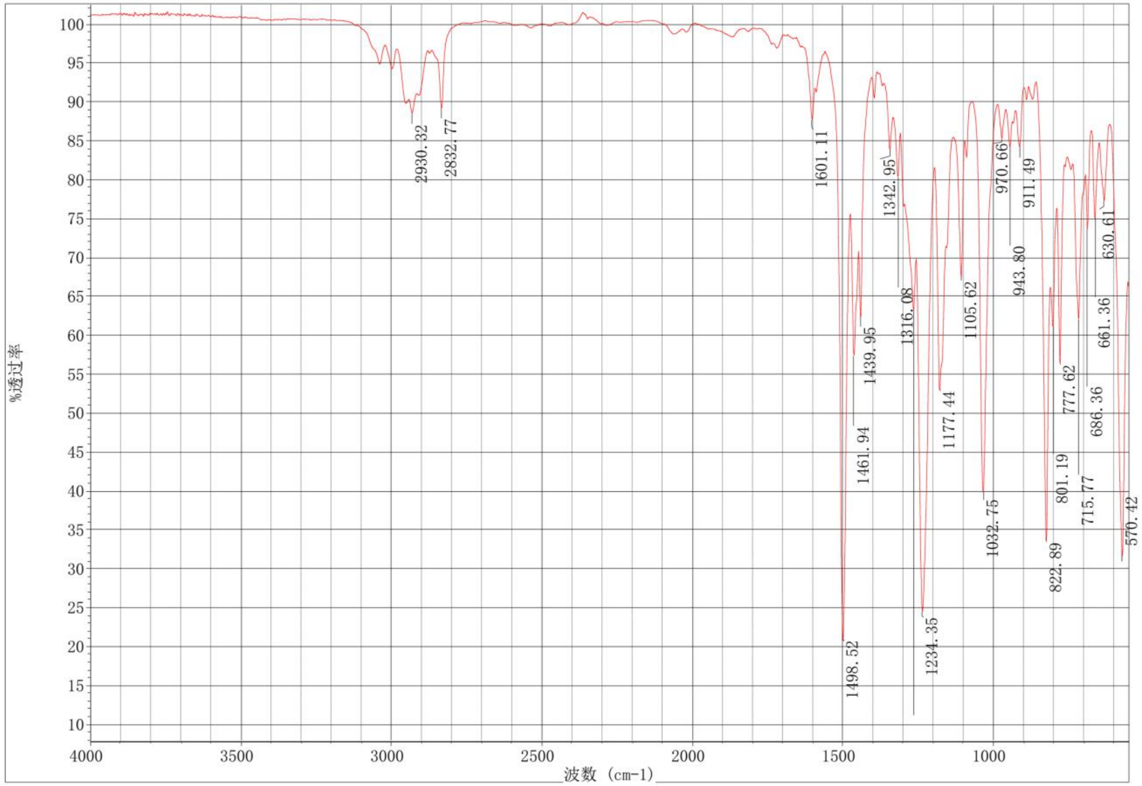

Supplementary Figure 124. IR Spectrum of **3f**

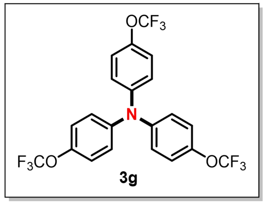

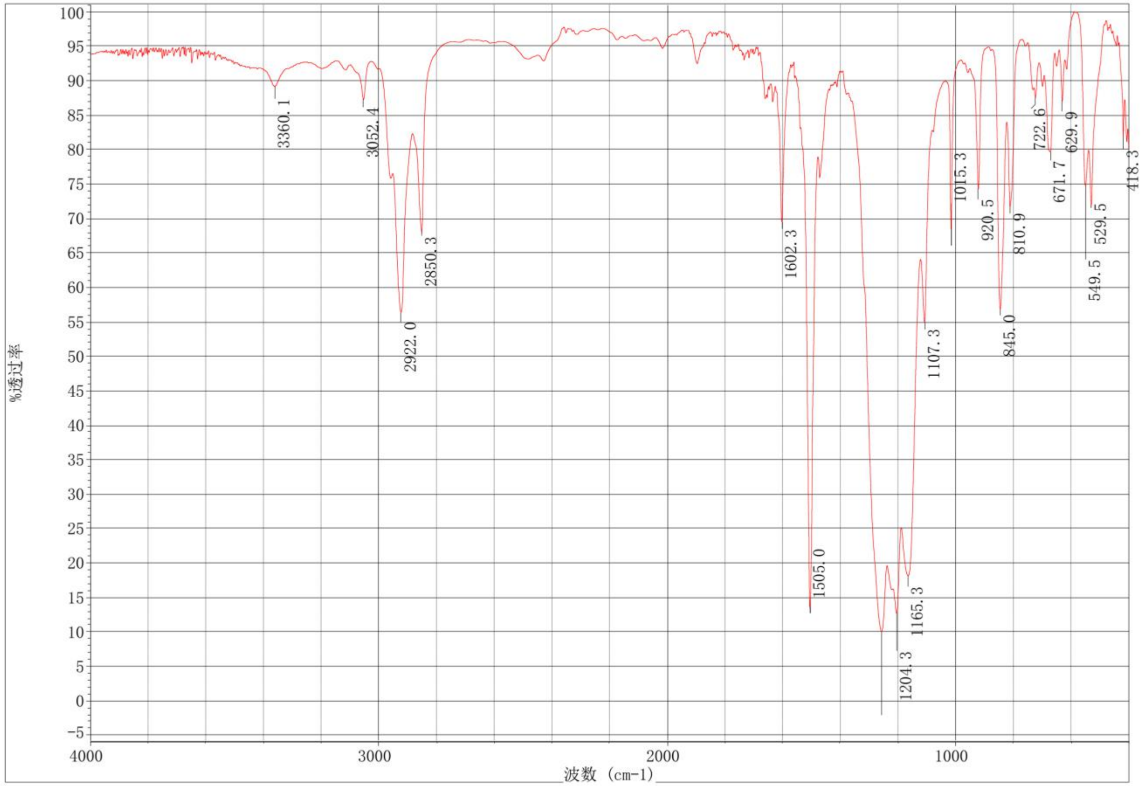

Supplementary Figure 125. IR Spectrum of **3g**

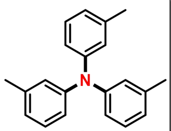

3h

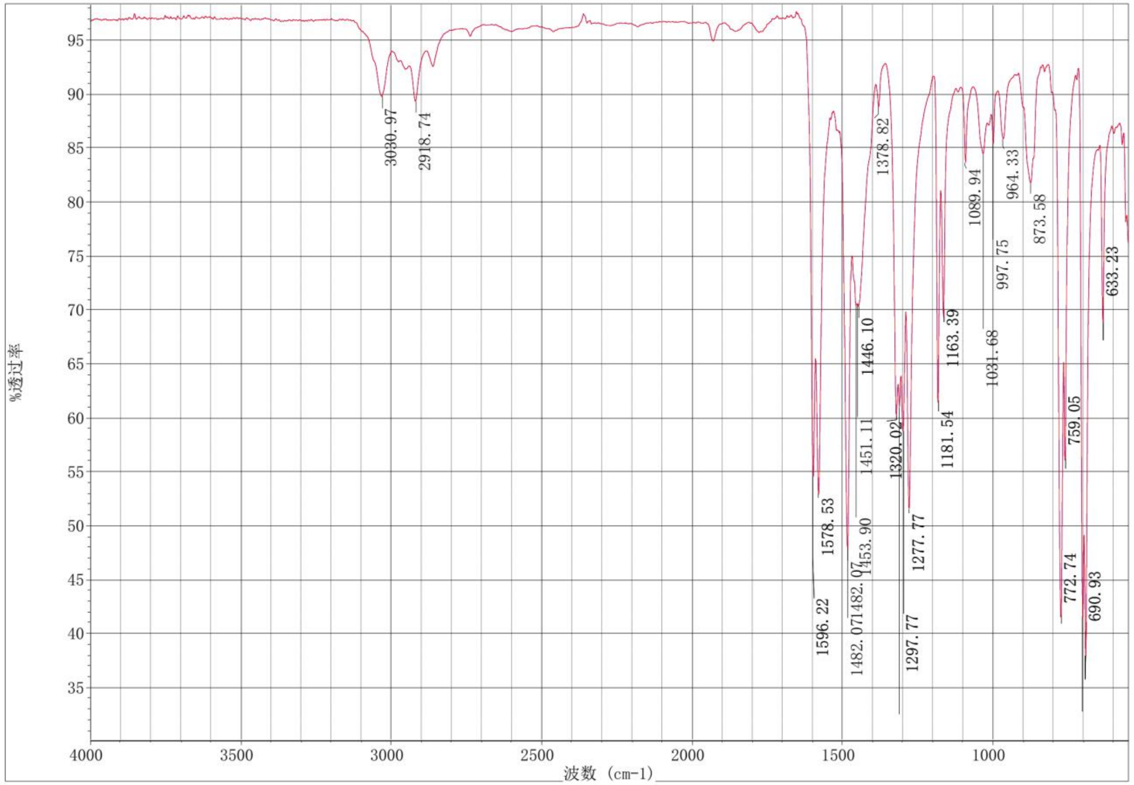

Supplementary Figure 126. IR Spectrum of **3h**

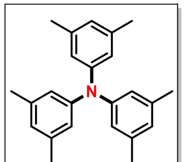

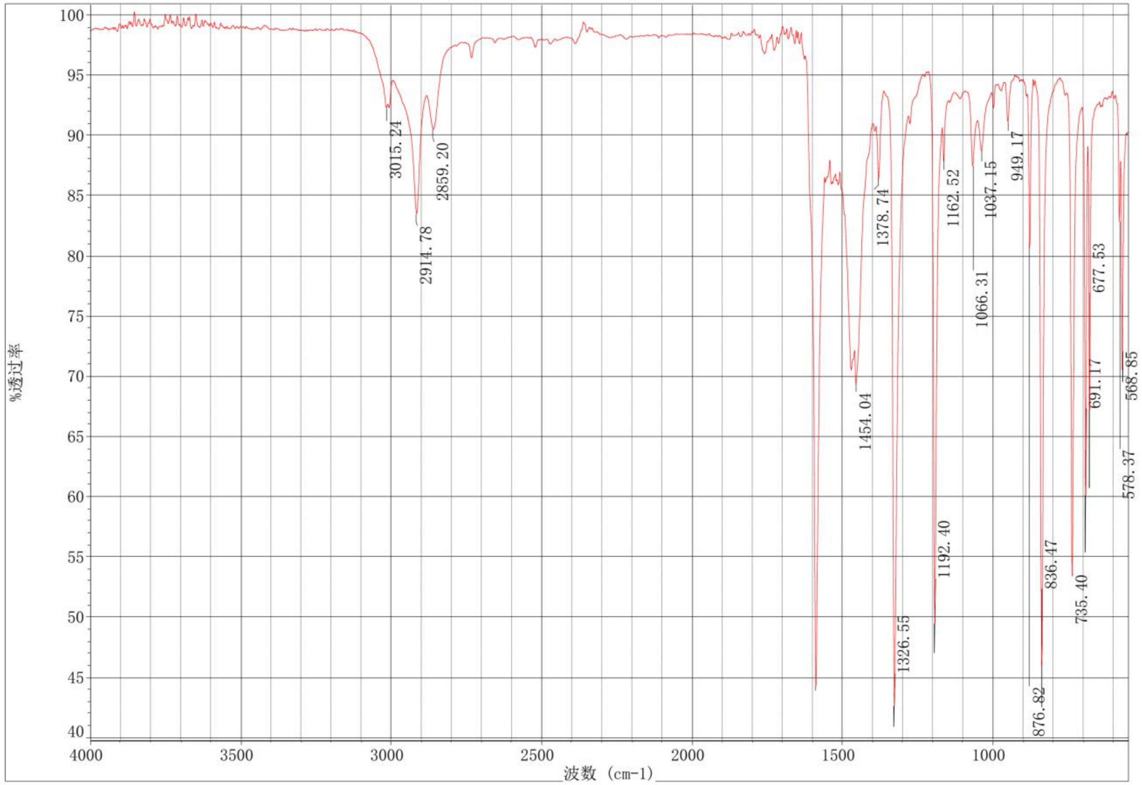

Supplementary Figure 127. IR Spectrum of **3i**

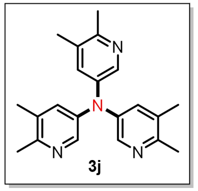

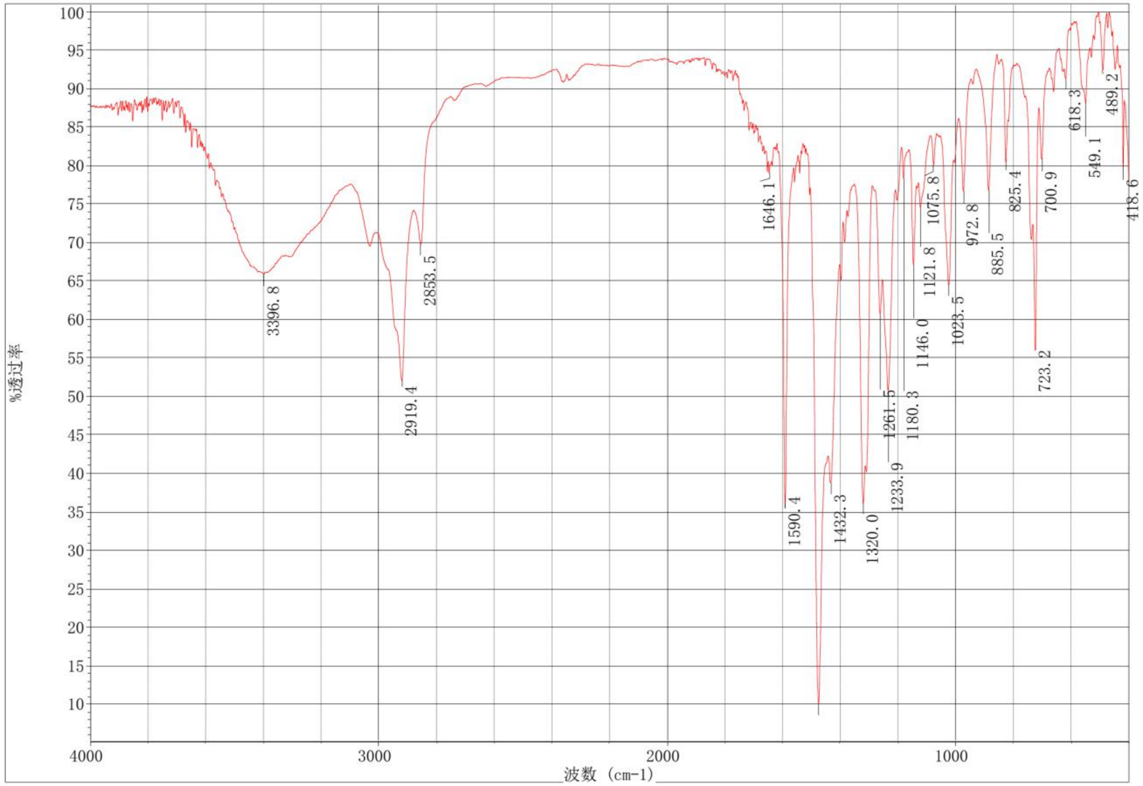

Supplementary Figure 128. IR Spectrum of **3j**

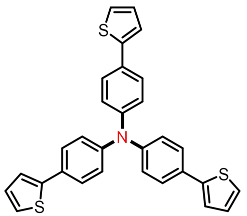

3k

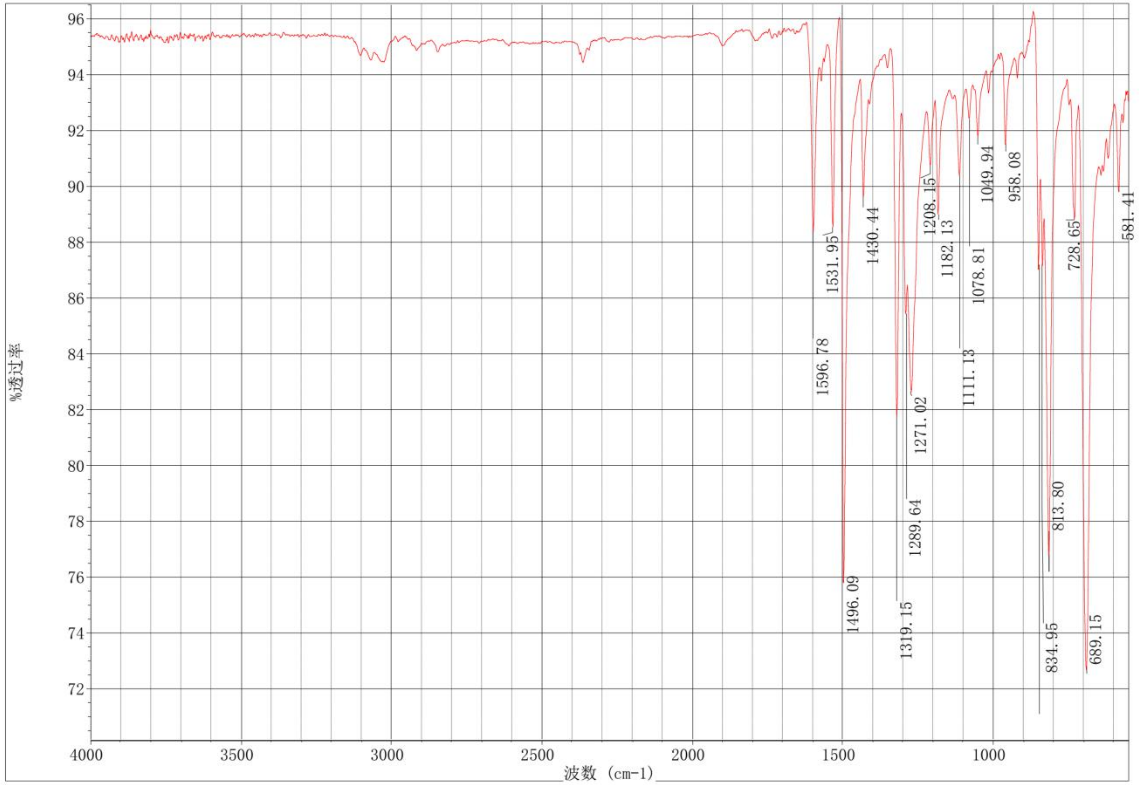

Supplementary Figure 129. IR Spectrum of **3k**

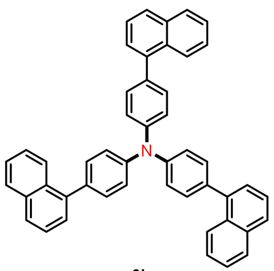

3l

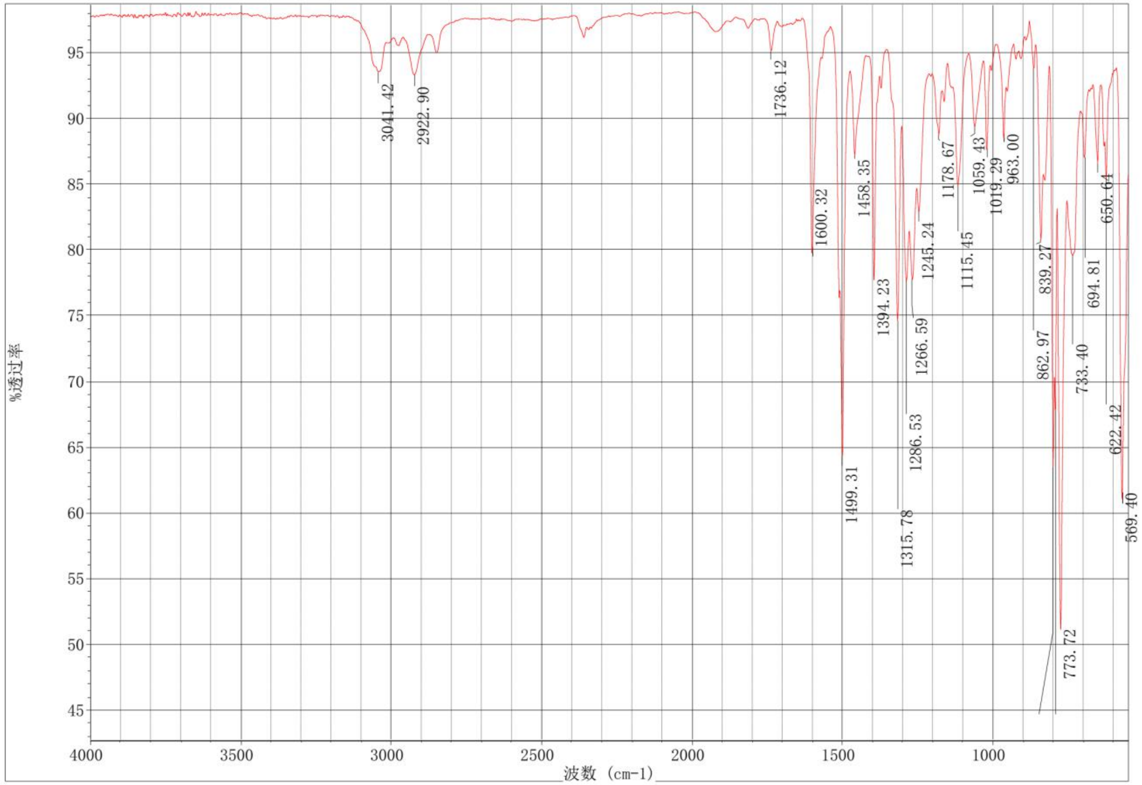

Supplementary Figure 130. IR Spectrum of **3l**

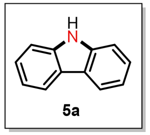

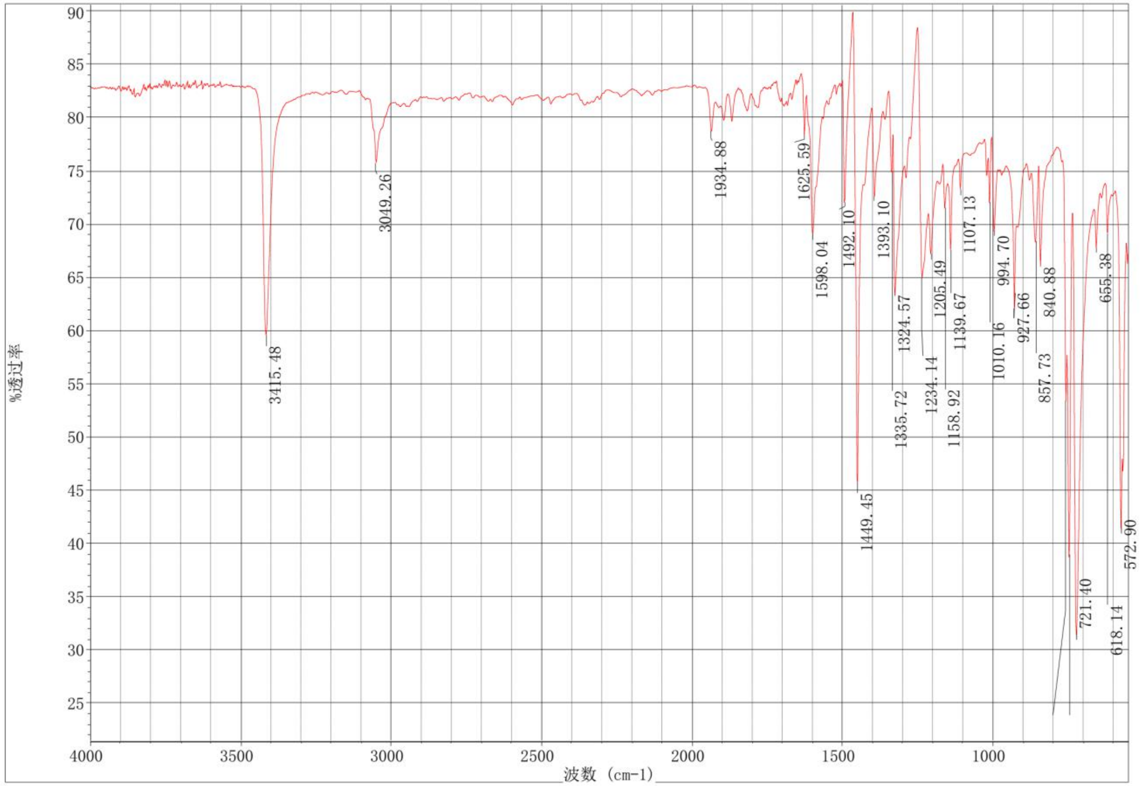

Supplementary Figure 131. IR Spectrum of **5a**

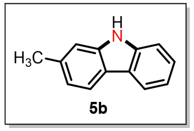

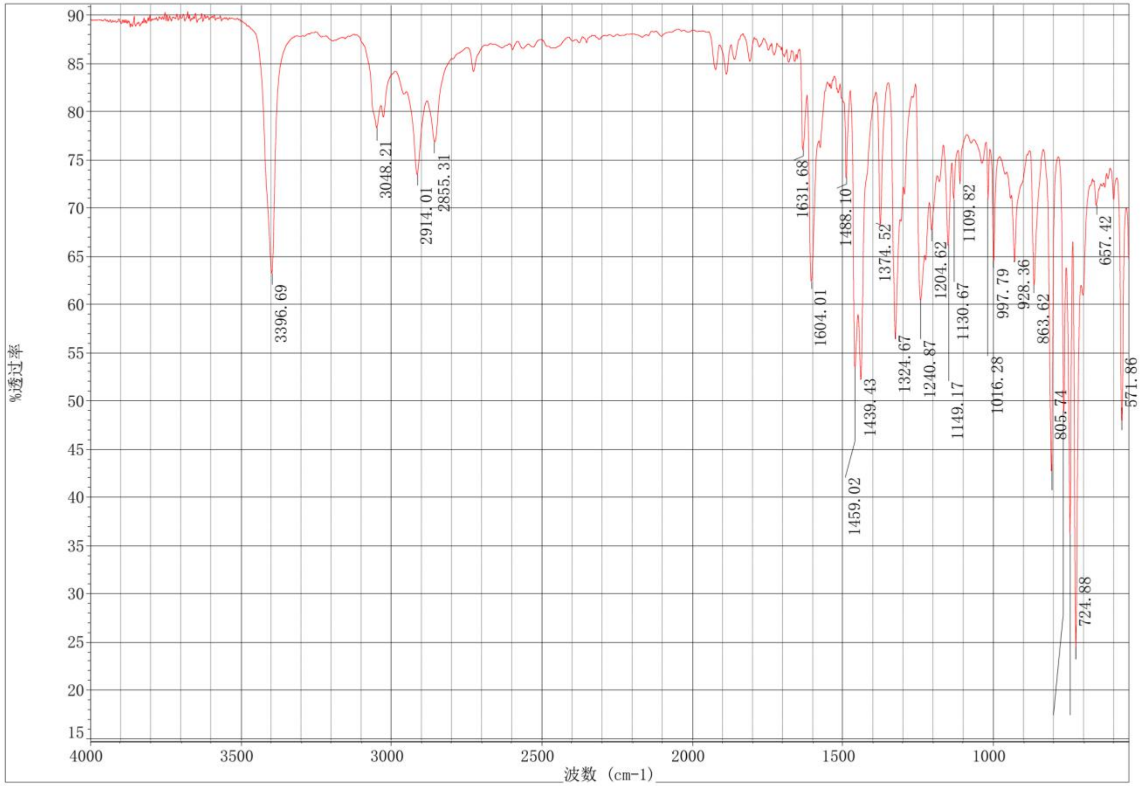

Supplementary Figure 132. IR Spectrum of **5b**

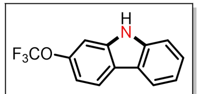

5c

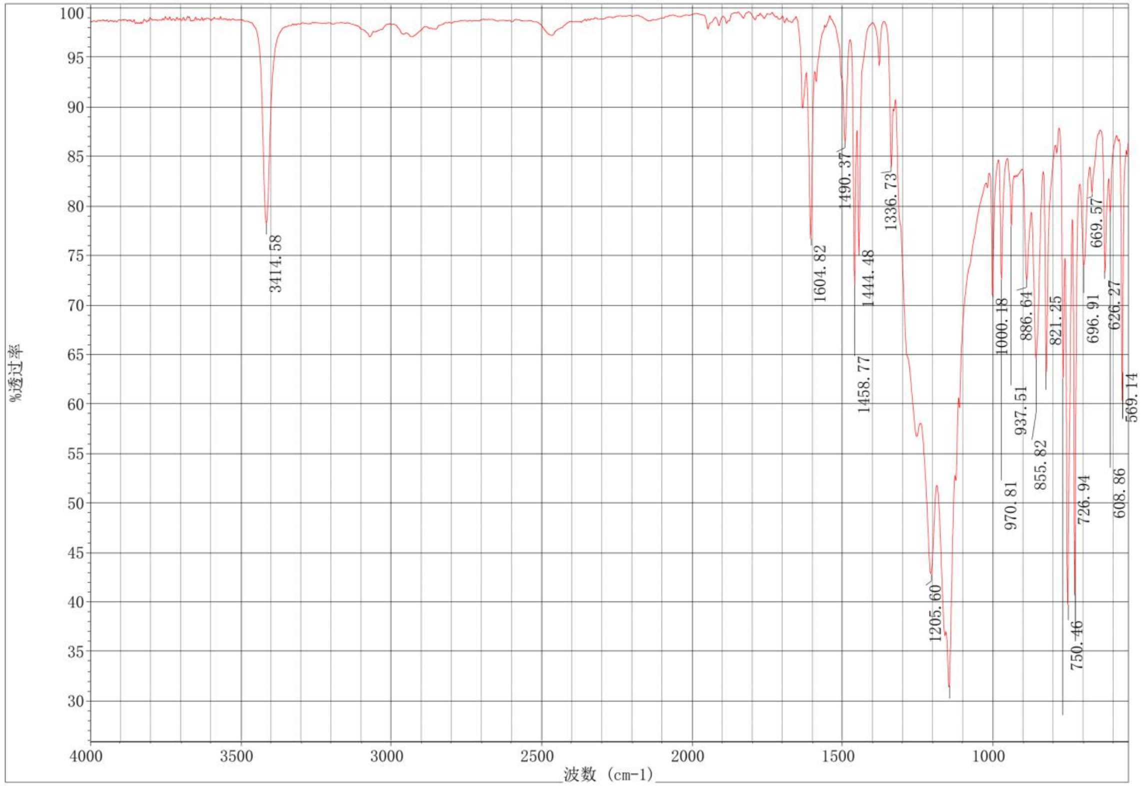

Supplementary Figure 133. IR Spectrum of **5c**

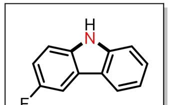

5d

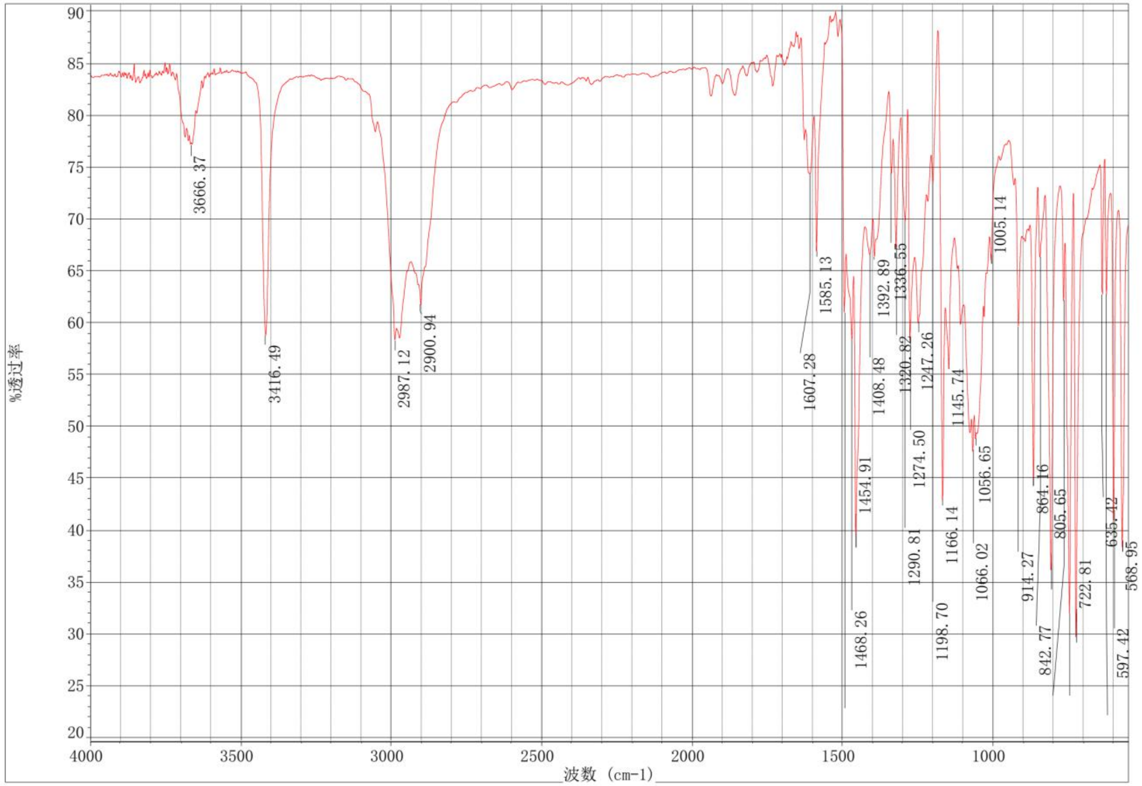

Supplementary Figure 134. IR Spectrum of **5d**

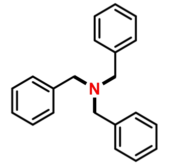

7

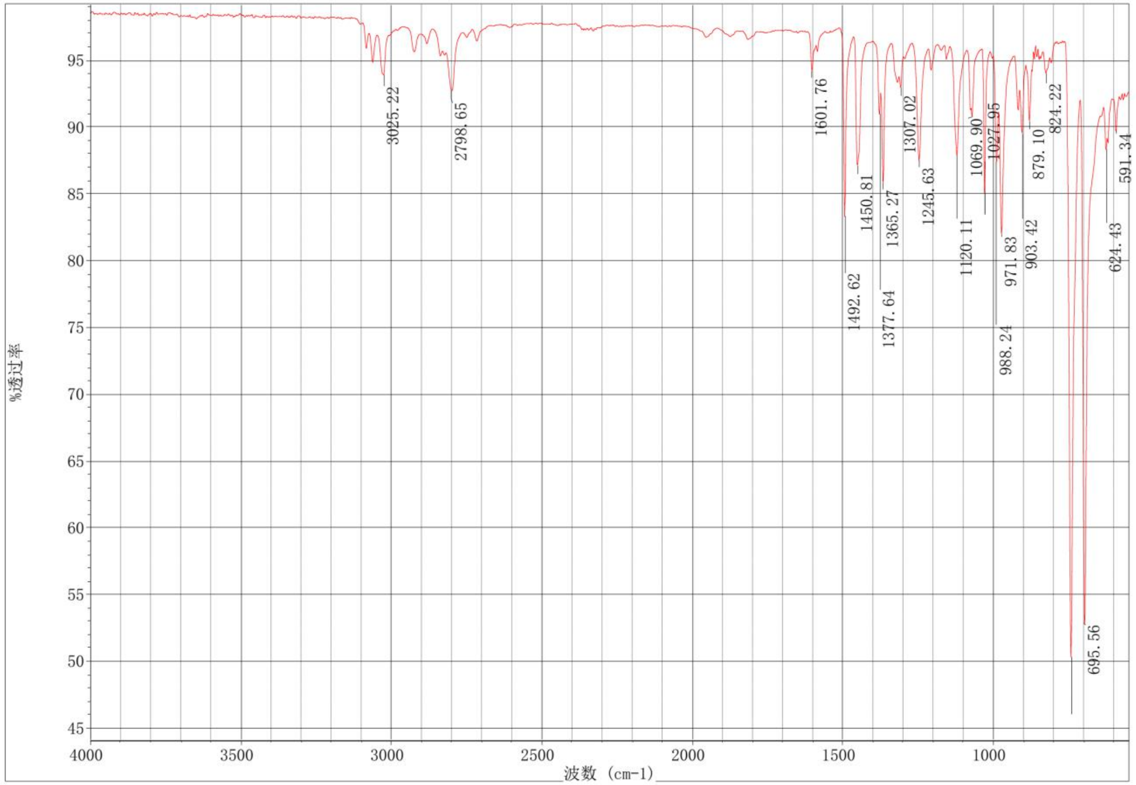

Supplementary Figure 135. IR Spectrum of **7**

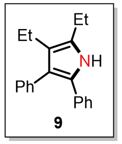

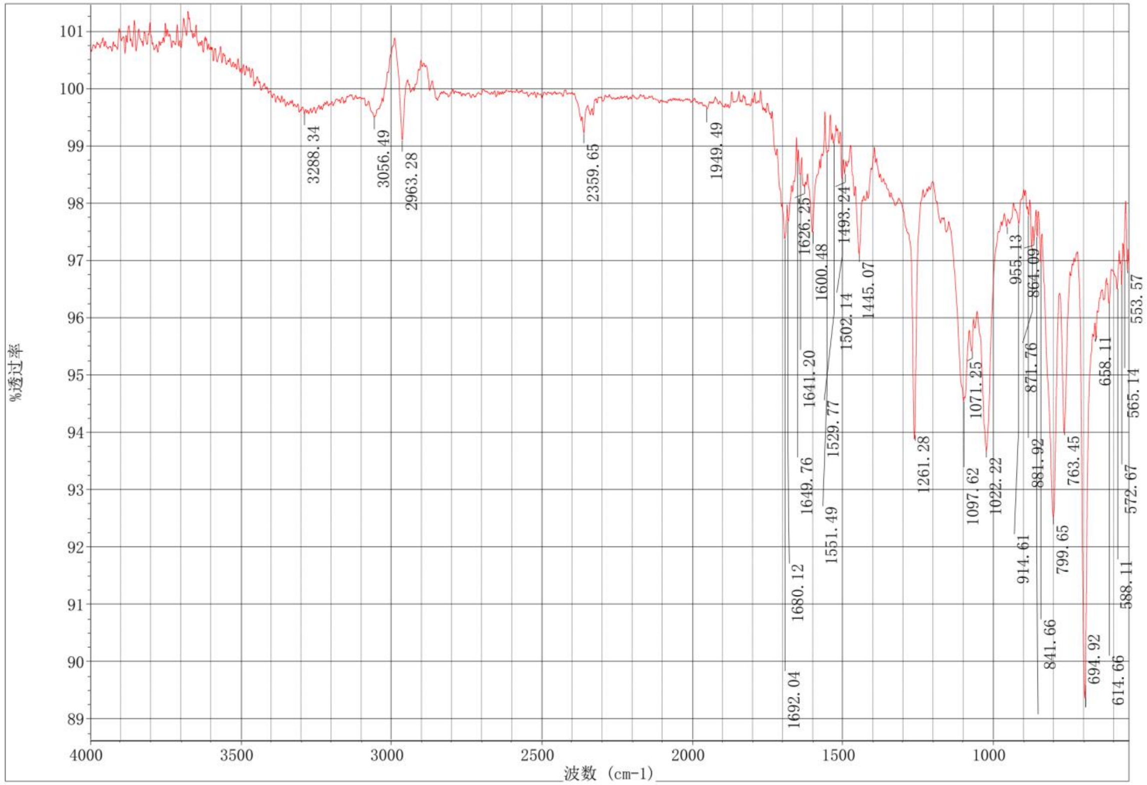

Supplementary Figure 136. IR Spectrum of **9**

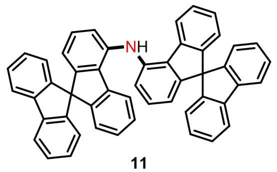

11

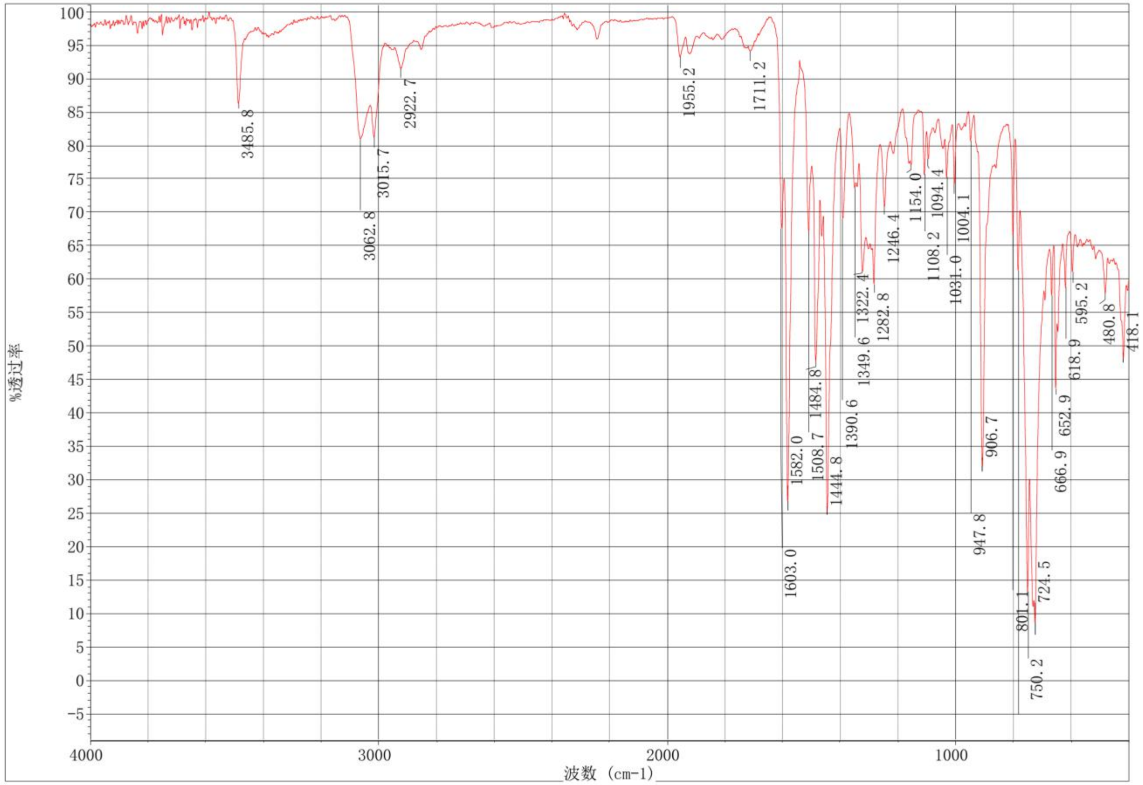

Supplementary Figure 137. IR Spectrum of **11**

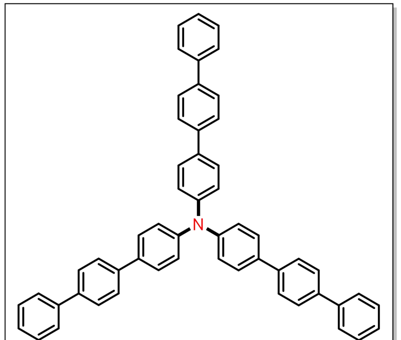

13

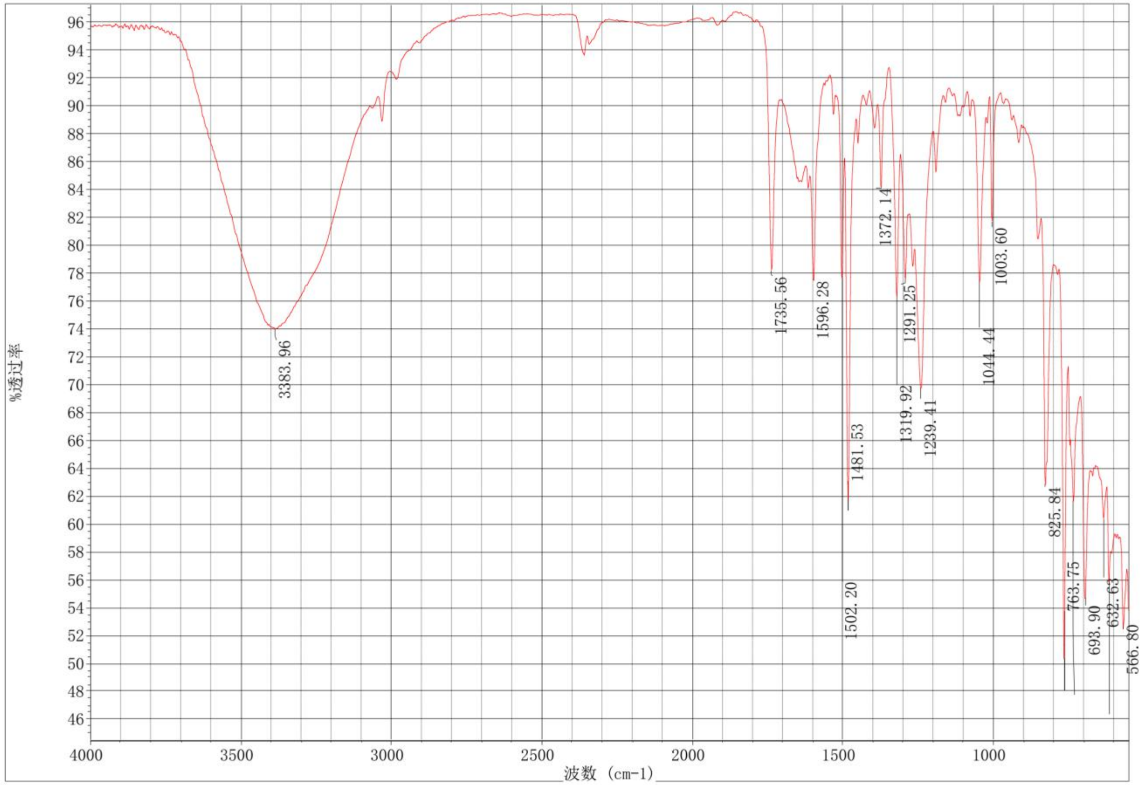

Supplementary Figure 138. IR Spectrum of **13**

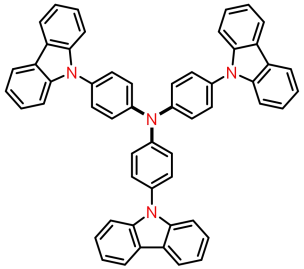

15

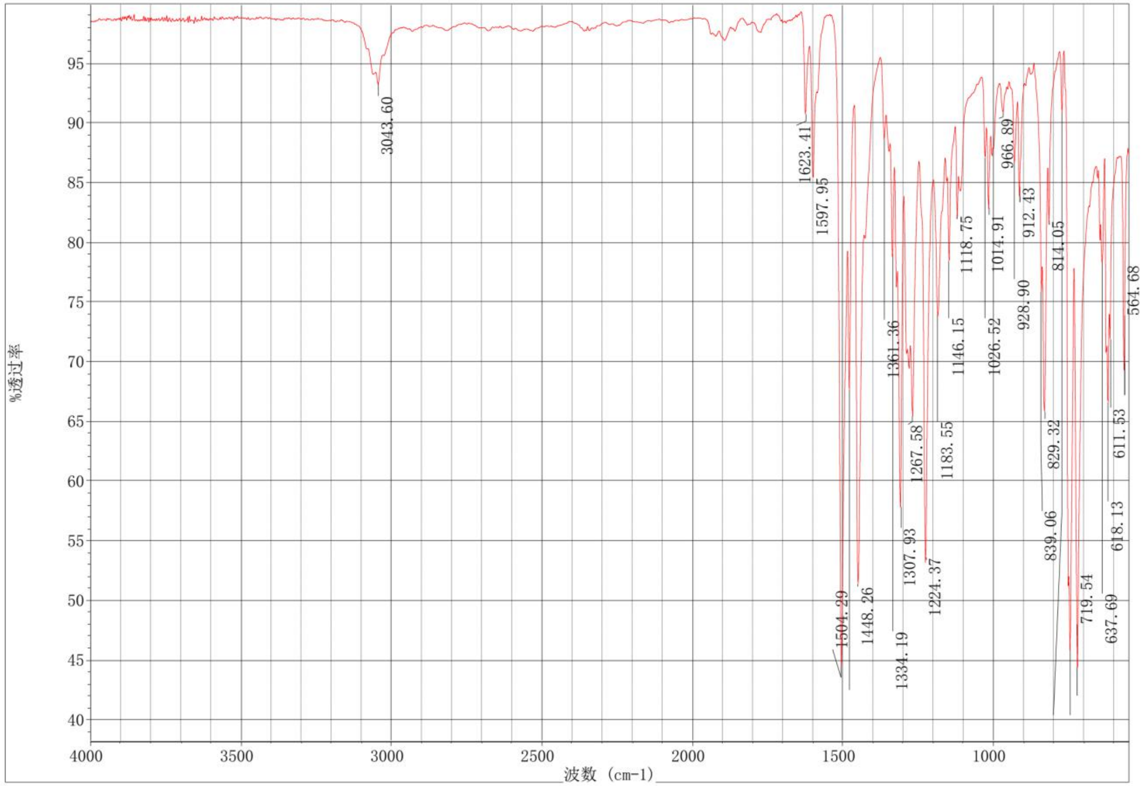

Supplementary Figure 139. IR Spectrum of **15**

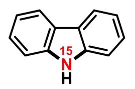

^{15}N-5a

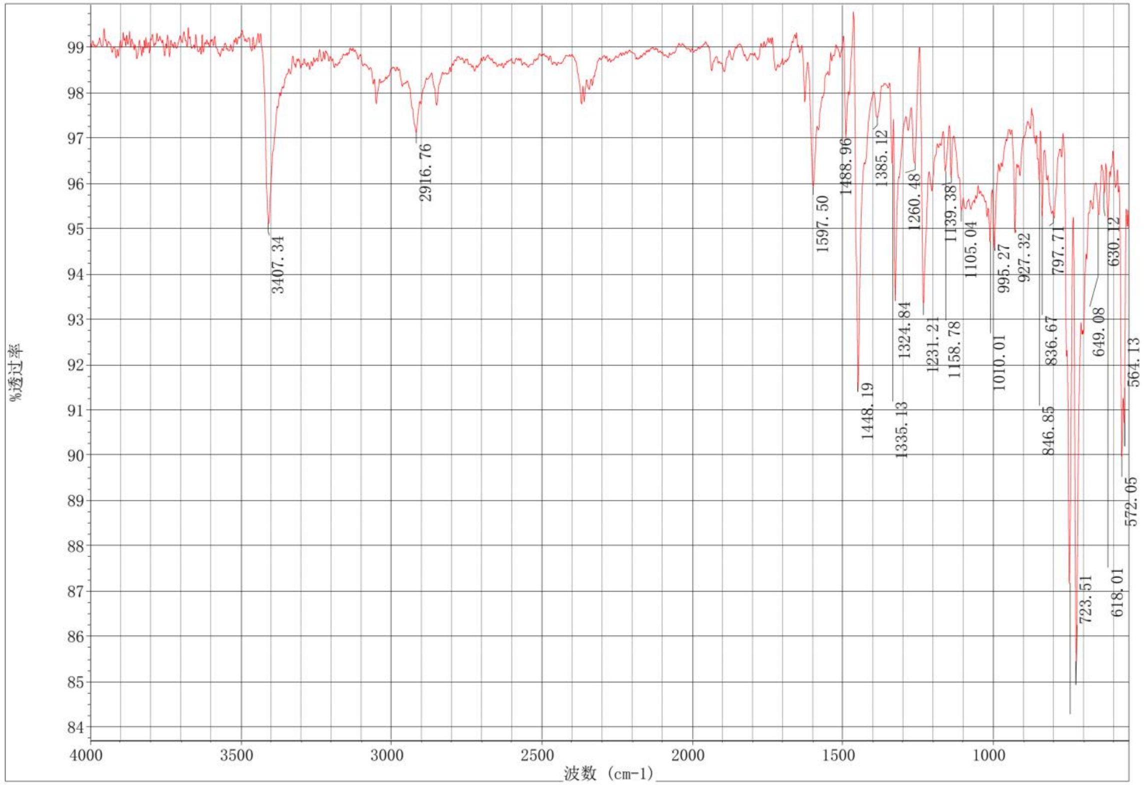

Supplementary Figure 140. IR Spectrum of **^{15}N-5a**

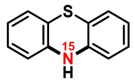

^{15}N-17

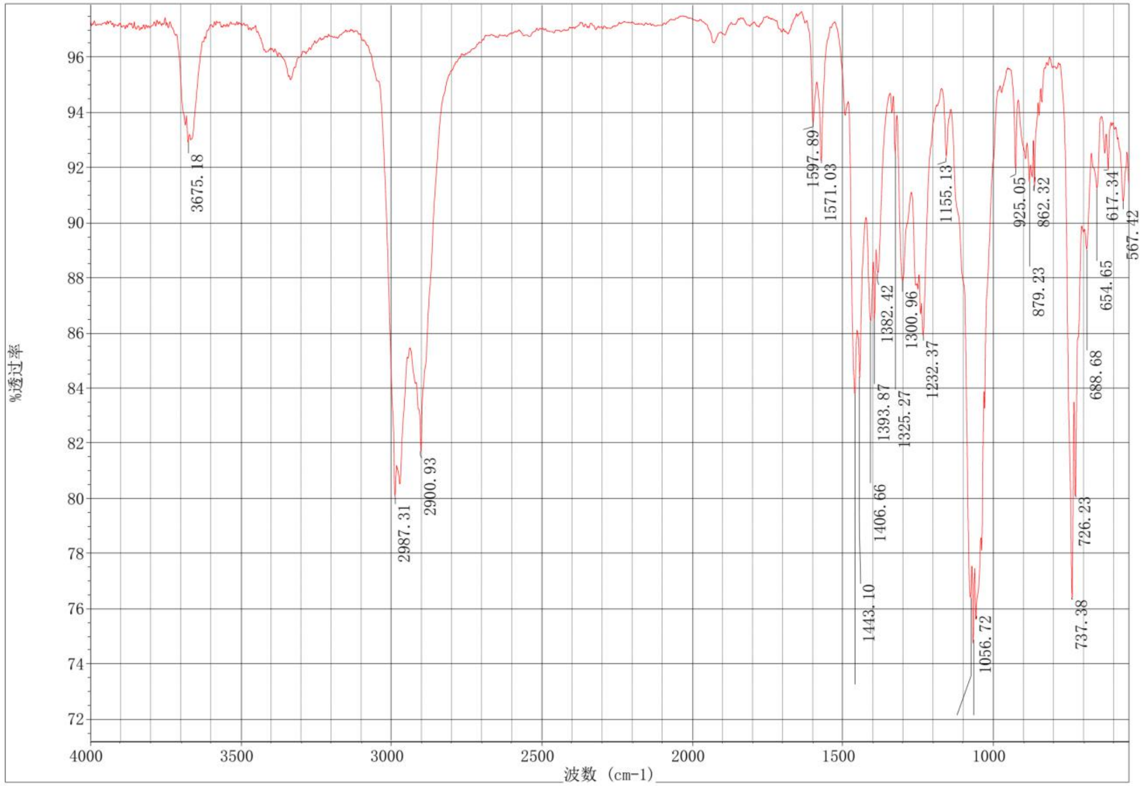

Supplementary Figure 141. IR Spectrum of **^{15}N-17**

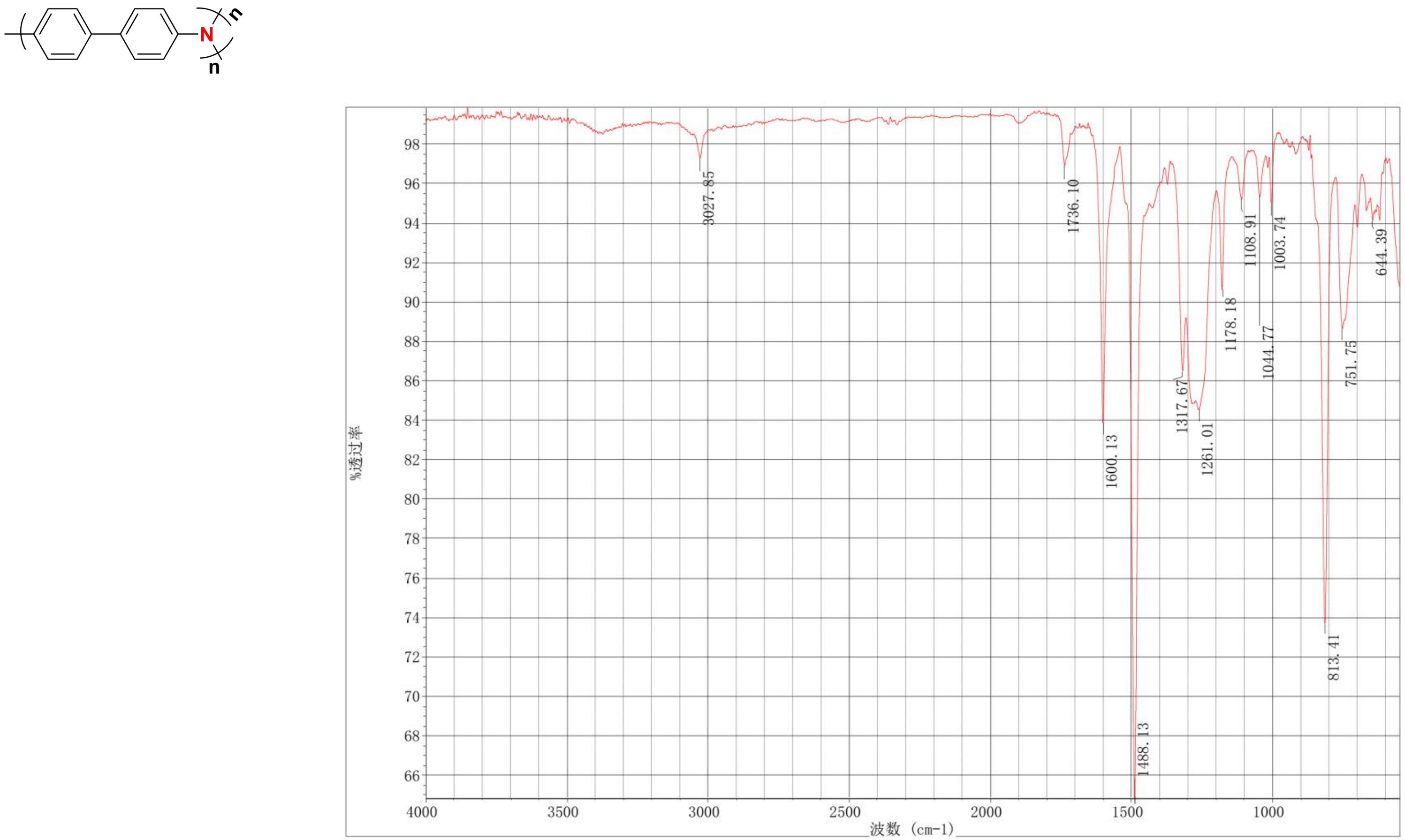

Supplementary Figure 142. IR Spectrum of **diphenyl-bridged polyaniline**

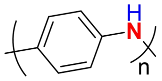

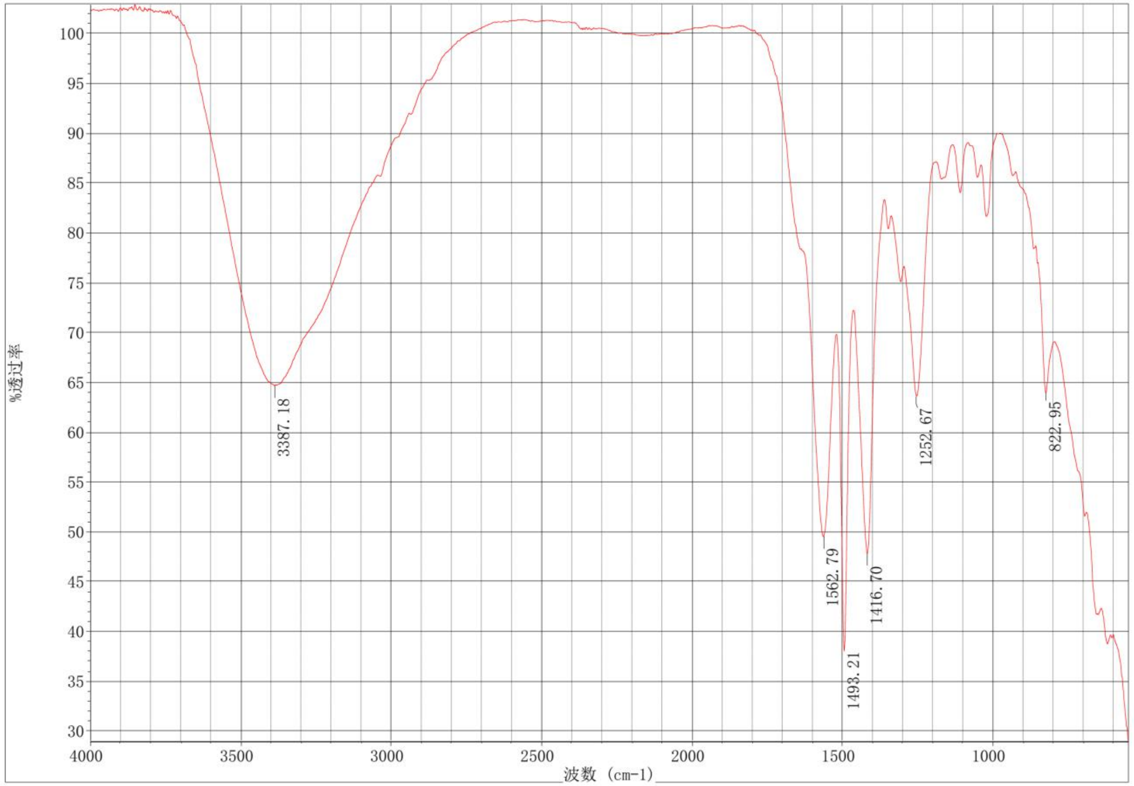

Supplementary Figure 143. IR Spectrum of **1,4-polyaniline**

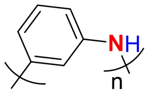

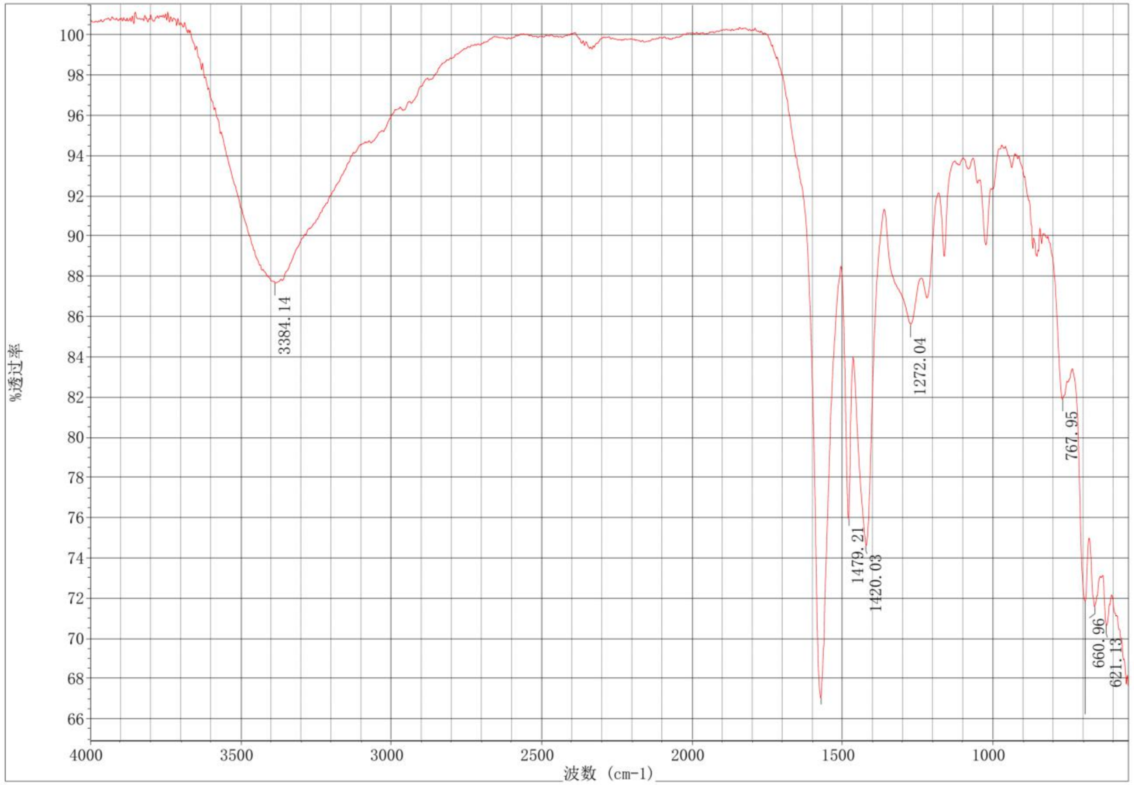

Supplementary Figure 144. IR Spectrum of **1,3-polyaniline**

(VII) GPC Spectral Data for Polyaniline Products

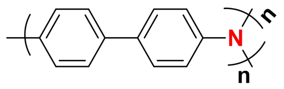

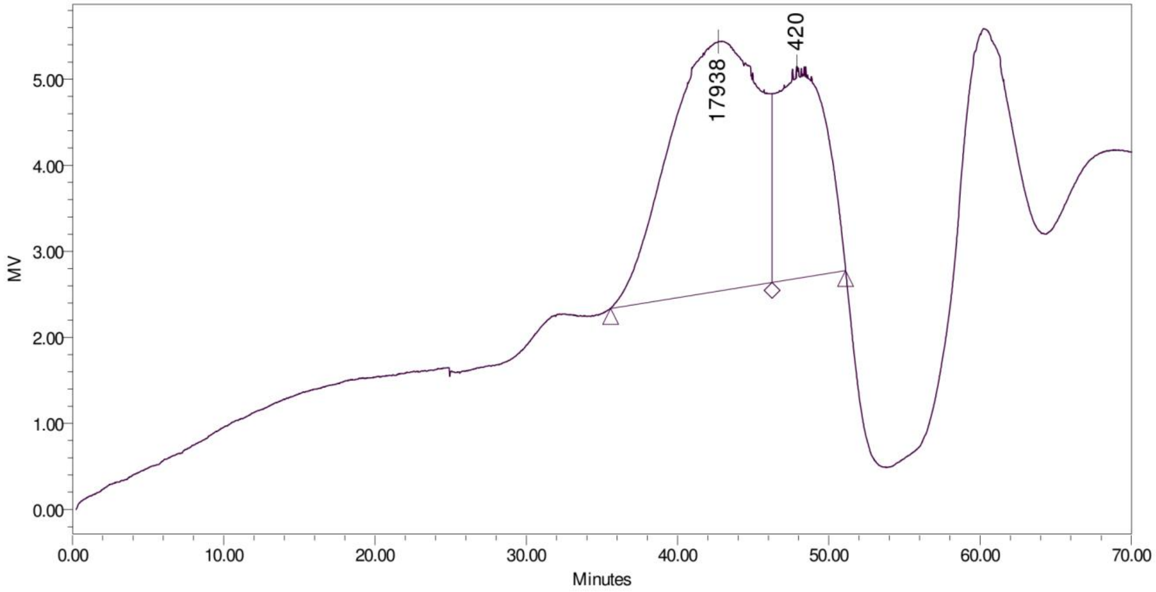

Broad Unknown Relative Peak Table

	Distribution Name	Mn (Daltons)	Mw (Daltons)	MP (Daltons)	Mz (Daltons)	Mz+1 (Daltons)	Polydispersity	Mz/Mw	Mz+1/Mw
1		15239	27023	17938	52975	97337	1.773279	1.960406	3.602043
2		17	859	420	2218	2751	49.870162	2.583145	3.203458

Supplementary Figure 145. GPC Spectrum of **diphenyl-bridged polyaniline**

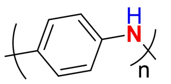

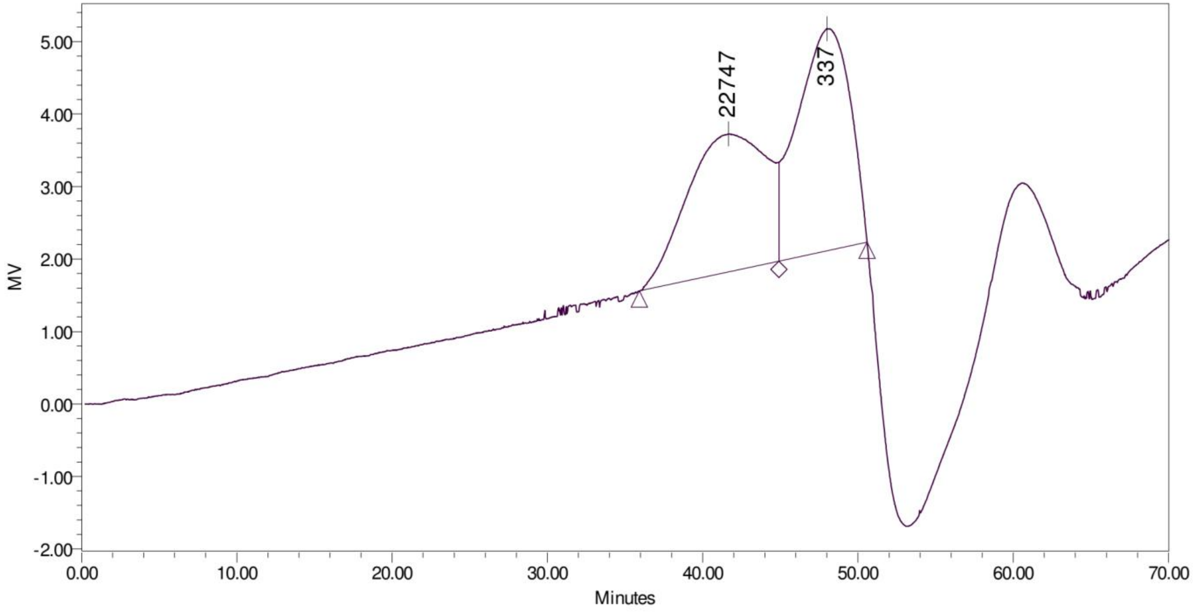

Broad Unknown Relative Peak Table

	Distribution Name	Mn (Daltons)	Mw (Daltons)	MP (Daltons)	Mz (Daltons)	Mz+1 (Daltons)	Polydispersity	Mz/Mw	Mz+1/Mw
1		22057	31850	22747	51650	82122	1.443957	1.621668	2.578392
2		26	1857	337	5077	6439	72.528002	2.734111	3.468092

Supplementary Figure 146. GPC Spectrum of **1,4-polyaniline**

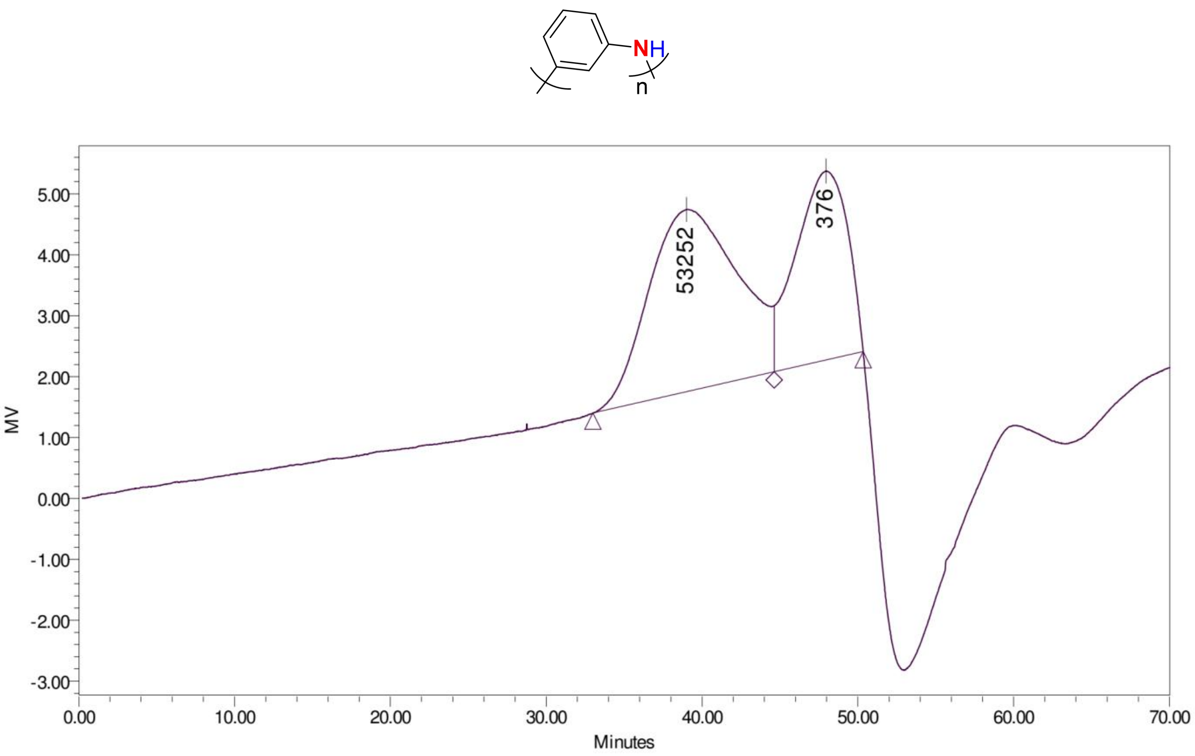

Broad Unknown Relative Peak Table

	Distribution Name	Mn (Daltons)	Mw (Daltons)	MP (Daltons)	Mz (Daltons)	Mz+1 (Daltons)	Polydispersity	Mz/Mw	Mz+1/Mw
1		35474	82593	53252	238244	517872	2.328283	2.884553	6.270168
2		30	2047	376	5565	7132	68.003897	2.718343	3.483980

Supplementary Figure 147. GPC Spectrum of **1,3-polyaniline**

(VIII) HRMS Spectral Data for Products

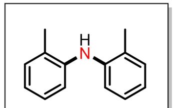

2a

Elemental composition search on mass 198.13

m/z= 193.13-203.13

m/z	Theo. Mass	Delta (ppm)	RDB equiv.	Composition
198.1274	198.1273	0.19	-5.0	$C_5H_{20}O_5F_2$
	198.1277	-1.80	7.5	$C_{14}H_{16}N$

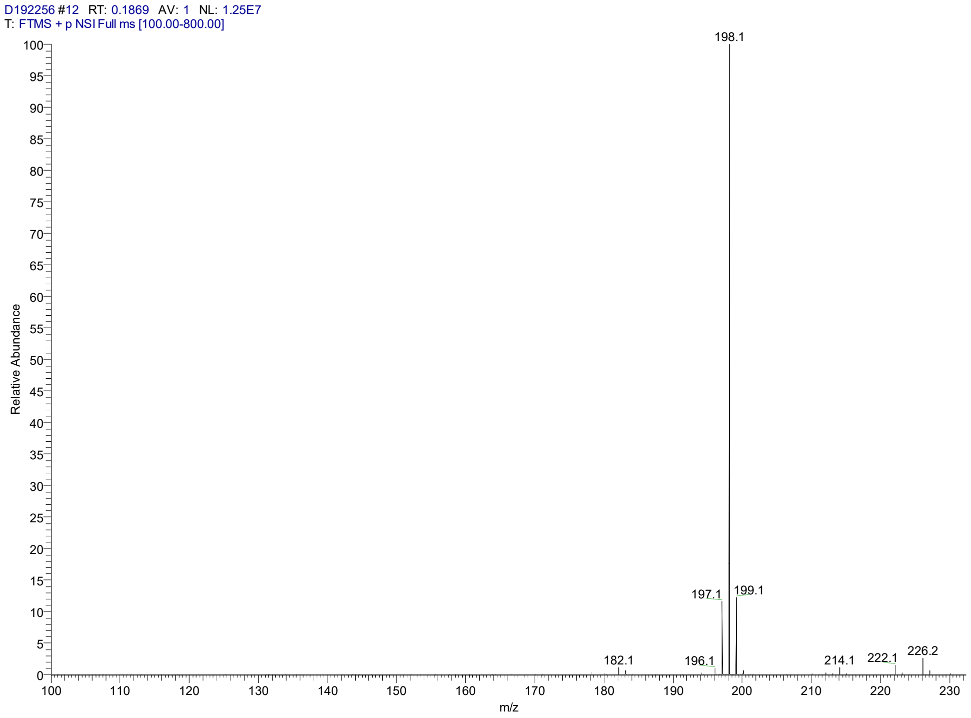

Supplementary Figure 148. HRMS Spectrum of **2a**

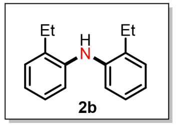

Elemental composition search on mass 226.16

m/z= 221.16-231.16

m/z	Theo. Mass	Delta (ppm)	RDB equiv.	Composition
226.1585	226.1586	-0.41	-5.0	$C_7H_{24}O_5F_2$
	226.1590	-2.15	7.5	$C_{16}H_{20}N$
	226.1575	4.65	-1.0	$C_{10}H_{23}O_4F$

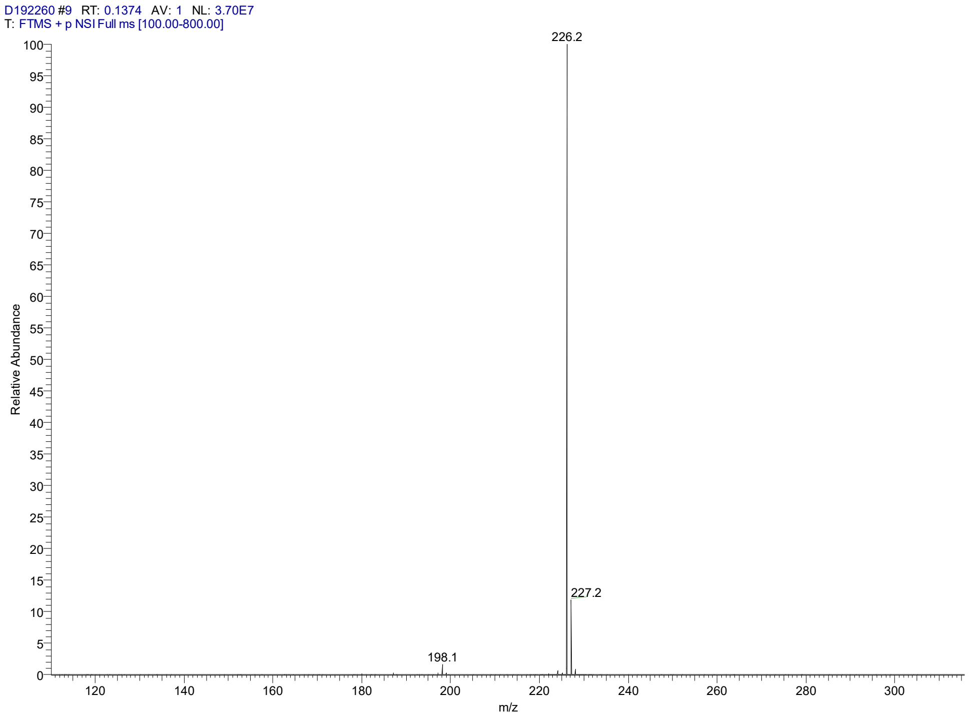

Supplementary Figure 149. HRMS Spectrum of **2b**

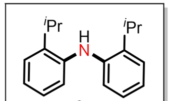

2c

Elemental composition search on mass 254.19

m/z= 249.19-259.19

m/z	Theo. Mass	Delta (ppm)	RDB equiv.	Composition
254.1901	254.1901	-0.03	4.0	$C_{13}H_{23}N_4F$
	254.1899	0.74	-5.0	$C_9H_{28}O_5F_2$
	254.1903	-0.81	7.5	$C_{18}H_{24}N$
	254.1897	1.53	-8.5	$C_4H_{27}O_5N_3F_3$
	254.1897	1.59	-9.0	$C_7H_{30}O_2F_4S$
	254.1897	1.75	-1.5	$C_{10}H_{28}O_2N_3S$
	254.1906	-1.89	-9.5	$C_5H_{31}N_3F_3S_2$
	254.1908	-2.68	-6.0	$C_{10}H_{32}F_2S_2$
	254.1908	-2.74	-5.5	$C_7H_{29}O_3N_3FS$
	254.1910	-3.53	-2.0	$C_{12}H_{30}O_3S$

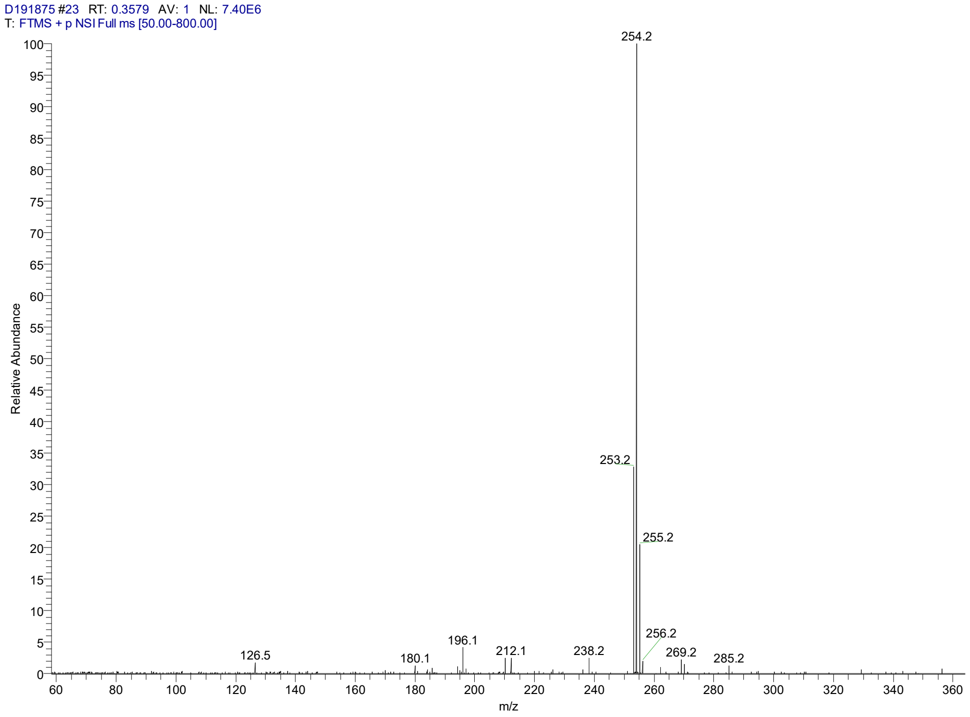

Supplementary Figure 150. HRMS Spectrum of **2c**

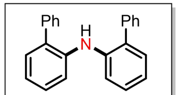

2d

Elemental composition search on mass 322.16

m/z= 317.16-327.16

m/z	Theo. Mass	Delta (ppm)	RDB equiv.	Composition
322.1587	322.1586	0.12	3.0	$C_{15}H_{24}O_5F_2$
	322.1590	-1.11	15.5	$C_{24}H_{20}N$
	322.1575	3.67	7.0	$C_{18}H_{23}O_4F$
	322.1602	-4.65	11.5	$C_{21}H_{21}ONF$

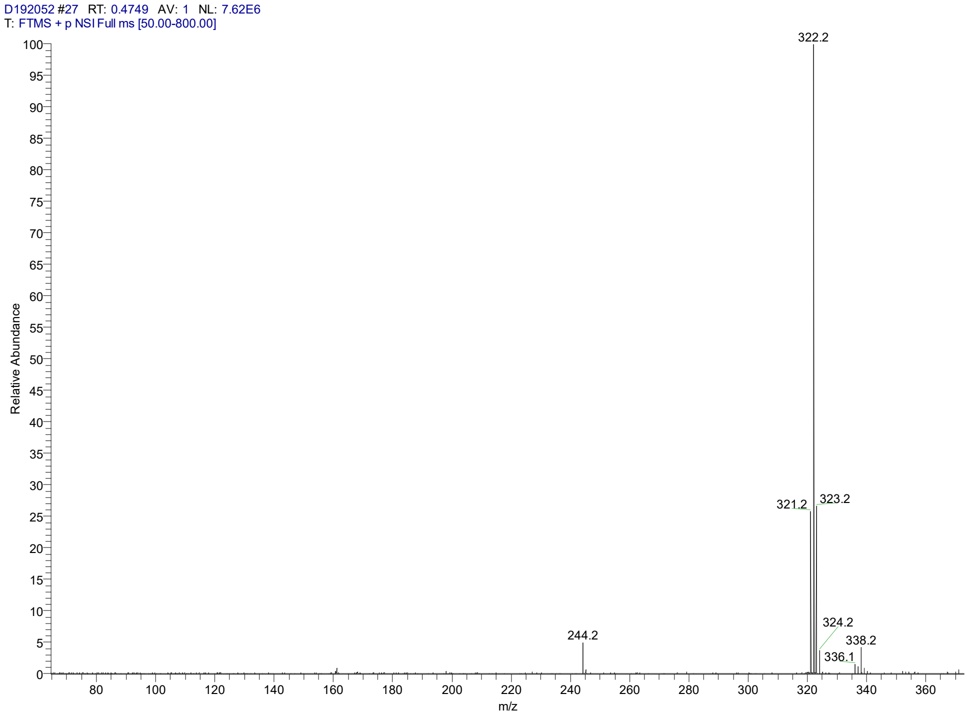

Supplementary Figure 151. HRMS Spectrum of **2d**

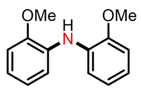

2e

Elemental composition search on mass 230.12

m/z= 225.12-235.12

m/z	Theo. Mass	Delta (ppm)	RDB equiv.	Composition
230.1173	230.1176	-1.28	7.5	$C_{14}H_{16}O_2N$

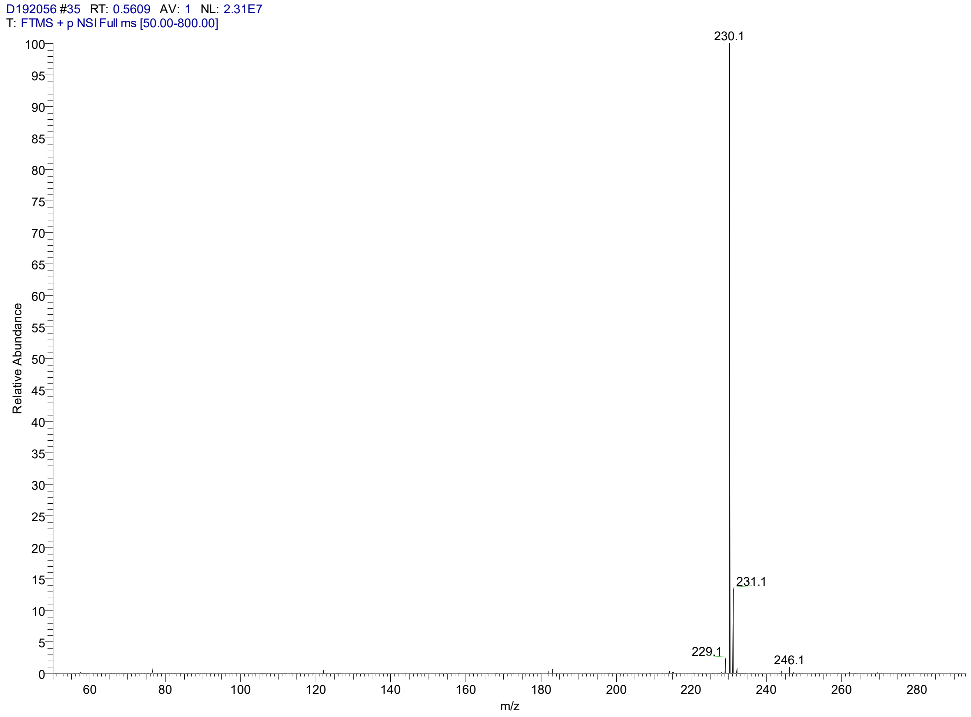

Supplementary Figure 152. HRMS Spectrum of **2e**

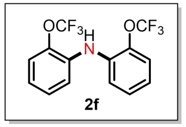

Elemental composition search on mass 338.06

m/z= 333.06-343.06

m/z	Theo. Mass	Delta (ppm)	RDB equiv.	Composition
338.0606	338.0610	-1.10	15.0	$C_{17}H_8O_2N_4F_2$
	338.0610	-1.23	7.5	$C_{14}H_{10}O_2NF_6$
	338.0600	1.68	22.5	$C_{25}H_8ON$
	338.0612	-1.70	18.5	$C_{22}H_9O_2NF$
	338.0599	2.16	11.5	$C_{17}H_9ONF_5$
	338.0598	2.28	19.0	$C_{20}H_7ON_4F$
	338.0597	2.75	8.0	$C_{12}H_8ON_4F_6$
	338.0621	-4.49	11.0	$C_{14}H_9O_3N_4F_3$

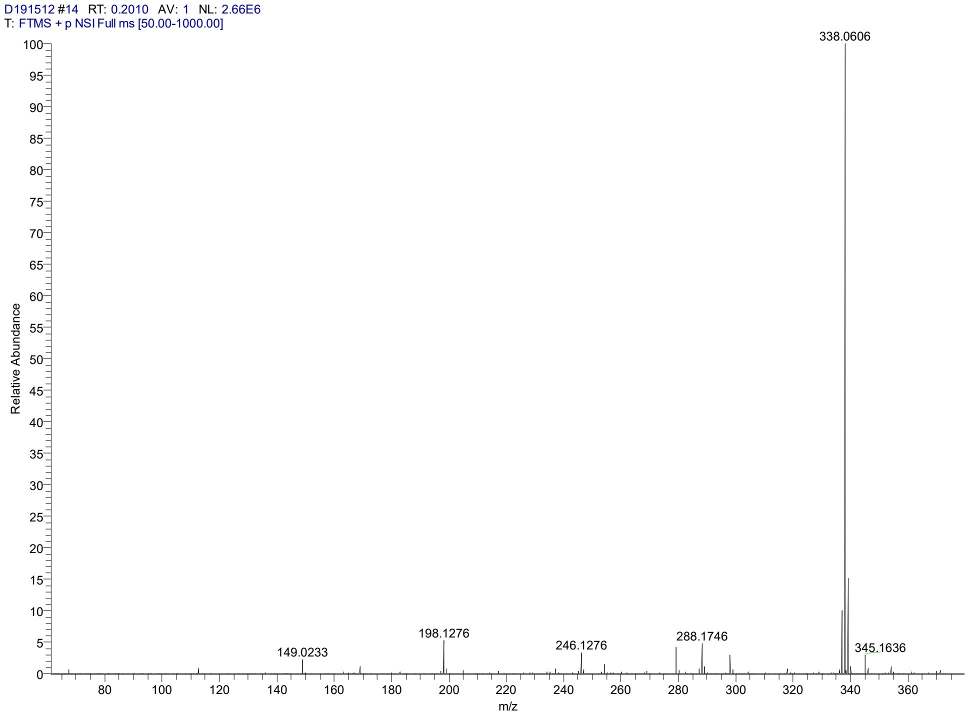

Supplementary Figure 153. HRMS Spectrum of **2f**

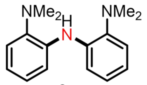

2g

Elemental composition search on mass 256.18

m/z= 251.18-261.18

m/z	Theo. Mass	Delta (ppm)	RDB equiv.	Composition
256.1809	256.1808	0.10	7.5	$C_{16}H_{22}N_3$

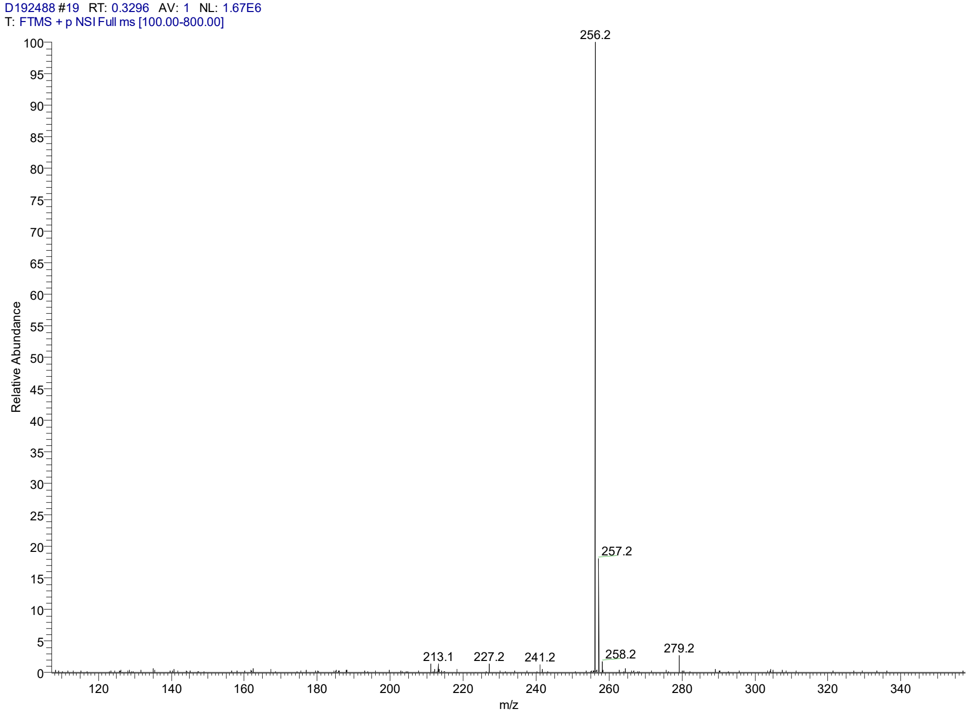

Supplementary Figure 154. HRMS Spectrum of **2g**

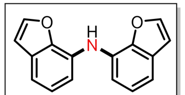

2h

Elemental composition search on mass 250.09

m/z= 245.09-255.09

m/z	Theo. Mass	Delta (ppm)	RDB equiv.	Composition
250.0860	250.0861	-0.30	0.5	$C_8 H_{13} O_2 N F_5$
	250.0863	-0.94	11.5	$C_{16} H_{12} O_2 N$
	250.0850	4.27	4.5	$C_{11} H_{12} O N F_4$
	250.0872	-4.87	-3.5	$C_5 H_{14} O_3 N F_6$
	250.0848	4.90	-6.5	$C_3 H_{13} O N F_9$

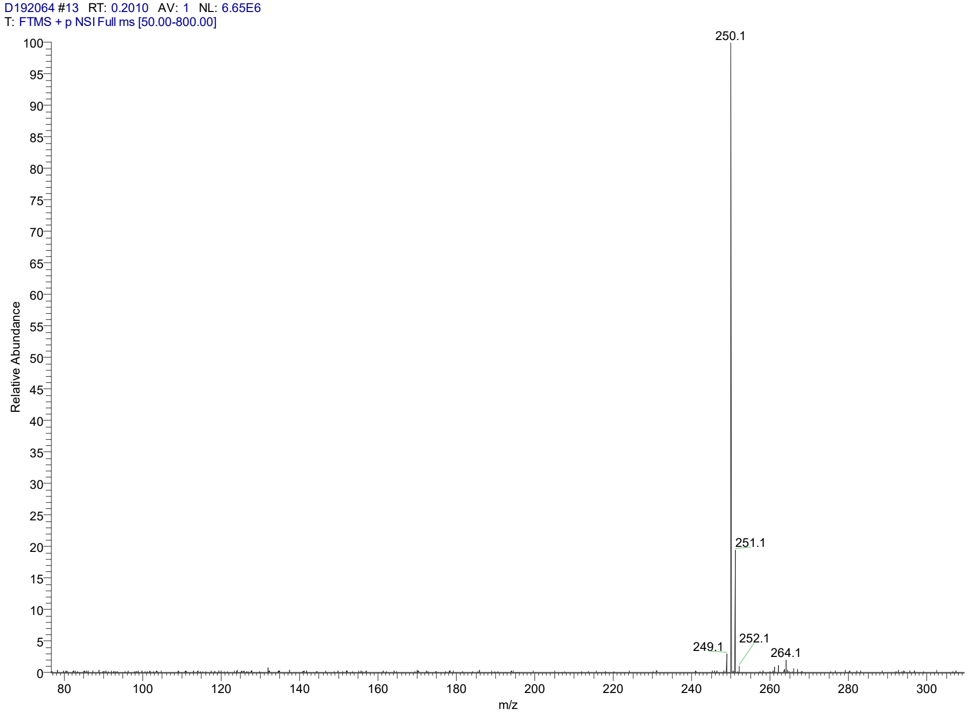

Supplementary Figure 155. HRMS Spectrum of **2h**

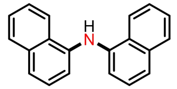

2i

Elemental composition search on mass 270.13

m/z= 265.13-275.13

m/z	Theo. Mass	Delta (ppm)	RDB equiv.	Composition
270.1275	270.1277	-0.84	13.5	$C_{20}H_{16}N$
	270.1278	-0.98	-5.0	$C_6H_{26}O_5N_2S_2$
	270.1271	1.58	4.5	$C_{12}H_{20}O_2N_3S$
	270.1284	-3.39	4.0	$C_{14}H_{22}O_3S$
	270.1264	3.99	-4.5	$C_4H_{24}O_4N_5S_2$

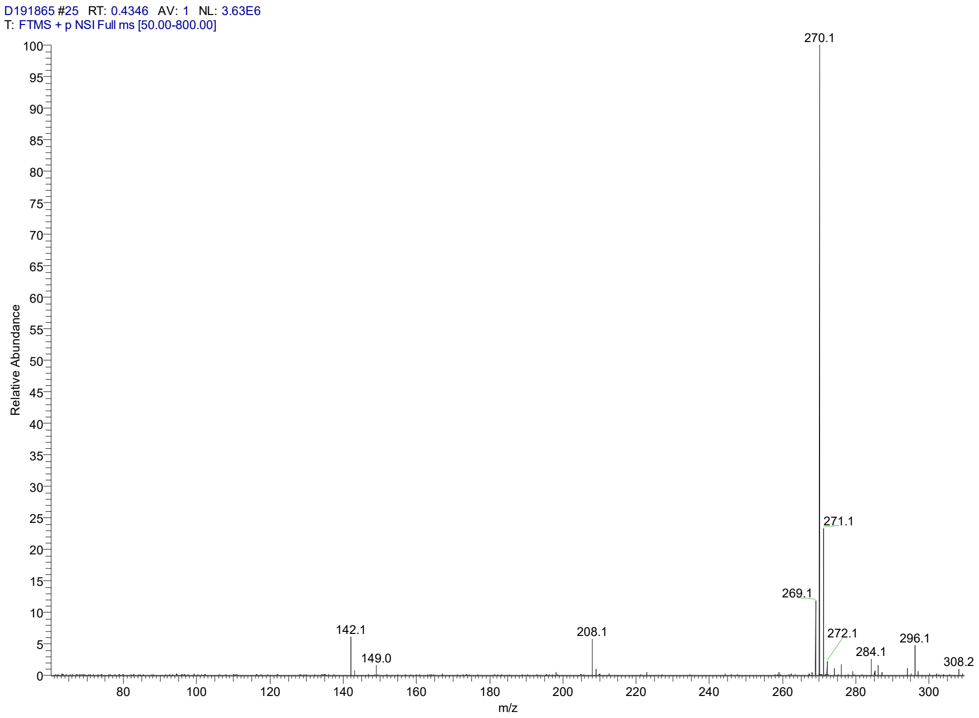

Supplementary Figure 156. HRMS Spectrum of **2i**

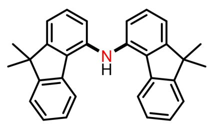

2j

Elemental composition search on mass 402.22

m/z= 397.22-407.22

m/z	Theo. Mass	Delta (ppm)	RDB equiv.	Composition
402.2211	402.2210	0.22	1.5	$C_{16}H_{31}O_5N_3F_3$
	402.2210	0.26	1.0	$C_{19}H_{34}O_2F_4S$
	402.2212	-0.28	5.0	$C_{21}H_{32}O_5F_2$
	402.2210	0.36	8.5	$C_{22}H_{32}O_2N_3S$
	402.2214	-0.76	14.0	$C_{25}H_{27}N_4F$
	402.2215	-0.86	-4.5	$C_{11}H_{37}O_5N_5FS_2$
	402.2216	-1.26	17.5	$C_{30}H_{28}N$
	402.2217	-1.36	-1.0	$C_{16}H_{38}O_5N_2S_2$
	402.2204	1.88	-8.0	$C_{11}H_{38}O_4N_2F_4S_2$
	402.2219	-1.94	0.5	$C_{17}H_{35}N_3F_3S_2$

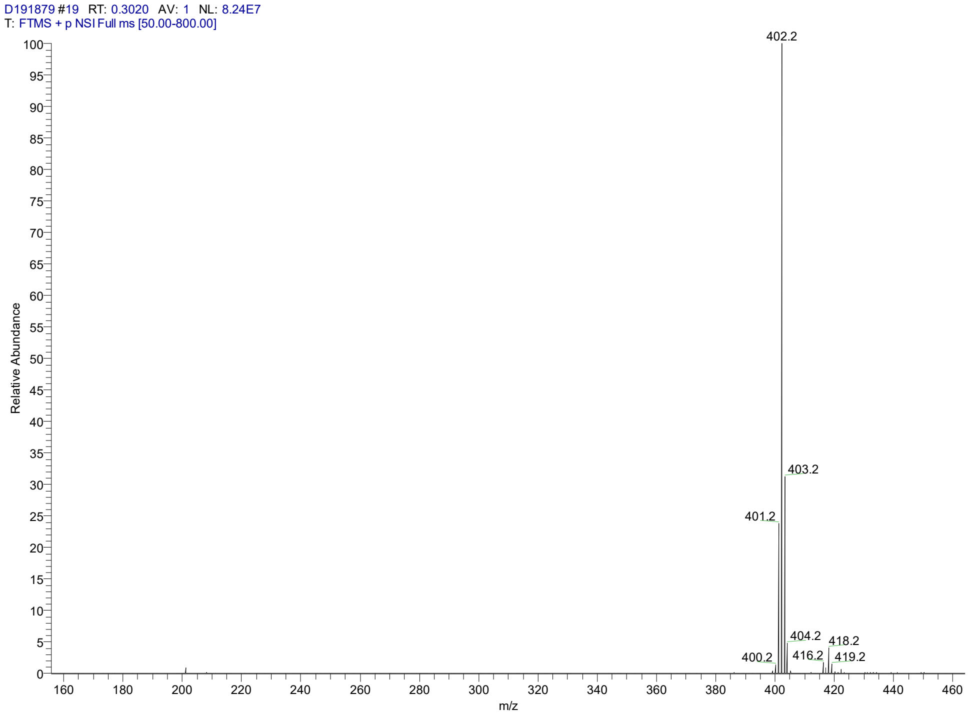

Supplementary Figure 157. HRMS Spectrum of **2j**

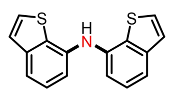

Elemental composition search on mass 282.04

m/z= 277.04-287.04

m/z	Theo. Mass	Delta (ppm)	RDB equiv.	Composition
282.0403	282.0406	-0.77	11.5	$C_{16}H_{12}NS_2$
	282.0397	2.31	12.5	$C_{15}H_8O_5N$
	282.0410	-2.43	17.5	$C_{16}H_4ON_5$
	282.0417	-4.88	8.0	$C_{10}H_{10}O_4N_4S$

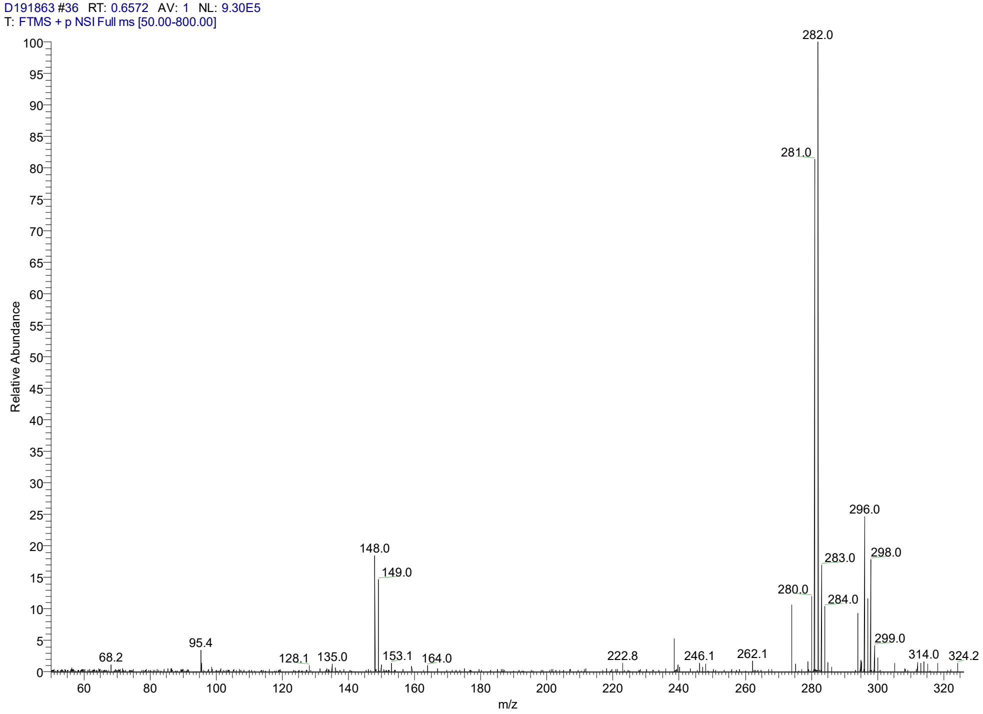

Supplementary Figure 158. HRMS Spectrum of **2k**

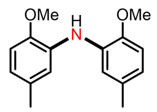

2l

Elemental composition search on mass 258.15

m/z= 253.15-263.15

m/z	Theo. Mass	Delta (ppm)	RDB equiv.	Composition
258.1486	258.1487	-0.06	4.0	$C_{11}H_{19}O_2N_4F$
	258.1489	-0.83	7.5	$C_{16}H_{20}O_2N$
	258.1491	-1.95	-0.5	$C_8H_{22}ON_5ClF$
	258.1480	2.32	-4.0	$C_8H_{23}N_2ClF_4$
	258.1480	2.48	3.5	$C_{11}H_{21}N_5Cl$
	258.1493	-2.72	3.0	$C_{13}H_{23}ON_2Cl$
	258.1478	3.23	-5.5	$C_7H_{26}O_5NClF$
	258.1476	4.01	-9.0	$C_2H_{25}O_5N_4ClF_2$
	258.1476	4.21	0.5	$C_{11}H_{20}ONF_4$
	258.1475	4.37	8.0	$C_{14}H_{18}ON_4$

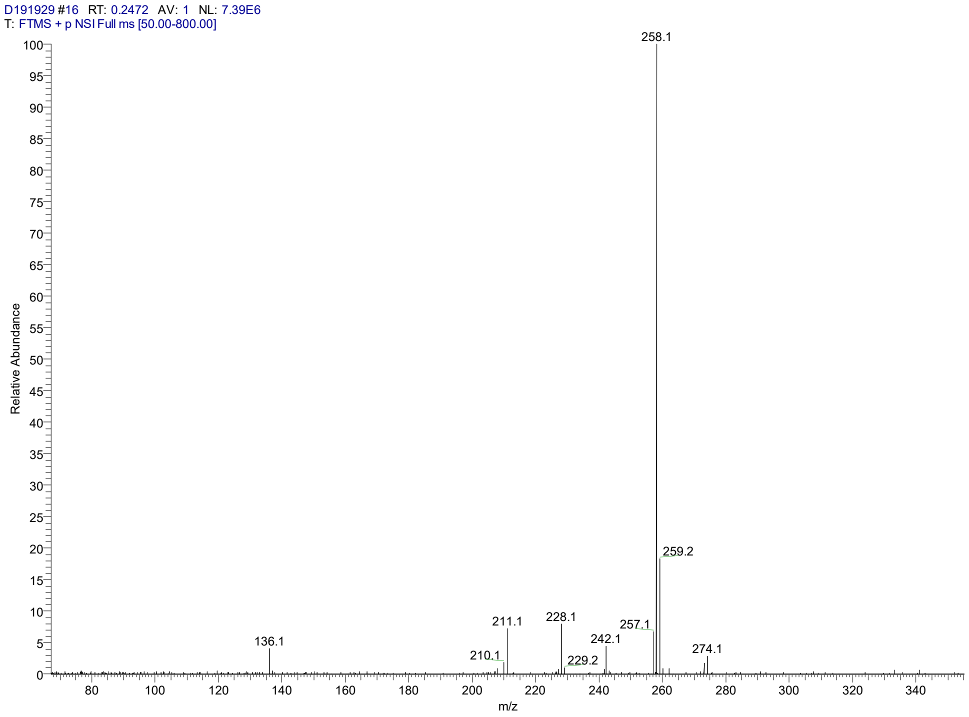

Supplementary Figure 159. HRMS Spectrum of **2l**

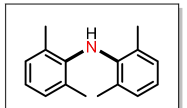

2m

Elemental composition search on mass 226.16

m/z= 221.16-231.16

m/z	Theo. Mass	Delta (ppm)	RDB equiv.	Composition
226.1589	226.1588	0.19	4.0	$C_{11}H_{19}N_4F$
	226.1590	-0.69	7.5	$C_{16}H_{20}N$
	226.1586	1.05	-5.0	$C_7H_{24}O_5F_2$
	226.1593	-1.90	-9.5	$C_3H_{27}N_3F_3S_2$
	226.1584	1.94	-8.5	$C_2H_{23}O_5N_3F_3$
	226.1584	2.01	-9.0	$C_5H_{26}O_2F_4S$
	226.1584	2.19	-1.5	$C_8H_{24}O_2N_3S$
	226.1595	-2.79	-6.0	$C_8H_{28}F_2S_2$
	226.1595	-2.86	-5.5	$C_5H_{25}O_3N_3FS$
	226.1597	-3.74	-2.0	$C_{10}H_{26}O_3S$

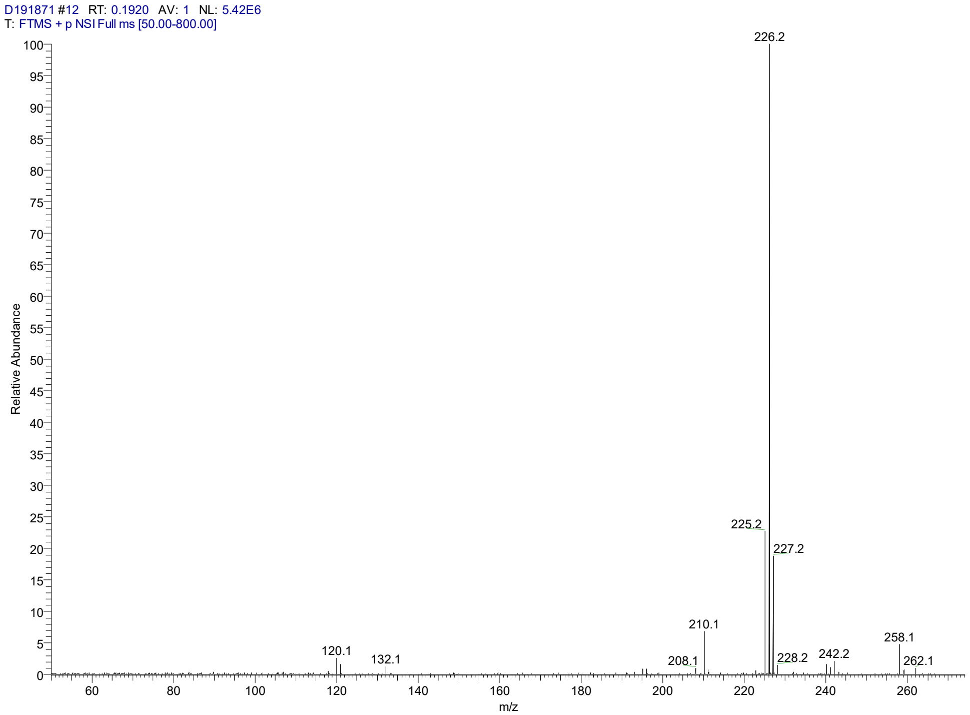

Supplementary Figure 160. HRMS Spectrum of **2m**

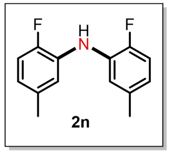

2n

Elemental composition search on mass 234.11

m/z= 229.11-239.11

m/z	Theo. Mass	Delta (ppm)	RDB equiv.	Composition
234.1087	234.1087	-0.10	4.0	$C_9 H_{13} N_4 F_3$
	234.1085	0.73	-5.0	$C_5 H_{18} O_5 F_4$
	234.1084	0.91	2.5	$C_8 H_{16} O_5 N_3$
	234.1089	-0.95	7.5	$C_{14} H_{14} N F_2$
	234.1084	0.98	2.0	$C_{11} H_{19} O_2 F S$
	234.1082	1.83	-1.5	$C_6 H_{18} O_2 N_3 F_2 S$
	234.1093	-2.80	1.5	$C_9 H_{20} N_3 S_2$
	234.1094	-2.97	-6.0	$C_6 H_{22} F_4 S_2$
	234.1094	-3.05	-5.5	$C_3 H_{19} O_3 N_3 F_3 S$
	234.1078	3.77	-7.0	$C_3 H_{23} O_4 N_2 F S_2$

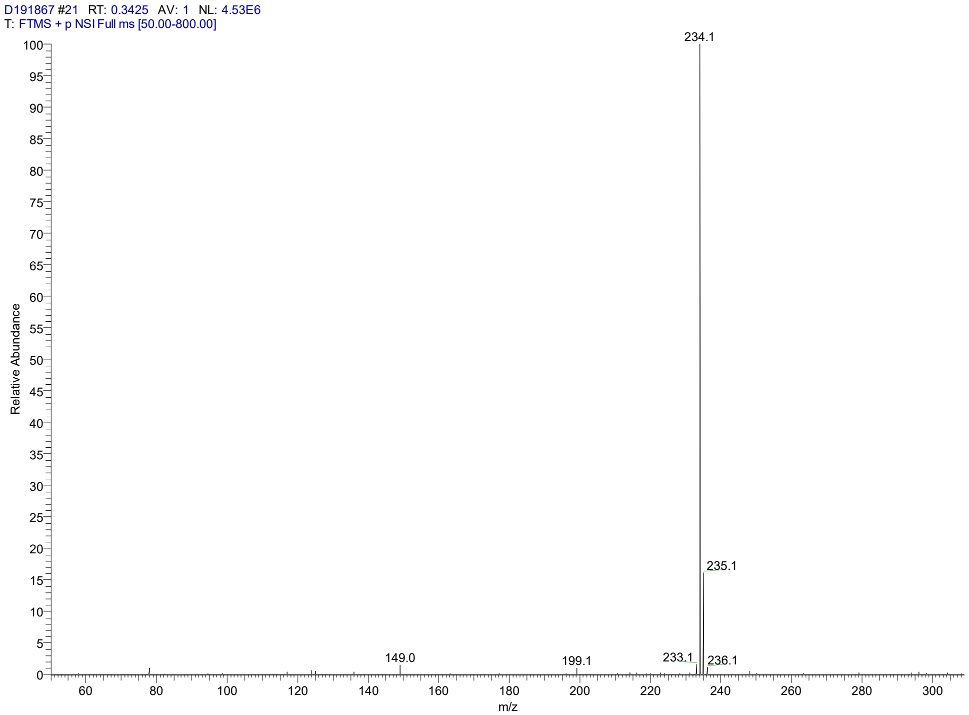

Supplementary Figure 161. HRMS Spectrum of **2n**

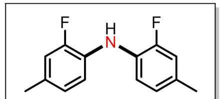

Elemental composition search on mass 234.11

m/z= 229.11-239.11

m/z	Theo. Mass	Delta (ppm)	RDB equiv.	Composition
234.1085	234.1089	-1.51	7.5	$C_{14}H_{14}NF_2$

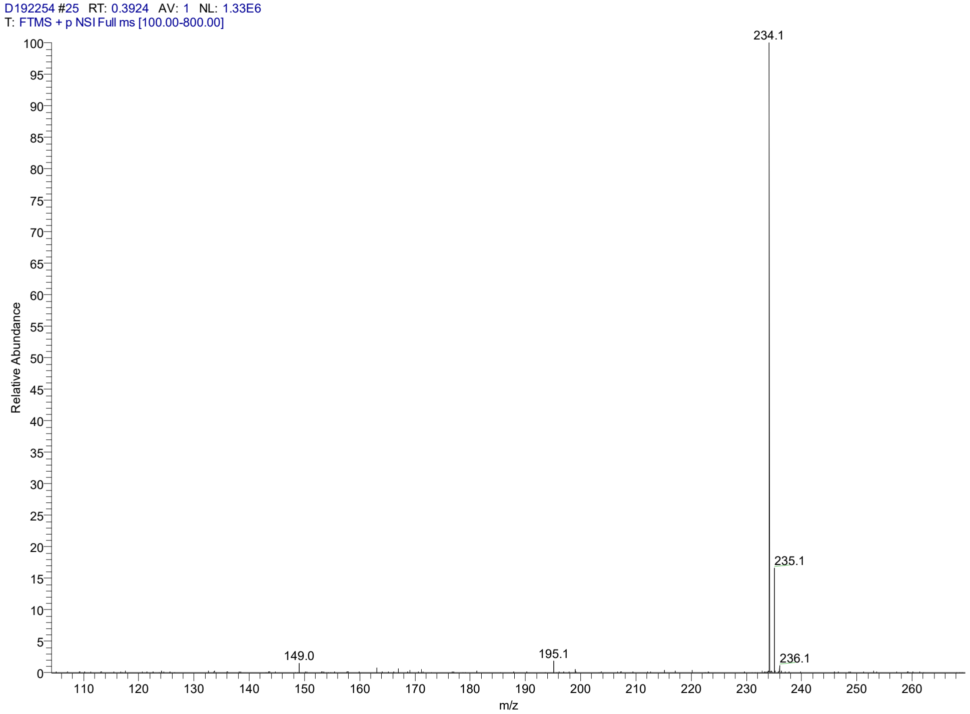

Supplementary Figure 162. HRMS Spectrum of **2o**

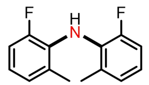

2p

Elemental composition search on mass 234.11

m/z= 229.11-239.11

m/z	Theo. Mass	Delta (ppm)	RDB equiv.	Composition
234.1090	234.1089	0.33	7.5	$C_{14}H_{14}NF_2$
	234.1084	2.19	2.5	$C_8H_{16}O_5N_3$
	234.1082	3.04	-1.0	$C_3H_{15}O_5N_6F$
	234.1098	-3.52	7.5	$C_9H_{12}ON_7$
	234.1098	-3.54	2.0	$C_{10}H_{18}O_6$

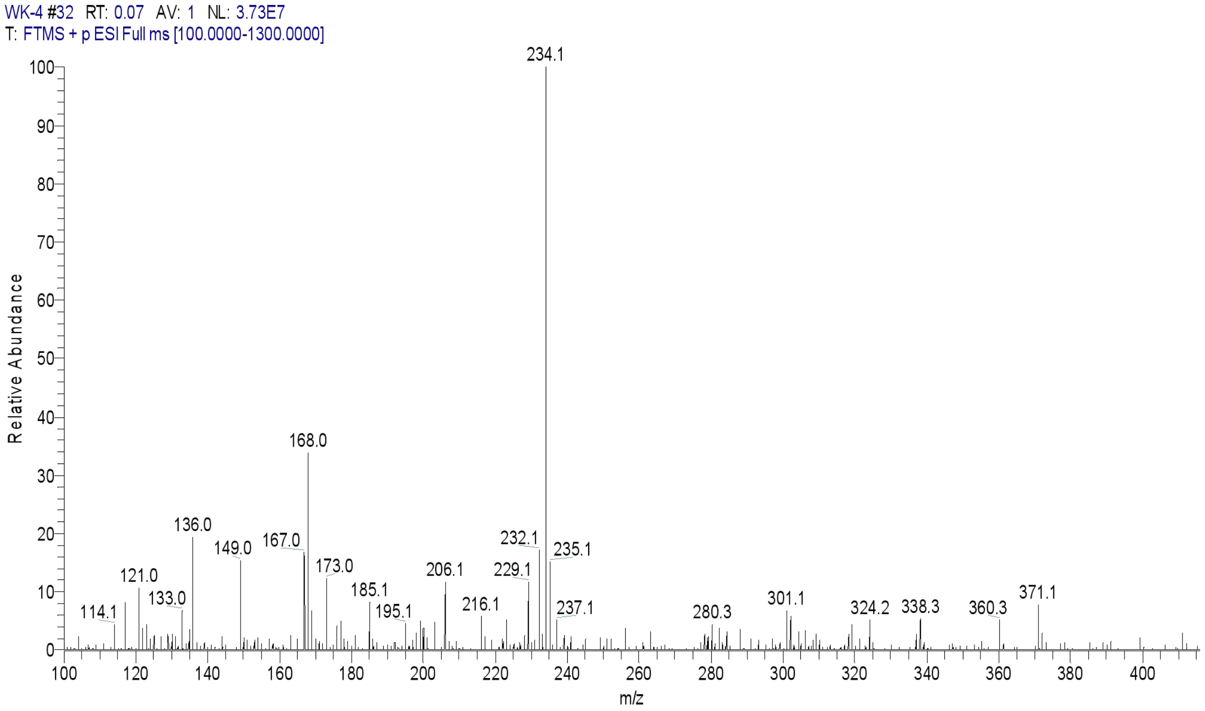

Supplementary Figure 163. HRMS Spectrum of **2p**

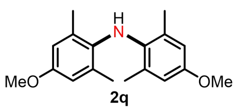

2q

Elemental composition search on mass 286.18

m/z= 281.18-291.18

m/z	Theo. Mass	Delta (ppm)	RDB equiv.	Composition
286.1800	286.1800	0.05	4.0	$C_{13}H_{23}O_2N_4F$
	286.1802	-0.65	7.5	$C_{18}H_{24}O_2N$
	286.1795	1.49	-9.0	$C_7H_{30}O_4F_4S$
	286.1804	-1.61	-9.5	$C_5H_{31}O_2N_3F_3S_2$
	286.1795	1.63	-1.5	$C_{10}H_{28}O_4N_3S$
	286.1795	1.69	-2.0	$C_{13}H_{31}OFS_2$
	286.1806	-2.30	-6.0	$C_{10}H_{32}O_2F_2S_2$
	286.1806	-2.36	-5.5	$C_7H_{29}O_5N_3FS$
	286.1793	2.39	-5.5	$C_8H_{30}ON_3F_2S_2$
	286.1808	-3.06	-2.0	$C_{12}H_{30}O_5S$

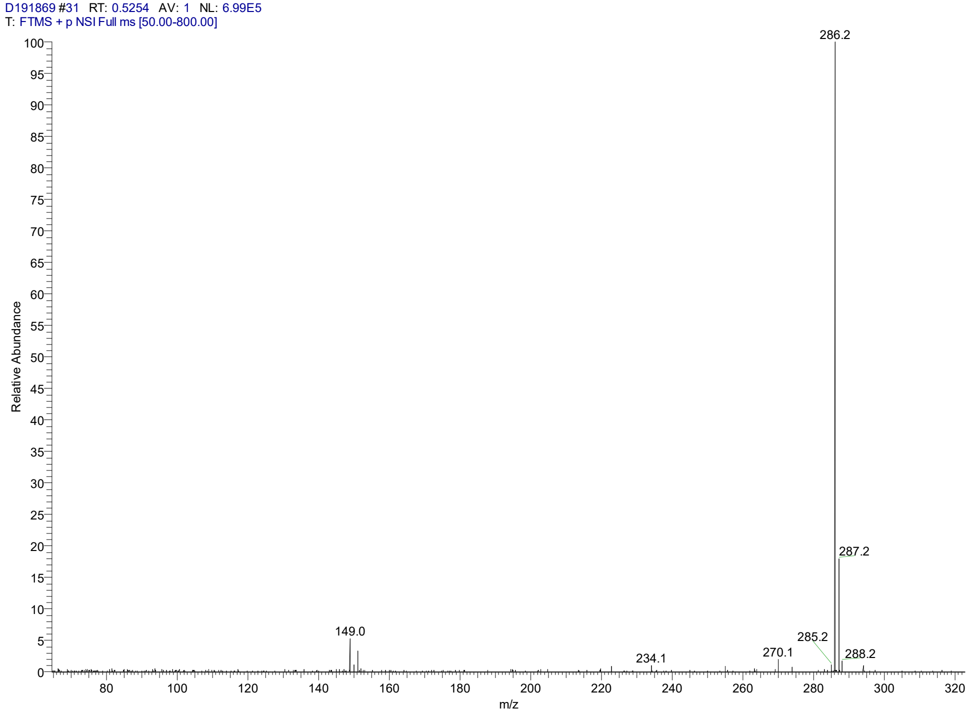

Supplementary Figure 164. HRMS Spectrum of **2q**

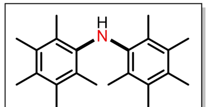

2r

Elemental composition search on mass 310.25

m/z= 305.25-315.25

m/z	Theo. Mass	Delta (ppm)	RDB equiv.	Composition
310.2527	310.2529	-0.57	7.5	$C_{22}H_{32}N$
	310.2532	-1.57	3.0	$C_{18}H_{35}N_2P$

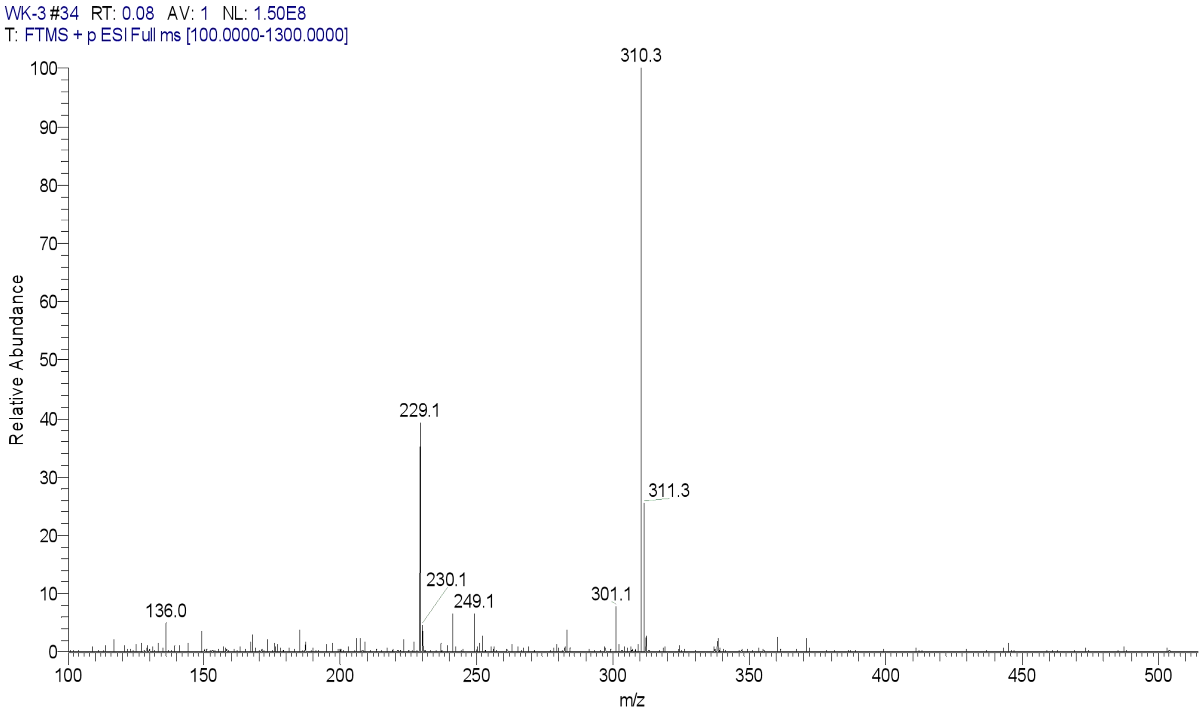

Supplementary Figure 165. HRMS Spectrum of **2r**

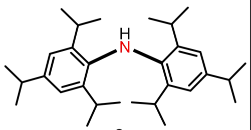

2s

Elemental composition search on mass 422.38

m/z= 417.38-427.38

m/z	Theo. Mass	Delta (ppm)	RDB equiv.	Composition
422.3776	422.3775	0.09	-8.5	$C_{16}H_{51}O_5N_3F_3$
	422.3775	0.13	-9.0	$C_{19}H_{54}O_2F_4S$
	422.3775	0.22	-1.5	$C_{22}H_{52}O_2N_3S$
	422.3777	-0.38	-5.0	$C_{21}H_{52}O_5F_2$
	422.3779	-0.85	4.0	$C_{25}H_{47}N_4F$
	422.3781	-1.32	7.5	$C_{30}H_{48}N$
	422.3784	-1.97	-9.5	$C_{17}H_{55}N_3F_3S_2$
	422.3766	2.32	-1.0	$C_{24}H_{51}O_4F$
	422.3786	-2.44	-6.0	$C_{22}H_{56}F_2S_2$
	422.3786	-2.48	-5.5	$C_{19}H_{53}O_3N_3FS$

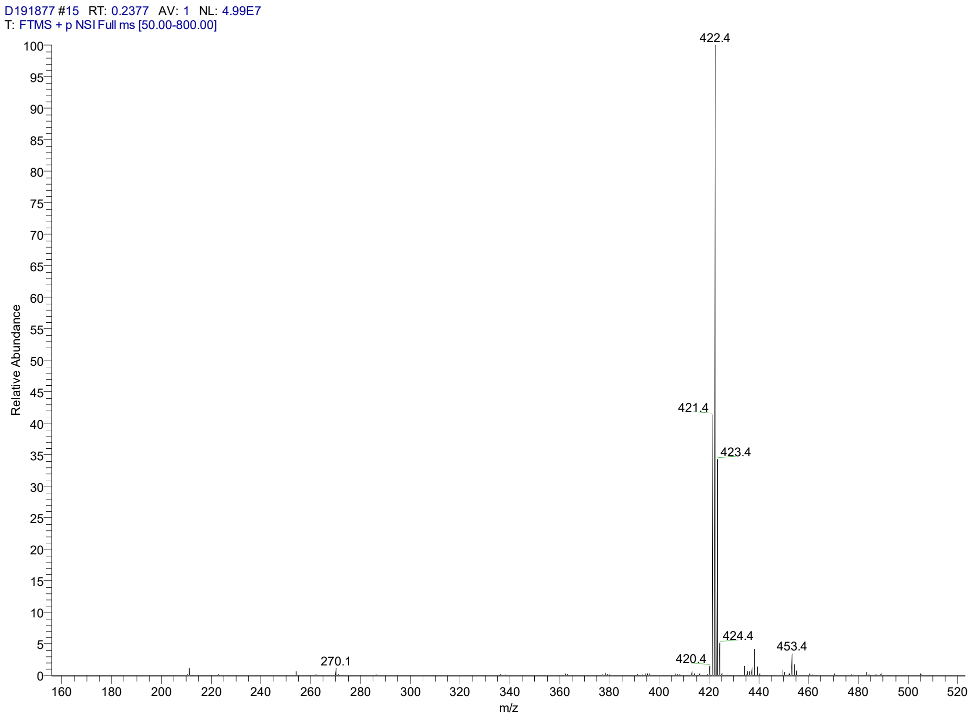

Supplementary Figure 166. HRMS Spectrum of **2s**

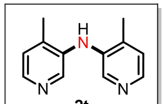

2t

Elemental composition search on mass 200.12

m/z= 195.12-205.12

m/z	Theo. Mass	Delta (ppm)	RDB equiv.	Composition
200.1183	200.1182	0.53	7.5	$C_{12}H_{14}N_3$
	200.1185	-1.02	3.0	$C_8H_{17}N_4P$

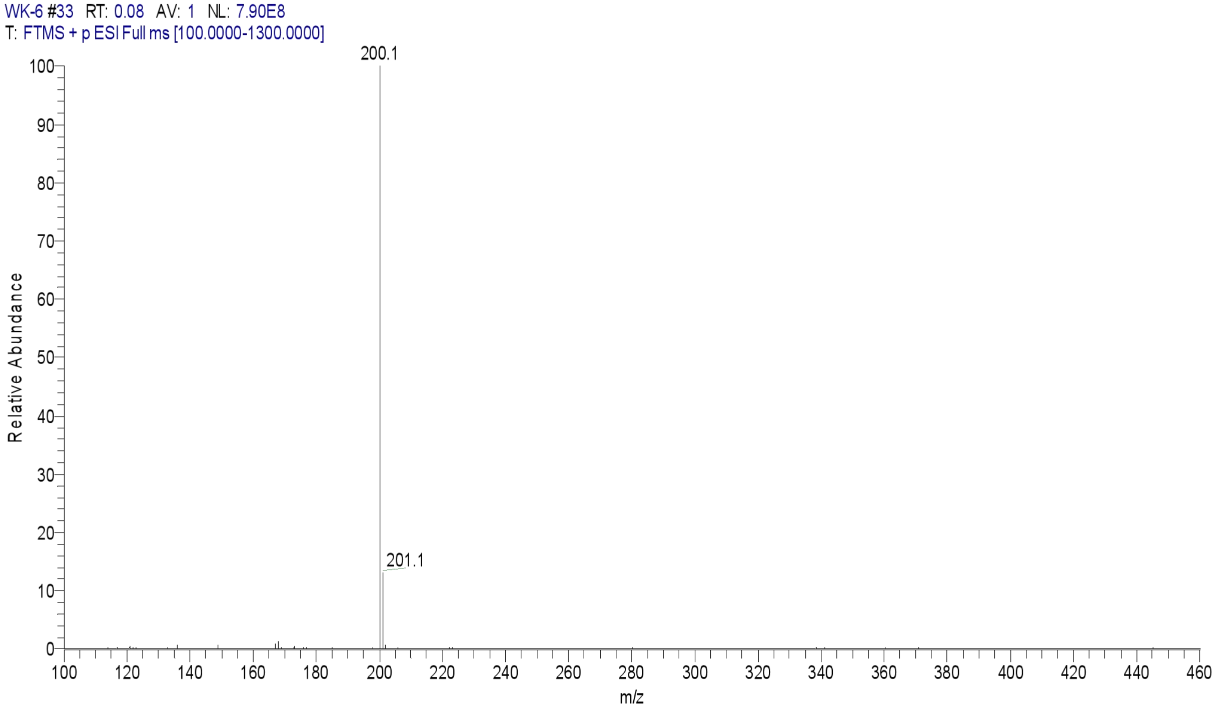

Supplementary Figure 167. HRMS Spectrum of **2t**

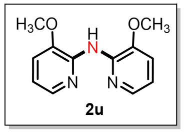

Elemental composition search on mass 232.11

m/z= 227.11-237.11

m/z	Theo. Mass	Delta (ppm)	RDB equiv.	Composition
232.1077	232.1070	3.05	4.0	$C_{12} H_{15} O F_3$

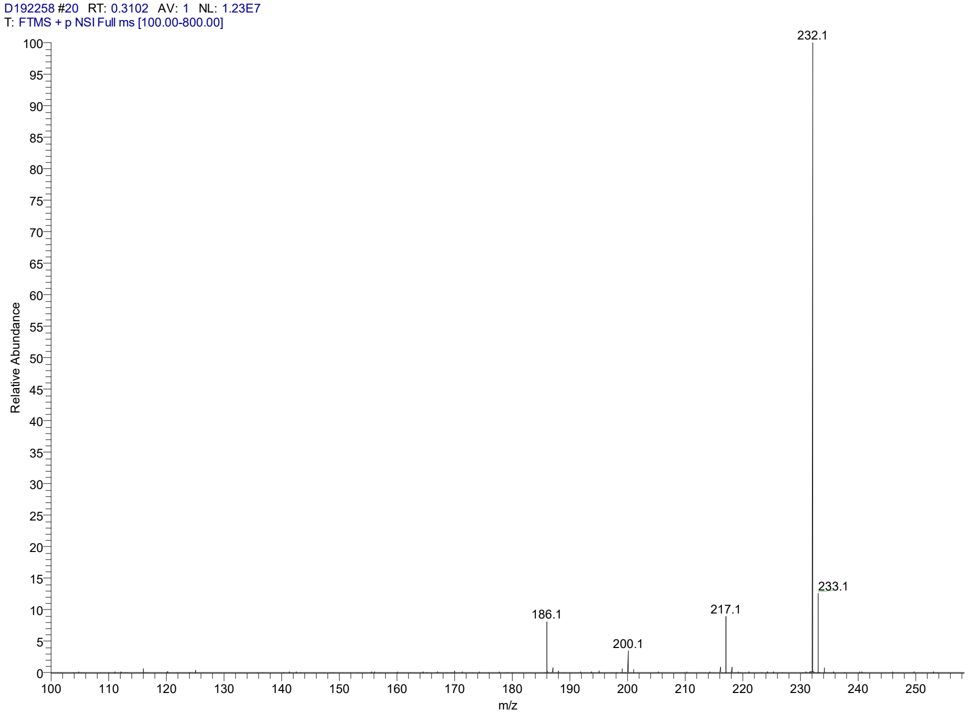

Supplementary Figure 168. HRMS Spectrum of **2u**

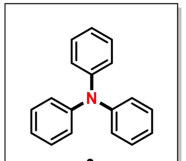

3a

Elemental composition search on mass 246.13

m/z= 241.13-251.13

m/z	Theo. Mass	Delta (ppm)	RDB equiv.	Composition
246.1273	246.1273	0.03	-1.0	$C_9H_{20}O_5F_2$
	246.1275	-0.76	8.0	$C_{13}H_{15}N_4F$
	246.1276	-0.90	-2.0	$C_7H_{23}ON_4ClS$
	246.1271	1.08	2.5	$C_{10}H_{20}O_2N_3S$
	246.1277	-1.57	11.5	$C_{18}H_{16}N$
	246.1278	-1.95	-5.5	$C_6H_{23}O_4NClF_2$
	246.1267	2.68	-10.0	$CH_{24}O_7N_2F_2S$
	246.1267	2.70	-1.5	$C_9H_{22}O_3NClF$
	246.1265	3.51	-5.0	$C_4H_{21}O_3N_4ClF_2$
	246.1282	-3.56	-1.5	$C_7H_{21}O_3N_3FS$

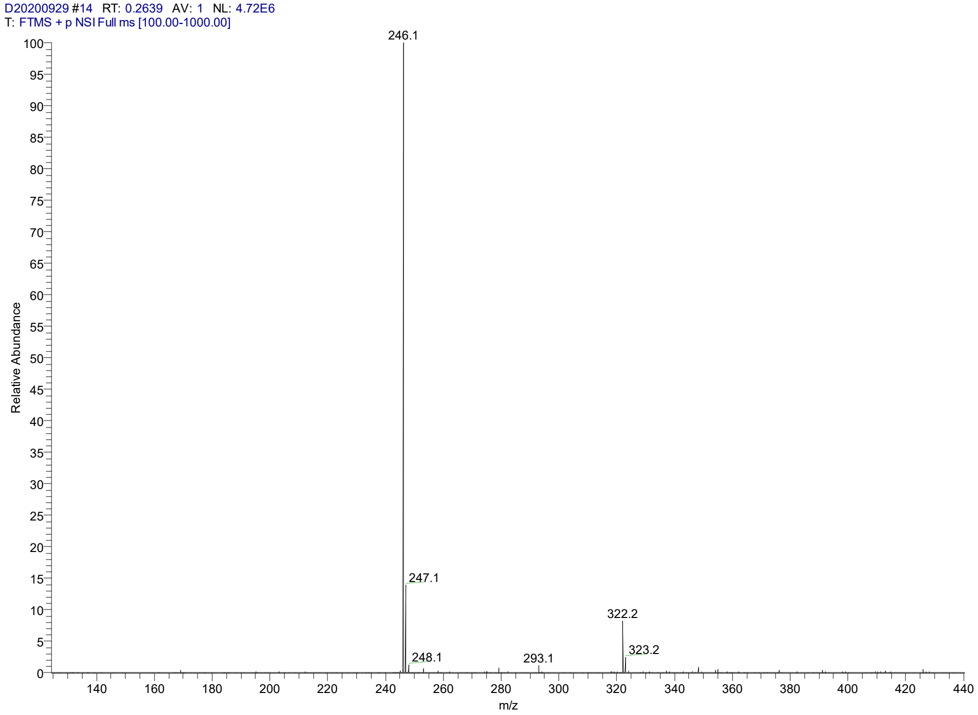

Supplementary Figure 169. HRMS Spectrum of **3a**

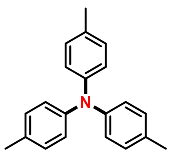

3b

Elemental composition search on mass 288.17

m/z= 283.17-293.17

m/z	Theo. Mass	Delta (ppm)	RDB equiv.	Composition
288.1746	288.1747	-0.09	11.5	$C_{21}H_{22}N$
	288.1745	0.60	8.0	$C_{16}H_{21}N_4F$
	288.1743	1.28	-1.0	$C_{12}H_{26}O_5F_2$
	288.1756	-3.36	4.0	$C_{13}H_{22}ON_4F_2$
	288.1758	-4.06	7.5	$C_{18}H_{23}ONF$

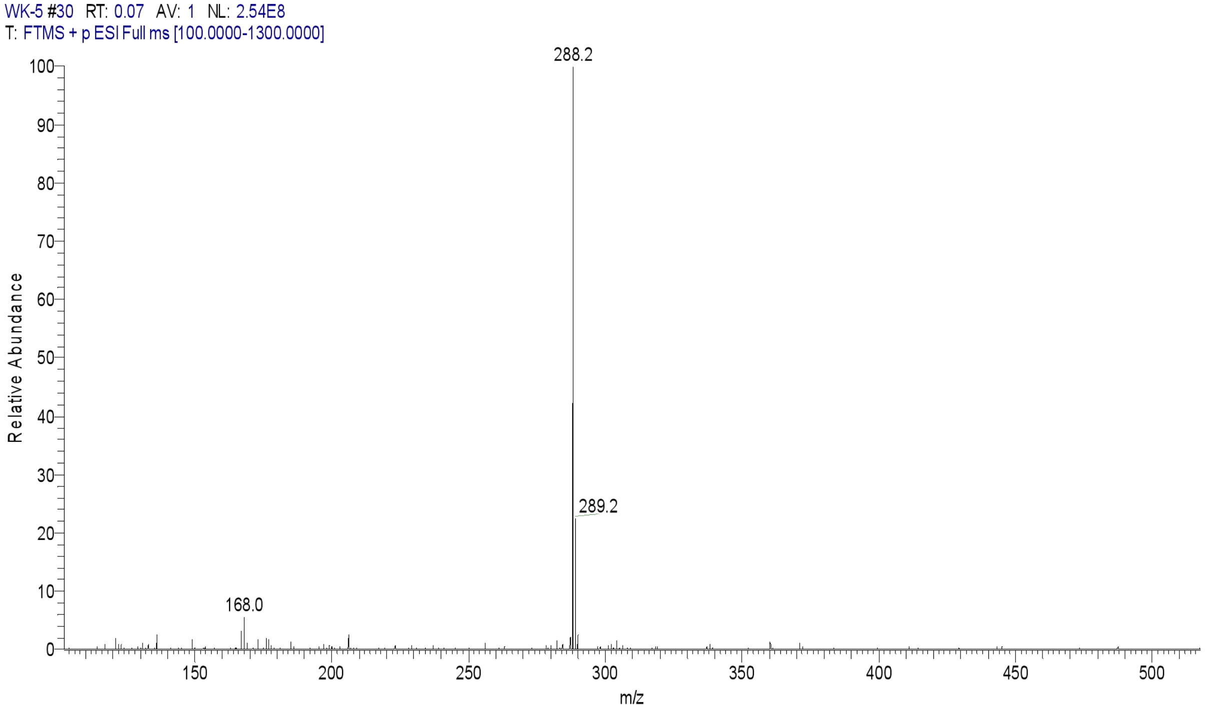

Supplementary Figure 170. HRMS Spectrum of **3b**

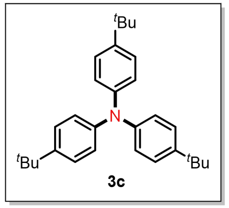

Elemental composition search on mass 414.32

m/z= 409.32-419.32

m/z	Theo. Mass	Delta (ppm)	RDB equiv.	Composition
414.3157	414.3155	0.42	11.5	$C_{30}H_{40}N$
	414.3162	-1.25	2.0	$C_{24}H_{46}O_3S$
	414.3149	1.99	2.5	$C_{22}H_{44}O_2N_3S$

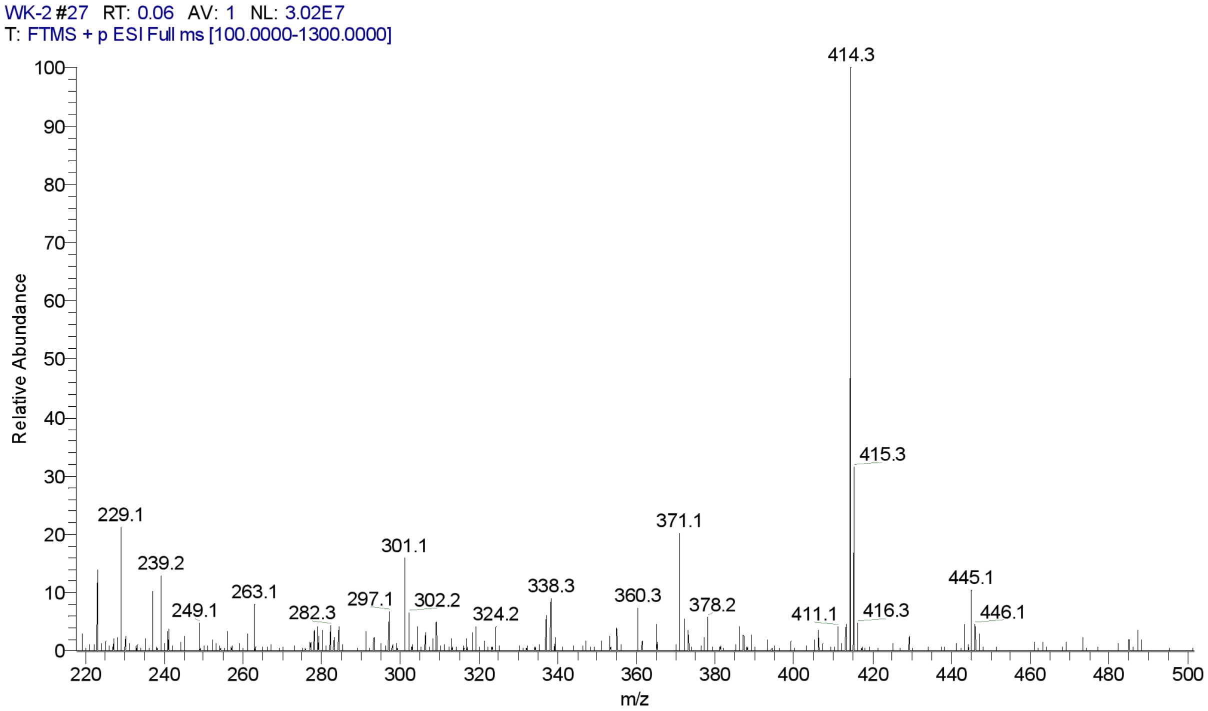

Supplementary Figure 171. HRMS Spectrum of **3c**

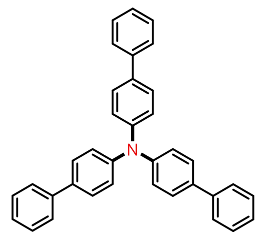

Mass	Intensity	Formula	Calculated Mass	Mass Difference [ppm]	DBE
474.22192	7424.38	C36 H28 N	474.22163	0.63	23.5

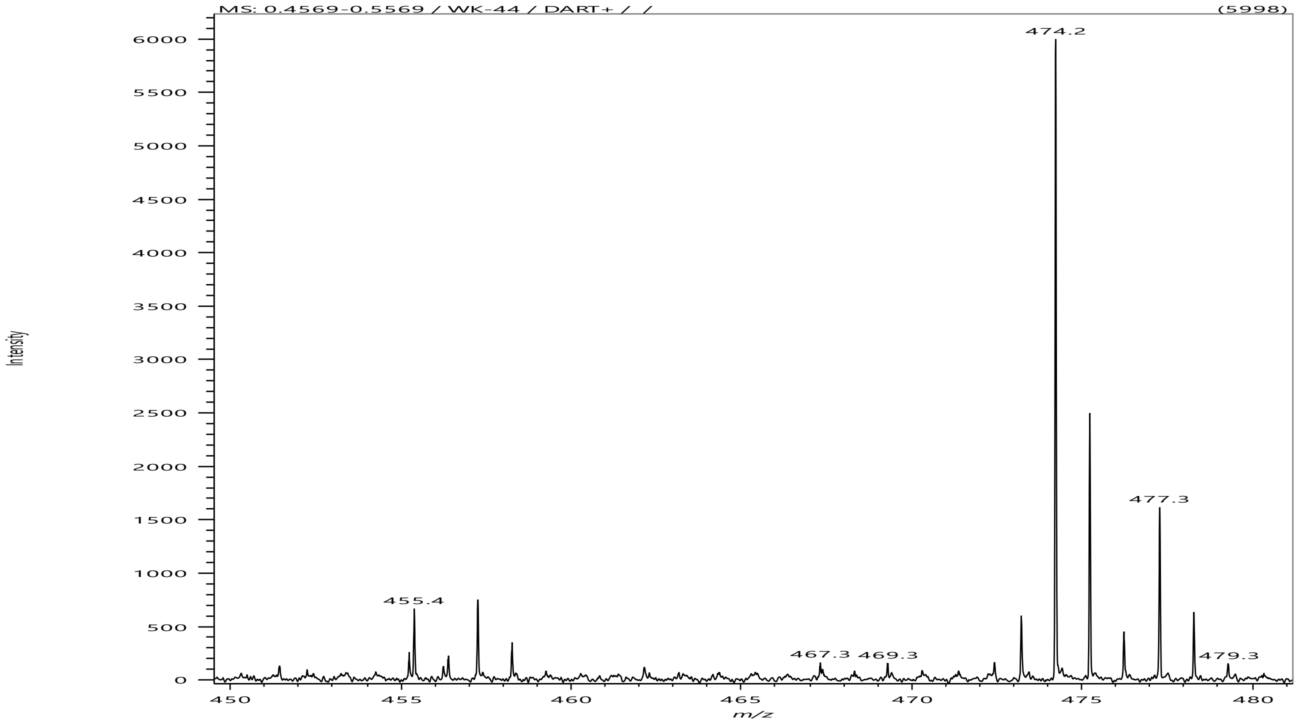

Supplementary Figure 172. HRMS Spectrum of **3d**

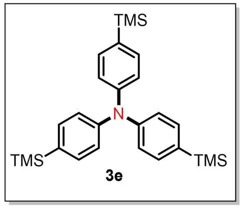

Elemental composition search on mass 462.25

m/z= 457.25-467.25

m/z	Theo. Mass	Delta (ppm)	RDB equiv.	Composition
462.2458	462.2457	0.13	0.5	$C_{17}H_{40}N_5ClF_3Si_2$
	462.2459	-0.30	4.0	$C_{22}H_{41}N_2ClF_2Si_2$
	462.2461	-0.66	8.0	$C_{22}H_{39}N_4FSi_3$
	462.2463	-1.10	11.5	$C_{27}H_{40}NSi_3$
	462.2464	-1.25	14.5	$C_{27}H_{30}N_5F_2$
	462.2466	-1.68	18.0	$C_{32}H_{31}N_2F$
	462.2443	3.23	12.5	$C_{28}H_{37}NFSi_2$
	462.2441	3.66	9.0	$C_{23}H_{36}N_4F_2Si_2$
	462.2439	4.02	5.0	$C_{23}H_{38}N_2ClF_3Si$
	462.2477	-4.19	-0.5	$C_{16}H_{43}N_5ClF_2Si_3$

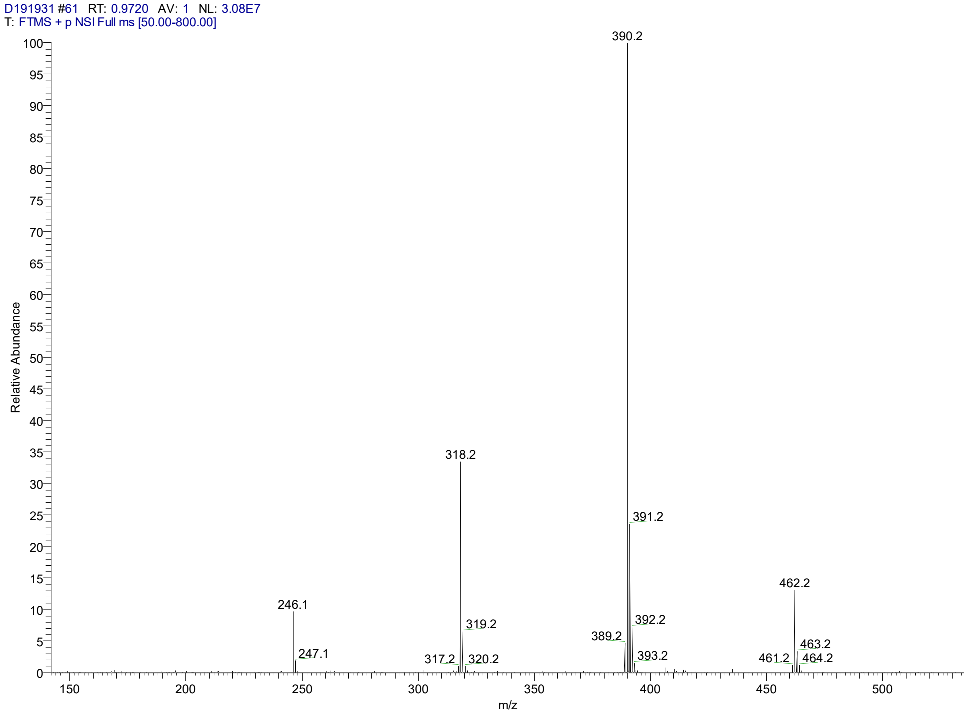

Supplementary Figure 173. HRMS Spectrum of **3e**

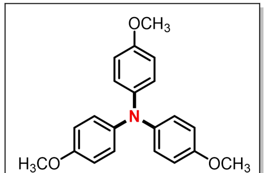

3f

Elemental composition search on mass 336.16

m/z= 331.16-341.16

m/z	Theo. Mass	Delta (ppm)	RDB equiv.	Composition
336.1591	336.1594	-0.86	11.5	$C_{21}H_{22}O_3N$
	336.1606	-4.26	7.5	$C_{18}H_{23}O_4NF$

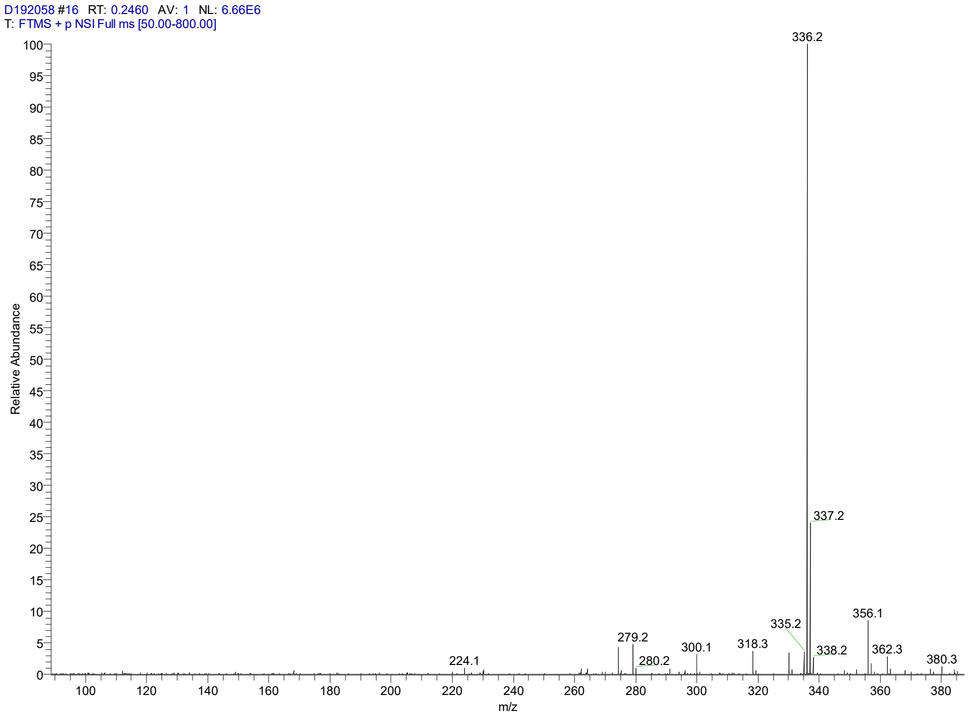

Supplementary Figure 174. HRMS Spectrum of **3f**

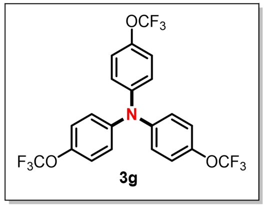

Elemental composition search on mass 497.07

m/z= 492.07-502.07

m/z	Theo. Mass	Delta (ppm)	RDB equiv.	Composition
497.0663	497.0668	-0.92	12.0	$C_{21}H_{12}O_3NF_9$
	497.0658	1.06	27.0	$C_{32}H_{10}O_2NF_3$
	497.0670	-1.24	23.0	$C_{29}H_{11}O_3NF_4$
	497.0657	1.38	16.0	$C_{24}H_{11}O_2NF_8$
	497.0647	3.36	31.0	$C_{35}H_9ONF_2$
	497.0681	-3.54	19.0	$C_{26}H_{12}O_4NF_5$
	497.0645	3.68	20.0	$C_{27}H_{10}ONF_7$
	497.0683	-3.86	30.0	$C_{34}H_{11}O_4N$
	497.0643	4.15	18.5	$C_{26}H_{13}O_6F_4$
	497.0641	4.47	7.5	$C_{18}H_{14}O_6F_9$

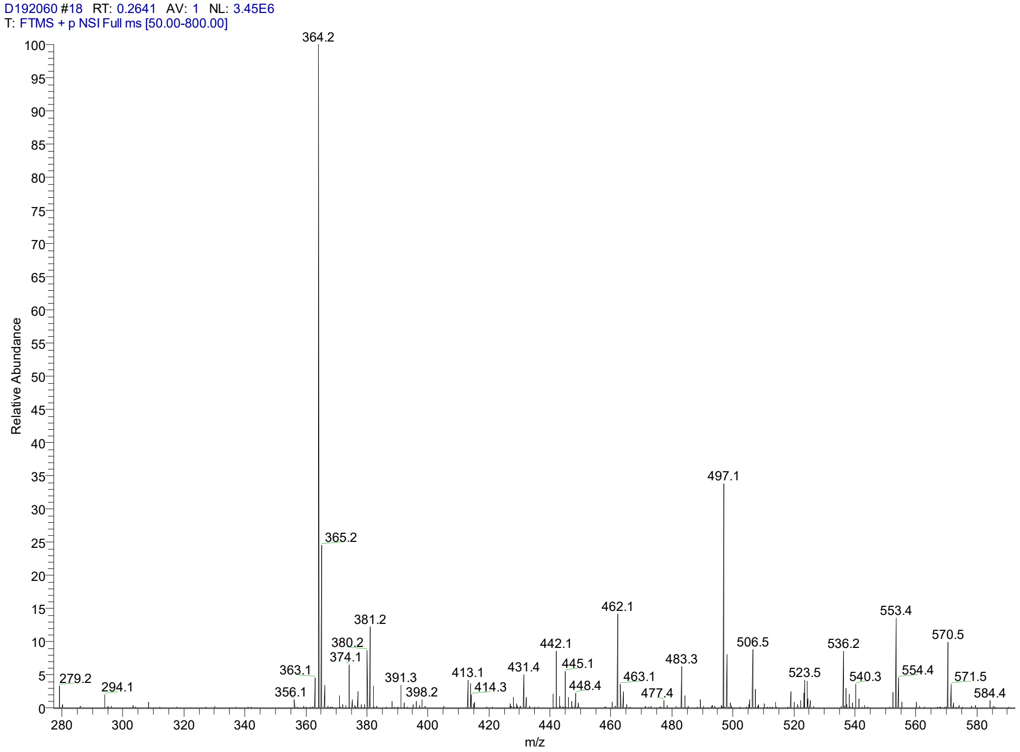

Supplementary Figure 175. HRMS Spectrum of **3g**

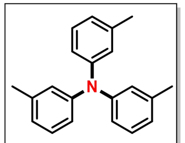

3h

Elemental composition search on mass 288.17

m/z= 283.17-293.17

m/z	Theo. Mass	Delta (ppm)	RDB equiv.	Composition
288.1743	288.1747	-1.31	11.5	$C_{21} H_{22} N$

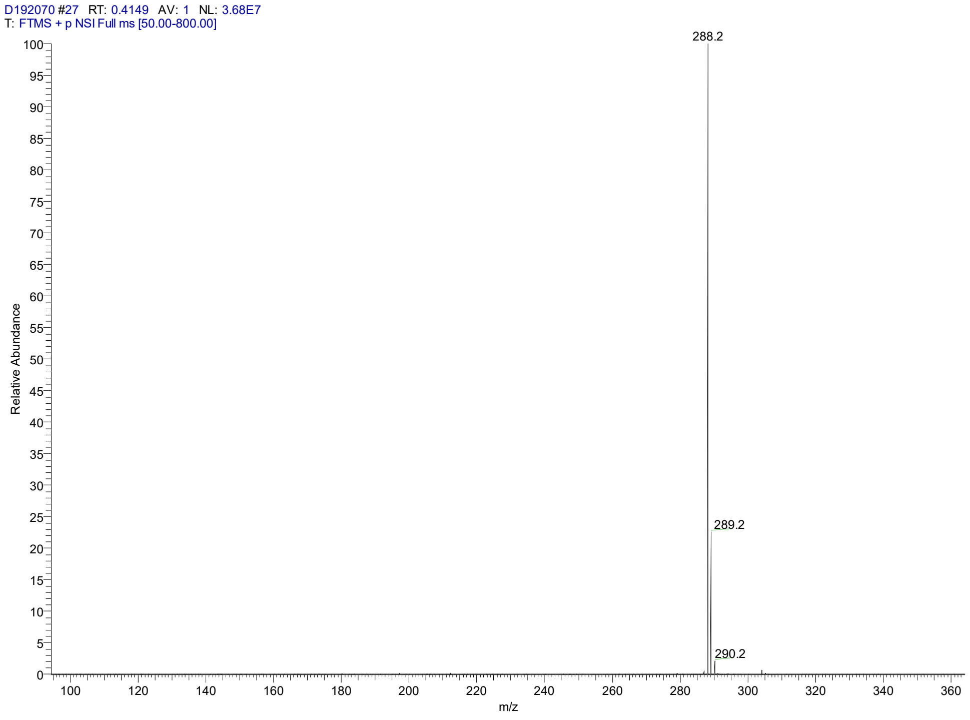

Supplementary Figure 176. HRMS Spectrum of **3h**

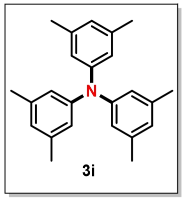

Elemental composition search on mass 330.22

m/z= 325.22-335.22

m/z	Theo. Mass	Delta (ppm)	RDB equiv.	Composition
330.2215	330.2216	-0.29	11.5	$C_{24}H_{28}N$
	330.2219	-1.23	7.0	$C_{20}H_{31}N_2P$
	330.2222	-2.17	2.5	$C_{16}H_{34}N_3P_2$

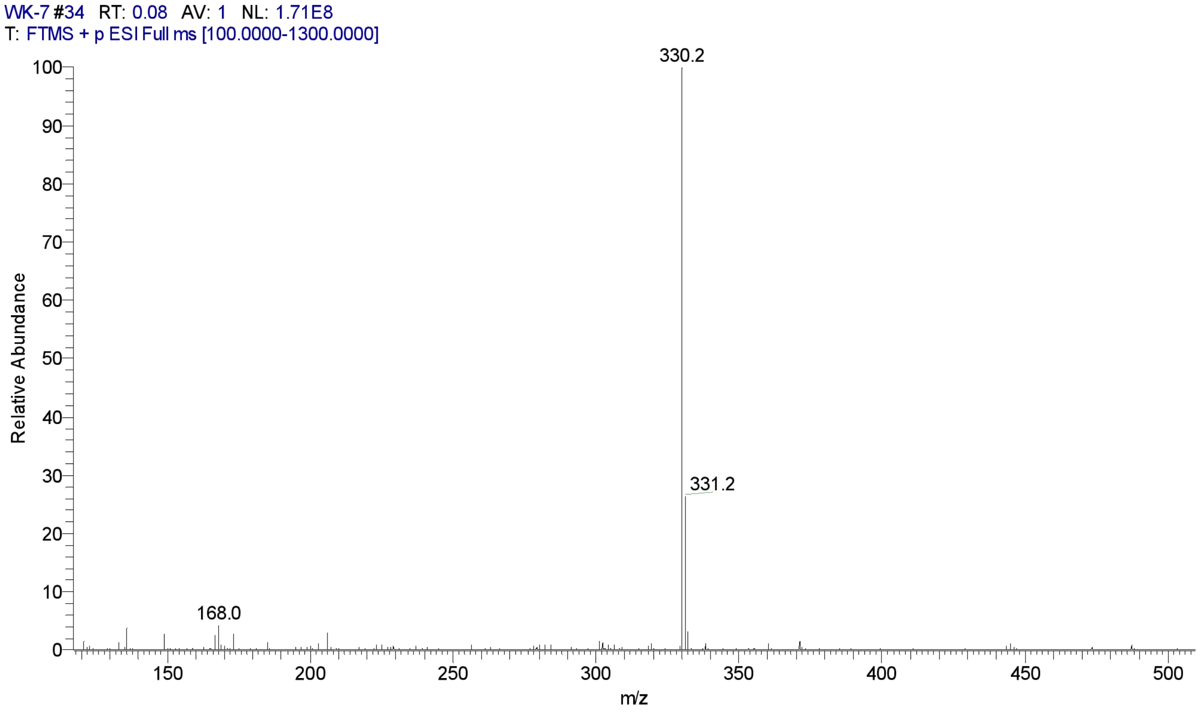

Supplementary Figure 177. HRMS Spectrum of **3i**

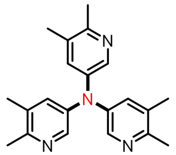

3j

Elemental composition search on mass 333.21

m/z= 328.21-338.21

m/z	Theo. Mass	Delta (ppm)	RDB equiv.	Composition
333.2071	333.2074	-0.91	11.5	$C_{21}H_{25}N_4$
	333.2060	3.10	6.5	$C_{20}H_{29}O_4$
	333.2087	-4.94	11.0	$C_{23}H_{27}ON$

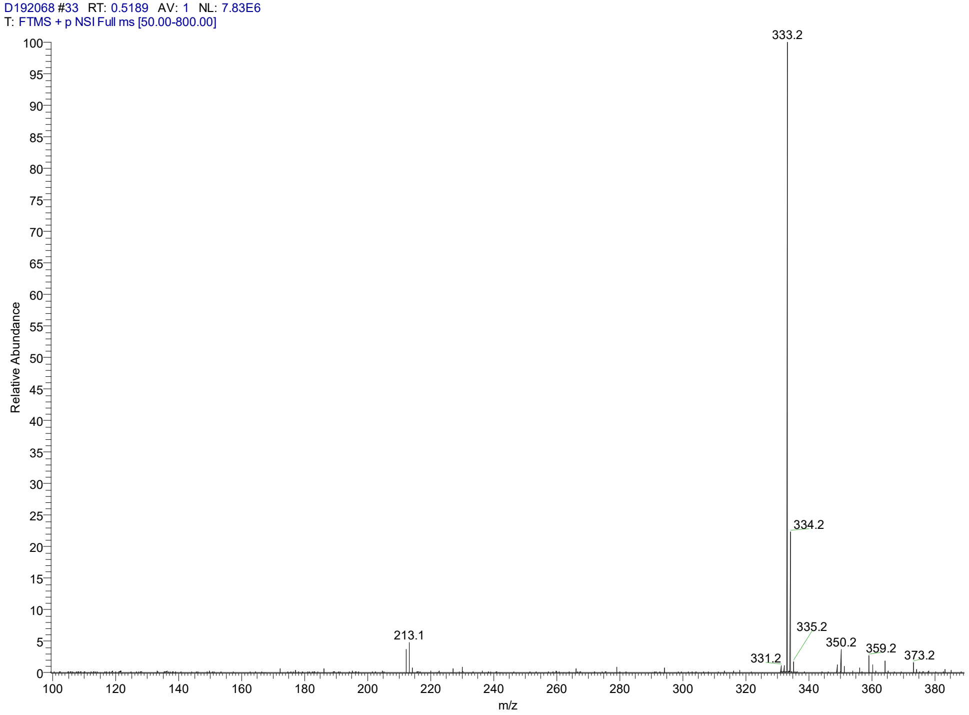

Supplementary Figure 178. HRMS Spectrum of **3j**

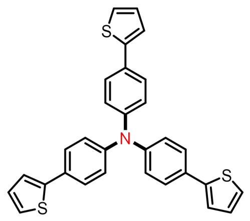

3k

Mass	Intensity	Formula	Calculated Mass	Mass Difference [ppm]	DBE
492.09087	1826.88	C30 H22 N S3	492.09089	-0.04	20.5

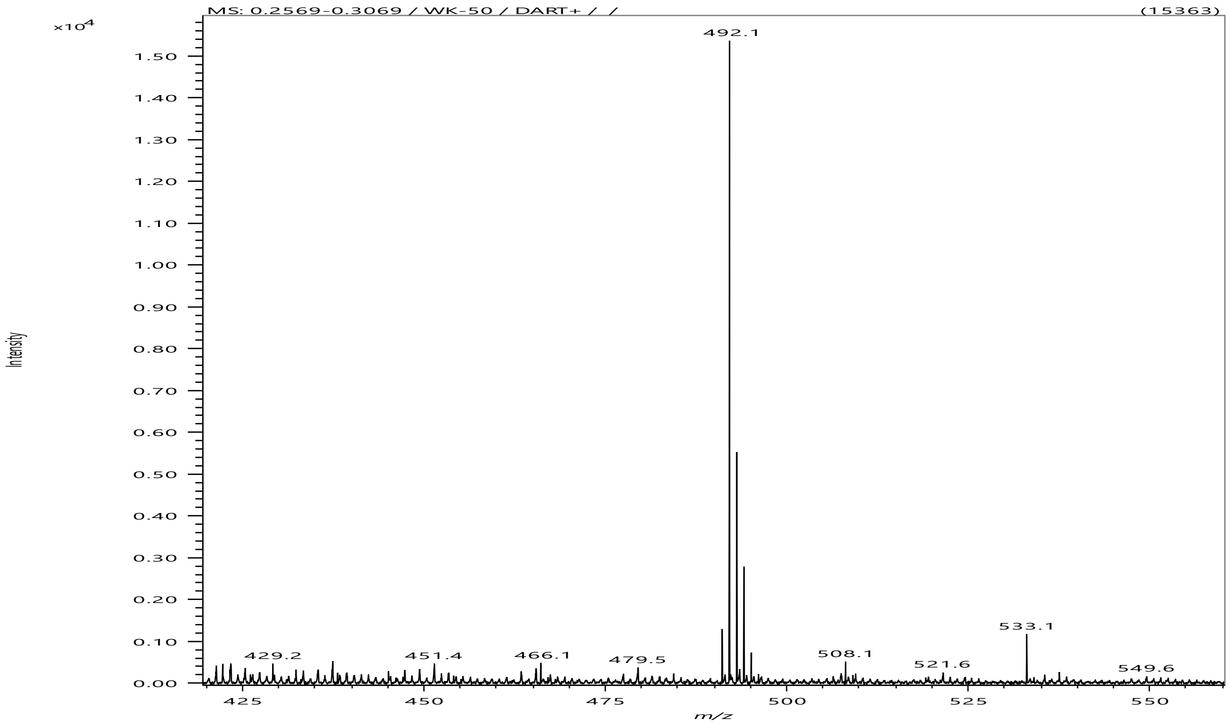

Supplementary Figure 179. HRMS Spectrum of **3k**

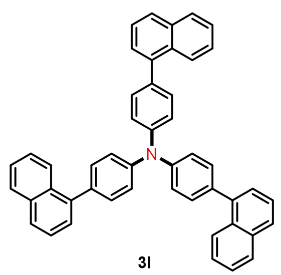

3l

Mass	Intensity	Formula	Calculated Mass	Mass Difference [ppm]	DBE
624.26682	659.00	C48 H34 N	624.26858	-2.82	32.5

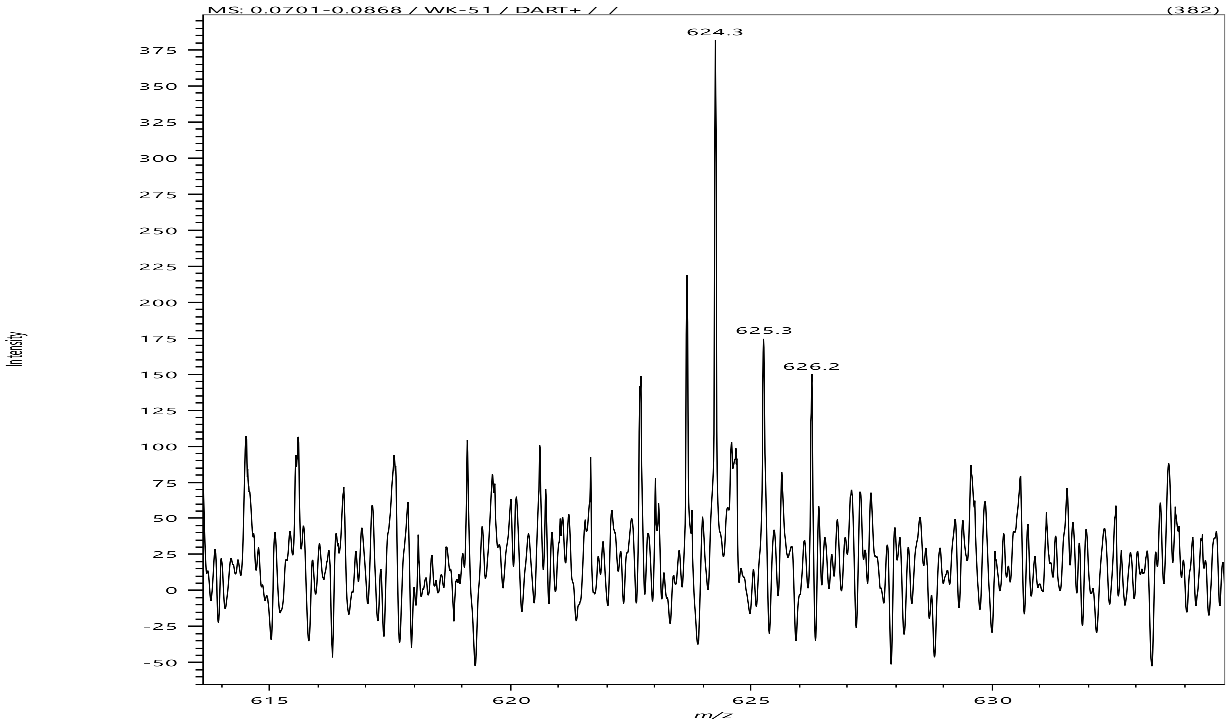

Supplementary Figure 180. HRMS Spectrum of **3l**

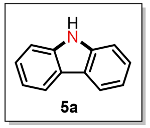

Elemental composition search on mass 168.08

m/z= 163.08-173.08

m/z	Theo. Mass	Delta (ppm)	RDB equiv.	Composition
168.0807	168.0806	0.50	5.0	$C_7H_9N_4F$
	168.0808	-0.69	8.5	$C_{12}H_{10}N$
	168.0809	-1.24	-8.5	$H_{17}O_4NClF_2$
	168.0804	1.66	-4.0	$C_3H_{14}O_5F_2$

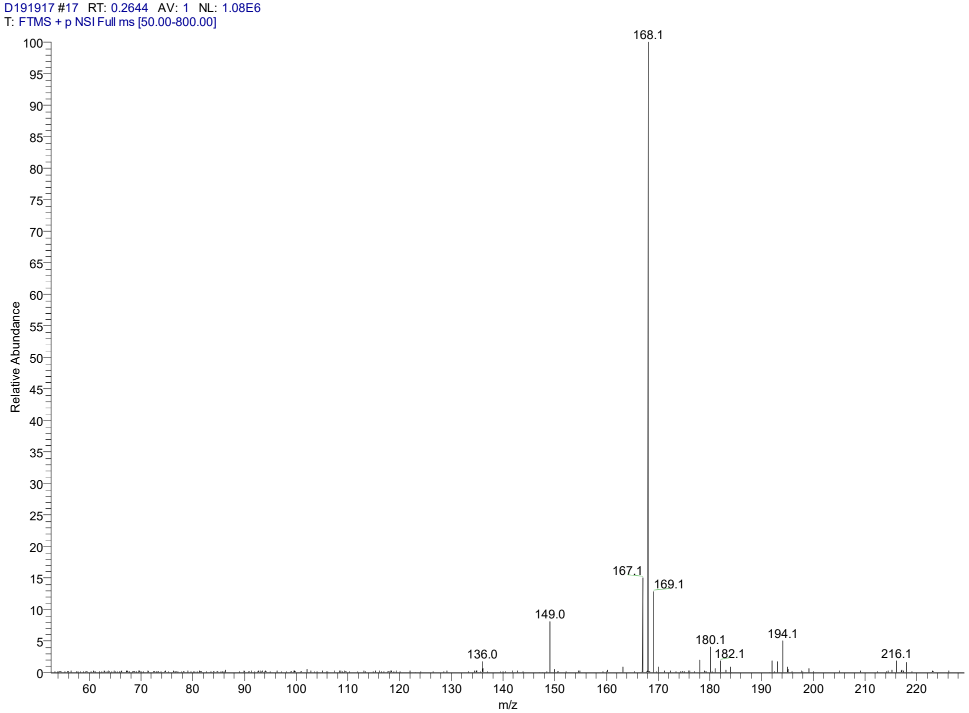

Supplementary Figure 181. HRMS Spectrum of **5a**

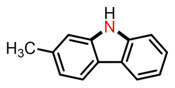

5b

Elemental composition search on mass 182.10

m/z= 177.10-187.10

m/z	Theo. Mass	Delta (ppm)	RDB equiv.	Composition
182.0963	182.0962	0.30	5.0	$C_8H_{11}N_4F$
	182.0964	-0.80	8.5	$C_{13}H_{12}N$
	182.0965	-1.31	-8.5	$CH_{19}O_4NClF_2$
	182.0960	1.36	-4.0	$C_4H_{16}O_5F_2$
	182.0954	4.97	-4.5	$C_4H_{18}O_3NClF$

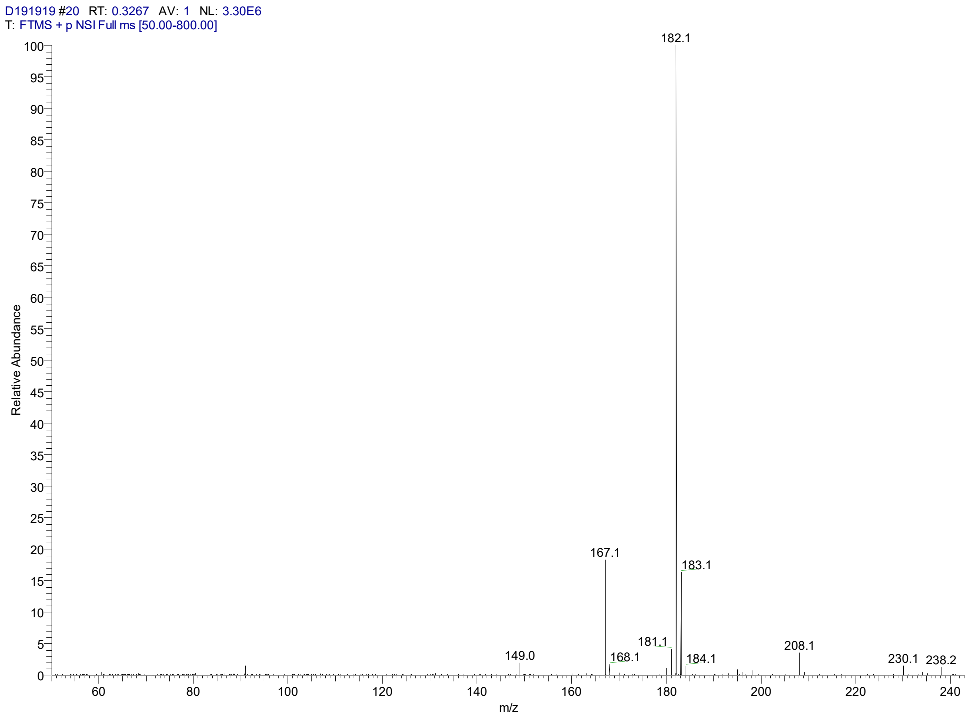

Supplementary Figure 182. HRMS Spectrum of **5b**

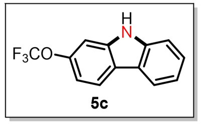

Elemental composition search on mass 252.06

m/z= 247.06-257.06

m/z	Theo. Mass	Delta (ppm)	RDB equiv.	Composition
252.0629	252.0629	0.10	5.0	$C_8H_8ON_4F_4$
	252.0631	-0.69	8.5	$C_{13}H_9ONF_3$
	252.0631	-0.90	-1.0	$C_4H_{14}O_5N_4ClF$
	252.0633	-1.69	2.5	$C_9H_{15}O_5NCl$
	252.0634	-1.83	0.5	$C_5H_{11}N_5ClF_4$
	252.0636	-2.63	4.0	$C_{10}H_{12}N_2ClF_3$
	252.0620	3.47	-4.5	$C_4H_{15}O_4NClF_4$
	252.0620	3.63	3.0	$C_7H_{13}O_4N_4Cl$
	252.0619	3.84	12.5	$C_{16}H_8NF_2$
	252.0617	4.63	9.0	$C_{11}H_7N_4F_3$

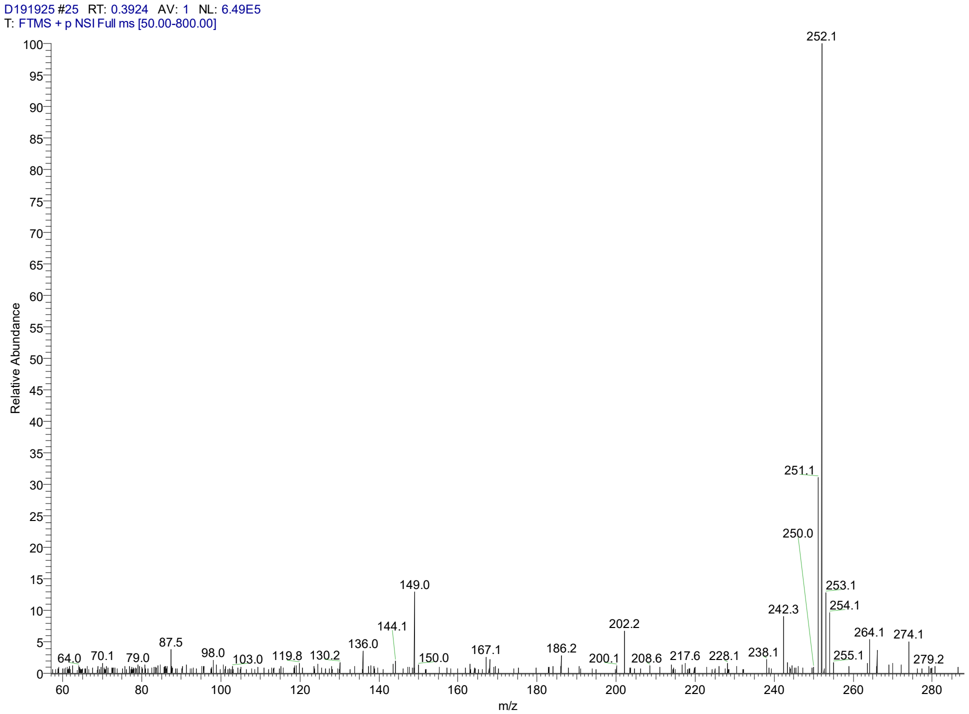

Supplementary Figure 183. HRMS Spectrum of **5c**

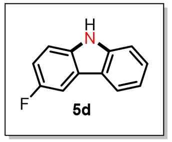

Elemental composition search on mass 186.07

m/z= 181.07-191.07

m/z	Theo. Mass	Delta (ppm)	RDB equiv.	Composition
186.0712	186.0712	0.46	5.0	$C_7H_8N_4F_2$
	186.0714	-0.61	8.5	$C_{12}H_9NF$
	186.0714	-1.11	-8.5	$H_{16}O_4NClF_3$
	186.0710	1.51	-4.0	$C_3H_{13}O_5F_3$

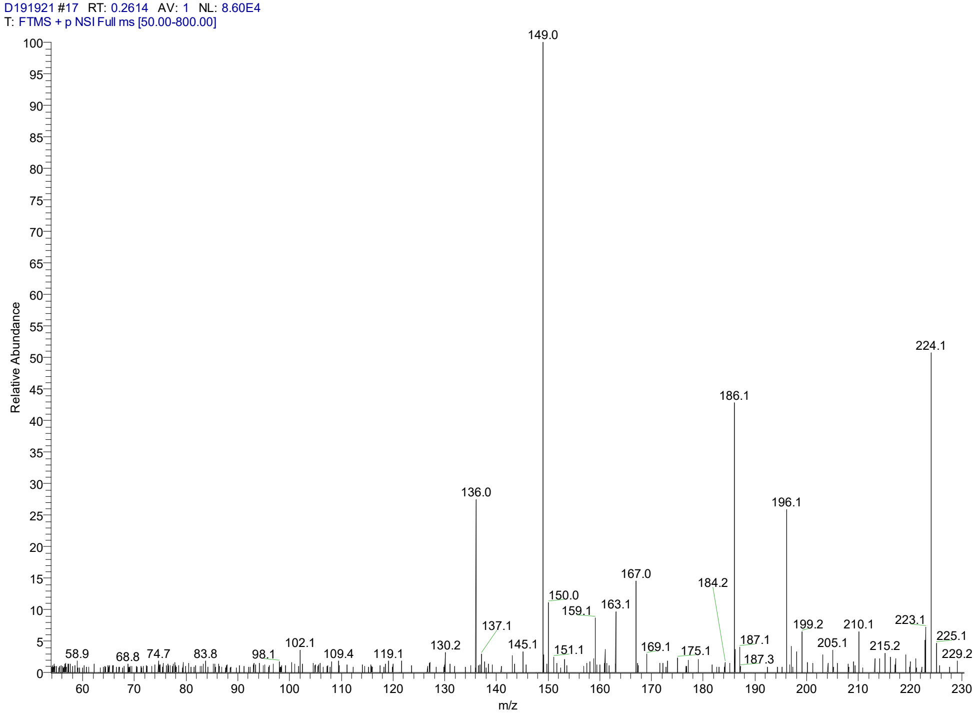

Supplementary Figure 184. HRMS Spectrum of **5d**

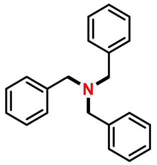

7

Elemental composition search on mass 288.17

m/z= 283.17-293.17

m/z	Theo. Mass	Delta (ppm)	RDB equiv.	Composition
288.1747	288.1747	0.01	11.5	$C_{21}H_{22}N$
	288.1752	-1.75	-1.0	$C_7H_{24}O_6N_6$
	288.1740	2.28	2.5	$C_{13}H_{26}O_2N_3S$
	288.1754	-2.38	2.0	$C_{15}H_{28}O_3S$

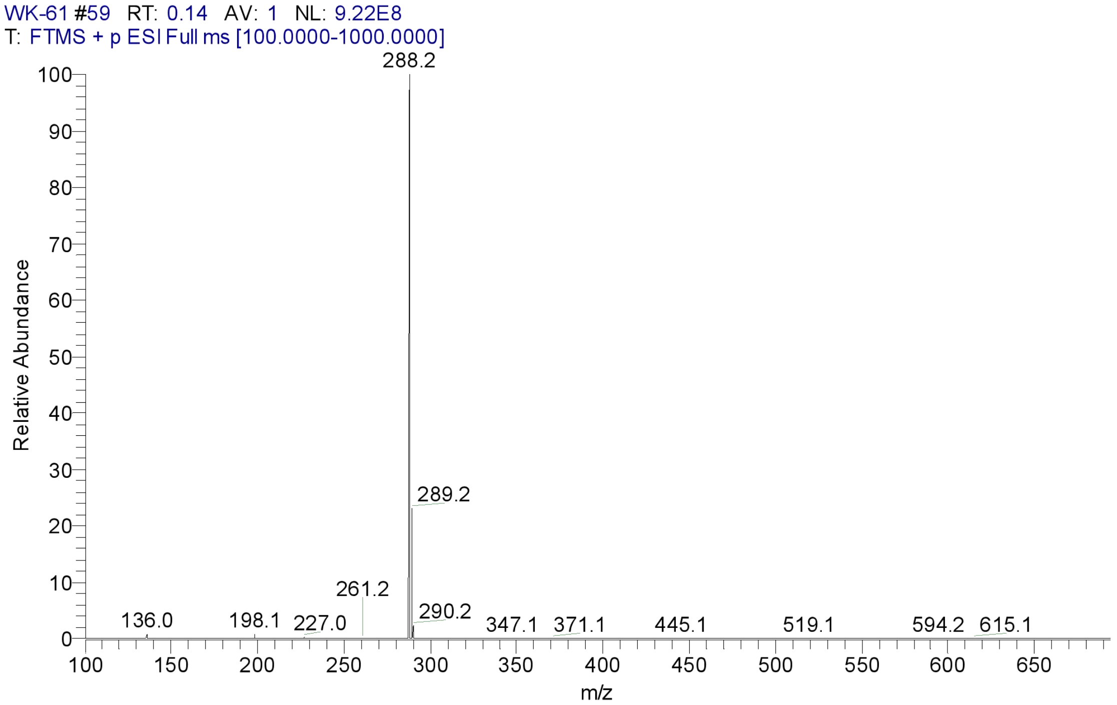

Supplementary Figure 185. HRMS Spectrum of **7**

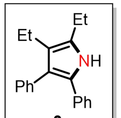

9

Elemental composition search on mass 276.17

m/z= 271.17-281.17

m/z	Theo. Mass	Delta (ppm)	RDB equiv.	Composition
276.1748	276.1747	0.59	10.5	$C_{20}H_{22}N$
	276.1752	-1.25	-2.0	$C_6H_{24}O_6N_6$
	276.1754	-1.91	1.0	$C_{14}H_{28}O_3S$
	276.1740	2.95	1.5	$C_{12}H_{26}O_2N_3S$

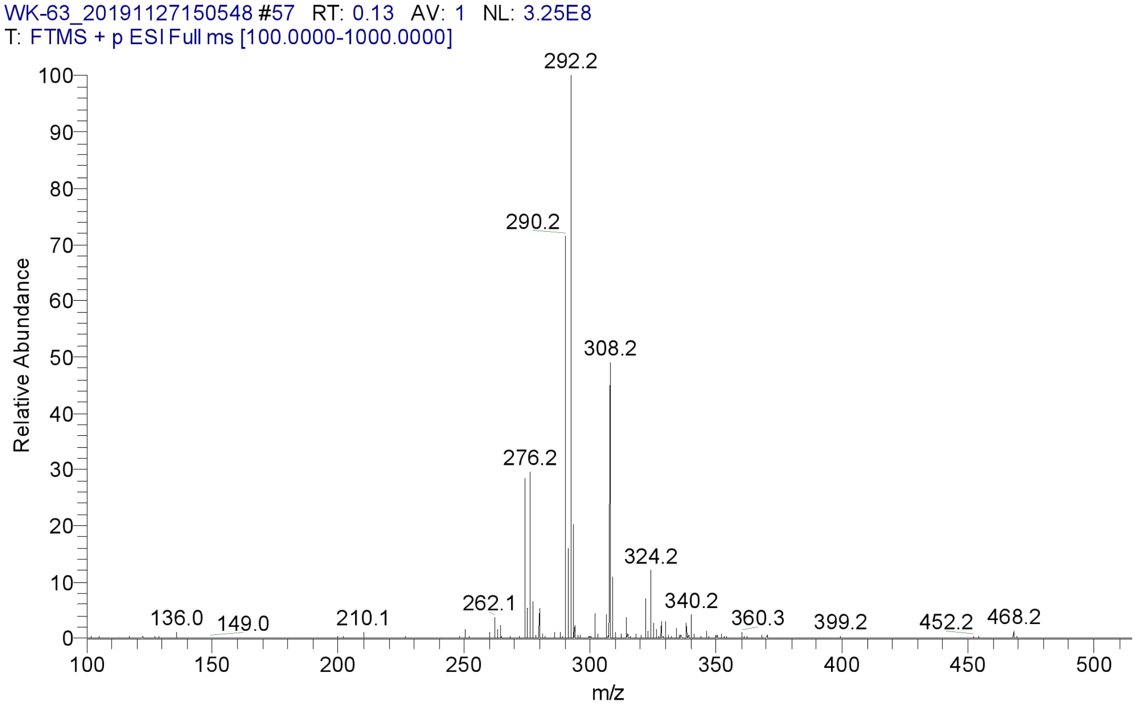

Supplementary Figure 186. HRMS Spectrum of **9**

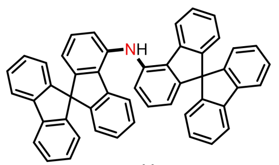

11

Elemental composition search on mass 646.25

m/z= 641.25-651.25

m/z	Theo. Mass	Delta (ppm)	RDB equiv.	Composition
646.2532	646.2529	0.45	35.5	$C_{50} H_{32} N$
	646.2548	-2.41	22.5	$C_{38} H_{36} O_7 N_3$
	646.2561	-4.49	22.0	$C_{40} H_{38} O_8$
	646.2502	4.60	31.0	$C_{47} H_{34} O_3$

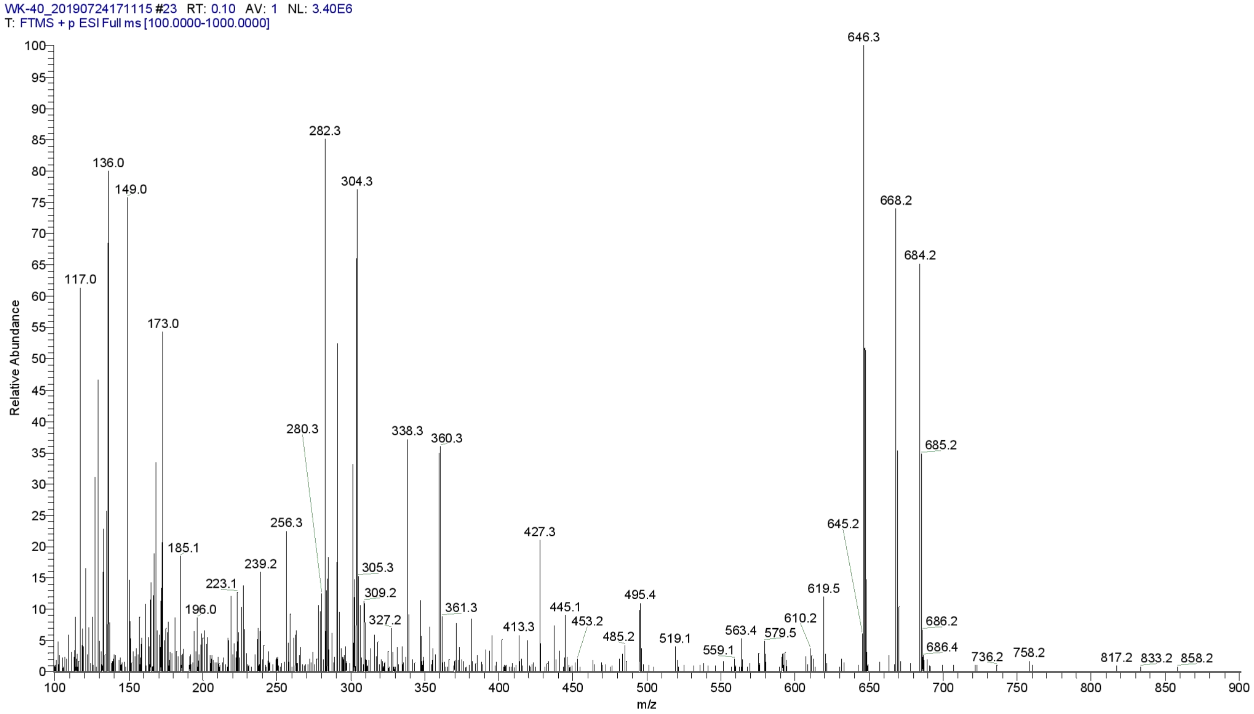

Supplementary Figure 187. HRMS Spectrum of **11**

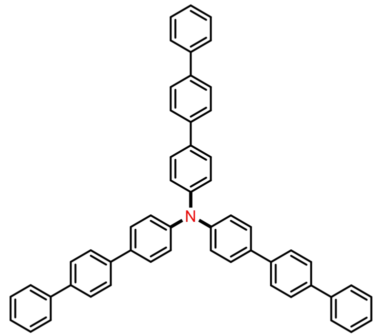

13

Mass	Intensity	Formula	Calculated Mass	Mass Difference [ppm]	DBE
702.31870	214.09	C54 H40 N	702.31553	4.51	35.5

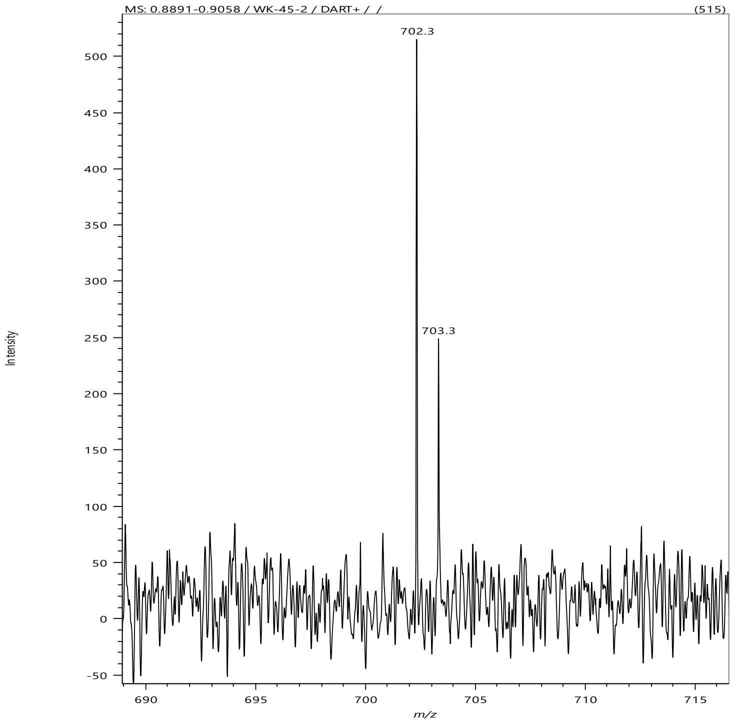

Supplementary Figure 188. HRMS Spectrum of **13**

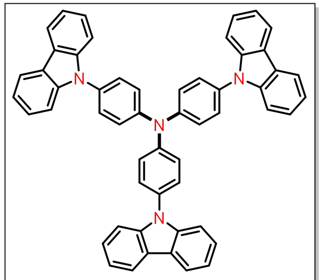

Mass	Intensity	Formula	Calculated Mass	Mass Difference [ppm]	DBE
741.29917	1026.00	C54 H37 N4	741.30127	-2.84	38.5

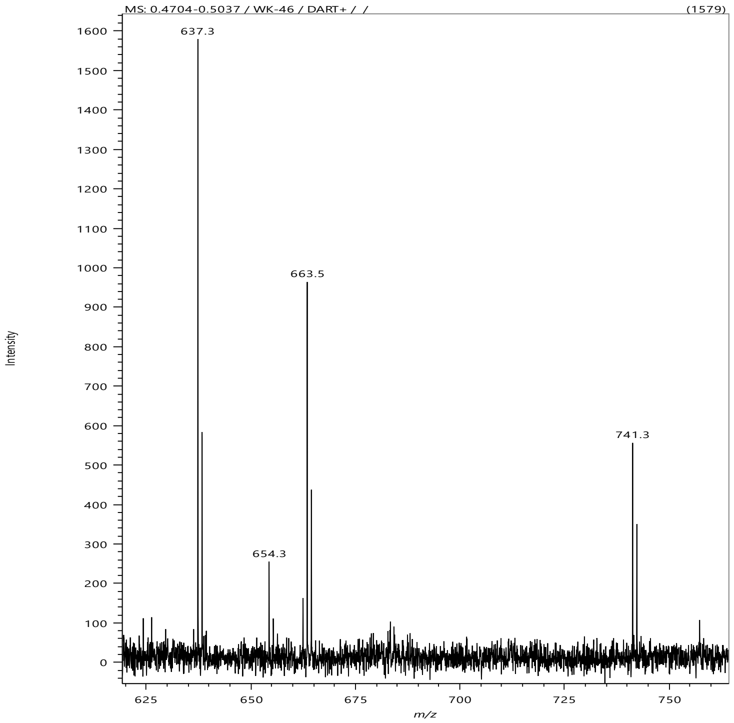

Supplementary Figure 189. HRMS Spectrum of **15**

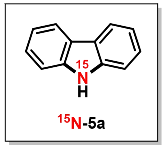

Elemental composition search on mass 169.08

m/z= 164.08-174.08

m/z	Theo. Mass	Delta (ppm)	RDB equiv.	Composition
169.0779	169.0778	0.65	8.5	$C_{12}H_{10}{}^{15}N$

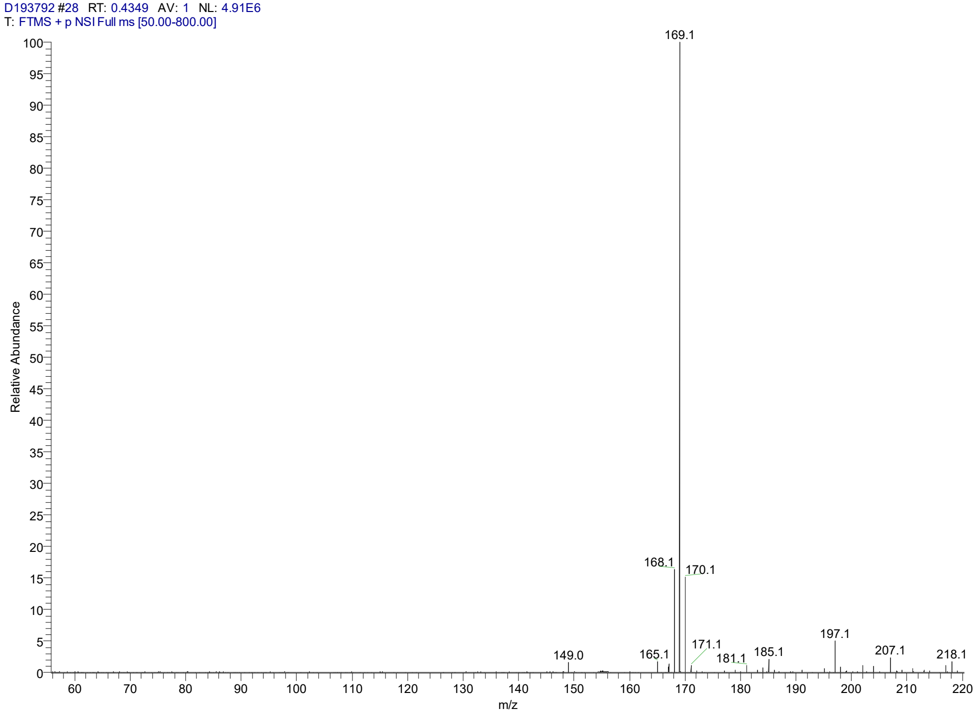

Supplementary Figure 190. HRMS Spectrum of **^{15}N-5a**

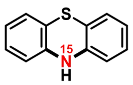

^{15}N-17

Elemental composition search on mass 201.05

m/z= 196.05-206.05

m/z	Theo. Mass	Delta (ppm)	RDB equiv.	Composition
201.0499	201.0499	0.24	8.5	$C_{12}H_{10}{}^{15}NS$
	201.0506	-3.32	4.5	$C_7H_9O_5N_2$
	201.0493	3.36	5.0	$C_5H_7O_4N_5$

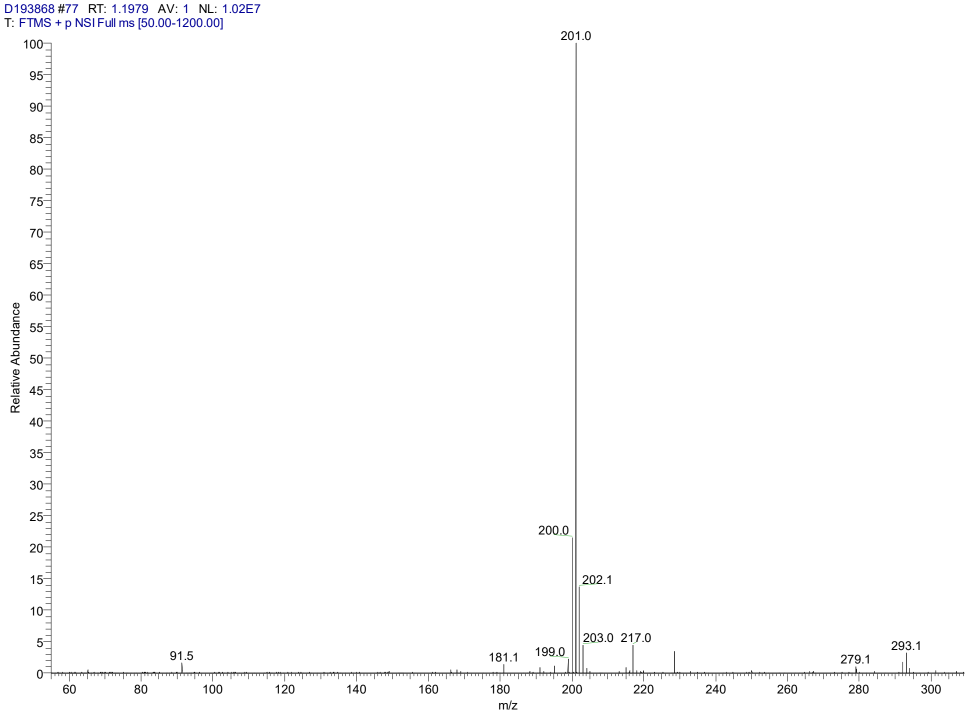

Supplementary Figure 191. HRMS Spectrum of **^{15}N-17**

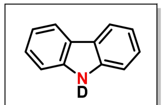

D-5a

m/z	Theo. Mass	Delta (ppm)	RDB equiv.	Composition
168.0789	168.0788	0.46	5.5	$C_9H_7{}^2H_2ONF$
	168.0792	-1.95	9.0	$C_{12}H_8{}^2HN$
	168.0792	-2.01	0.0	$C_6H_{13}O_4F$
	168.0784	3.15	0.0	$C_6H_{12}{}^2H_2O_3S$
	168.0781	4.78	4.0	$C_9H_{12}O_3$

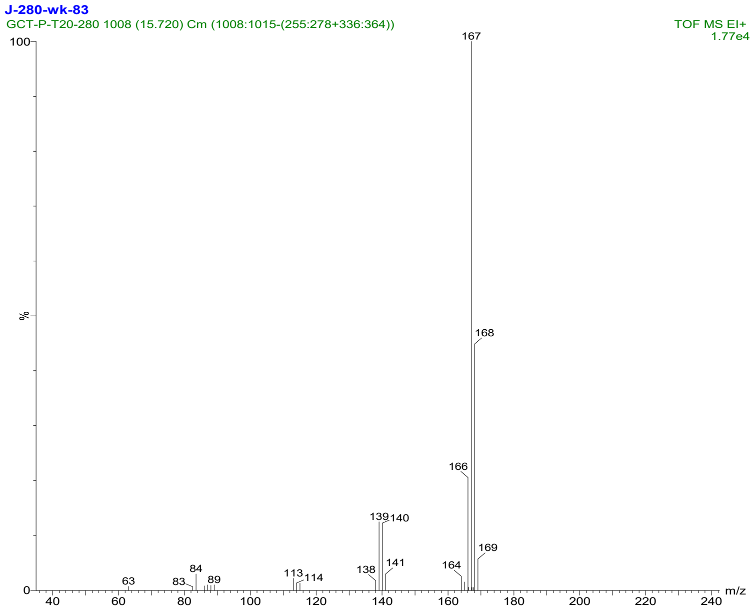

Supplementary Figure 192. HRMS Spectrum of **D-5a**

(IX) Proposed Mechanism

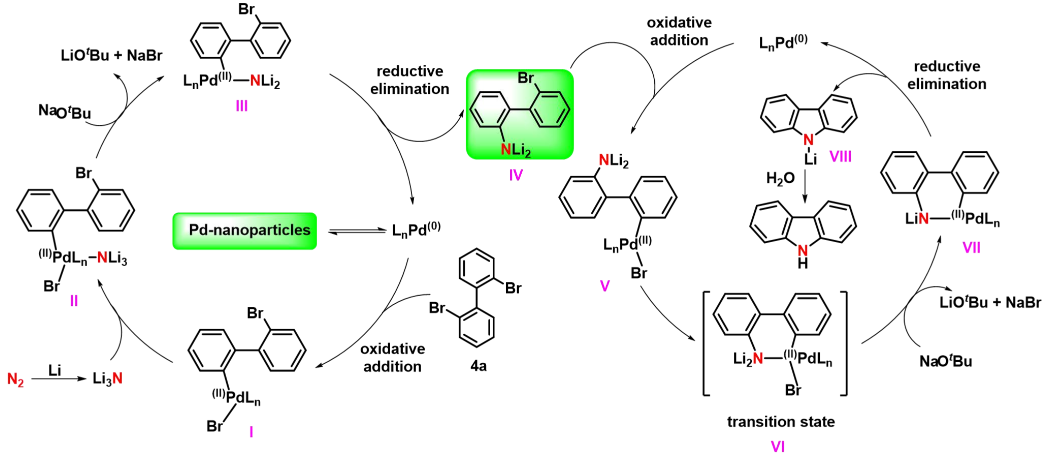

Supplementary Figure 193. Proposed reaction mechanism

Thus, the catalytic cycle to form carbazole as an example was proposed as shown in Supplementary Figure 193. Pd(II) species **I** formed via the oxidative addition of Pd(0) catalyst with **4a**. In the presence of lithium nitride, the Pd(II) species **II** was generated *via* the coordination of Li_3N to **I**. **III** formed through the ligand exchange with NaO^{t}Bu and **II**. **IV** was released through the reductive elimination of **III**. Meanwhile, Pd(0) catalyst was regenerated, thus fulfilling the first catalytic cycle. Subsequently, the intermediate **V** formed through the oxidative addition of Pd(0) catalyst with **IV**. Then Pd(II) species **VI** was generated. In the presence of NaO^{t}Bu, the Pd(II) species **VII** was further afforded through the ligand exchange. Afterwards, **VIII** was formed through the reductive elimination of **VII** and finally Pd(0) catalyst was regenerated again the catalytic cycle was fully completed. Finally, the desired carbazole product was produced by hydrolysis of **VIII**, which was partially supported by the hydrolysis of the reaction mixture with D_2O (please see Supplementary Figure 192).

(X) Supplementary References

1. Huang, X. H. & Buchwald, S. L. New Ammonia Equivalents for the Pd-Catalyzed Amination of Aryl Halides. *Org. Lett.* **3**, 3417-3419 (2001).
2. Csok, Z., Vechorkin, O., Harkins, S. B., Scopelliti, R. & Hu, X. L. Nickel Complexes of a Pincer NN_2 Ligand: Multiple Carbon-Chloride Activation of CH_2Cl_2 and $CHCl_3$ Leads to Selective Carbon-Carbon Bond Formation. *J. Am. Chem. Soc.* **130**, 8156-8157 (2008).
3. Field, J. E., Hill, T. J. & Venkataraman, D. Bridged Triarylamines: A New Class of Heterohelicenes. *J. Org. Chem.* **68**, 6071-6078 (2003).
4. Barham, J. P., John, M. P. & Murphy, J. A. Contra-thermodynamic Hydrogen Atom Abstraction in the Selective C−H Functionalization of Trialkylamine N-CH_3 Groups. *J. Am. Chem. Soc.* **138**, 15482-15487 (2016).
5. Bonhôte, P., Moser, J.-E., Humphry-Baker, R., Vlachopoulos, N., Zakeeruddin, S. M., Walder, L. & Grätzel, M. Long-Lived Photoinduced Charge Separation and Redox-Type Photochromism on Mesoporous Oxide Films Sensitized by Molecular Dyads. *J. Am. Chem. Soc.* **121**, 1324-1336 (1999).
6. Surry, D. S. & Buchwald, S. L. Selective Palladium-Catalyzed Arylation of Ammonia: Synthesis of Anilines as Well as Symmetrical and Unsymmetrical Di- and Triarylamines. *J. Am. Chem. Soc.* **129**, 10354-10355 (2007).
7. Roquet, S., Cravino, A., Leriche, P., Alévêque, O., Frère, P. & Roncali, J. Triphenylamine-Thienylenevinylene Hybrid Systems with Internal Charge Transfer as Donor Materials for Heterojunction Solar Cells. *J. Am. Chem. Soc.* **128**, 3459-3466 (2006).
8. Kwon, J., Kim, M. K., Hong, J.-P., Lee, W., Noh, S., Lee, C., Lee, S. & Hong, J.-I. 4,4',4"-Tris(4-naphthalen-1-yl-phenyl)amine as a multifunctional material for organic light-emitting diodes, organic solar cells, and organic thin-film transistors. *Org. Electron.* **11**, 1288-1295 (2010).
9. Suzuki, C., Hirano, K., Satoh, T. & Miura, M. Direct Synthesis of N-H Carbazoles via Iridium(III)-Catalyzed Intramolecular C−H Amination. *Org. Lett.* **17**, 1597-1600 (2015).
10. Xu, S., Shangguan, X., Li, H., Zhang, Y. & Wang, J. Pd(0)-Catalyzed Cross-Coupling of 1,1-Diboronates with 2,2′-Dibromobiphenyls: Synthesis of 9H-Fluorenes. *J. Org. Chem.* **80**,

7779-7784 (2015).

11. Bhatthula, B. K. G., Kanchani, J. R., Arava, V. R. & Subha, M. C. S. Total Synthesis of Carbazole Alkaloids. *Tetrahedron* **75**, 874-887 (2019).

12. Dalvi, B. A. & Lokhande, P. D. Copper(II) Catalyzed Aromatization of Tetrahydrocarbazole: An Unprecedented Protocol and Its Utility towards The Synthesis of Carbazole Alkaloids. *Tetrahedron Lett.* **59**, 2145-2149 (2018).

13. Xie, W., Zhao, M. & Cui, C. Cesium Carbonate-Catalyzed Reduction of Amides with Hydrosilanes. *Organometallics* **32**, 7440-7444 (2013).

14. Geng, W., Zhang, W. X., Hao, W. & Xi, Z. F. Cyclopentadiene−Phosphine/Palladium-Catalyzed Cleavage of C−N Bonds in Secondary Amines: Synthesis of Pyrrole and Indole Derivatives from Secondary Amines and Alkenyl or Aryl Dibromides. *J. Am. Chem. Soc.* **134**, 20230-20233 (2012).

15. Dai, B., Liu, N., Wang, L. & Ji, E. Site-Selective N-Arylation of Carbazoles with Halogenated Fluorobenzenes. *Synthesis* **48**, 737-750 (2016).

16. Dahl, T., Bang-Andersen, B., Nielsen, P. & Jørgensen, M. Palladium-Catalyzed Three-Component Approach to Promazine with Formation of One Carbon–Sulfur and Two Carbon–Nitrogen Bonds. *Angew. Chem. Int. Ed.* **47**, 1726-1728 (2008).
